# Specific Changes in *Arabidopsis thaliana* Rosette Lipids during Freezing Can Be Associated with Freezing Tolerance

**DOI:** 10.3390/metabo12050385

**Published:** 2022-04-23

**Authors:** Hieu Sy Vu, Sunitha Shiva, Thilani Samarakoon, Maoyin Li, Sujon Sarowar, Mary R. Roth, Pamela Tamura, Samuel Honey, Kaleb Lowe, Hollie Porras, Neema Prakash, Charles A. Roach, Morgan Stuke, Xuemin Wang, Jyoti Shah, Gary Gadbury, Haiyan Wang, Ruth Welti

**Affiliations:** 1 Kansas Lipidomics Research Center, Division of Biology, Kansas State University, 1717 Claflin Rd, Manhattan, KS 66506, USA; hieus.vu@utsouthwestern.edu (H.S.V.); ssunitha@ksu.edu (S.S.); thilani.samarakoon@gmail.com (T.S.); mrroth@ksu.edu (M.R.R.); ptamura@ksu.edu (P.T.); svhoney@gmail.com (S.H.); kalebalowe91@gmail.com (K.L.); hollieporras11@gmail.com (H.P.); prakashneema8@gmail.com (N.P.); drewroachcar@gmail.com (C.A.R.); morganarmbruster@icloud.com (M.S.); 2 Donald Danforth Plant Science Center, 975 N Warson Rd, St. Louis, MO 63132, USA; maoyinli2@gmail.com (M.L.); swang@danforthcenter.org (X.W.); 3 Department of Biology, University of Missouri-St. Louis, St. Louis, MO 63121, USA; 4 Department of Biological Sciences, University of North Texas, 1155 Union Circle #305220, Denton, TX 76203, USA; ssarowar@hotmail.com (S.S.); jyoti.shah@unt.edu (J.S.); 5 Department of Statistics, Kansas State University, Manhattan, KS 66506, USA; gadbury@ksu.edu (G.G.); hwang@ksu.edu (H.W.)

**Keywords:** *Arabidopsis thaliana*, freezing tolerance, lipids, lipidomics, mass spectrometry-based lipid analysis

## Abstract

While the roles of a few specific lipids in plant freezing tolerance are understood, the effect of many plant lipids remains to be determined. Acclimation of plants to non-freezing cold before exposure to freezing temperatures improves the outcome of plants, compared to plants exposed to freezing without acclimation. *Arabidopsis thaliana* plants were subjected to one of three treatments: (1) “control”, i.e., growth at 21 °C, (2) “non-acclimated”, i.e., 3 days at 21 °C, 2 h at −8 °C, and 24 h recovery at 21 °C, and (3) “acclimated”, i.e., 3 days at 4 °C, 2 h at −8 °C, and 24 h recovery at 21 °C. Plants were harvested at seven time points during the treatments, and lipid levels were measured by direct-infusion electrospray ionization tandem mass spectrometry. Ion leakage was measured at the same time points. To examine the function of lipid species in relation to freezing tolerance, the lipid levels in plants immediately following the freezing treatment were correlated with the outcome, i.e., ion leakage 24-h post-freezing. Based on the correlations, hypotheses about the functions of specific lipids were generated. Additionally, analysis of the lipid levels in plants with mutations in genes encoding patatin-like phospholipases, lipoxygenases, and 12-oxophytodienoic acid reductase 3 (*opr3*), under the same treatments as the wild-type plants, identified only the *opr3-2* mutant as having major lipid compositional differences compared to wild-type plants.

## 1. Introduction

Exposure of plants to freezing causes damage to plant cells. The damage often involves the destabilization of cellular membranes, particularly the plasma membrane [1]. Cold acclimation, or exposure of plants to cold, non-freezing temperatures, can greatly increase the ability of plants to tolerate freezing. Cold and freezing result in changes in plant lipid metabolism and lipid composition. When exposed to cold and/or freezing temperatures, plants increase the fatty acyl desaturation of membrane lipids, produce phosphatidic acid (PA), processively galactosylate and acylate galactolipids, and decrease levels of membrane structural lipids [2,3,4,5,6,7,8]. 

Analysis of loss-of-function mutants has yielded critical insights into the roles of several of these modifications. Inability to desaturate the fatty acyl chains of membrane lipids leads to poor ability to grow at above-freezing cold temperatures, and, thus, to undergo cold acclimation. Hugly and Somerville [2] demonstrated that loss of plastidic fatty acid desaturases 5 or 6 (FAD5 or FAD6) led to a chlorotic phenotype when plants were grown at 5 °C. The *fad3 fad7 fad8* triple mutant, which is defective in the synthesis of trienoic fatty acids, failed to recover from cold-induced photoinhibition [9]. Miquel et al. [3] showed that plants defective in extraplastidic FAD2 also grew very poorly in the cold (6 °C). These observations imply that unsaturated fatty acids are important for optimal plant growth in the cold. During freezing, polygalactosylated diacylglycerols, and likely triacylglycerols (TGs), have a beneficial effect on plant survival. Polygalactosylated diacylglycerols are plastidic lipid derivatives formed in response to freezing or near-freezing temperatures. The lack of polygalactosylated diacylglycerols in the *sensitive to freezing 2* (*sfr2*) mutant is associated with a poor outcome during freezing compared to wild-type plants [5,10]. Triacylglycerols also accumulate during freezing, perhaps to rid cells of unhelpful free fatty acids or other lipids. Lower expression of diacylglycerol acyltransferase 1 (DGAT1), which forms TGs, is associated with poorer outcomes in freezing [11,12]. 

On the other hand, the loss of phospholipase Dα (PLDα) or diacylglycerol kinases, which form PA, is beneficial for plants during freezing. In wild-type plants, PLDα produces about 50% of the PA formed during freezing. Antisense suppression of PLDα offers 2 to 4 degrees of freezing tolerance, indicating that higher levels of PA are associated with a poor outcome for plants subjected to freezing [4]. Diacylglycerol kinases also contribute to PA formation during freezing, perhaps particularly at early times after exposure to low temperature, and their loss is associated with improved freezing tolerance [11,13].

Using mass spectrometry, we have the ability to measure a wide range of lipid molecular species, but the potential roles of many of the lipid species produced in plant response to freezing are not well understood. In this work, we take a comprehensive look at changes in freezing-induced lipid composition throughout the freezing process. We identify lipid compositional changes in *Arabidopsis thaliana* during the cold acclimation and freezing process and use correlation analysis to associate the presence of specific lipids with good and poor outcomes, as measured by leaf ion leakage one day after the freezing challenge.

## 2. Results and Discussion

### 2.1. Experimental Design

**Figure 1:**
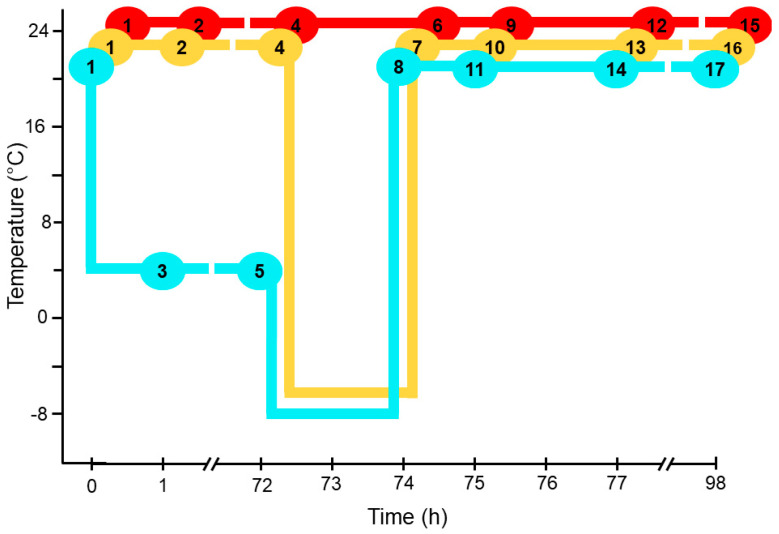
Plant trays and treatments. Plants entered the experiment at 28 days old. Each oval marks the time and temperature at which the correspondingly numbered tray was harvested. The colored lines connecting each circle indicate the temperature regimes applied to the tray. Red line is control treatment (C); yellow line is non-acclimated treatment (N); and blue line is acclimated treatment (A). Specifically, treatments applied to each tray are: tray **1** (no treatment), tray **2** (1 h at 21 °C), tray **3** (1 h at 4 °C), tray **4** (72 h at 21 °C), tray **5** (72 h at 4 °C), tray **6** (74 h at 21 °C), tray **7** (72 h at 21 °C and 2 h at −8 °C), tray **8** (72 h at 4 °C and 2 h at −8 °C), tray **9** (75 h at 21 °C), tray **10** (72 h at 21 °C, 2 h at −8 °C, and 1 h at 21 °C), tray **11** (72 h at 4 °C, 2 h at −8 °C, and 1 h at 21 °C), tray **12** (77 h at 21 °C), tray **13** (72 h at 21 °C, 2 h at −8 °C, and 3 h at 21 °C), tray **14** (72 h at 4 °C, 2 h at −8 °C, and 3 h at 21 °C), tray **15** (98 h at 21 °C), tray **16** (72 h at 21 °C, 2 h at −8 °C, and 24 h at 21 °C), tray **17** (72 h at 4 °C, 2 h at −8 °C, and 24 h at 21 °C).

Plants were grown in 72-well trays until the start of the experiment. In each tray, six wild-type plants were planted in randomized positions, and plants of other genotypes were planted in the remaining wells. Trays were subjected to one of 17 treatments, which created three experimental time courses (Figure 1). All time courses started with 28-day-old plants grown at 21 °C. The trays of plants in the control time course were held at a growth temperature of 21 °C throughout the experiment, with trays sampled for lipid analysis and ion leakage at 0, 1, 72, 74, 75, 77, and 98 h from the start of the experiment (Figure 1). The “non-acclimated” time course started with plants grown at 21 °C (0 h) with additional sampling at 1 h and 72 h at 21 °C, before switching to −8 °C for 2 h, with sampling at the end of the freezing treatment (74 h). The “acclimated” time course started with plants grown at 21° (0 h), switched to 4 °C, with sampling for lipid analysis at 1 h and 72 h, before switching to −8 °C for 2 h, with sampling for lipid analysis at 74 h. In both the non-acclimated and acclimated time courses, plants were grown at 21 °C for 24 h after the freezing treatment with sampling after 1 h (75 h), 3 h (77 h), and 24 h (98 h) into the 21 °C period. The experiment was carried out three times, creating three statistical blocks. 

### 2.2. Plant Response to Treatment

**Figure 2:**
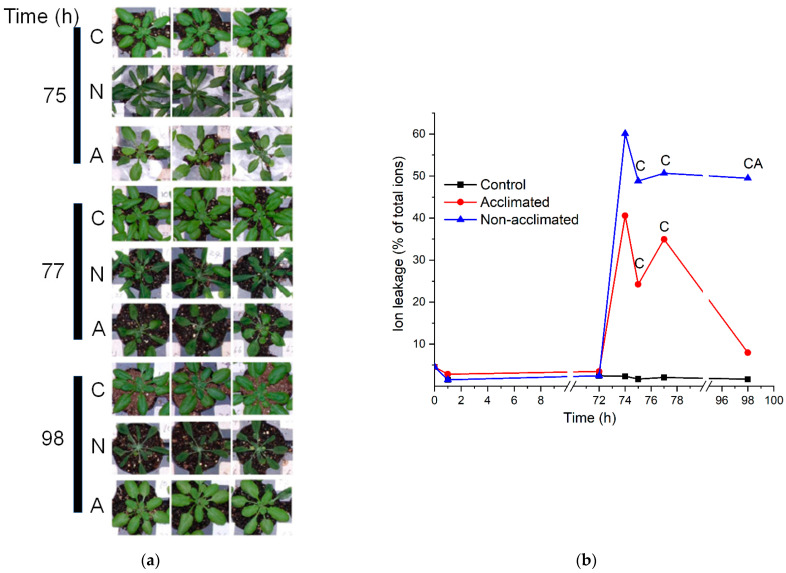
Plant appearance following freezing treatments and ion leakage throughout experiment. The freezing treatment occurred between 72 and 74 h. (**a**). Photographs depict three wild-type plants from control (C), non-acclimated frozen (N), and acclimated frozen (A) trays, 1 h (75 h into experiment), 3 h (77 h), and 24 h (98 h) after the freezing treatment. (**b**). Ion leakage as a percentage of total ions for plants undergoing each of the treatment courses: control, acclimated, and non-acclimated. *n* = 18. The symbol “C” means that the value for acclimated or non-acclimated plants is significantly different from the control plants, whereas the symbol “A” means that the value for non-acclimated plants is significantly different from acclimated plants (Table S2).

Examination of the wild-type plants after the freezing treatment indicated that plants subjected to freezing treatment sustained damage (Figure 2a), and damage to both non-acclimated and acclimated plants was clearly visible at 1 and 3 h after the freezing treatment. However, by 24 h after the freezing treatment, acclimated plants appeared very similar to control plants, while non-acclimated plants still appeared to be damaged (Figure 2a). The visible results were confirmed by analysis of ion leakage (Figure 2b and Tables S1 and S2). While there was substantial variability in ion leakage among the plants subject to the same treatment, both non-acclimated and acclimated plants experienced high levels of ion leakage after the −8 °C freezing treatment. However, at 24 h after freezing, the non-acclimated plants had significantly higher ion leakage than both control and acclimated plants, which had recovered nearly completely (Figure 2b).

### 2.3. Overview of Lipid Changes in Wild-Type Plants

**Figure 3:**
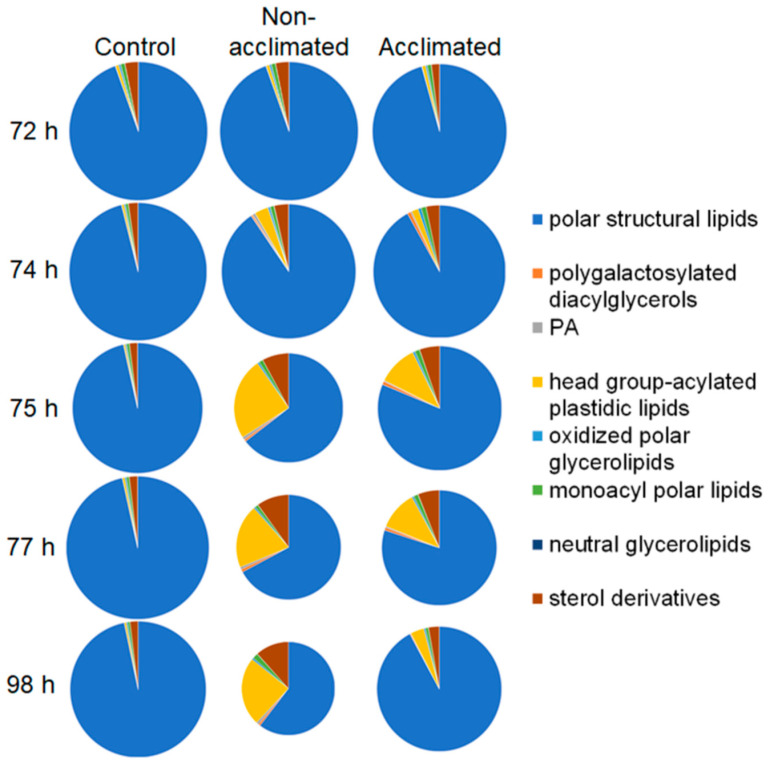
Changes in levels and proportions of the total amounts of each of the major lipid groups in wild-type Arabidopsis rosettes as a function of freezing treatment and time. Size of slices and areas of each pie are proportional to the total mass spectral signals for each group normalized to internal standards and dry mass of the rosette. Treatments and conditions at each time (*n* = 18) are shown in Figure 1.

The lipid composition was measured using electrospray ionization triple quadrupole mass spectrometry and multiple (parallel) reaction monitoring in direct-infusion mode, as previously described [14,15]. Data are reported as normalized mass spectral intensities, by normalizing to internal standards. The data allow for the comparison of lipid levels from sample to sample, but absolute amounts of some lipids may not be accurate due to the use of a limited number of internal standards (Table S3) and varying responses of the mass spectrometer for individual lipid molecular species. For example, MGDG and DGDG are over-represented in absolute amounts, due to the high mass spectral response of the polyunsaturated versions of these compounds when detected with neutral loss of the ammoniated head groups, compared to that of the saturated internal standards. An overview of the changes in the composition of major lipid groups, starting from 72 h (when no treatment had occurred in non-acclimated plants and acclimated plants had completed the 3-day cold acclimation), through a 2-h freezing treatment to 74 h, and at 1 h (75 h), 3 h (77 h), and 24 h (98 h) after freezing, is shown in Figure 3. The data are provided in Table S1 with the statistical significance of changes detailed in Table S2. Nomenclature has been updated [16]; comparisons between current and previous nomenclatures are given in Table S4.

Changes due to cold acclimation (72-h time point, control/non-acclimated vs. acclimated) are relatively minor, with the largest changes in lipid levels being a 271% increase in polygalactosylated diacylglycerols, a 45% increase in neutral glycerolipids, and a 41% decrease in sterol derivatives. Freezing (between 72 h and 74 h) resulted in larger changes, and even larger changes occurred at 1 h and 3 h after freezing (75 h and 77 h). Overall, changes were greater in non-acclimated than in acclimated plants, both with regard to the overall loss of lipid and in relation to changes in the proportions of the lipid groups. In the first 3 h after freezing, differences in lipid levels in non-acclimated vs. acclimated plants were most apparent at 1 h after freezing (75 h). The highest fold changes in lipid levels, over those of control plants, at 75 h were 31-fold and 53-fold increases in polygalactosylated diacylglycerols in non-acclimated and acclimated plants, respectively, 17-fold and 7-fold increases in phosphatidic acid in non-acclimated and acclimated plants, respectively, and 46-fold and 26-fold increases in head group-acylated plastidic lipids in non-acclimated and acclimated plants, respectively. At 24 h after the freezing treatment (98 h), the non-acclimated plant lipid composition remained highly perturbed, whereas the lipid composition of acclimated plants had moved toward the control composition. Most notably, while the total amount of lipids, normalized to dry mass, in the acclimated plants at 98 h, was 83% as high as that of the control plants, the total lipid amount in the non-acclimated plants 24 h after freezing was only 46% of the control level.

### 2.4. Correlation Analysis as a Predictor of the Role of Lipids in Freezing Response

**Figure 4:**
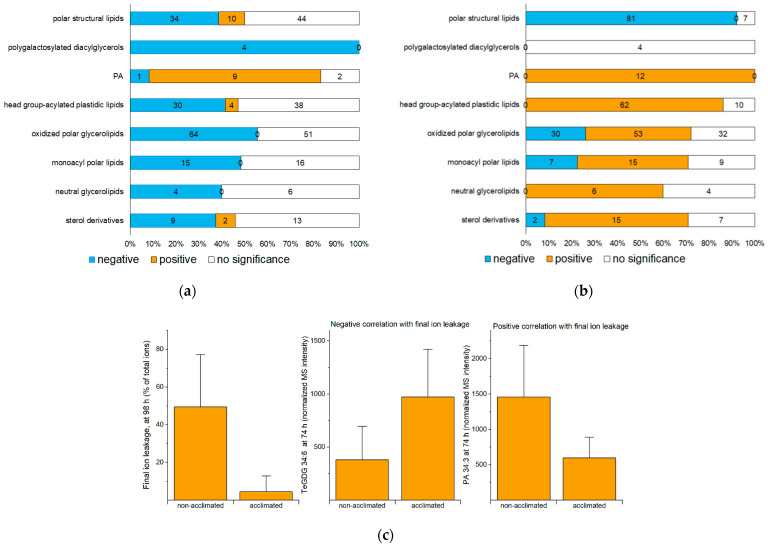
Positive and negative correlations of lipid levels with ion leakage at the final (98-h) time point. Positive correlation indicates that higher levels of the lipid are associated with more ion leakage (i.e., a deleterious effect), while negative correlations indicate that higher levels of the lipid are associated with lower ion leakage (potentially a beneficial effect). (**a**) Number of lipid species at 74 h (at the end of the freezing treatment) with positive and negative correlations with 98-h ion leakage. (**b**) Number of lipid species at 98 h (24 h after the end of the freezing treatment) with positive and negative correlations with 98-h ion leakage. (**c**) Examples of lipid levels at 74 h showing negative (TeGDG 34:6, **middle**) and positive (PA 34:3, **right**) correlation with final ion leakage (**left**) at the final time point (98 h). Error bars in (**c**) indicate standard deviation. Mass spectral (MS) intensities are normalized to internal standards and dry plant mass. *n* = 18.

Compared to non-acclimated plants, acclimated plants have a better outcome, as defined by decreased ion leakage from the rosette leaves, 24 h after freezing (Figure 2b). To identify lipids and lipid metabolic processes associated with a good outcome or a poor outcome after freezing, rosette lipid levels in non-acclimated and acclimated plants sampled at 72, 74, 75, 77, and 98 h were correlated with the ion leakage on the final trays. The Spearman’s correlations of lipid levels with ion leakage, along with average values and fold-changes compared to control of each lipid at each time point, are provided in Table S5. Figure 4a,b show the number of significant positive and negative correlations between ion leakage at 98 h and levels of lipid molecular species within each lipid group at 74 h and 98 h, respectively. Figure S1 shows numbers of correlations between ion leakage at 98 h and lipids at 72, 75, and 77 h. Because acclimated plants have lower ion leakage at the final time point, a negative correlation of a lipid level with ion leakage is synonymous with a higher level of lipid in acclimated plants compared to non-acclimated plants, whereas a positive correlation indicates that the lipid level is higher in non-acclimated plants than acclimated plants. 

The use of correlation analysis for identifying lipid molecular species associated with freezing tolerance was pioneered by Degenkolbe et al. [17], who correlated levels of lipids formed in 14-day acclimated plants and non-acclimated plants of 15 Arabidopsis accessions with different tolerances to freezing [18]. This is in contrast to the current analysis of plants subjected to a 2-h freezing treatment with or without a preceding 3-day cold acclimation. Our thought is that the 2-h freezing treatment manifests the differences in lipid metabolic potential generated during the acclimation period (or not generated in the non-acclimated plants). Degenkolbe and coworkers [17] identified lipids correlated with freezing tolerance, which are what we are here describing as “negatively correlated with ion leakage”. Another difference between the two studies is in the varieties of lipids analyzed. Degenkolbe et al. [17] analyzed many polar lipids and more triacylglycerols and diacylglycerols (DGs) than the current study, while the current analysis includes a wide range of head group-acylated and oxidized compounds, phosphatidic acids, and sterol derivatives not included in the Degenkolbe et al. study [17]. 

A comparison of correlations observed between ion leakage at 98 h and levels of lipids at 74 h with data on the role of lipids from mutants suggests that this correlation can be used to predict the role of the lipids in freezing tolerance. For example, 74-h levels of four out of four analyzed molecular species of polygalactosylated diacylglycerols, which are known from mutant analysis to play a beneficial role in freezing survival [5,10] (Section 2.6), display a negative correlation with ion leakage. An example shows the 74-h levels of tetragalactosyldiacylglycerol 34:6 (TeGDG 34:6) in relation to 98-h ion leakage (Figure 4c). The 74-h levels of most PAs, which are known from mutant analysis to play a detrimental role in freezing tolerance [4,11,13] (Section 2.7), are positively correlated with 98-h ion leakage. An example showing the positive correlation of the 74-h value for PA 34:3 with ion leakage at the final time point is also shown in Figure 4c. We infer that the time point immediately post-freezing (74 h) is the optimal time for identification of the roles of specific lipids in freezing tolerance by comparing non-acclimated and cold-acclimated plants and suggest that this is because, at that time, many changes potentiated during acclimation are expressed.

Different than the relationships between lipid levels at 74 h and ion leakage at 98 h, levels of many lipids other than structural polar lipids, when measured at 98 h, display a positive correlation with ion leakage at 98 h. Not surprisingly, high levels of structural polar lipids (81/88 measured molecular species) are negatively correlated with ion leakage, when both are measured at 98 h (24 h post-freezing). Lipids other than structural polar lipids decrease as acclimated plants recover from exposure to freezing, and their retention at 98 h is associated with a poor outcome. In the following sections, more detail on changes of specific lipid species and the positive or negative correlations of their levels at 74 h with ion leakage at the final time point is presented.

### 2.5. Structural Polar Lipids

**Figure 5:**
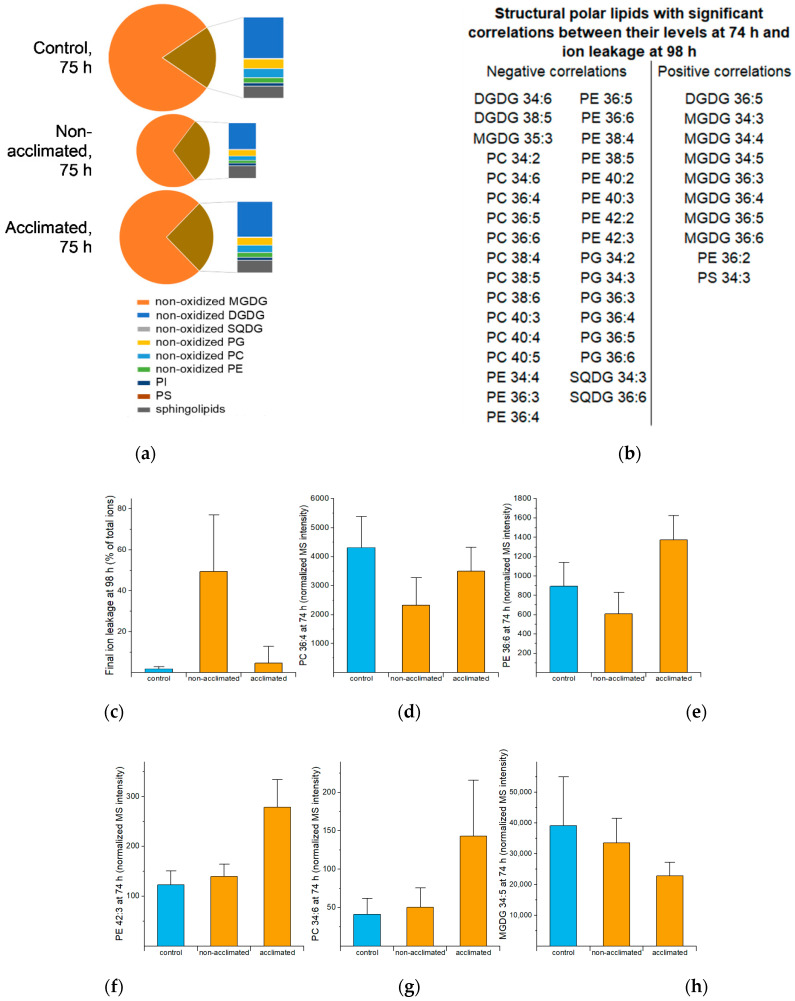
Structural polar lipid composition and correlations in response to freezing. (**a**) Structural lipid class composition at 75 h. The area of the pie is proportional to the total normalized MS intensity for structural polar lipids, and the brown area, which is expanded to the right of the pie, represents all the lipids except MGDG. (**b**) Lipids with significant correlations between levels at 74 h and ion leakage at 98 h. (**c**) Ion leakage at 98 h (same data as Figure 4c). (**d**) Levels of PC 36:4 at 74 h. (**e**) Levels of PE 36:6 at 74 h. (**f**) Levels of PE 42:3 at 74 h. (**g**) Levels of PC 34:6 at 74 h. (**h**) Levels of MGDG 34:5 at 74 h. In (**b**), only the primary annotations are shown; other annotation options are included in Table S4. Error bars in (**c**–**h**) indicate standard deviation. *n* = 18. MS intensities are normalized to internal standards and dry plant mass.

As shown in Figure 3, total structural polar lipids decrease in overall amount and as a fraction of the total lipid pool in the early hours (0 to 3 h) after freezing. This can also be seen in the time courses for many structural polar lipids in Figure S2. Figure 5a shows the composition of the structural polar lipid group at 75 h (1 h after freezing), which is when the compositional differences between non-acclimated and control plants are the greatest. Among control, non-acclimated, and acclimated rosettes, the differences in the percentage of each lipid class within the structural polar lipid group are relatively modest; the main difference among the three groups is in the total amount of the structural lipid pool. Comparing the structural polar lipid levels, as measured by total normalized mass spectral intensity, among the three treatments at 75 h, non-acclimated plants had only 46% of the lipid levels of the control plants, while acclimated plants had 76% of the amount of the control level. 

Figure 5b shows the structural polar lipids with levels at 74 h significantly correlated with ion leakage at 98 h. Lipids with negative correlations include 11 phosphatidylcholines (PCs), 11 phosphatidylethanolamines (PEs), 6 phosphatidylglycerols (PGs), 2 sulfoquinovosyldiacylglycerols (SQDGs), 2 digalactosyldiacylglycerols (DGDGs), and 1 monogalactosyldiacylglycerol (MGDG). DGDG 34:6 and PC 38:4 are found as negatively associated with final ion leakage here and also are on the list of lipids that Degenkolbe et al. [17] found to be associated with freezing tolerance. Lipid levels at 74 h with positive correlations with final ion leakage include seven MGDG species, one DGDG, one PE, and one phosphatidylserine (PS). Bar graphs of example lipids in Figure 5c–h show examples of the relationships between the lipid levels and the ion leakage outcome. Although control sample data were not used in the correlation analysis, they are shown for comparison. Levels of lipids in the group with negative correlations (e.g., Figure 5d–g) tend to increase or be maintained during acclimation and freezing, while lipids in the group with positive correlation (e.g., Figure 5h) decrease during acclimation and freezing. 

The group of structural polar lipids with negative correlations includes some of the major PC and PE molecular species, exemplified by PC 36:4 (Figure 5d) and PE 36:6 (Figure 5e). The lipids, with levels at 74 h negatively correlated with final ion leakage, include twelve 38C, 40C, or 42C PC or PE molecular species, exemplified by PE 42:3 (Figure 5f), which we hypothesize to be beneficial in withstanding freezing. This coincides with Degenkolbe and coworkers’ prediction about two 38C molecular species [17]. The 38C, 40C, and 42C PC and PE species that we identified contain 20C, 22C, or 24C very long chains in combination with 18C acyl chains [19]. Chen et al. [20] recently demonstrated that higher levels of very long-chain fatty acids (up to 22C) are made when 3-ketoacyl-CoA synthase 1 (KCS1) expression is higher, and that higher levels of very long-chain fatty acid levels provide increased chilling tolerance. These authors also showed that overexpression of KCS1 increased levels of very long-chain fatty acids and decreased ion leakage [20]. If the very long-chain fatty acids made by KCS1 are incorporated in PC and PE, the genetic manipulation of very long-chain fatty acid levels supports the hypothesis that these species are beneficial in response to freezing. Because phospholipids with longer chains have higher phase transition temperatures, i.e., form more rigid membranes, it might seem counterintuitive that they would be beneficial at low temperatures, but one possibility is that the long chains reduce the propensity of membranes to undergo membrane-destabilizing hexagonal phase formation, which can be induced by dehydration at low temperatures. This notion may better explain the benefit of the very long chains in freezing than at the chilling temperatures examined by Chen et al. [20], as hexagonal phase formation is usually associated with the formation of ice crystals [1]. 

PC 34:6 is associated with low ion leakage and has distinctly different variations in levels with time, compared to most structural lipids. Whereas most structural lipids decrease during and following freezing (Figure 3) and some increase slightly, (e.g., PE 42:3, Figure 5f), PC 34:6 increases more dramatically and is 1.2-fold and 3.5-fold higher in non-acclimated and acclimated rosettes at 74 h, respectively, compared to control rosettes (Figure 5g), and 4.6- and 10.6-fold higher at 75 h (Table S5 and Figure S2, p. 2Q). The inclusion of 16:3 in the fatty acid combination making up PC 34:6, 18:3, and 16:3, suggests that it derives from metabolism in the chloroplast [21], rather than by de novo synthesis or acyl editing in the endoplasmic reticulum, and the association with low ion leakage suggests that the metabolism forming this species is beneficial. 

Lipid molecular species displaying a positive correlation between final ion leakage and their levels at 74 h include six not-fully-unsaturated MGDG species, one not-fully-unsaturated DGDG species, one fully-unsaturated MGDG (MGDG 36:6), one PE, and one PS (Figure 5b). Unsaturation of galactolipids and phospholipids increases during acclimation, and the less-than-fully-unsaturated MGDGs tend to decrease in amount during acclimation. Failure to reduce these species may be associated with a poor outcome, as demonstrated for desaturase mutants in the cold [2,3]. On the other hand, galactosylation of MGDG to form DGDG and polygalactosylated lipids and acylation of MGDG to form acylated MGDG (acMGDG) also decrease MGDG levels and these reactions are potentiated by cold acclimation. Thus, it is possible that the higher level of MGDG 36:6, (i.e., MGDG 18:3/18:3), the second most common MGDG molecular species, in non-acclimated plants (1% lower than control), compared to acclimated plants (21% lower than control) (Table S5), is due to reduced activation of galactosylation and head group-acylation pathways during the 2-h freezing period in non-acclimated plants.

### 2.6. Polygalactosylated Diacylglycerols

**Figure 6:**
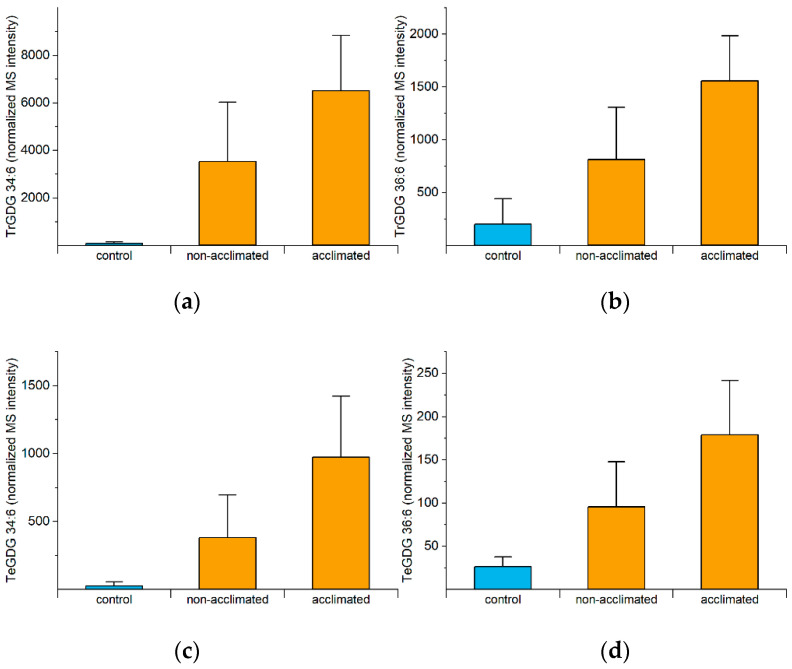
Levels of TrGDGs and TeGDGs at 74 h (immediately after freezing). All measured molecular species of polygalactosylated diacylglycerols are significantly negatively correlated with final (98-h) ion leakage (Figure 4a). Correlation analysis was performed on acclimated and non-acclimated plants (Table S5). (**a**) TrGDG 34:6, (**b**) TrGDG 36:6, (**c**) TeGDG 34:6, (**d**) TeGDG 36:6. Error bars indicate standard deviation. *n* = 18. MS intensities are normalized to internal standards and dry plant mass.

As mentioned previously, the formation of polygalactosylated diacylglycerols during freezing, mediated by SFR2, is beneficial, as confirmed by mutant analysis [5,10]. The reaction catalyzed by SFR2 is the transfer of a galactose moiety from an MGDG to a growing polygalactosyldiacylglycerol, resulting in a polygalactosyldiacylglycerol, such as DGDG, trigalactosyldiacylglycerol (TrGDG), or TeGDG, and a diacylglycerol (DG). Levels of TrGDGs and TeGDGs increase rapidly in acclimated plants, reaching peak values at 74 or 75 h (0 to 1 h post-freezing), while levels in non-acclimated plants increase later (Figure S3). Differences between non-acclimated and acclimated plants are the largest right at the end of the freezing period (74 h). Figure 6 shows the 74-h levels of the four common polygalactosylated galactolipid molecular species, which are all negatively correlated with final ion leakage (Figure 4a). 

### 2.7. Phosphatidic Acid

It has been shown previously that about half of the PA formed in freezing is formed by phospholipase Dα1, mainly by hydrolysis of PC, and that suppression of that enzyme improves plant freezing survival [4]. In contrast, the expression of phospholipase Dδ is associated with freezing tolerance [22,23]. The two phospholipase Ds produce distinct PA pools, which are likely in different intracellular locations, made with different timing, and have different functions. The amount of PA formed by phospholipase Dδ during freezing is also considerably smaller than that made by phospholipase Dα1, with the main production of PA by phospholipase Dδ occurring during the post-freezing recovery period [23]. In addition to phospholipase Ds, diacylglycerol kinases (DGKs), which add a phosphate group to DG to form PA, contribute to PA formation, and knockout mutants, *dgk2*, *dgk3*, and *dgk5*, show better freezing tolerance than wild-type plants [11,13]. 

**Figure 7:**
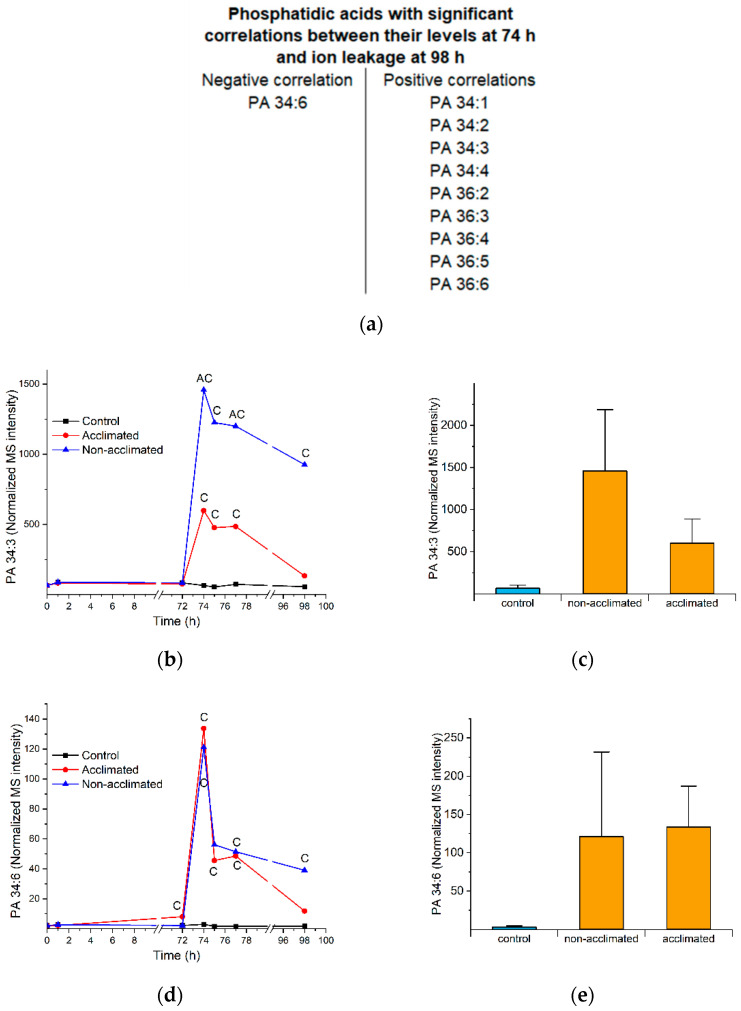
Phosphatidic acid levels and correlations in response to freezing. (**a**) List of PAs with levels at 74 h that have significant negative and positive correlations with ion leakage at 98 h. (**b**) Time course of levels of PA 34:3 in control, non-acclimated, and acclimated rosettes. (**c**) Levels of PA 34:3 at 74 h in control, non-acclimated, and acclimated plants. (**d**) Time course of levels of PA 34:6 in control, non-acclimated, and acclimated rosettes. (**e**) Levels of PA 34:6 at 74 h in control, non-acclimated, and acclimated plants. Panels (**b**,**d**) are the same graphs as Figure S4, pp. 4B and 4F. In panels (**b**,**d**), “C” indicates that the lipid level in non-acclimated or acclimated plants is significantly different than the control level, and “A” indicates that the lipid level in non-acclimated plants is significantly different than the level in acclimated plants (Table S2). In panels (**c**,**e**), the error bars indicate standard deviation. *n* = 18. MS intensities are normalized to internal standards and dry plant mass.

Whereas PA levels did not change significantly during cold acclimation, the level of total PA at 74 h (immediately after freezing) in non-acclimated plants was 17 times higher than control levels and in acclimated plants was 8 times higher than control levels (Table S5). Similar large fold changes occurred for all major PA species, and most PA molecular species were positively correlated with ion leakage at the final time point (Figure 7a and Figure S4, Table S5). The time course for a representative positively correlated PA, PA 34:3, is shown in Figure 7b,c, and the time courses for other PA species are shown in Figure S4. In agreement with results from genetic manipulation of phospholipase Dα1 and DGK levels, the positive correlation means high levels of most PAs present at the end of freezing are associated with a poor outcome. The lack of correlation between most PA levels and the effect of phospholipase Dδ is expected, given the relatively small amount of PA produced by phospholipase Dδ during freezing [22]. The poor outcome associated with high levels of PA may be related to potentially membrane-destabilizing effects of PA (discussed in [4]) or to PA signaling [24].

The only PA species at 74 h with a negative correlation with ion leakage at 98 h is PA 34:6 (Figure 4a and Figure 7a,d,e). Thus, we hypothesize that this molecular species is a marker of freezing tolerance. Welti et al. [4] also showed that PA 34:6 is formed during freezing and that, unlike the formation of most PA molecular species, PA 34:6 formation is independent of phospholipase Dα1 expression. Like PC 34:6, PA 34:6 has an 18:3_16:3 combination, and at least the 16:3 is likely to derive from MGDG, or possibly DGDG, as these galactolipids are the only lipid classes with significant amounts of 16:3. In wounding, the formation of PA 34:6 is tightly coupled to the formation of polygalactosylated diacylglycerols, which are formed by the transfer of a galactose from MGDG to a growing chain of galactolipids by SFR2, leaving behind a diacylglycerol [14]. The DG can be phosphorylated to form PA, (e.g., PA 34:6). The DG can also be acylated to form triacylglycerol (TG) [5], although TG produced from acylation of DG 34:6 is a relatively minor component of total leaf TG [25].

### 2.8. Head Group-Acylated Plastidic Lipids

Head group-acylated plastidic (chloroplast) lipids include head group-acylated MGDG (acMGDG), head group-acylated DGDG (acDGDG), and head group-acylated PG (acPG). Head group-acylated lipids are formed by the action of acylated galactolipid-associated phospholipase 1 (AGAP1), which transfers an acyl chain from a DGDG or MGDG to the head group of another lipid, resulting in a head group-acylated plastidic lipid and a di- or mono-galactosylmonoacylglycerol (DGMG or MGMG) [26]. While head group-acylated plastidic lipids are widespread throughout the plant kingdom, the degree of fatty acyl oxidization within the class varies among plant species and accessions [7,26,27]. In the Columbia−0 accession of Arabidopsis, used in this work, head group-acylated plastidic lipids, formed under wounding stress and during bacterial infections, tend to be primarily oxidized molecular species, but oxidation is less prevalent under freezing stress [7,28]. 

In the current work, 67 acMGDG species, 4 acDGDG species, and 1 acPG species were measured. acMGDG was identified by a head group fragment that included the acyl chain. Because we targeted head-group fragments undergoing water loss (and only formed by head groups with oxidized fatty acids) when detecting head groups acylated with oxidized fatty acids, we were able to differentiate oxidized lipids from normal chain lipids of the same nominal mass, and, thus, our head group identifications are unequivocal. However, in the diacylglycerol portion of the acMGDG, there are some fatty acid combinations that share a nominal mass, leading to some ambiguity in our ability to annotate molecular species associated with particular MRM transitions. Thus, we have divided our head group-acylated lipid group into three sub-groups: non-oxidized acylated plastidic lipids (7 species), oxidized acylated plastidic lipids (54 species), and ambiguous acylated plastidic lipids (11 species). “Oxidized” indicates lipid species with one or more oxidized fatty acyl chains, and “ambiguous” indicates uncertainty with regard to whether they contain one or more oxidized fatty acids (Table S5). In this classification, “molecular species” refers to a head group-acylated plastidic lipid represented by a particular MRM transition. In some cases, a single transition may represent more than one lipid, and in one case, two transitions represent the same lipid. 

**Figure 8:**
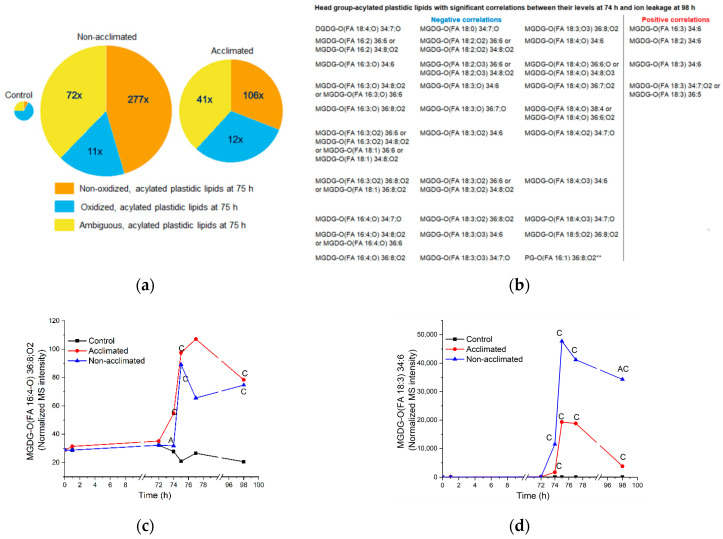
Head group-acylated plastidic lipid levels and correlations in response to freezing. (**a**) Pie graph of head group-acylated plastidic lipids from control, non-acclimated, and acclimated plants. (**b**) List of head group-acylated plastidic lipids with levels at 74 h that have significant negative and positive correlations with ion leakage at 98 h. (**c**) Time course of levels of MGDG-O(FA 16:4;O) 36:8;O2 in control, non-acclimated, and acclimated rosettes. (**d**) Time course of levels of MGDG-O(FA 18:3) 34:6 in control, non-acclimated, and acclimated rosettes. In (**a**), the size of the pies reflects the size of each acylated lipid pool, and the values in the non-acclimated and acclimated pies indicate the fold increases of each sub-class, compared to the levels in control plants. Panels (**c**,**d**) are the same graphs as Figure S5, pp. 5I and 5D. In panels (**c**,**d**), “C” indicates that the lipid level in non-acclimated or acclimated plants is significantly different than the control level, and “A” indicates that the lipid level in non-acclimated plants is significantly different than the level in acclimated plants (Table S2). *n* = 18. MS intensities are normalized to internal standards and dry plant mass.

Levels of head group-acylated plastidic lipids began to increase rapidly after freezing, with most reaching peak values at 75 h (Figure S5). At 75 h, head group-acylated plastidic lipids account for 24% of the normalized MS signal from non-acclimated rosettes and 10% of the signal from acclimated rosettes (Figure 3 and Table S5). Figure 8a compares the sizes of the control, non-acclimated, and acclimated head group-acylated plastidic lipid pools at 75 h. At 75 h, the pool of head group-acylated plastidic lipids was 46× higher in non-acclimated rosettes and 26× times higher in acclimated rosettes compared to control plants. Non-oxidized molecular species rose the most, increasing their proportion from 8% of the control head group-acylated plastidic lipids to 45% of the group in non-acclimated plants and 31% in acclimated plants.

There are 30 head group-acylated “molecular species” with negative correlations between levels at 74 h and final ion leakage at 98 h and 4 head group-acylated “molecular species” with positive correlations (Figure 4a). Of the 30 molecular species with significant negative correlations between lipid levels at 74 h and final ion leakage, 27 are oxidized acylated plastidic lipids and 3 are ambiguous acylated plastidic lipids. On the other hand, of the four species with significant positive correlations, three are non-oxidized acylated plastidic lipids and one is an ambiguous acylated plastidic lipid. This information allows us to hypothesize that certain oxidized head group-acylated plastidic lipids are associated with reduced ion leakage 24 h after freezing, while certain non-oxidized acylated plastidic lipids, which increase to particularly high levels in non-acclimated plants, are associated with a poor outcome. Figure 8c,d shows examples of time courses of head group-acylated MGDGs with a negative (MGDG-O(FA 16:4,O2) 36:8,O2) and a positive (MGDG-O(FA 18:3) 34:6) correlation between their levels at 74 h and ion leakage at 98 h. In general, the positive correlation of the non-oxidized molecular species at 74 h with final ion leakage is more clear-cut than the negative correlation of the oxidized molecular species with final ion leakage. This is apparent in comparing panels c and d in Figure 8. MGDG-O(FA 18:3) 34:6 is about 7-fold higher in non-acclimated plants than in acclimated plants at 74 h (Figure 8d), while the difference between non-acclimated and acclimated levels of MGDG-O(FA 16:4;O2) 36:8,O2 (less than 2-fold) at 74 h is smaller (Figure 8c).

### 2.9. Oxidized Polar Diacylglycerolipids

Several different oxidized fatty acids, identified by mass spectrometry by the number of carbons, double bond equivalents, and the number of oxygens, have been identified as components of oxidized polar diacylglycerolipids, as well as components of oxidized head group-acylated plastidic lipids. In the Columbia-0 accession of Arabidopsis, oxophytodienoic acid (OPDA; 18:4;O2) and dinor-oxophytodienoic acid (dnOPDA; 16:4;O2) are typically the most abundant oxidized fatty acyl chains, but other detected oxidized acyl chains include hydroxy fatty acids (e.g., 18:3;O), ketols (e.g., 18:3;O2), and others, some of which are of unknown structure [14,29,30]. Arabidopsides are a special class of oxidized galactolipids containing OPDA and dnOPDA.

**Figure 9:**
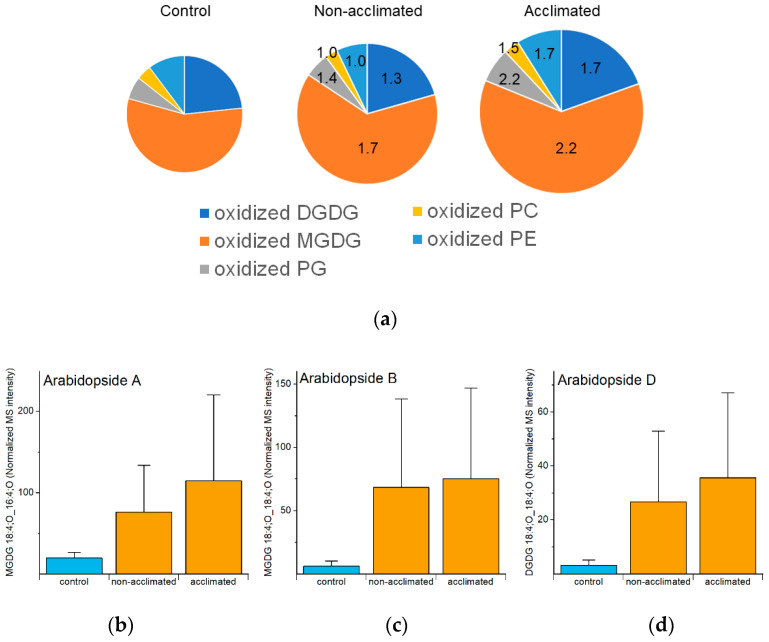
Oxidized polar diacylglycerolipid levels and correlations in response to freezing. (**a**) Pie graph of levels of polar diacylglycerolipids (as normalized MS intensity) from control, non-acclimated, and acclimated plants at 74 h. (**b**–**d**) Levels of the three most common diacyl Arabidopsides at 74 h in control, non-acclimated and acclimated rosettes. MS intensities are normalized to internal standards and dry plant mass. In (**a**), the size of the pies reflects the size of each oxidized lipid pool, and the values in the non-acclimated and acclimated pies indicate the fold increases of each sub-class, compared to the levels in control plants. In (**b**–**d**), errors bars indicate standard deviation. *n* = 18.

Analyzed oxidized polar lipids include DGDGs, MGDGs, PGs, PCs, and PEs. As a group, oxidized polar lipids were higher in non-acclimated and acclimated plants at 74 h than in control plants, and 74 h was the time point at which the levels of oxidized lipids in plants exposed to freezing were the highest. However, compared to changes in other groups of lipids, the changes were modest, and somewhat noisy, with non-acclimated rosettes having 1.5 times higher levels than control plants and acclimated plants having 2.0 times higher levels than control plants (Figure 9a and Figure S6, Table S5). The oxidized diacyl lipids in the plastidic classes (DGDG, MGDG, and PG), which contain OPDA and dnOPDA, tended to increase more than oxidized PC and PE, which do not contain OPDA and dnOPDA. The 1.5- to 2-fold increases for the total are perhaps a bit misleading because they include some lipid species with relatively high MS intensities that did not differ much from control, while a number of oxidized polar glycerolipids with lower apparent levels showed higher fold changes. For example, Arabidopsides A, B, and D were 4- to 11-fold higher in non-acclimated plants, compared to control levels, and 6- to 12-fold higher in acclimated plants, compared to control levels, at 74 h (Figure 9b–d). Previous work demonstrated that these three lipid molecular species were modestly increased during cold treatment [31].

Sixty-four of the one hundred and fifteen lipids in the oxidized polar diacylglycerolipid group had negative correlations between their levels immediately after freezing (74 h) and ion leakage at the final time point (98 h) (Table S5). None had positive correlations. Many of the compounds in the oxidized polar diacylglycerolipid group, including many that have negative correlations with ion leakage at 98 h, are present in very low amounts, with some samples near or perhaps below the limit of detection of the MS method (Table S6). The criterion for the presentation of lipids was a coefficient of variation in the quality control samples of <0.20 (indicative of repeatable data); no criterion for the limit of detection was applied. Low-abundance lipids are identified with asterisks in Figure S6 and elsewhere. Although the data on many oxidized polar diacylglycerolipids appear to be quite noisy (Figure S6), the identification of 64 significant negative correlations and 0 significant positive correlations suggests that formation of at least most of the identified compounds is associated with a good outcome.

The negatively correlated oxidized diacyl lipids contain a variety of oxidized fatty acids shown in Table 1. There is evidence that the jasmonate pathway, which produces OPDA and dnOPDA, is associated with a beneficial outcome in response to freezing. Hu et al. [32] found that jasmonate is an upstream activator of the ICE-CBF/DREB1 pathway that regulates the transcription of genes leading to freezing tolerance. They found that mutations in the jasmonate pathway made non-acclimated and cold-acclimated plants more sensitive to freezing. Indeed, overexpression of the jasmonate biosynthetic enzyme 12-oxophytodienoic acid reductase 3 (OPR3) in wheat conferred short-term freezing resistance [33]. There is less data on the role of fatty acid oxidization via other pathways with regard to freezing tolerance. Using diaminobenzidine staining, Chen et al. [34] showed that freezing increased the production of reactive oxidized species under conditions very similar to those used in the current work. They also demonstrated that mutants with increased freezing tolerance had lower reactive oxygen species production. It is reasonable to imply that reactive oxygen species might interact with lipids resulting in the production of oxidized lipids, and that this might reduce freezing tolerance. However, the current data do not provide support for that potential mechanism of freezing tolerance, since levels of nine out of ten of the oxidized polar glycerolipid species are consistent with identification as non-enzymatically produced phytoprostanes, (i.e., those containing 18:3;O3, Table 1), were negatively correlated with ion leakage 24 h after freezing (Table S5). Another possible explanation for the negative correlation of 74-h levels of oxidized polar lipids with 98-h ion leakage considers the fact that acclimated plants have more intact structural lipids, particularly polyunsaturated ones, after freezing (Figure 5 and Figure S2). The larger pool of structural lipids in acclimated plants may be able to serve as a sink for more reactive oxygen species than the structural lipids of non-acclimated plants. The improved outcome could be due to a reduction in reactive oxygen species interaction with other, perhaps non-lipid, targets. Alternatively, the improved outcome could be causally associated with the higher levels of polar structural lipids, which coincidentally generate higher levels of oxidized polar lipids.

### 2.10. Monoacyl Polar Lipids

**Figure 10:**
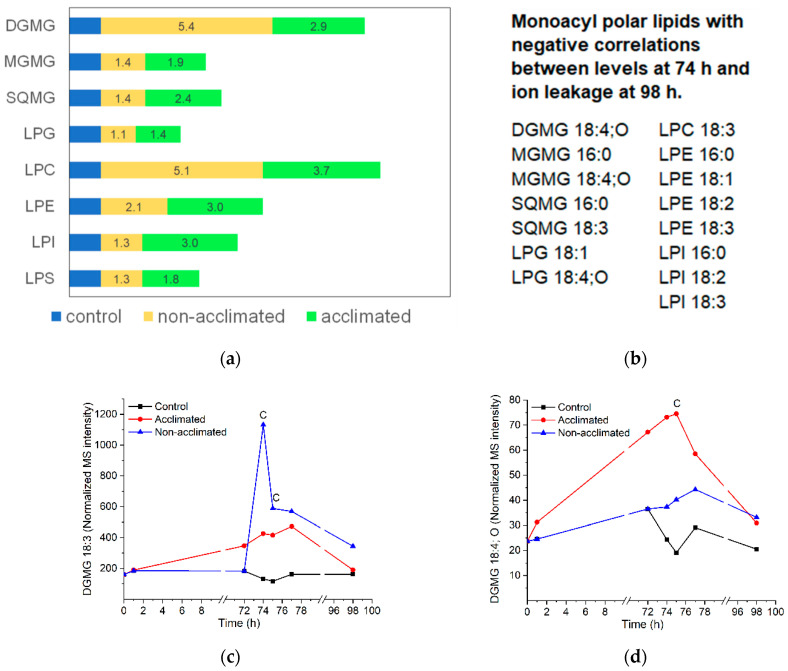
Monoacyl polar lipid levels and correlations in response to freezing. (**a**) Levels of each class of monoacyl polar lipid in non-acclimated and acclimated plants, normalized to levels in control plants, which were set at 1. (**b**) Monoacyl polar lipids with negative correlations of levels at 74 h with ion leakage at 98 h. (**c**) Time course of DGMG 18:3 in control, non-acclimated, and acclimated plant rosettes. (**d**) Time course of DGMG 18:4;O in control, non-acclimated, and acclimated plant rosettes. Panels (**c**,**d**) show the same data as Figure S7, pp. 7B and 7C. In panels (**c**,**d**), “C” indicates that the lipid level in non-acclimated or acclimated plants is significantly different than the control level (Table S2). MS intensities are normalized to internal standards and dry plant mass. *n* = 18. Abbreviations: sulfoquinovosylmonoacylglycerol (SQMG), lysoPG (LPG), lysoPC (LPC), lysoPE (LPE), and lysoPI (LPI).

Similar to the oxidized polar diacylglycerolipids, the levels of monoacyl polar lipids are generally low, and the data are noisy. All classes of monoacyl polar lipids increase when subjected to freezing (Figure 10a), and most peak at 74 or 75 h (Table S5 and Figure S7). In some classes, the total levels of monoacyl polar lipids are higher in non-acclimated than in acclimated plants, and in other classes, the reverse is true; within classes, there is considerable variation in pattern for different molecular species (Figure S7). Perhaps a bit surprisingly, the levels at 74 h of about half (15/31) of the molecular species are negatively correlated with ion leakage at the final time point, while none are positively correlated. Negatively correlated molecular species include both plastidic (DGMG, MGMG, SQMG, and LPG) and non-plastidic (LPC, LPE, and LPI) species (Figure 10b).

During freezing, monoacyl polar lipids are likely to be formed, at least in part, by acyl hydrolase activity, although the identity of the enzymes that produce the majority of molecular species in this group is not clear. There are a number of types of acyl hydrolases in Arabidopsis, including the patatin-like enzymes, that often have specificity for both phospholipids and galactolipids [41]. In particular, the expression of pPLAIIα is increased in cold and can act on a variety of substrates [42,43], but no direct evidence has demonstrated its role in cold or freezing. Three proteins that act together and as a complex in plant defense, SAG101, EDS1, and PAD4, have lipase (acyl hydrolase) or lipase-like motifs, suggesting they may be triacylglycerol lipases [44,45]. Only SAG101 has demonstrated acyl hydrolase activity, and the demonstration indicated the production of free fatty acid from TG [46]. However, it is important to note that acyl hydrolases often act on a variety of substrates. Knock-out *sag101*, *eds1*, and *pad4* mutants have been identified as having increased freezing tolerance and lower LPC levels under freezing stress [46]. If LPC is the product of the putative lipases, the genetic data are contrary to the prediction from the correlation data indicating that higher levels of monoacyl polar lipids immediately after freezing are associated with beneficial function. On the other hand, PA levels were also substantially reduced in the *sag101*, *eds1*, and *pad4* mutant plants, and overall lower levels of PA immediately after freezing are associated with a better outcome, so it is conceivable that the PA alteration could account for the improved freezing tolerance of the mutants.

While many monoacyl polar molecular species are formed by uncertain pathways, DGMG can be formed by AGAP1 action on its preferred substrate, DGDG, as an acyl chain is transferred to the head group of another plastidic lipid (often MGDG) [7,47]. Oxidation of fatty acids on intact galactolipids generally results in the oxidation of both esterified fatty acids [29]. Because of this, it is likely that DGMG with an oxidized fatty acid was derived from the transfer of an oxidized fatty acid to an acylated lipid’s head group, and DGMG with a non-oxidized fatty acid is likely to derive from the transfer of a non-oxidized fatty acid. Figure 10c shows the profiles for the formation of DGMG 18:3, which, by this logic, is produced in the formation of non-oxidized head group-acylated plastidic lipids, and DGMG 18:4;O (Figure 10d), which is produced in the formation of oxidized head group-acylated plastidic lipids. Consistent with the positive correlation between non-oxidized head group-acylated lipid production at 74 h and ion leakage 24 h after the freezing treatment, DGMG 18:3 production tends to be higher in non-acclimated plants than in acclimated plants, although its 74-h levels did not exhibit a significant correlation with ion leakage at 98 h. Consistent with a negative correlation between oxidized head group-acylated lipid levels at 74 h and ion leakage 24 h after the freezing treatment, DGMG 18:4;O production tends to be higher in the acclimated treatment than in non-acclimated plants. Indeed, DGMG 18:4;O levels at 74 h are also negatively correlated with ion leakage at 98 h (Figure 10b).

### 2.11. Neutral Glycerolipids

**Figure 11:**
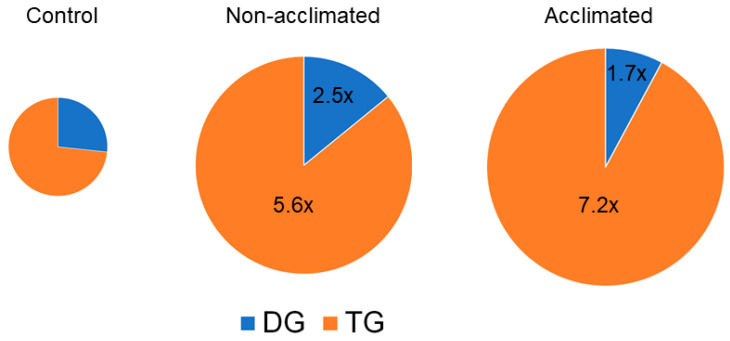
Neutral glycerolipid levels in response to freezing. Pie graph of levels of neutral glycerolipids (as normalized MS intensity) from control, non-acclimated, and acclimated plants at 77 h. The size of the pies reflects the size of each oxidized lipid pool, and the values in the non-acclimated and acclimated pies indicate the fold increases of each sub-class, compared to the levels in control plants. *n* = 18.

DG and TG both tended to increase in response to cold and freezing (Figure 11 and Figure S8). The level of DG in both non-acclimated and acclimated plants at the 74-, 75-, and 77-h time points was approximately 2- to 3-fold higher than control. In contrast, the level of the measured TGs in non-acclimated plants peaked at 77 h, when the level in non-acclimated rosettes was 5.6-fold higher than the level in control plants, and the level in acclimated plants was 7.2 times higher. 

The repertoire of TG species measured in the current work was limited to TG 18:3_34:2, TG 18:3_36:5, TG 18:2_36:5, and TG 18:3_36:6. The levels of all of these TG species at 74 h were negatively correlated with ion leakage at 98 h, while none of the six DG species were correlated. Degenkolbe et al. [17] measured a larger number of TG molecular species during a 14-day cold acclimation and identified 14 TG molecular species that were positively correlated with freezing tolerance, leading to the hypothesis that TG formation is beneficial to plants undergoing freezing stress. One species they identified as correlated with freezing tolerance is TG 52:5, which is consistent with our identification of TG 18:3_34:2. The association of TG with freezing tolerance was verified by increasing TG levels in a wild relative of Arabidopsis by over-expression of DGAT1 [12], which catalyzes the transfer of an acyl chain from acyl-CoA to DG to form TG, and by a similar experiment in Arabidopsis [11]. Arisz and coworkers showed that, among naturally-occurring variants, the freezing-tolerant plants had higher DGAT1 expression compared to freezing-sensitive plants. Tan et al. [11] also demonstrated lower freezing tolerance in an Arabidopsis *dgat1* knockout mutant. In addition to DGAT1, phospholipid:diacylglycerol acyltransferase 1 (PDAT1) should also be considered as a potential player in TG accumulation during freezing. PDAT1 can play a major role in the accumulation of TG in leaves and has been demonstrated to play a role in heat tolerance [25,48]. Recent work indicates that PDAT1-overexpressing lines grow better and *pdat1* knockouts grow worse than wild-type plants and that the differences between the mutants and wild-type plants were enhanced when the plants were grown at 4 °C [49].

### 2.12. Sterol Derivatives

Sterol derivatives include sterol hexosides (three molecular species; accounting for ~34% of the total normalized MS signal of sterol derivatives under control conditions at 74 h), acyl sterol hexosides (twelve molecular species; accounting for ~65% of sterol derivative MS signal), and sterol esters (nine molecular species; accounting for ~0.1% of sterol derivative MS signal) (Table S5). OPDA-containing (i.e., “oxidized”) versions of acyl sterol hexosides and sterol esters were measured, but they represent ≤ 2% of the acyl sterol hexoside or sterol ester classes under all conditions (Table S5). 

**Figure 12:**
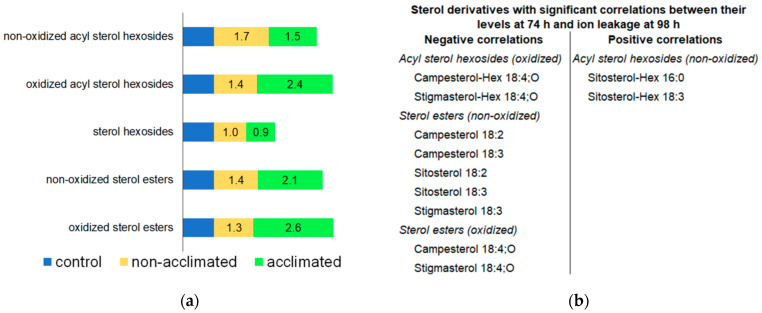
Sterol derivative levels in response to freezing. (**a**). Levels of each class of sterol derivative at 74 h in non-acclimated and acclimated plants, normalized to levels in control plants, which were set at 1. (**b**). Sterol derivatives with negative or positive correlations of levels at 74 h with ion leakage at 98 h. *n* = 18.

At the 74-h time point, acyl sterol hexoside and sterol ester levels were modestly increased (1.5–2.6-fold) in plants that underwent freezing, whereas sterol hexosides were virtually unchanged (Figure 12a and Figure S9). Tarazona et al. [8] also observed increased levels of sterol esters in Arabidopsis when plants were subjected to cold (6 °C) with or without −2 °C freezing. Levels of oxidized acyl sterol hexosides and some normal-chain- and OPDA-containing sterol esters immediately after freezing (74 h) have significant negative correlations with ion leakage 24 h later (at 98 h) (Figure 12b). On the other hand, two of the most prominent acyl sterol hexosides, sitosterol-Hex 16:0 and sitosterol-Hex 18:3, have positive correlations of levels at 74 h with ion leakage at 98 h. Uemura and Steponkus’ data on lipid levels and freezing tolerance in summer oat and winter rye indicated a role of acyl sterol hexosides in creating a poorer outcome in freezing due to increasing the propensity for hexagonal phase formation [50]. Acyl sterol hexosides in the plasma membrane are higher in the less freeze-resistant summer oat compared to winter rye, and they also decrease in both species during cold acclimation. These data are consistent with the positive correlation that we observed for sitosterol-Hex 16:0 and sitosterol-Hex 18:3 with ion leakage and the hypothesis that these acyl sterol hexosides are not beneficial. On the other hand, Mishra et al. [51], who examined the role of Arabidopsis sterol glycosyltransferase, UDP GLYCOSYLTRANSFERASE 80B1 (UGT80B1), one of the enzymes producing sterol hexosides in freezing tolerance, found that the presence of UGT80B1 in wild-type plants increased their ability to tolerate freezing, compared to knockouts lacking the enzyme. Mishra and coworkers’ results imply that sterol hexosides and their products would have negative correlations with ion leakage at the final timepoint. Indeed, we observed negative correlations for some sterol derivative species, although we did not find any significant correlation for the immediate product of UGT80B1, sterol hexosides. Because the plants tested by Mishra et al. [51] were 14-day-old plants, which sustained significant damage at −1 °C, their results might not be directly comparable to the plants in the current study, which were 30 days old and subjected to −8 °C.

Little is known about acyl sterol hexosides containing OPDA, which are present at low levels. We previously reported that the level of Stigmasterol-Hex 18:4-O increased in response to heat stress [15]. In freezing, levels of two OPDA-containing acyl sterol hexosides immediately after freezing are negatively associated with ion leakage at 98 h (Figure 12b). Thus, we hypothesize that they are markers of freezing tolerance, similar to head-group acylated MGDGs in which OPDA is also linked to a sugar (Section 2.8).

### 2.13. Lipids of Plants with Mutations in Lipid-Related Genes

**Figure 13:**
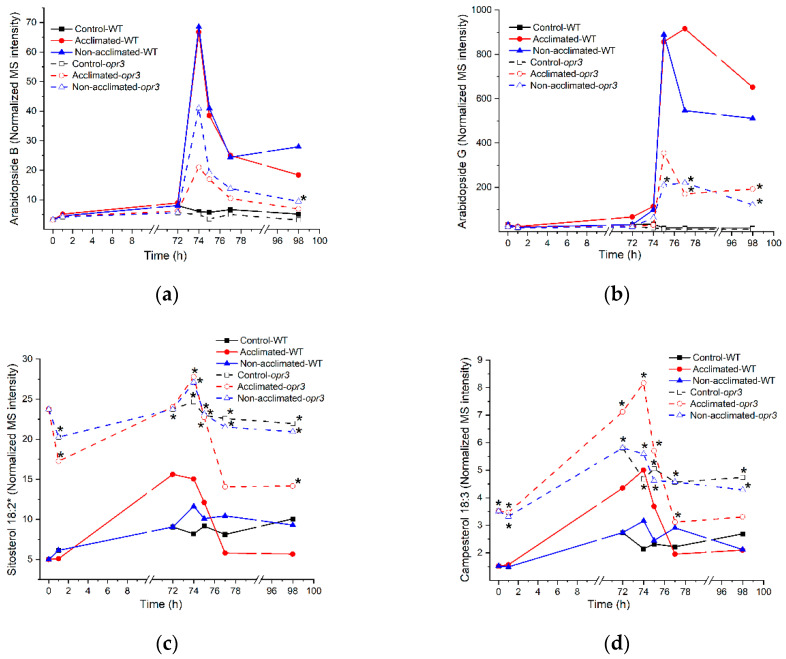
Time courses of selected lipids that differ in levels between rosettes of *opr3* mutants and wild-type plants. (**a**) MGDG 18:4;O_18:4;O (Arabidopside B); (**b**) MGDG-O(FA 18:4;O) 36:8;O2 (Arabidopside G); (**c**) Sitosterol 18:2; (**d**) Campesterol 18:3. The same graphs can be found in Figure S10. MS intensities are normalized to internal standards and dry plant mass. Asterisks on the graphs indicate that the lipid levels in *opr3* plants are significantly different than those in wild-type plants (Table S9). *n* = 18 for wild type and *n* = 9 for the *opr3* mutant.

Lipid levels in plants with mutations in genes encoding patatin-like phospholipases, lipoxygenases, and OXOPHYTODIENOIC ACID REDUCTASE 3 (OPR3) were compared with those of wild-type plants at each time point and treatment, (i.e., on each tray). The list of mutants can be found in Table S7, with the nomenclature for the patatin-like lipases following that in Chen et al. [41]. The ion leakage and lipid level data are in Table S8. Using the conservative Bonferroni correction, only 177 significant lipid differences were identified [52,53] (Table S9). Ion leakage did not differ significantly among the genotypes. Of the observed differences, 139 were between lipids in wild-type plants and those in *opr3*, while the other 38 differences from wild-type lipid levels were distributed among lipid levels of several other mutants (Table S9). Time courses of levels of selected lipids that have significant differences between wild-type and *opr3* rosettes are shown for these genotypes in Figure 13 and Figure S10. Two major groups of lipids are different between wild type and *opr3* (Figure 13a,b). Lipids containing OPDA are significantly lower in *opr3* than in the wild type, but the difference is observed primarily after freezing treatment. Because oxophytodienoic acid reductase 3 catalyzes the final step in the formation of OPDA, lower levels of OPDA-containing lipids in the mutant were expected. The magnitude of the reduction of Arabidopsides A and B to about half of their wild-type levels is consistent with data from plants with the same mutation in OPR3 tested during the wounding response [54], suggesting that either the point mutation in this *opr3* mutant results in an enzyme with partial enzymatic activity compared to wild-type OPR3 protein or that the OPR1 and OPR2 proteins may also produce Arabidopsides. Levels of sterol esters are higher in *opr3* than in wild-type plants, and this is true regardless of control, cold, freezing, or recovery treatments (Figure 13c,d). The basis for the higher levels of sterol esters in *opr3* is not clear, but it is conceivable that a product of OPR3 could serve as a negative regulator of sterol ester biosynthesis.

### 2.14. Differences in Sizes of Mutant Plants Compared to Wild-Type Plants

Counting the number of leaves at 20 and 27 days after planting indicated that the *pplaIIα* mutant and the *pplaII* × *pplaIIIβ* double mutant had more leaves than wild-type plants of the same age (Table 2, Tables S10 and S11). On the other hand, the plants with mutations in cytosolic lipoxygenases, *lox1-1*, *lox5-1*, and a double mutant of these genotypes, as well as *opr3*, had fewer leaves than wild-type plants of the same age. The *lox1-1* plants also had a smaller dry mass per plant compared to wild-type plants. The differences were small, but detectable because a large number of plants (204 of wild type and 102 of each other genotype) were observed. 

### 2.15. Summary of Hypotheses Generated from Correlation Analsysis

Correlation of the levels of Arabidopsis rosette lipids produced during a 2-h exposure of the plants to freezing (with or without cold acclimation) with the outcome of the freezing treatment, as measured by ion leakage 24 h after the freezing treatment, has allowed the development of hypotheses about the roles of specific lipids in relation to freezing tolerance (Table 3). In particular, higher levels of MGDGs that are not fully unsaturated, most PAs, non-oxidized, (i.e., normal-chain) acylated MGDGs, and non-oxidized sterol hexosides are associated with a poor outcome, whereas many structural lipids, polygalactosylated galactolipids, PA 34:6, a number of oxidized lipid species, monoacyl polar lipids, triacylglycerols, and sterol esters are associated with a good outcome after exposure to freezing temperatures. 

Do lipids with negative correlations with final ion leakage play causative roles in freezing tolerance? Do lipids with positive correlations with ion leakage cause freezing-induced damage? In some cases, the relationships between the lipids and their effects on freezing tolerance have been convincingly demonstrated by genetic analysis, and in those cases, observed associations are consistent with the results of genetic analyses (Table 3). For other lipids, the generated hypotheses provide starting points for additional testing of the roles of lipids and pathways in freezing damage and tolerance.

## 3. Materials and Methods

### 3.1. Experimental Design

Twenty-three Arabidopsis lines are shown in Table S7 and described in the Supplemental Methods section. Each line was given a letter label (A to X) to indicate the genotype (Table S7), and details about the source and confirmation of the identity of the lines can be found in the Supplemental Methods. Wild-type Columbia-0 (Col-0) accession was duplicated (A and M). The lines were grown in triplicate in 72-well plug trays (Figures S11 and S12). Each tray was treated with one of the conditions indicated in Figure 1. All 17 treatments (17 trays) were repeated three times (referred to as blocks 1, 2, and 3).

Each of the plug tray wells was labeled from 1–72 (Figures S11 and S12). The positions of each line were the same in each tray within a block, but different in each block (Figure S11). The positions of each line in a tray were randomized, but the exchange of positions was performed according to rules that assured that 1–2 plants of each of the 24 lines were in an outside well. Sample naming is: F (for freezing) block tray position. Seed sowing of the 17 trays of each block was done on 5 consecutive days (day 1: trays 1, 2, 4, 7, 10, and 12; day 2: trays 3, 5, 8, and 11; day 3: trays 6, 9, 14, and 16; day 4: trays 13 and 17; day 5: tray 15). All handling and treatments of each tray (watering, thinning, fertilizing, photographing, treating, and harvesting) were done according to a staggered schedule, so that time-consuming steps, such as thinning or harvesting, could be completed, and so that no more than two trays would require freezing treatment on the same day.

### 3.2. Plant Growth

Pro-Mix “PGX” soil (Hummert International, Earth City, MO, USA) was mixed with tap water until fully wetted, autoclaved for 1 h, and cooled to room temperature before potting. A 72-well TLC Square Plug tray (International Greenhouse Company, Danville, IL, USA) was placed inside a tray with holes, and both were placed inside another tray without holes (Hummert International). The trays were filled with 2.5 L of fertilizer solution (0.01% Peters 20: 20: 20 (Hummert International) in tap water) before sowing.

Seeds were planted in randomized positions as described above and shown in Figure S11. Each plant was labeled with the tray and a well number. Four seeds were placed near the center of each well. After sowing, the tray was drained, covered with a propagation dome (Hummert International), and kept at 4 °C for 2 d before transfer to growth conditions (21 °C, 60% humidity, 80–100 µmol m^−2^ s^−1^ photon flux, 14 h light/10 h dark). On day 9, counting from the time the tray was transferred, the propagation dome was removed. On day 11, plants were thinned so that only the largest plant remained. Trays were watered by sub-irrigation once a week. On day 20, trays were sub-irrigated with the 0.01% fertilizer solution. Plants began low-temperature treatments on day 28.

### 3.3. Cold Acclimation and Freezing Treatment

Cold acclimation was performed in a 4 °C room equipped with a light cart with the same day/night schedule as the lights in the growth chambers. Freezing treatment was performed in a programmable freezing chamber (Espec Corporation, Hudsonville, MI, USA). For freezing treatment, each tray of plants in soil was partly submerged in an ice slurry (made by adding tap water to approximately 1.5 kg of ice chips to a total volume of 4 L) to avoid supercooling during freezing treatment at −8 °C for 2 h. The soil was completely in contact with the ice slurry through the irrigation holes at the bottom of the growing tray. The temperature was dropped to −8 °C without gradual decreasing; at the end of the freezing treatment, plants were transferred to their growth condition (21 °C, 60% humidity). 

### 3.4. Sampling, Lipid Extraction, and Profiling by ESI Triple Quadrupole Mass Spectrometry

Two types of samples were collected from each plant (72 plants per tray): (1) leaves 5 and 6 in a 50-mL tube containing 25 mL of distilled water (Dillons Supermarket, Manhattan, KS, USA) for ion leakage measurements (described below) and (2) the rest of the rosette in a 20-mL vial containing 4 mL of isopropanol with 0.01% butylated hydroxytoluene (BHT) at 75 °C for lipid extraction. Leaf number is the order of leaf appearance [55]. Lipid extraction and sample preparation were very similar to that described previously [14,15]. Additional details of how the sampling was accomplished are in the Supplemental Methods section. 

To begin lipid extraction, each set of 72 leaf samples in isopropanol from one tray stored at −80 °C was allowed to warm to room temperature. To each vial, 12 mL of the extraction solvent (chloroform: methanol: 300 mM ammonium acetate in water, 30: 41.5: 3.5, *v*/*v*/*v*) was added. The vials were shaken on an orbital shaker at 100 rpm for 24 h. After being shaken, the extracted rosette from each vial was removed and put into an empty vial with the same label. The original vials with solvent were stored at −20 °C. The extracted rosettes in non-capped vials were dried first in a fume hood for 1–2 h and then in an oven at 105 °C overnight. The dried rosettes were allowed to cool to room temperature and weighed using a Mettler-Toledo AX balance (Mettler-Toledo, Greifensee, Switzerland). To eliminate electrostatic forces resulting from drying of the rosettes, the rosettes were passed through an anti-static U ionizer (Haug, Germany). 

A mixture of internal standards in chloroform was included in all mass spectrometry samples for analysis (including the sample vials, the internal standard-only vials, and the quality control (QC) vials). The composition of the internal standard mixture (20 µL) added per 0.04 mg dry mass of leaf tissue is listed in Table S3.

A quality control (QC) stock was prepared by pooling 1 mL from samples 1–10 of all the trays of blocks 1 and 2 (a total of 34 trays). The total volume of the QC stock was 340 mL and represented 0.688 mg leaf dry mass ml^−1^. The QC stock was divided into 34 aliquots of 10 mL each and the aliquots were stored at −20 °C. To make mass spectrometry QC vials, a QC stock aliquot was mixed with 3.4 mL of the internal standard mix and 224.6 mL of mass spectrometry solvent (isopropanol: chloroform: methanol: 300 mM ammonium acetate in water, 25: 30: 41.5: 3.5, *v*/*v*/*v*/*v*). After shaking, 1.4 mL of the mixture was dispensed into each of 156 amber 2-mL vials labeled “QC1” to “QC39” (enough for analysis of 4 trays). The prepared QC mass spectrometry vials were stored at −80 °C and were brought to room temperature 1 h before analysis.

To prepare the sample mass spectrometry vials, the 20-mL vials containing the extracted total lipids were brought to room temperature from −20 °C~2 h prior to handling, one tray (72 vials) at a time. To each of the 72 2-mL amber vials (labeled “1” to “72”), 20 µL of the internal standard mixture was added first. Then a volume that contained 0.04 mg leaf dry mass from a 20-mL vial was added to the corresponding 2-mL amber vial. A volume of mass spectrometry solvent (isopropanol: chloroform: methanol: 300 mM ammonium acetate in water, 25: 30: 41.5: 3.5, *v*/*v*/*v*/*v*) was added to bring the total volume to 1.4 mL, and the amber vial was capped. 

In each tray, 6 internal standard-only vials were included (labeled “IS1” to “IS6”); each contained 20 µL of the internal standard mix and 1.38 mL (total of 1.4 mL) of the mass spectrometry solvent. For mass spectrometry analysis, the 72 sample vials from each tray, together with 6 “IS” vials and 39 “QC” vials, were arranged in 3 VT-54 racks. Table S12 lists the positions of mass spectrometry vials in the first VT-54 rack, with QC vials 1–13, IS vials 1–2, and sample vials 1–24. The second and third VT-54 racks have the same arrangement, with QC 14-26, IS 3-4, and sample 25–48 on the second VT-54 rack and QC 27-39, IS 5-6, and sample 49–72 on the third VT-54 rack.

### 3.5. Mass Spectrometry Data Collection and Processing

Data collected by direct-infusion multiple reaction monitoring on a Xevo TQS mass spectrometer (Waters Corporation) were exported to Excel, isotopically deconvoluted, and compared to internal standards (indicated in Table S3). Data from background (“internal standard-only”) samples were subtracted, and the data were normalized to QC samples to reduce analytical variation over time, as described by Vu et al. [14]. After calculation of the lipid levels in each sample, the values were divided by the dry mass of the sample analyzed (0.04 mg). The lipid values are normalized intensity per mg leaf dry mass, where a value of 1 is the intensity of 1 pmol of internal standard. Because the internal standards were not uniformly well-matched to the lipids analyzed (some differ in class; many differ substantially in *m*/*z*), the absolute values of the analytes provide only a rough guide to absolute amount of each lipid.

### 3.6. Phenotype Analysis

In parallel with lipid analysis, ion leakage measurements were performed to detect membrane damage caused by the treatments. When each plant was harvested, leaves number 5 and 6 (as determined in [55]) were dropped into a 50-mL glass tube containing 25 mL of distilled water (purchased from Dillons Supermarket, Manhattan, KS, USA). The tubes were tightly capped and shaken at 150 rpm for 2 h. Conductivity was measured using an electrical conductivity meter, CON 510 (Oakton Instruments, Vernon Hills, IL, USA). After the first measurement, the tubes were re-capped and incubated in a water bath at 80–90 °C for 2 h and were allowed to cool to room temperature so the conductivity corresponding to the total ion content could be measured. The conductivity of distilled water alone was also measured. Ion leakage (in percent) was calculated as (the first conductivity measurement minus that of water alone) × 100 divided by (the total conductivity value minus that of water alone) for each plant.

The counting of leaf numbers is described in the Supplemental Methods section.

### 3.7. Statistical Analysis

#### 3.7.1. Comparison of Lipid Levels and Ion Leakage of Wild-Type Plants at Each Time Point as a Function of Treatment

Because the control, non-acclimated, and acclimated pathways share some of the same observations, only pairwise comparisons were performed. To compare control and non-acclimated plants, the data on the 244 lipids and ion leakage, as indicated in Table S13, from trays 6, 9, 12, and 15 for control plants and from trays 7, 10, 13, and 16 for non-acclimated plants, were used. To compare control and acclimated plants, the data from trays 2, 4, 6, 9, 12, and 15 for control plants and from 3, 5, 8, 11, 14, and 17 for acclimated plants were used. To compare non-acclimated and acclimated plants, trays 2, 4, 7, 10, 13, and 16 and trays 3, 5, 8, 11, 14, and 17, respectively, were compared. For each of the pairwise comparisons, modeling was conducted for each lipid separately. For each lipid, an ANOVA model was fitted to the data with the following effects included in the model: block effect (each replicate experiment is a block), time effect, and path effect (control, non-acclimated, and acclimated). The p-values for all effects from all three pairwise comparisons were recorded. The adjusted p-values, based on the false discovery rate [56], were calculated, and *p* < 0.05 was considered significant (Table S2).

#### 3.7.2. Correlation Analysis of Lipid Levels and Ion Leakage in Wild-Type Plants

Using the MetaboAnalyst website, levels of each lipid in wild-type plants on non-acclimated and acclimated trays at each time point from 72 h to 98 h were correlated with ion leakage on trays of non-acclimated and acclimated plants at 98 h (24 h after the freezing treatment), using Spearman’s correlation analysis [57,58,59,60]. To calculate the correlation coefficients and their p values between ion leakage at 98 h and lipid levels at other time points, the ion leakage data were aligned with lipid data from wild-type plants in the same tray position, subjected to the same treatment (non-acclimated or acclimated), and in the same block. In the case where data from a plant in a particular position on a tray were missing, the data from that position on the corresponding tray were also eliminated from the correlation analysis and from determined averages. Uncorrected p values of less than 0.05 were considered significant for the purpose of assessing the number of significant values in each lipid group.

#### 3.7.3. Comparison of Lipid Levels and Ion Leakage at Each Time Point as a Function of Genotype (Wild Type vs. Mutant)

Using the data for one tray at a time, significant genotype effects were determined. All measured lipids were included in the analysis. A split-plot design model was fitted with block and genotype as fixed effects, and block as a random effect. (The fixed effect of block accounts for any systematic bias due to block, whereas the random effect of block was included to account for possible positive correlation due to sharing the same block.) To see which genotypes are different from wild type (A and M groups) in lipid levels, a Dunnett multiple comparison test with control was conducted between each genotype’s lipids and wild-type lipids for each lipid on each tray, where each tray represents the same time point and treatment. The Dunnett *p*-values were adjusted using the Bonferroni correction. Lipid levels were considered significantly different between a genotype and wild type if their Bonferroni-corrected *p* values were < 0.05 (Table S9). 

## 4. Conclusions

The ability of *Arabidopsis thaliana* to recover after exposure to −8 °C was enhanced by previous cold acclimation at 4 °C, as demonstrated by lower ion leakage from leaves of acclimated plants than from leaves of non-acclimated plants at 24 h after freezing. Mass spectrometry-based analysis of leaf lipids demonstrated that levels of structural lipids decreased, while levels of many other lipids increased during exposure to freezing. Correlation coefficients between levels of lipids formed during freezing exposure and ion leakage after 24 h of recovery in acclimated and non-acclimated plants were determined. Many structural lipids, polygalactosylated galactolipids, PA containing 16:3, a number of oxidized lipid species, monoacyl polar lipids, TGs, and sterol esters were associated with low ion leakage at 24 h post-freezing. Levels of less-than-fully-unsaturated MGDG, most PAs, non-oxidized acylated MGDGs, and non-oxidized acyl sterol hexosides were associated with high ion leakage. The correlations were consistent with expectations based on previous genetic manipulation of levels of PA, polygalactosylated galactolipids, and unsaturation in MGDG, suggesting that it may be possible to infer information about lipid function by assessing the correlation of lipid levels with a quantitative measure of freezing outcome. The approach thus provided new, testable hypotheses about the functions of the lipids analyzed.

## Figures and Tables

**Table 1:** Oxidized fatty acids in oxidized diacylglycerolipids with negative correlations of 74-h levels with 98-h ion leakage.

Designation	Putative Identification	Reference(s) to Fatty Acid in Plants and/or in Complex Lipids
7:1;O	7-oxoheptanoic acid	[35]
7:1;O2	pimelic acid	[35]
9:1;O	9-oxononanoic acid	[35]
9:1;O2	azelaic acid	[35]
16:4;O	dinorOPDA	[28,29,36,37]
16:3;O	hydroxy 16:3	
18:5;O2	unknown	
18:4;O	OPDA, keto 18:3	[28,29,30,36,37,38,39]
18:4,O3	unknown	
18:3;O	hydroxy 18:3, keto fatty acid	[30]
18:3;O2	ketol fatty acid, hydroperoxy 18:3, dihydroxy 18:3	[29,30]
18:3;O3	phytoprostane	[40]
18:2;O	hydroxy 18:2	

**Table 2:** Genotypes with significant differences compared to wild type in rosette leaf number or dry mass. “H” indicates a significantly higher value than wild type, and “L” indicates a significantly lower value than wild type. ANOVA was performed with Tukey’s HSD post hoc test. Errors are standard deviations. Full data set is in Tables S10 and S11.

	Leaf Number at 20 Days	Leaf Number at 27 Days	Rosette Dry Mass
wild type	5.16 ± 0.82	9.25 ± 1.21	9.04 ± 5.69
*pplaIIα*	5.47 ± 0.78	10.10 ± 0.98 ^H^	9.40 ± 5.73
*pplaIIα* × *pplaIIIβ*	5.71 ± 0.67 ^H^	10.08 ± 1.00 ^H^	10.38 ± 5.78
*lox1-1*	4.33 ± 0.89 ^L^	7.71 ± 1.25 ^L^	6.23 ± 3.72 ^L^
*lox5-1*	4.50 ± 0.87 ^L^	8.35 ± 1.68 ^L^	7.79 ± 5.53
*lox1-1* × *lox5-1*	4.47 ± 0.99 ^L^	8.37 ± 1.48 ^L^	7.96 ± 8.44
*opr3-2*	4.54 ± 0.77 ^L^	8.37 ± 1.00 ^L^	8.93 ± 6.29

**Table 3:** Summary of hypothesized roles of lipids in freezing tolerance based on correlation analysis of lipid levels immediately after freezing exposure with leaf ion leakage 24 h later. The table is divided into hypotheses based on negative (Part 1) and positive (Part 2) correlations. The last column indicates the authors’ view of the most relevant evidence for the hypotheses.

Part 1. Negative Correlations of Level at 74 h with Final Ion Leakage, i.e., Associated with Good Outcome.			
Hypothesis: *Increasing the Amounts of “Lipid or Lipid group” Produced During Freezing Will Increase Freezing Tolerance*.			
Lipid or Lipid Group	Possible Role in Freezing Sensitivity or Tolerance	Supporting Evidence from Previous Work/Comments	Status
Many structural polar lipids, especially those with polyunsaturated fatty acyl chains (Figure 5)	Form and stabilize membranes	Extensive biophysical evidence indicates most lipids in this group participate in bilayer structures. This group of lipids is the only group at 98 h negatively correlated with ion leakage at 98 h (Figure 4b), consistent with these lipids being present when plants are recovered.	
Structural lipids with very long-chain fatty acyl chains (Figure 5)	May reduce propensity for non-bilayer phases caused by dehydration during ice formation	Genetic manipulation of very long-chain fatty acid content shows higher content of very long-chain fatty acids is associated with chilling tolerance [20].	Some evidence in chilling
Polygalactosylated diacylglycerols (Figure 6)	Stabilize the chloroplast envelope	The *sfr2* mutant has a poor outcome upon freezing compared to wild type [5].	Good evidence
PA 34:6 (i.e., PA 18:3_16:3) (Figure 7)	Is a byproduct of polygalactosylated diacylglycerol synthesis	Synthesis of PA 34:6 after wounding is highly correlated with polygalactosylated diacylglycerol synthesis. It may be a side product rather than causative [14].	No direct evidence
Oxidized head group-acylated plastidic lipids (Figure 8), oxidized polar diacyl lipids (Figure 9), and OPDA-containing sterol derivatives (Figure 12)	Some may serve as a sink for reactive oxygen species; some might serve as signaling molecules.	OPDA-containing species are formed by the same pathway as jasmonates, which activate the ICE-CBF/DREB1 pathway leading to freezing tolerance [32].	No direct evidence about the role of the measured oxidized lipids
Monoacyl polar lipids (Figure 10)	May be intermediates in lipid remodeling	DGMG and MGMG can be formed during head-group acylation of plastidic lipids	No direct evidence
Triacylglycerols (Figure 11)	Sequester free fatty acids removed during lipid remodeling	DGAT1 overexpression increases freezing tolerance [11,12]. The role of PDAT1 remains to be tested.	Moderate evidence
Sterol esters (Figure 12)	May serve as a reservoir for a small amount of fatty acids removed from membranes		No evidence
**Part 2. Positive Correlations of Level at 74 h with Final Ion Leakage, i.e., Associated with Poor Outcome.**			
**Hypothesis: *Decreasing the amounts of “Lipid or lipid group” produced during freezing will increase freezing tolerance*.**			
**Lipid or Lipid Group**	**Possible Role in Freezing Sensitivity or Tolerance**	**Supporting Evidence from Previous Work/Comments**	**Status**
Some MGDGs without full unsaturation (Figure 5)	May cause chloroplast membranes to be too rigid at low temperatures, causing photoinhibition	Defects in chloroplast desaturases lead to photoinhibition and poor growth in cold [2,9].	Good evidence for effect in cold, but effect in freezing is less clear
PAs not containing 16:3 (Figure 7)	May destabilize extra-plastidic membranes by facilitating hexagonal phase formation	PLDα1 suppression increases freezing survival and decreases ion leakage [4]. Knockout of DGKs decreases freezing tolerance [11].	Good evidence for effect, but mechanism of PA action is unproven
Non-oxidized head group-acylated MGDGs (Figure 8)	May destabilize membrane structure when present at high levels, as occurs after freezing		No evidence
Non-oxidized acyl sterol hexosides (Figure 12)	May destabilize extra-plastidic membranes by facilitating hexagonal phase formation	Acyl sterol hexosides decrease in cold acclimation and are present at lower levels in the plasma membrane of a freeze-tolerant rye than in a freeze-susceptible oat [50].	Associative evidence only

## Data Availability

Data supporting reported results can be found in the supplementary data.

## Supplementary Materials

The following supporting information can be downloaded at: https://www.mdpi.com/article/10.3390/metabo12050385/s1, Supplemental Methods; Figure S1: Positive and negative correlations of lipid levels with ion leakage at the final (98 h) time point; Figure S2: Time courses of levels of selected polar structural lipids in rosettes of control, non-acclimated, and acclimated plants; Figure S3: Time courses of levels of polygalactosylated lipids in rosettes of control, non-acclimated, and acclimated plants; Figure S4: Time courses of levels of selected phosphatidic acids in rosettes of control, non-acclimated, and acclimated plants; Figure S5: Time courses of levels of selected acylated chloroplast lipids in rosettes of control, non-acclimated, and acclimated plants: Figure S6: Time courses of levels of selected oxidized polar lipids in rosettes of control, non-acclimated, and acclimated plants; Figure S7: Time courses of levels of selected monoacyl polar lipids in rosettes of control, non-acclimated, and acclimated plants; Figure S8: Time courses of levels of neutral glycerolipids in rosettes of control, non-acclimated, and acclimated plants: Figure S9: Time courses of levels of selected sterol derivatives in rosettes of control, non-acclimated, and acclimated plants: Figure S10: Time courses of levels of ion leakage and selected lipids in rosettes of control, non-acclimated, and acclimated wild-type and opr3 plants; Figure S11: Positions of seeds/plants in trays: Figure S12: Numbering on tray F1.01; Table S1: Rosette lipid amounts from the freezing time course of wild-type plants; Table S2: Statistically significant pairwise comparisons of lipid levels and ion leakage between treatments at particular time points in wild-type Arabidopsis rosettes; Table S3: Internal standards; Table S4: Lipid nomenclature used in paper; Table S5: Significant correlations of lipids from wild-type plants from various time points with final ion leakage, average amounts of rosette lipids in plants at selected time points and treatments, and ratios of lipid levels from non-acclimated plants over control plants and acclimated plants over control plants at time points after treatments; Table S6: Limit of detection (LOD) and coefficient of variation (CoV, standard deviation/average) for the lipids analyzed and reported; Table S7: Arabidopsis lines used; Table S8: Rosette lipid amounts from the freezing time course of plants of all genotypes; Table S9: Statistically significant pairwise comparisons of lipid levels between wild-type and mutant Arabidopsis rosettes undergoing the same treatment; Table S10: Leaf numbers of Arabidopsis rosettes of various genotypes at 20 days and 27 days; Table S11: Averages and standard deviations for leaf numbers and masses of Arabidopsis rosettes of various genotypes; Table S12: Positions of sample vials on a mass spectrometry sample tray; Table S13: Experimental parameters for lipids analyzed. References [61,62] are cited in the supplementary materials.
