# Correction: Vu et al. Specific Changes in *Arabidopsis thaliana* Rosette Lipids during Freezing Can Be Associated with Freezing Tolerance. *Metabolites* 2022, *12*, 385

**DOI:** 10.3390/metabo13040499

**Published:** 2023-03-30

**Authors:** Hieu Sy Vu, Sunitha Shiva, Thilani Samarakoon, Maoyin Li, Sujon Sarowar, Mary R. Roth, Pamela Tamura, Samuel Honey, Kaleb Lowe, Hollie Porras, Neema Prakash, Charles A. Roach, Morgan Stuke, Xuemin Wang, Jyoti Shah, Gary Gadbury, Haiyan Wang, Ruth Welti

**Affiliations:** 1 Kansas Lipidomics Research Center, Division of Biology, Kansas State University, 1717 Claflin Rd., Manhattan, KS 66506, USA; 2 Donald Danforth Plant Science Center, 975 N Warson Rd., St. Louis, MO 63132, USA; 3 Department of Biology, University of Missouri-St. Louis, St. Louis, MO 63121, USA; 4 Department of Biological Sciences, University of North Texas, 1155 Union Circle #305220, Denton, TX 76203, USA; 5 Department of Statistics, Kansas State University, Manhattan, KS 66506, USA

There was an error in the original publication [1]. The enzymatic step catalyzed by oxophytodienoic acid reductase 3 was misstated. A correction has been made to Section 2. Results and Discussion, Subsection 2.13. Lipids of Plants with Mutations in Lipid-Related Genes, Paragraph 1.

*2.13. Lipids of Plants with Mutations in Lipid-Related Genes*

Lipid levels in plants with mutations in genes encoding patatin-like phospholipases, lipoxygenases and OXOPHYTODIENOIC ACID REDUCTASE 3 (OPR3) were compared with those of wild-type plants at each time point and treatment (i.e., on each tray). The list of mutants can be found in Table S7, with the nomenclature system for patatin-like lipases following that in Chen et al. [41]. Ion leakage and lipid level data are shown in Table S8. Using the conservative Bonferroni correction, only 177 significant lipid differences were identified [52,53] (Table S9). Ion leakage did not differ significantly among the genotypes. Of the observed differences, 139 were between lipids in wild-type plants and those in *opr3*, while the other 38 differences from wild-type lipid levels were distributed among lipid levels of several other mutants (Table S9). The time courses of levels of selected lipids that have significant differences between wild-type and *opr3* rosettes are shown for these genotypes in Figures 13 and S10. Two major groups of lipids are different between wild type and *opr3* (Figure 13a,b). Lipids containing OPDA are significantly lower in *opr3* than in the wild type, but the difference is observed primarily after the freezing treatment. Oxophytodienoic acid reductase 3 catalyzes the first step in the production of jasmonic acid from OPDA. The reason for the lower levels of OPDA is not clear, but the magnitude of the reduction in Arabidopsides A and B to about half of their wild-type levels is consistent with data from plants with the same mutation in OPR3 tested during the wounding response [54]. The levels of sterol esters are higher in *opr3* than in wild-type plants, and this is true regardless of control, cold, freezing, or recovery treatments (Figure 13c,d). The basis for the higher levels of sterol esters in *opr3* is not clear, but it is conceivable that a product of OPR3 could serve as a negative regulator of sterol ester biosynthesis.

The authors state that the scientific conclusions are unaffected. This correction was approved by the Academic Editor. The original publication has also been updated.
