# Supplementary material for: Specific Changes in Arabidopsis thaliana Rosette Lipids during Freezing Can Be Associated with Freezing Tolerance
Source: Metabolites. 2022 Apr 23;12(5):385. doi: 10.3390/metabo12050385 (PMC9145600; doi:10.3390/metabo12050385)

Figure S1. Positive and negative correlations of lipid levels with ion leakage at the final (98-h) time point. Positive correlation indicates that higher levels of the lipid are associated with more ion leakage (i.e., a deleterious effect), while negative correlations indicate that higher levels of the lipid are associated with lower ion leakage (potentially a beneficial effect). A. Lipid levels at 72 h. B. Lipid levels at 75 h. C. Lipid levels at 77 h.

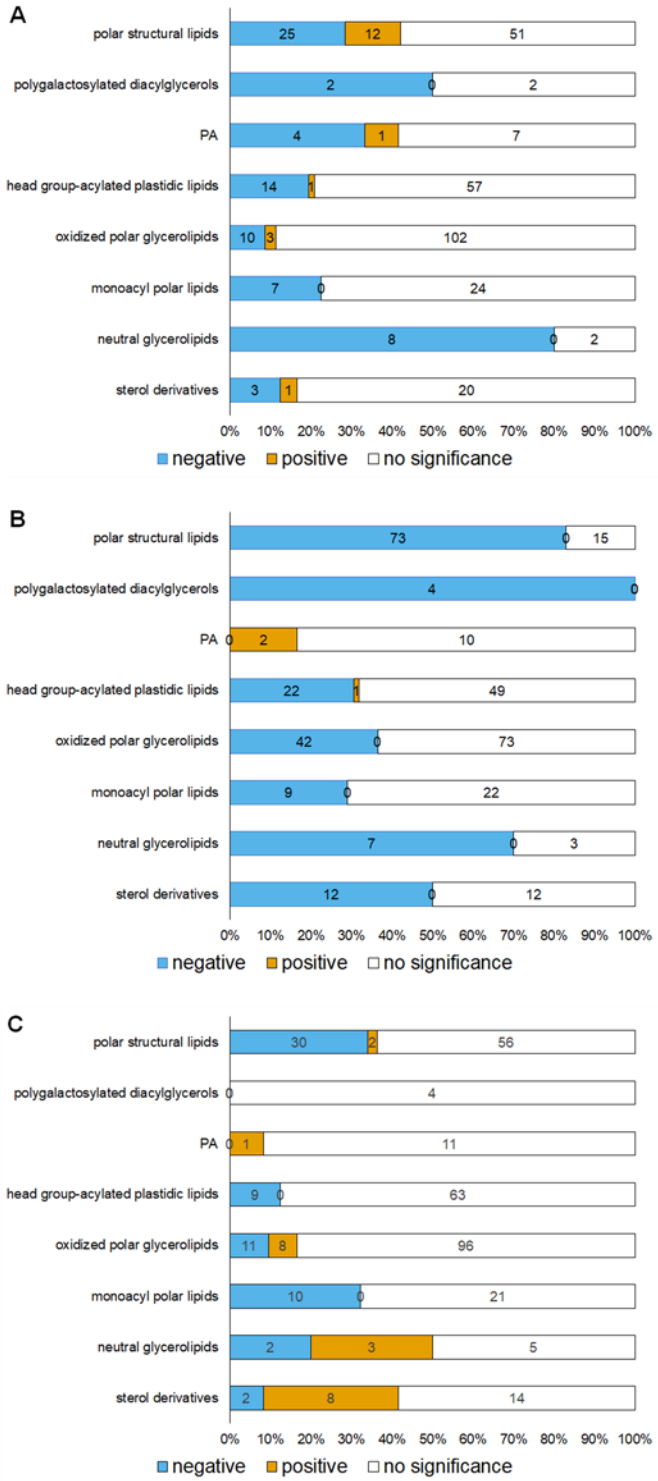

Figure S2. Time courses of levels of selected polar structural lipids in rosettes of control, non-acclimated, and acclimated plants. Treatments are shown in Figure 1. Asterisks indicate lipids with quality control (pooled sample) levels less than 0.75 (*) or 0.25 (**) units of normalized mass spectral intensity, where 1 = intensity of 1 pmol of internal standard. "C" indicates that the lipid level in non-acclimated or acclimated plants is significantly different than the control level, and "A" indicates that the lipid level in non-acclimated plants is significantly different than the level in acclimated plants (Table S2). Indicated on each plot is whether there is significant correlation of lipid level at the 74, 75, and 77 h time points with final (98 h) ion leakage.

Lipid number	Panel	Class, oxidation	Lipid name
**non-oxidized DGDG**			
lipid0164	2A	DGDG, non-oxidized	DGDG 34:3
lipid0163	2A	DGDG, non-oxidized	DGDG 34:4
lipid0170	2B	DGDG, non-oxidized	DGDG 36:3
lipid0169	2B	DGDG, non-oxidized	DGDG 36:4
lipid0168	2C	DGDG, non-oxidized	DGDG 36:5
lipid0167	2C	DGDG, non-oxidized	DGDG 36:6
lipid0174	2D	DGDG, ambiguous	DGDG 38:5 or DGDG 36:7;O2
**non-oxidized MGDG**			
lipid0143	2D	MGDG, non-oxidized	MGDG 34:3
lipid0142	2E	MGDG, non-oxidized	MGDG 34:4
lipid0141	2E	MGDG, non-oxidized	MGDG 34:5
lipid0140	2F	MGDG, non-oxidized	MGDG 34:6
lipid0808	2F	MGDG, non-oxidized	MGDG 35:3
lipid0149	2G	MGDG, non-oxidized	MGDG 36:3
lipid0148	2G	MGDG, ambiguous	MGDG 36:4 or MGDG 34:6;O2
lipid0147	2H	MGDG, ambiguous	MGDG 36:5 or MGDG 34:7;O2
lipid0146	2H	MGDG, ambiguous	MGDG 36:6 or MGDG 34:8;O2
lipid0153	2I	MGDG, ambiguous	MGDG 38:5 or MGDG 36:7;O2
**non-oxidized PG**			
lipid0008	2I	PG, non-oxidized	PG 32:0
lipid0007	2J	PG, non-oxidized	PG 32:1
lipid0012	2J	PG, non-oxidized	PG 34:1
lipid0011	2K	PG, non-oxidized	PG 34:2
lipid0010	2K	PG, non-oxidized	PG 34:3
lipid0009	2L	PG, non-oxidized	PG 34:4
lipid0018	2L	PG, non-oxidized	PG 36:2
lipid0017	2M	PG, non-oxidized	PG 36:3
lipid0016	2M	PG, non-oxidized	PG 36:4

lipid0015	2N	PG, non-oxidized	PG 36:5
lipid0014	2N	PG, non-oxidized	PG 36:6
**non-oxidized PC**			
lipid0841	2O	PC, non-oxidized	PC 32:1
lipid0840	2O	PC, non-oxidized	PC 32:3
lipid0035	2P	PC, non-oxidized	PC 34:1
lipid0034	2P	PC, non-oxidized	PC 34:2
lipid0842	2Q	PC, non-oxidized	PC 34:6
lipid0040	2Q	PC, non-oxidized	PC 36:2
lipid0039	2R	PC, non-oxidized	PC 36:3
lipid0038	2R	PC, non-oxidized	PC 36:4
lipid0037	2S	PC, non-oxidized	PC 36:5
lipid0046	2S	PC, non-oxidized	PC 38:2
lipid0045	2T	PC, non-oxidized	PC 38:3
lipid0044	2T	PC, non-oxidized	PC 38:4
lipid0043	2U	PC, non-oxidized	PC 38:5
lipid0042	2U	PC, non-oxidized	PC 38:6
lipid0050	2V	PC, non-oxidized	PC 40:2*
lipid0049	2V	PC, non-oxidized	PC 40:3*
lipid0048	2W	PC, non-oxidized	PC 40:4*
lipid0047	2W	PC, non-oxidized	PC 40:5*
**non-oxidized PE**			
lipid0063	2X	PE, non-oxidized	PE 34:2
lipid0062	2X	PE, non-oxidized	PE 34:3
lipid0069	2Y	PE, non-oxidized	PE 36:2
lipid0068	2Y	PE, non-oxidized	PE 36:3
lipid0067	2Z	PE, non-oxidized	PE 36:4
lipid0066	2Z	PE, non-oxidized	PE 36:5
lipid0065	2AA	PE, non-oxidized	PE 36:6
lipid0073	2AA	PE, non-oxidized	PE 38:4
lipid0072	2BB	PE, non-oxidized	PE 38:5
lipid0075	2BB	PE, non-oxidized	PE 40:3
lipid0079	2CC	PE, non-oxidized	PE 42:2
lipid0078	2CC	PE, non-oxidized	PE 42:3 (measured in positive mode)
**PS**			
lipid0095	2DD	PS, non-oxidized	PS 34:3
lipid0116	2DD	PS, non-oxidized	PS 42:2
lipid0115	2EE	PS, non-oxidized	PS 42:3
**sphingolipids**			
lipid0210	2EE	GIPC, non-oxidized	GIPC 42:1;O4
lipid0212	2FF	GIPC, non-oxidized	GIPC 42:2;O4

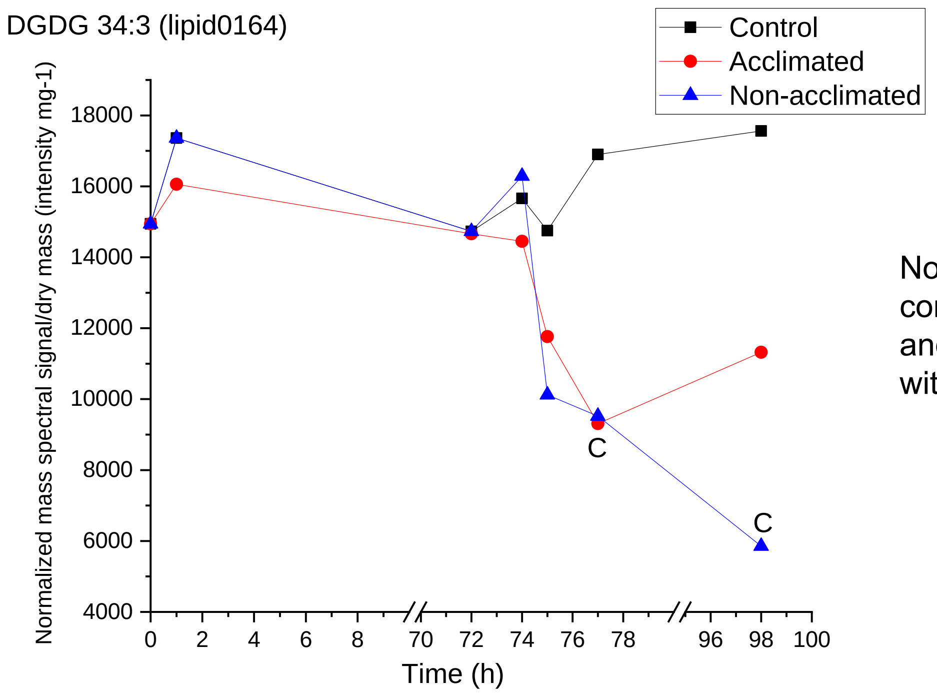

No significant correlation of 74, 75, and 77 h lipid levels with final ion leakage

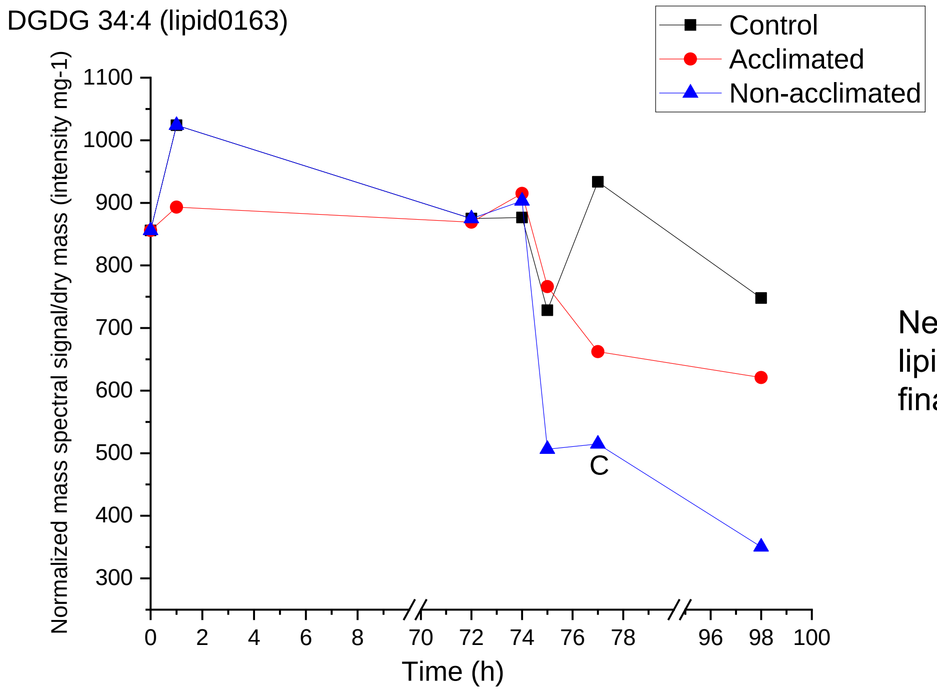

Negative correlation of lipid level at 75 h with final ion leakage

2A

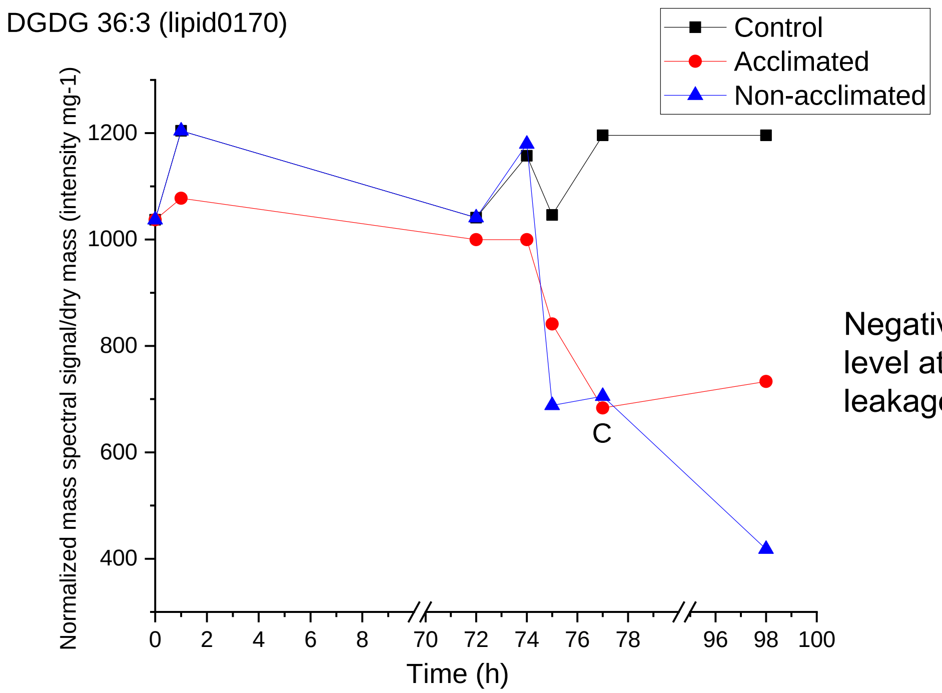

Negative correlation of lipid level at 75 h with final ion leakage

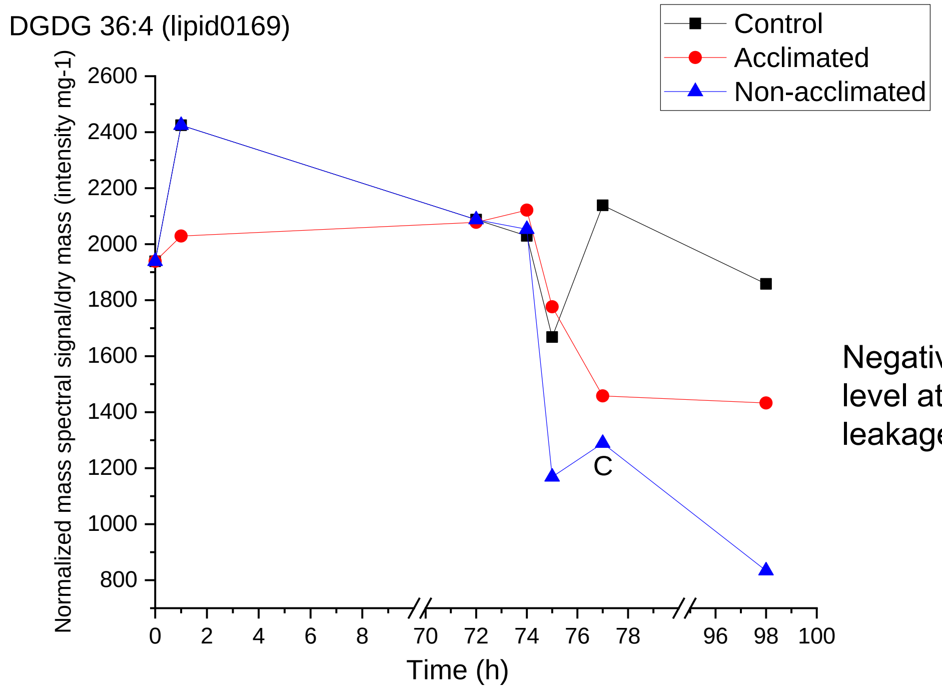

Negative correlation of lipid level at 75 h with final ion leakage

2B

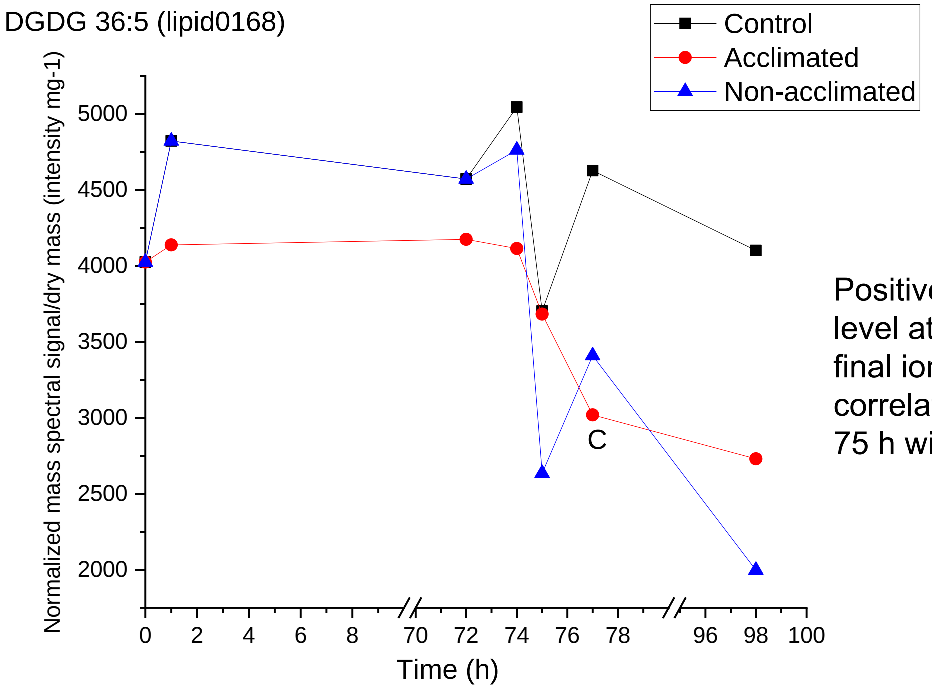

Positive correlation of lipid level at 74 h and 77 h with final ion leakage. Negative correlation of lipid level at 75 h with final ion leakage

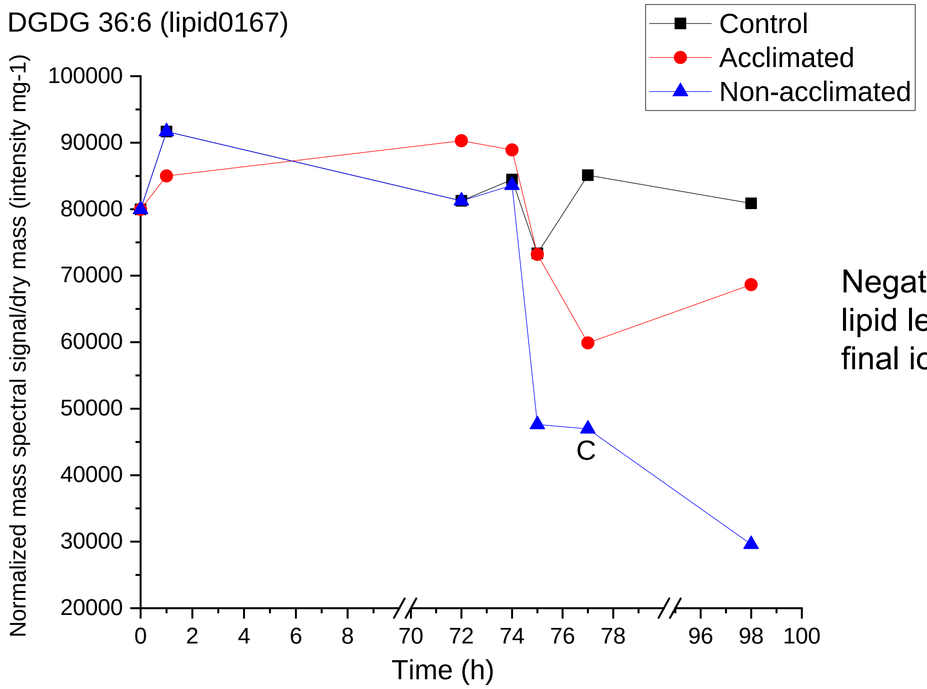

Negative correlation of lipid level at 75 h with final ion leakage

2C

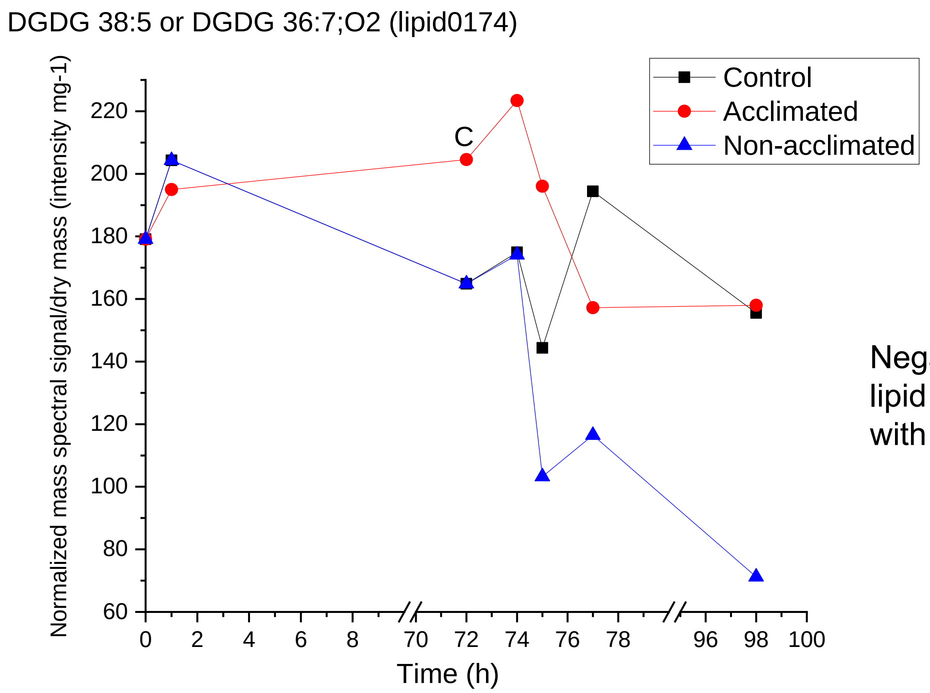

Negative correlation of lipid level at 74 and 75 h with final ion leakage

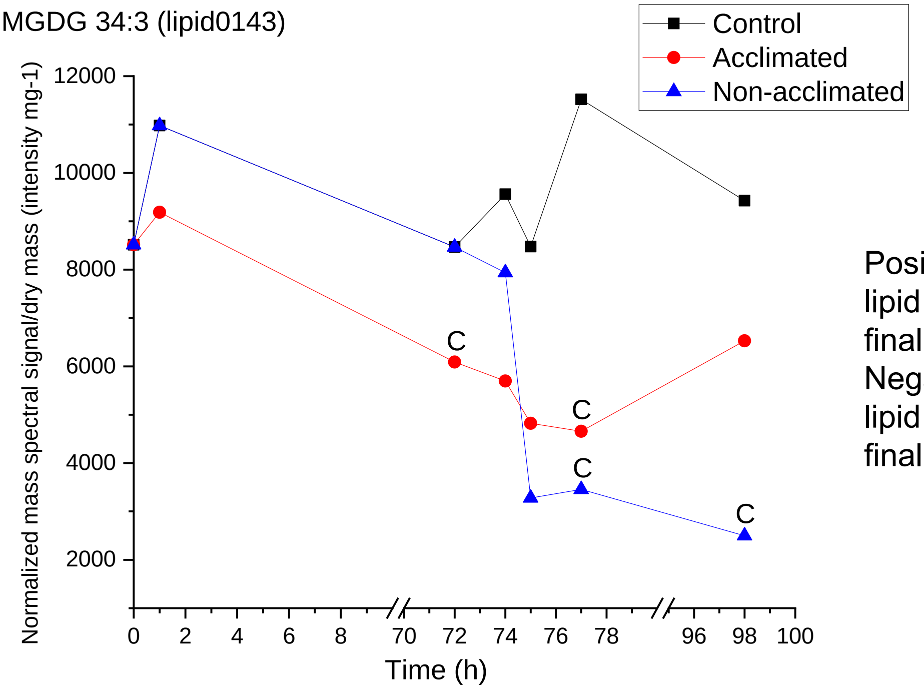

Positive correlation of lipid level at 74 h with final ion leakage. Negative correlation of lipid level at 75 h with final ion leakage

2D

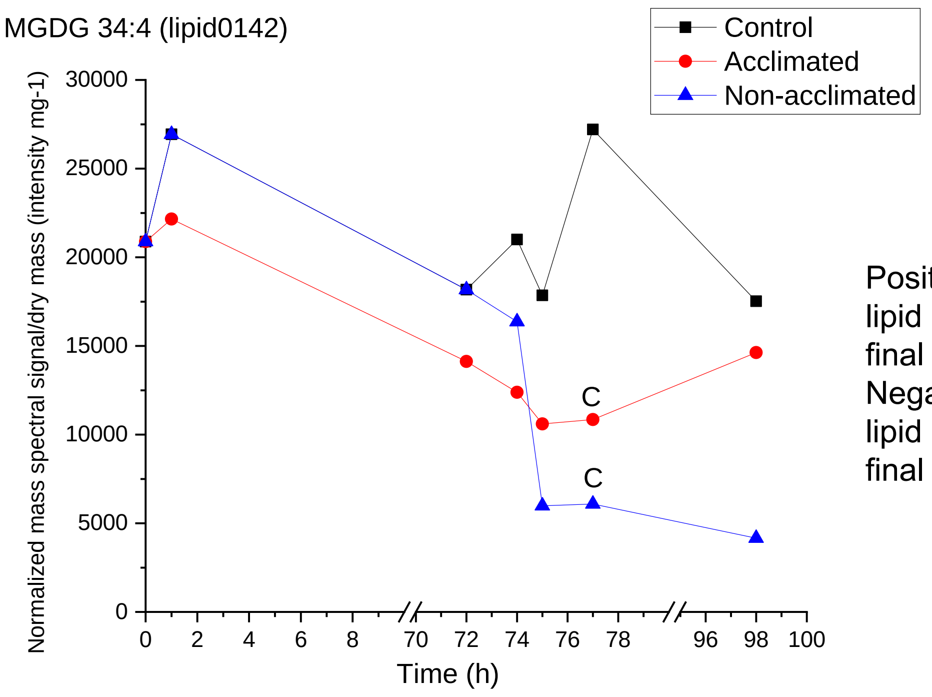

Positive correlation of lipid level at 74 h with final ion leakage. Negative correlation of lipid level at 75 h with final ion leakage

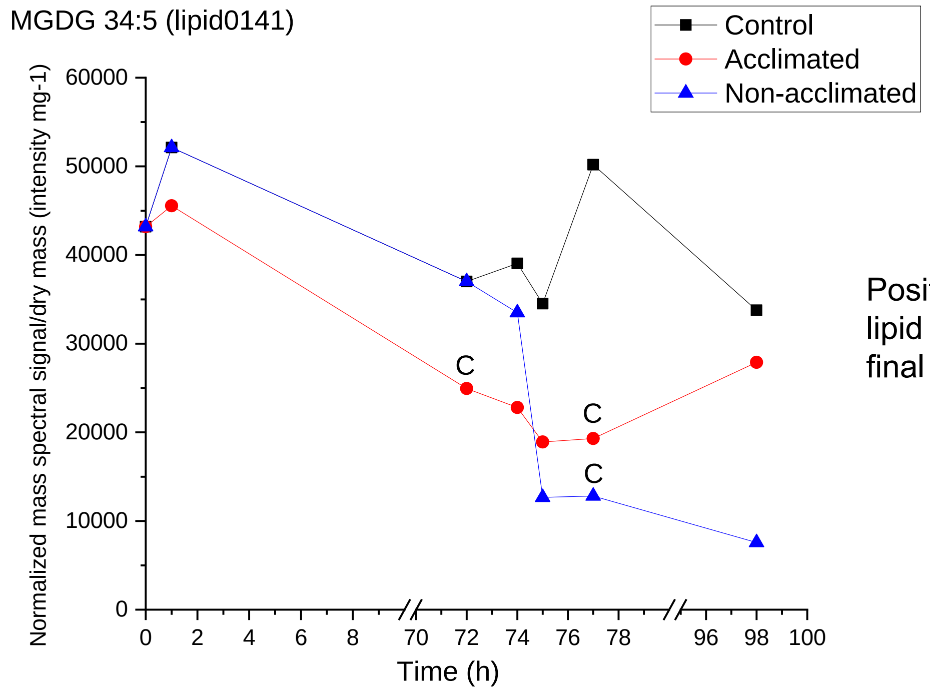

Positive correlation of lipid level at 74 h with final ion leakage

2E

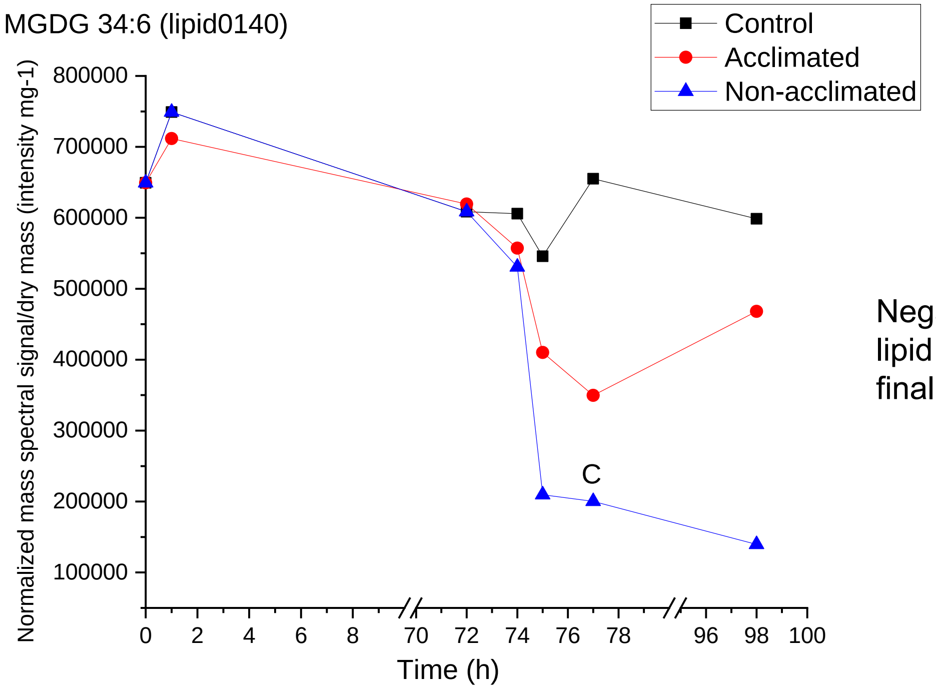

Negative correlation of lipid level at 75 h with final ion leakage

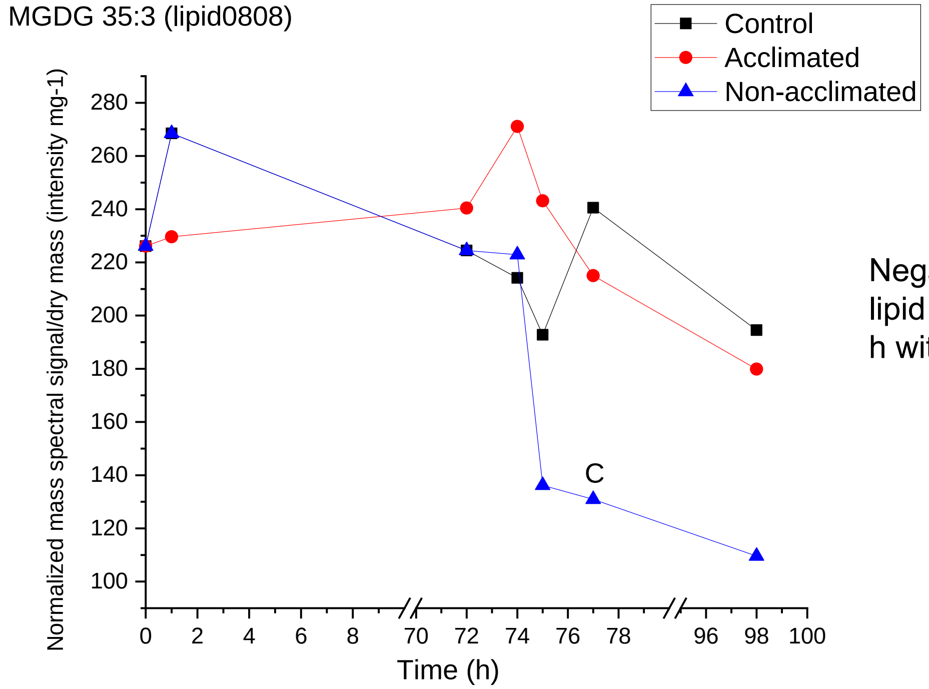

Negative correlation of lipid level at 74 and 75 h with final ion leakage

2F

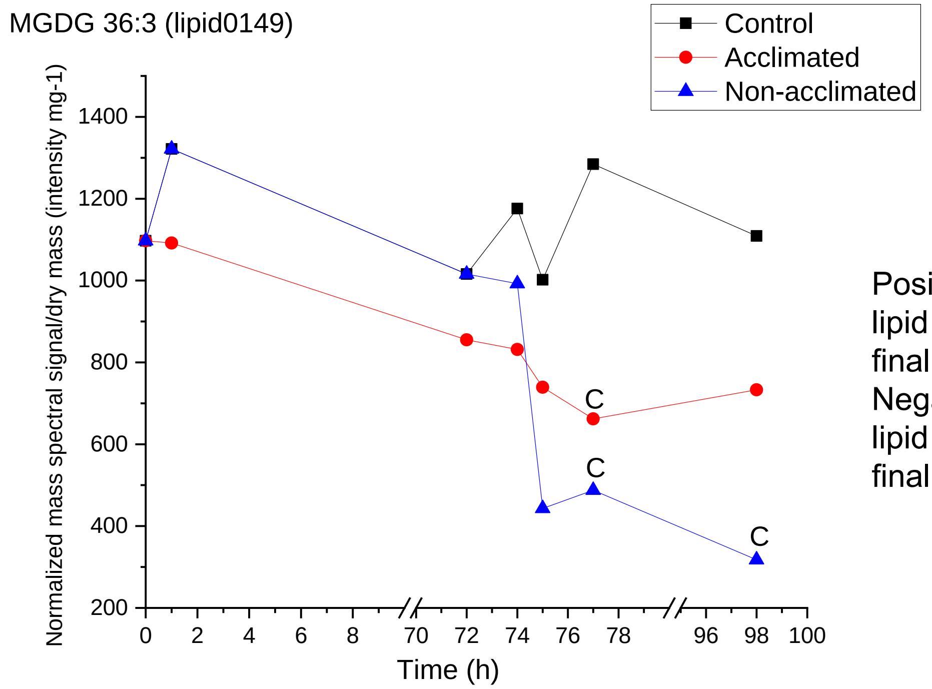

Positive correlation of lipid level at 74 h with final ion leakage
Negative correlation of lipid level at 75 h with final ion leakage

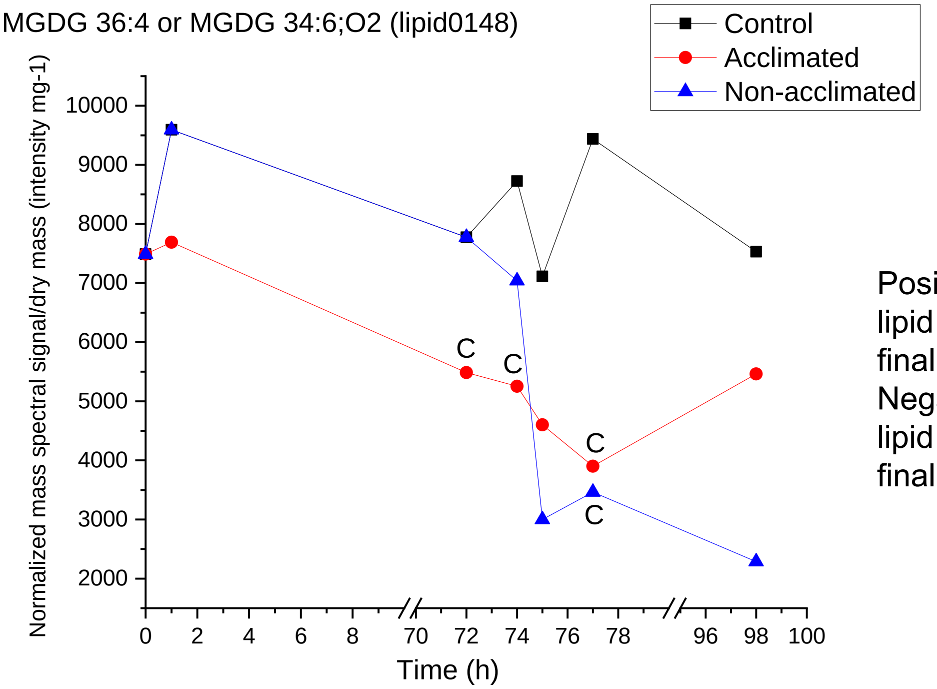

Positive correlation of lipid level at 74 h with final ion leakage
Negative correlation of lipid level at 75 h with final ion leakage

2G

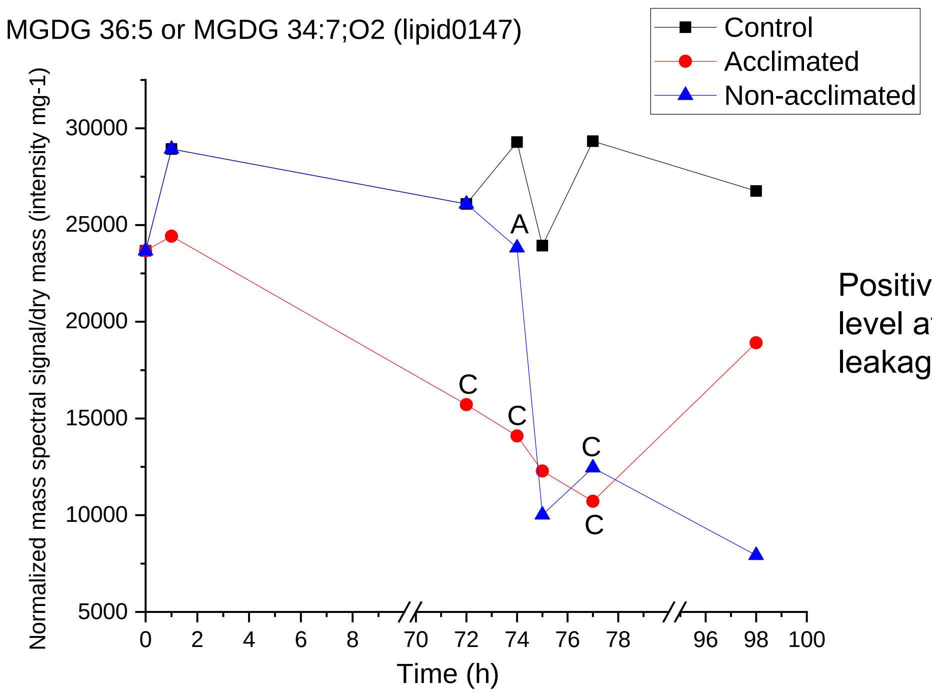

Positive correlation of lipid level at 74 h with final ion leakage

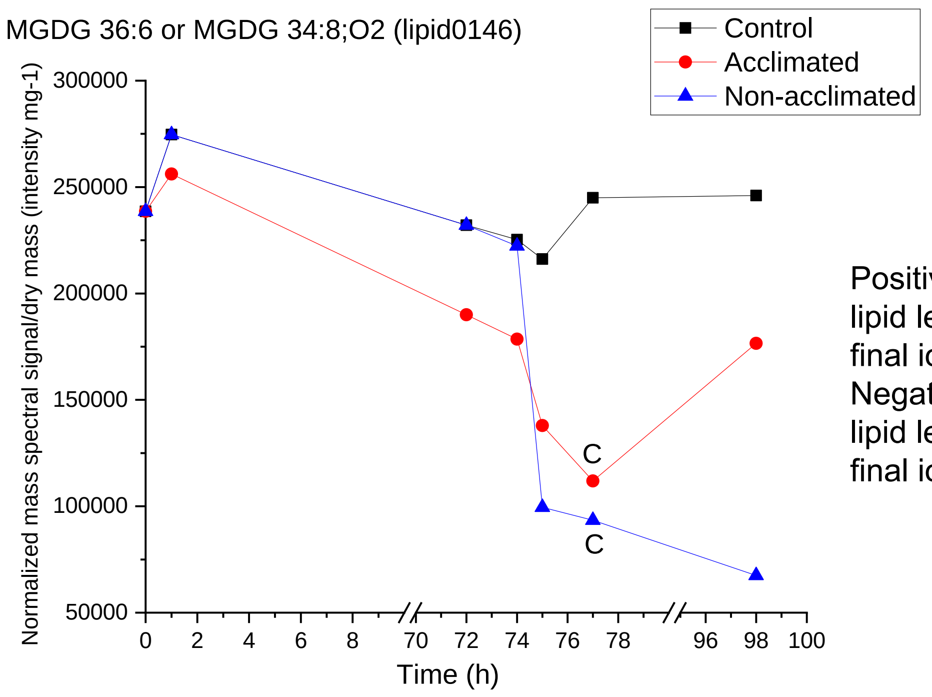

Positive correlation of lipid level at 74 h with final ion leakage
Negative correlation of lipid level at 75 h with final ion leakage

2H

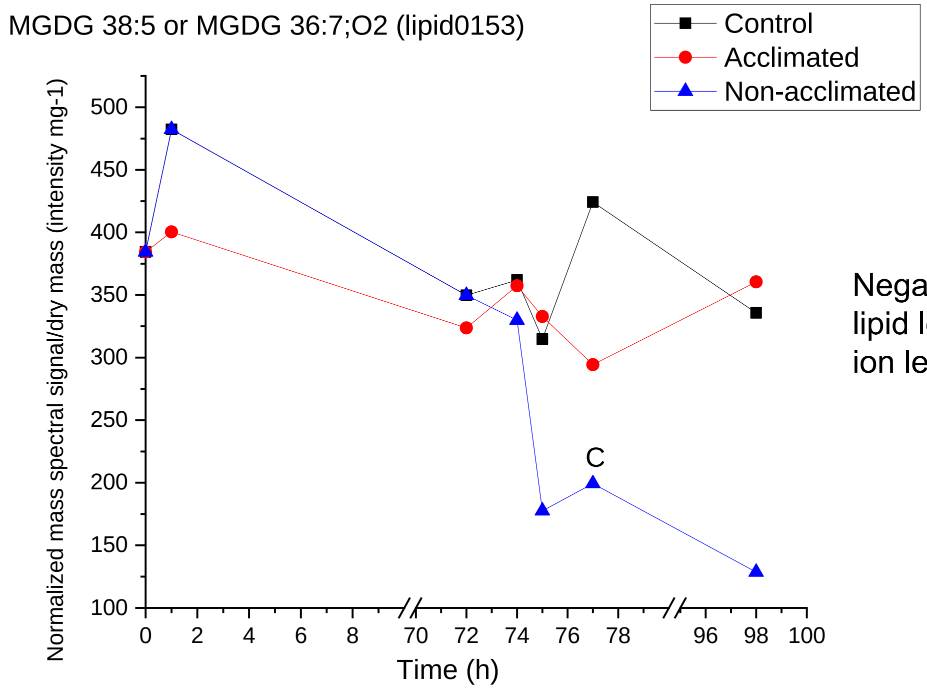

Negative correlation of lipid level at 75 h with final ion leakage

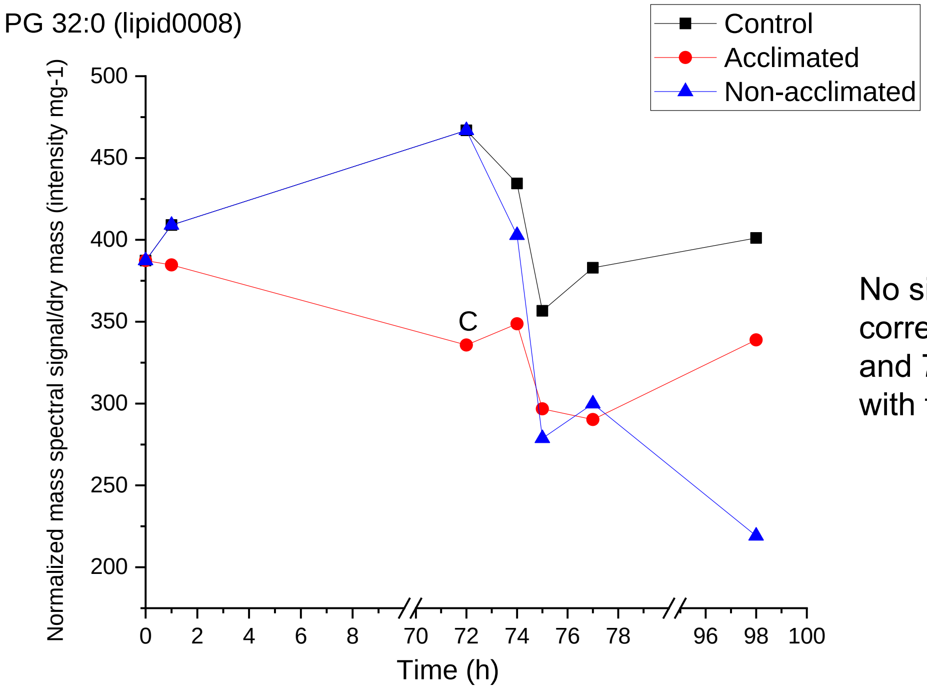

No significant correlation of 74, 75, and 77 h lipid levels with final ion leakage

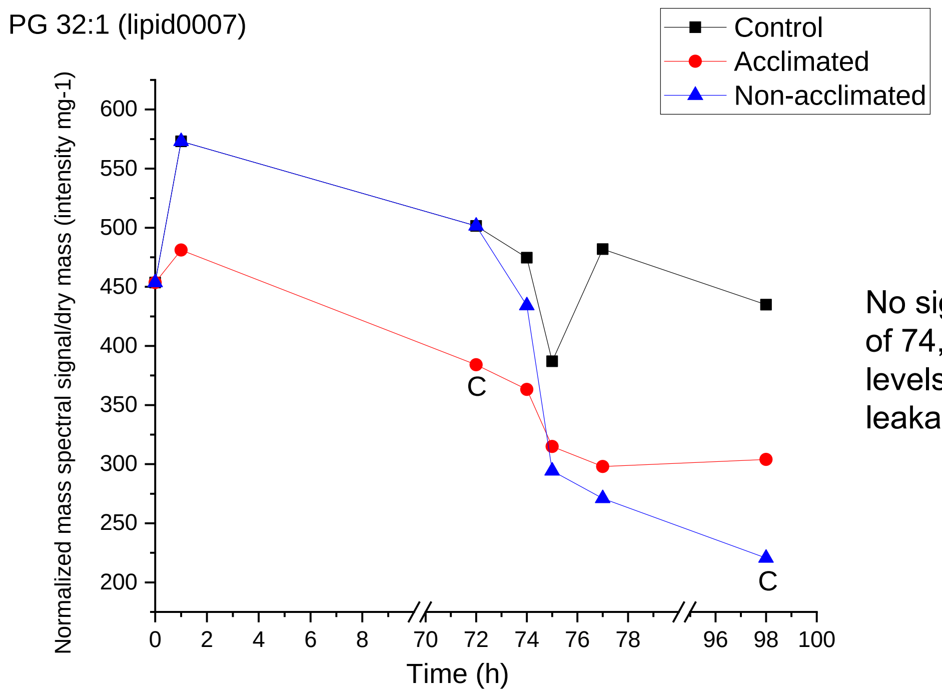

No significant correlation of 74, 75, and 77 h lipid levels with final ion leakage

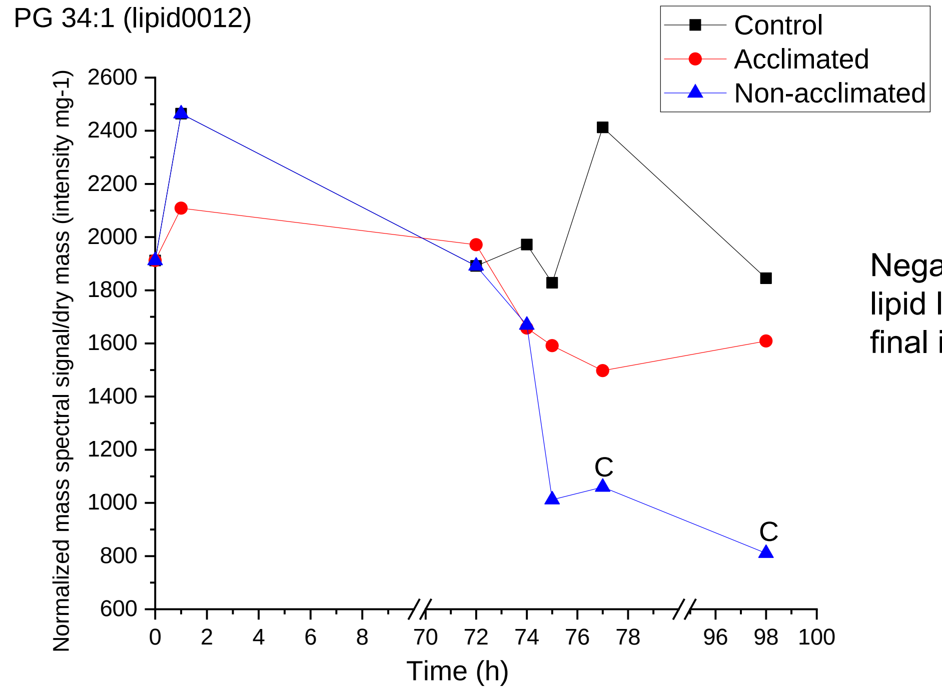

Negative correlation of lipid level at 75 h with final ion leakage

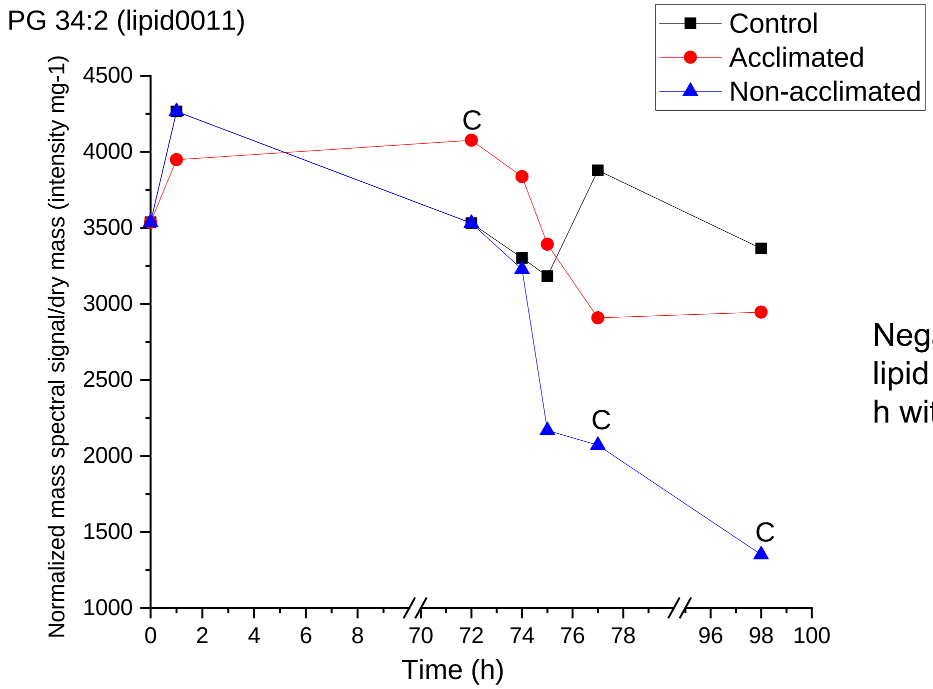

Negative correlation of lipid level at 74 and 75 h with final ion leakage

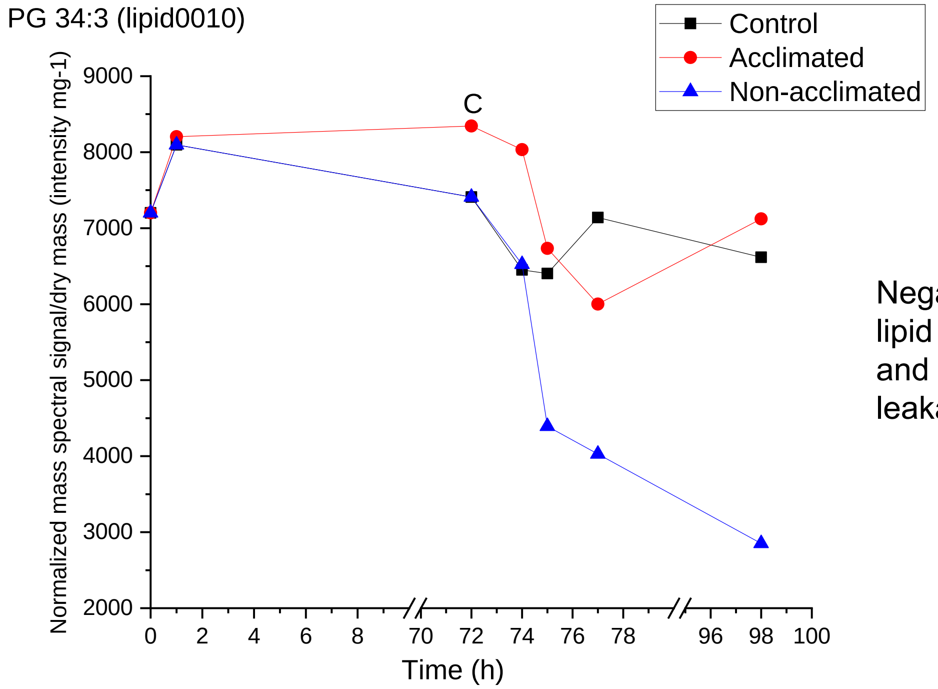

Negative correlation of lipid level at 74, 75, and 77 h with final ion leakage

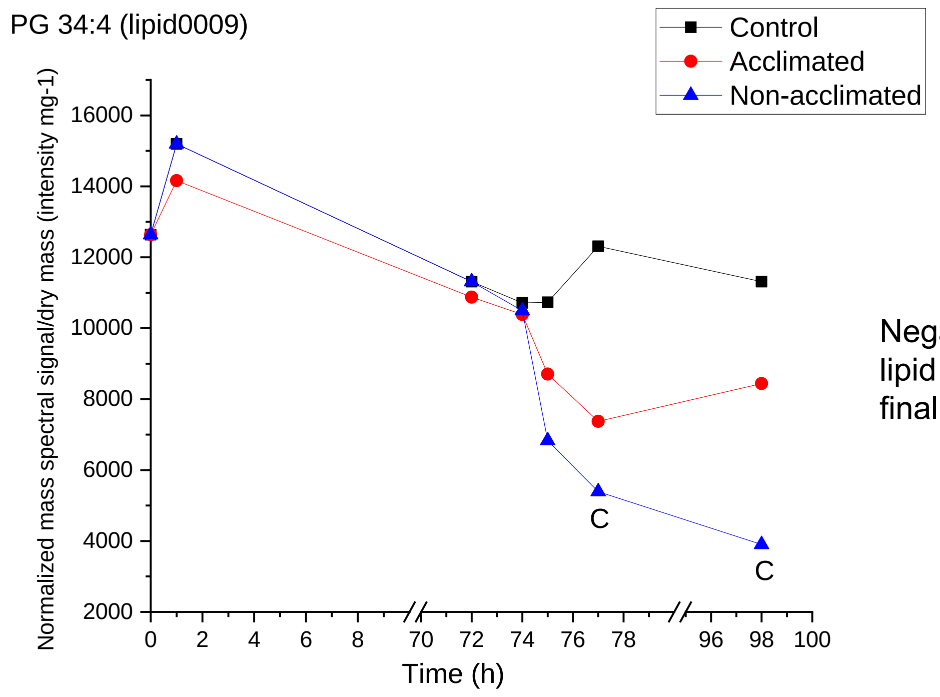

Negative correlation of lipid level at 75 h with final ion leakage

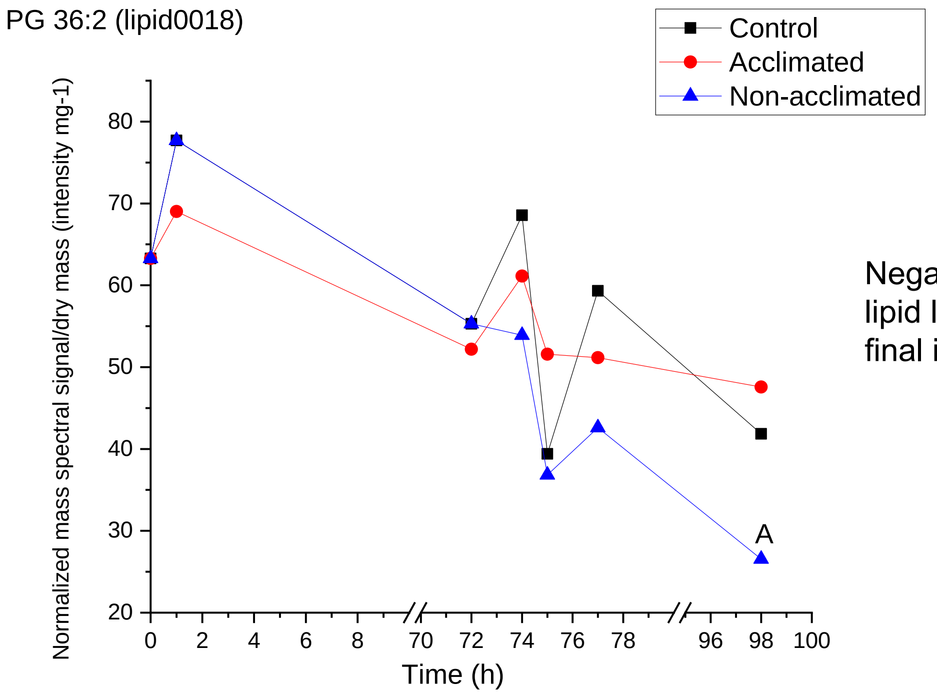

Negative correlation of lipid level at 75 h with final ion leakage

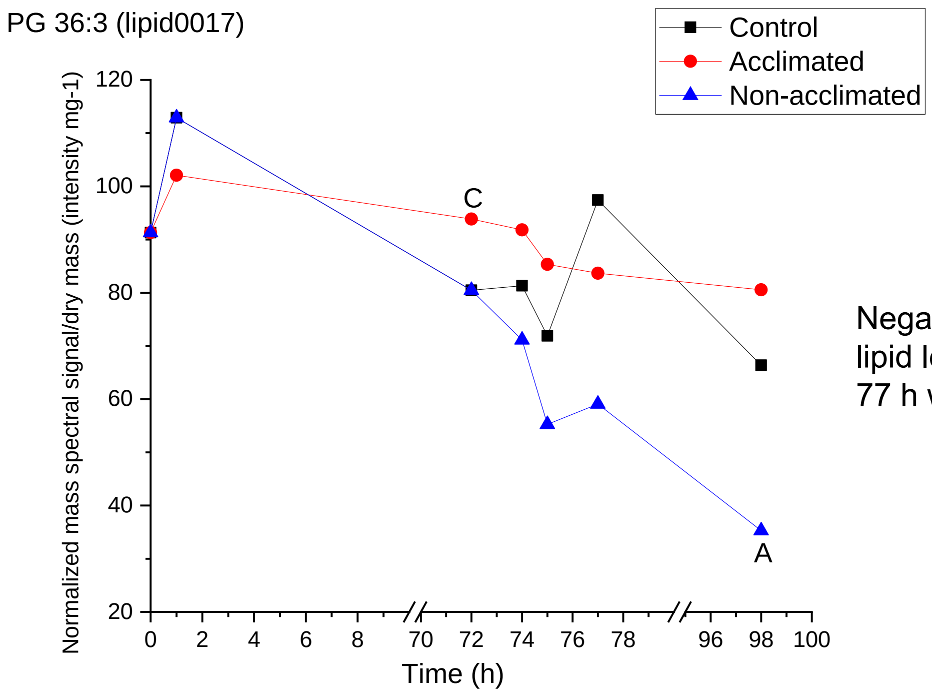

Negative correlation of lipid level at 74, 75, and 77 h with final ion leakage

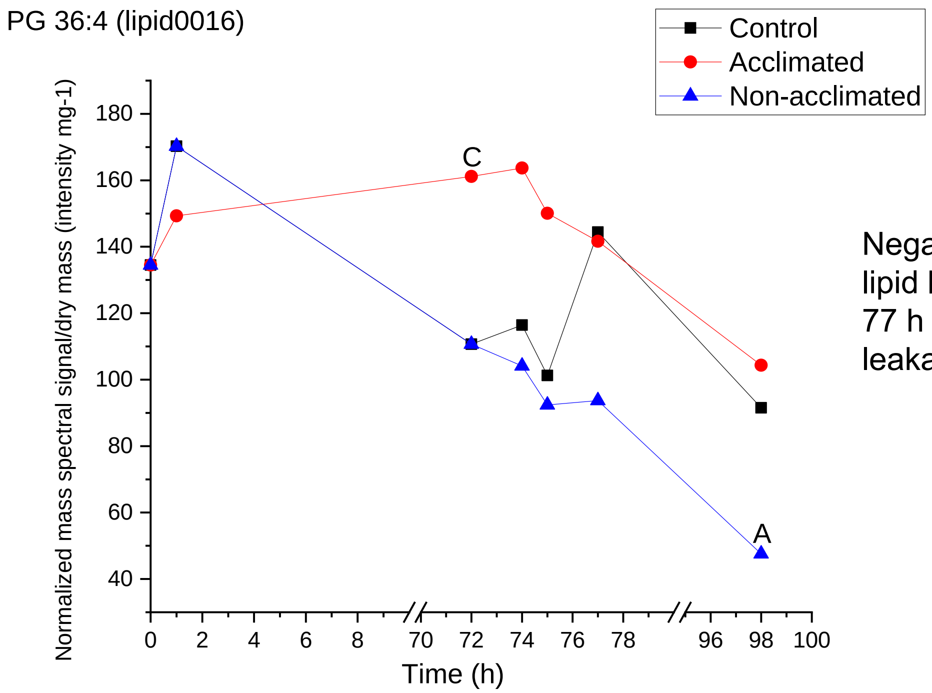

Negative correlation of lipid level at 74, 75, and 77 h with final ion leakage

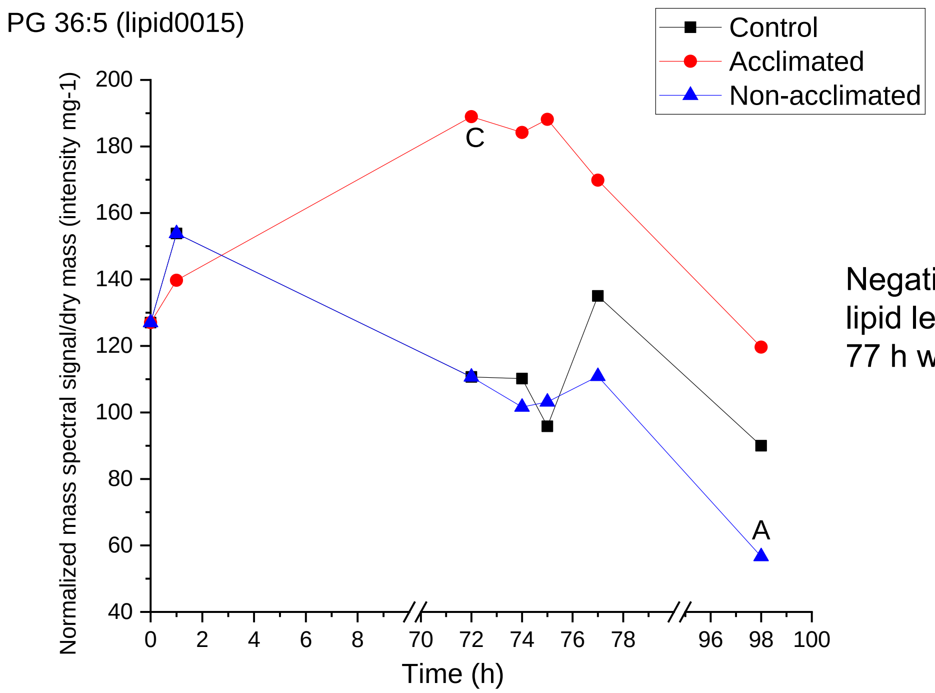

Negative correlation of lipid level at 74, 75, and 77 h with final ion leakage

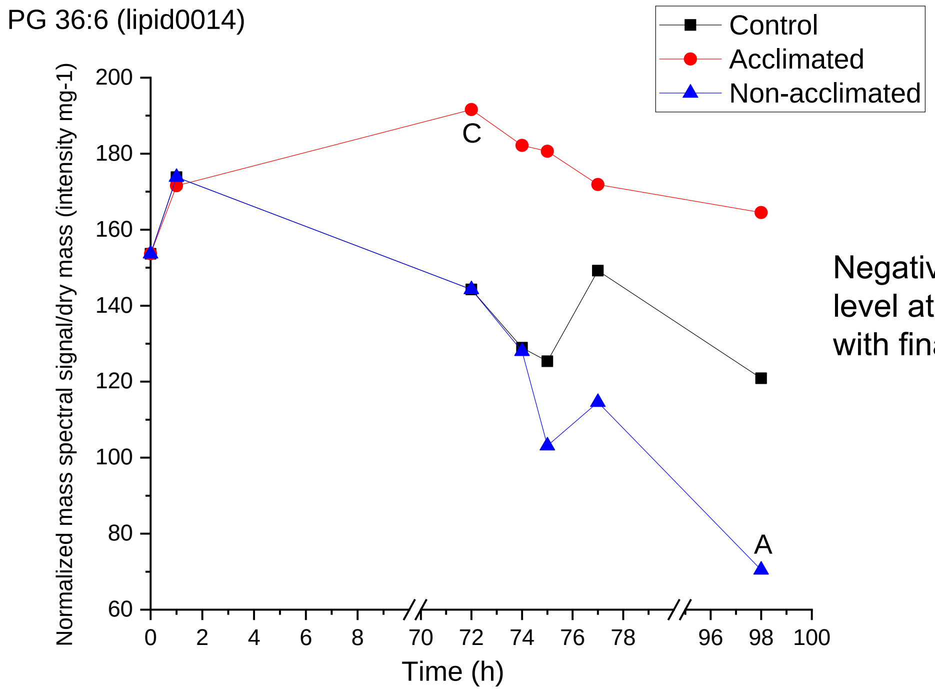

Negative correlation of lipid level at 74, 75, and 77 h with final ion leakage

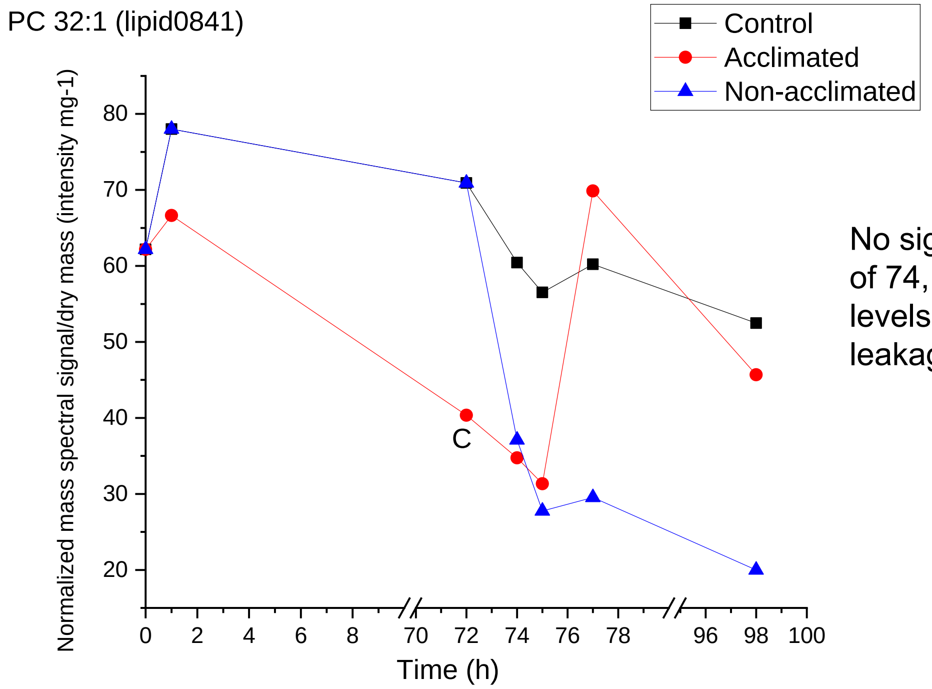

No significant correlation of 74, 75, and 77 h lipid levels with final ion leakage

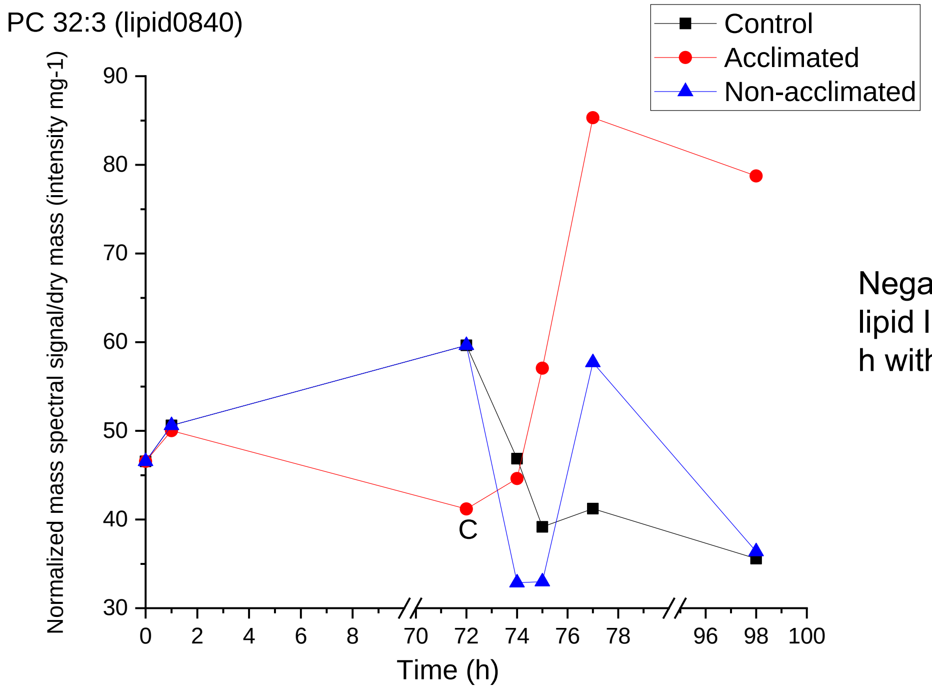

Negative correlation of lipid level at 75 and 77 h with final ion leakage

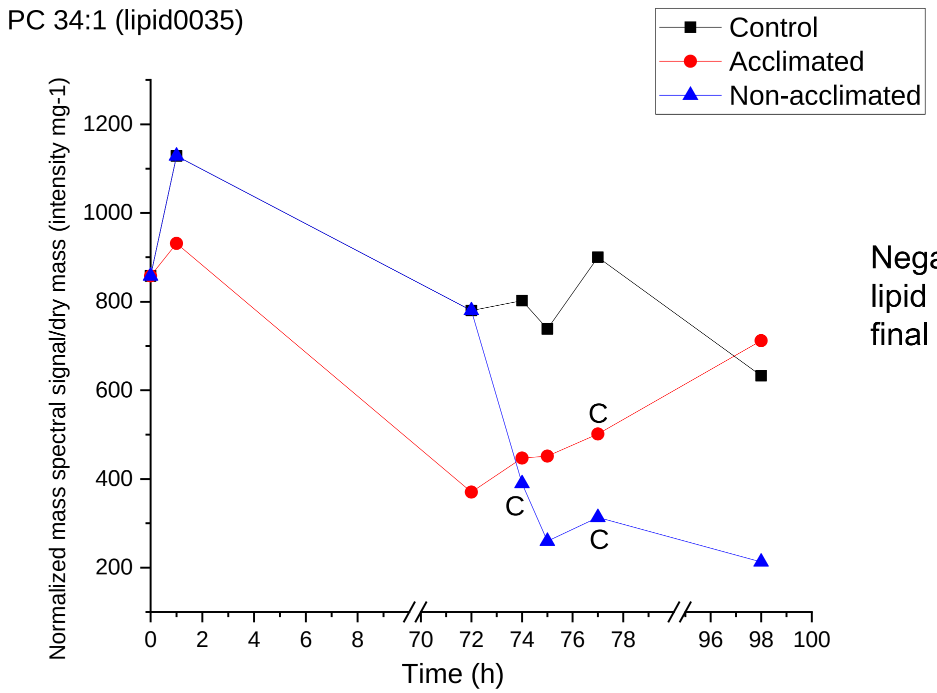

Negative correlation of lipid level at 75 h with final ion leakage

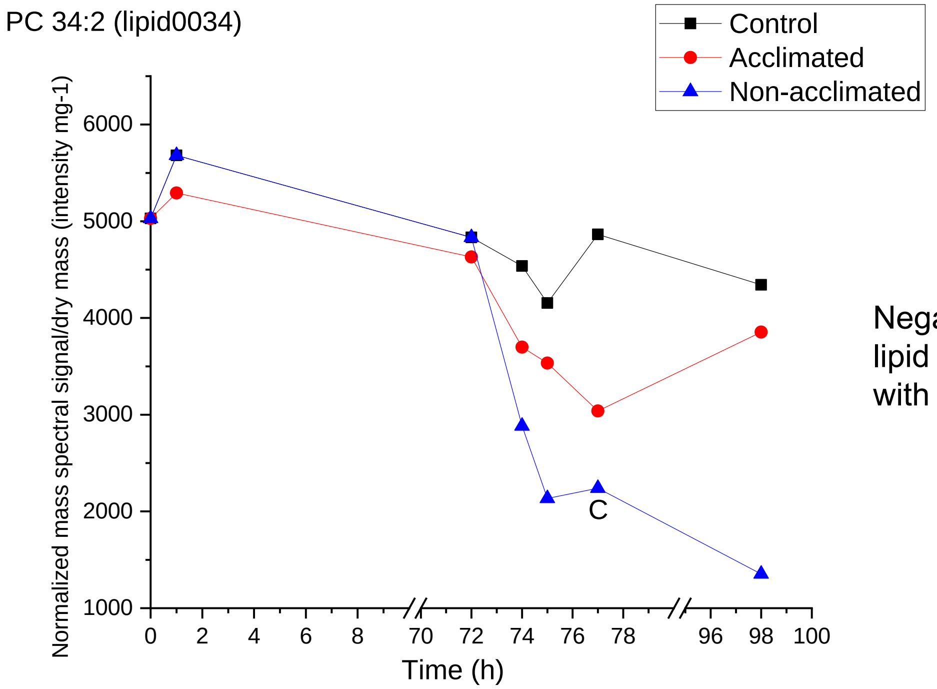

Negative correlation of lipid level at 74 and 75 h with final ion leakage

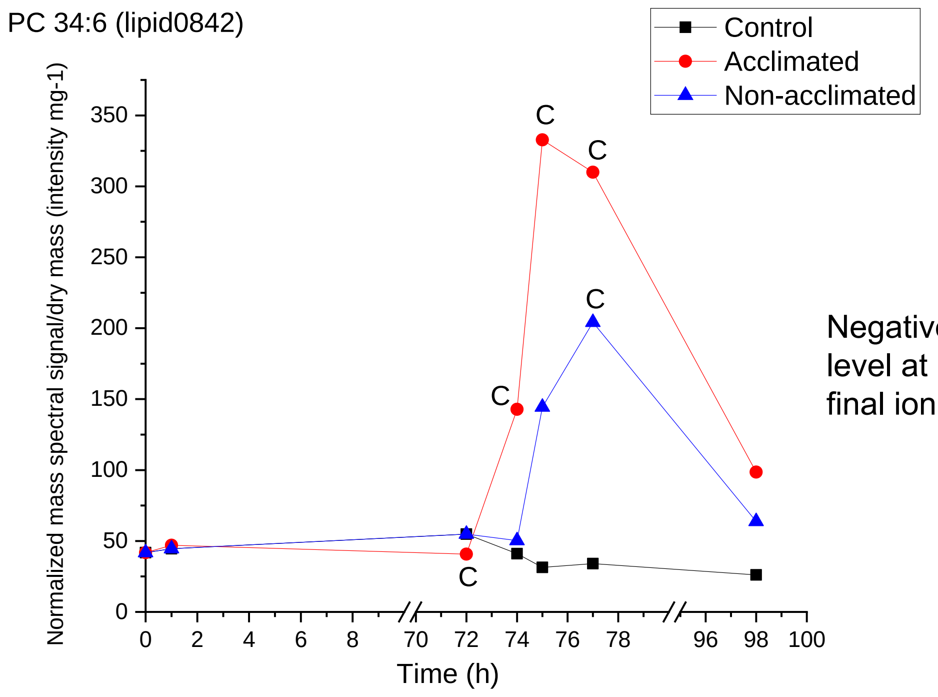

Negative correlation of lipid level at 74 and 75 h with final ion leakage

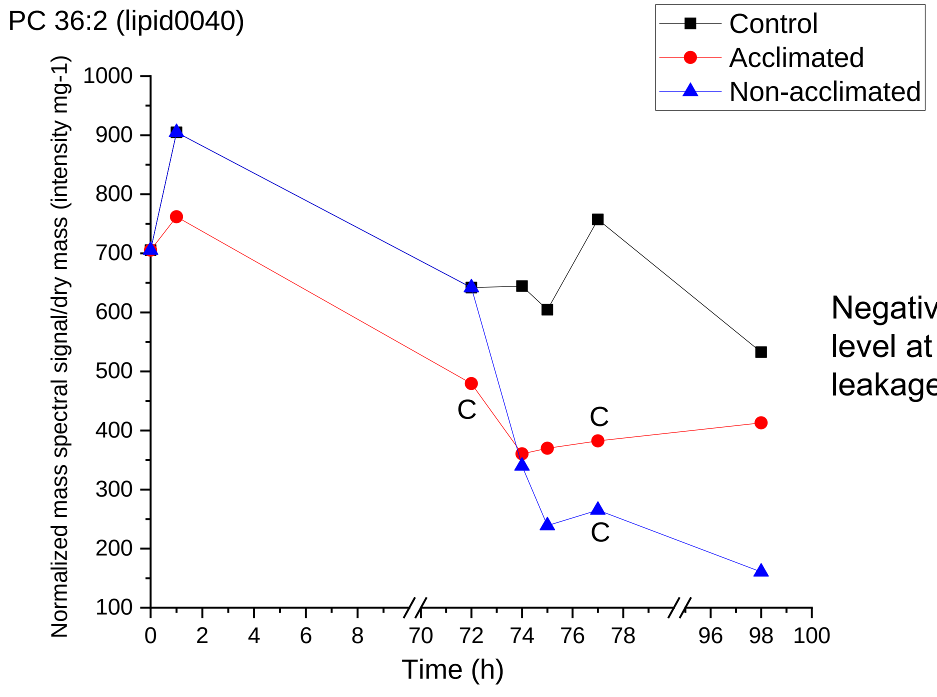

Negative correlation of lipid level at 75 h with final ion leakage

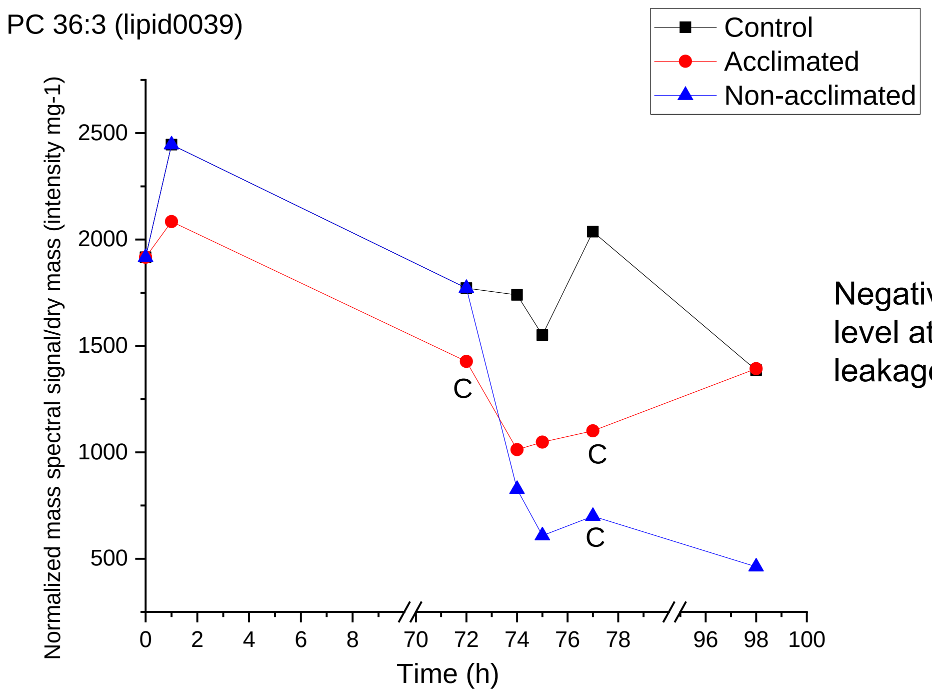

Negative correlation of lipid level at 75 h with final ion leakage

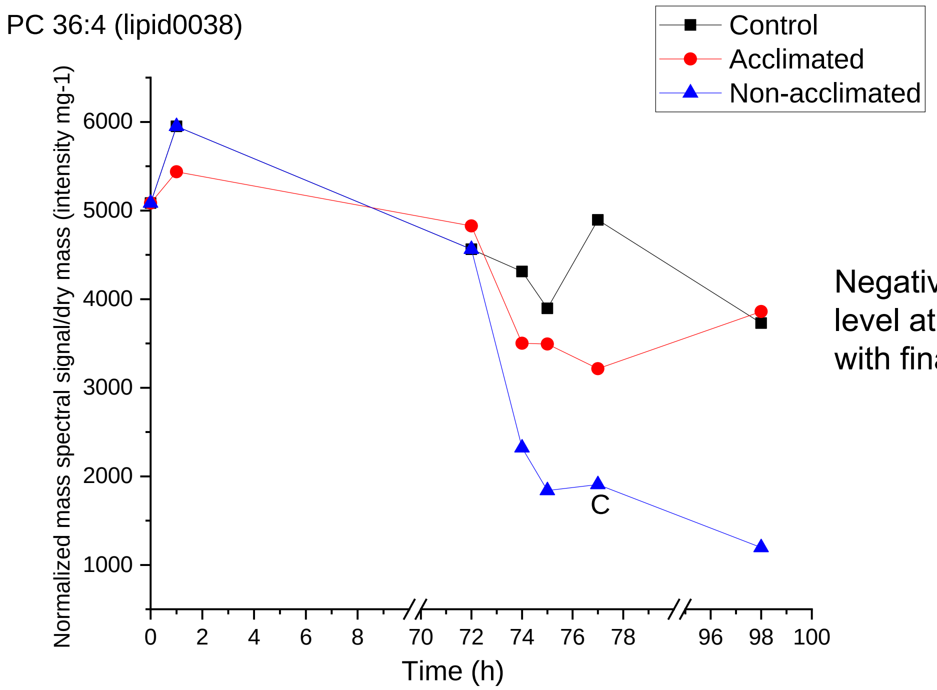

Negative correlation of lipid level at 74, 75, and 77 h with final ion leakage

PC 36:5 (lipid0037)

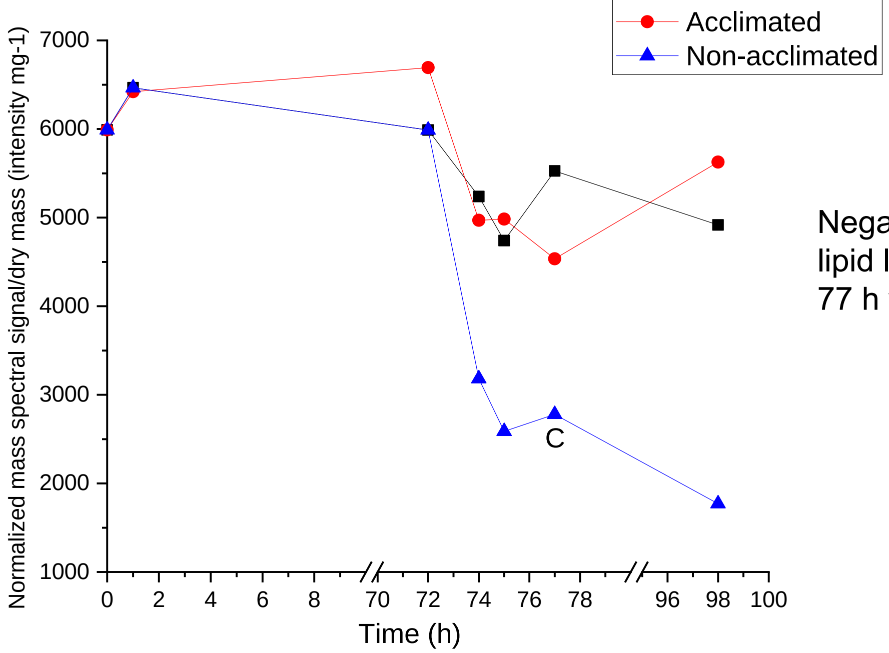

Negative correlation of lipid level at 74, 75, and 77 h with final ion leakage

PC 38:2 (lipid0046)

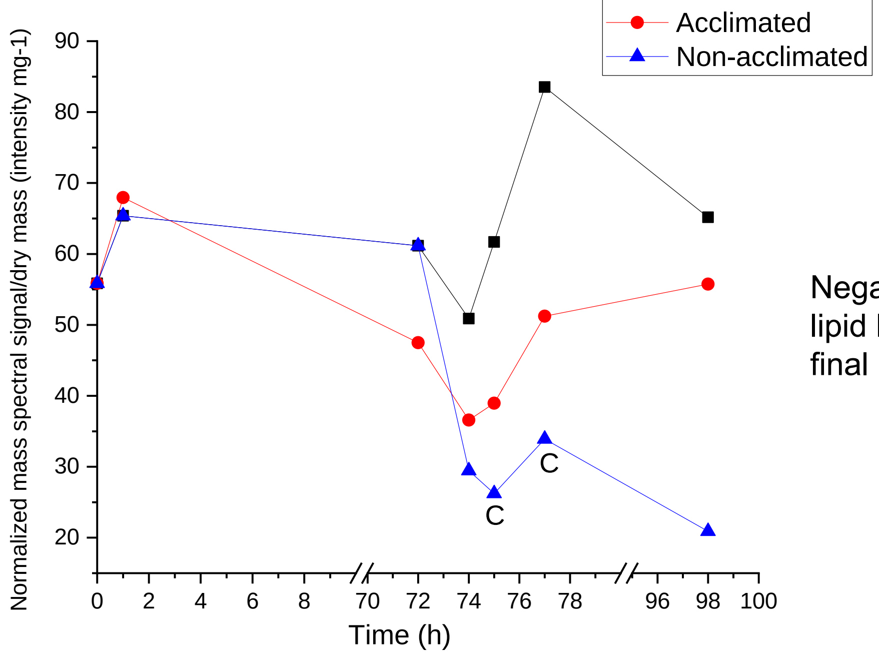

Negative correlation of lipid level at 75 h with final ion leakage

PC 38:3 (lipid0045)

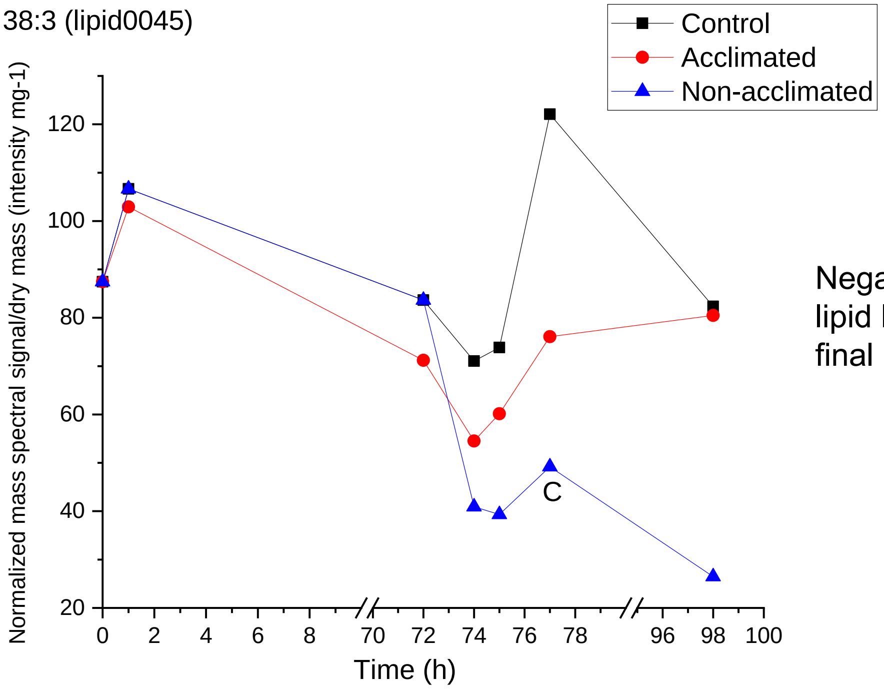

Negative correlation of lipid level at 75 h with final ion leakage

PC 38:4 (lipid0044)

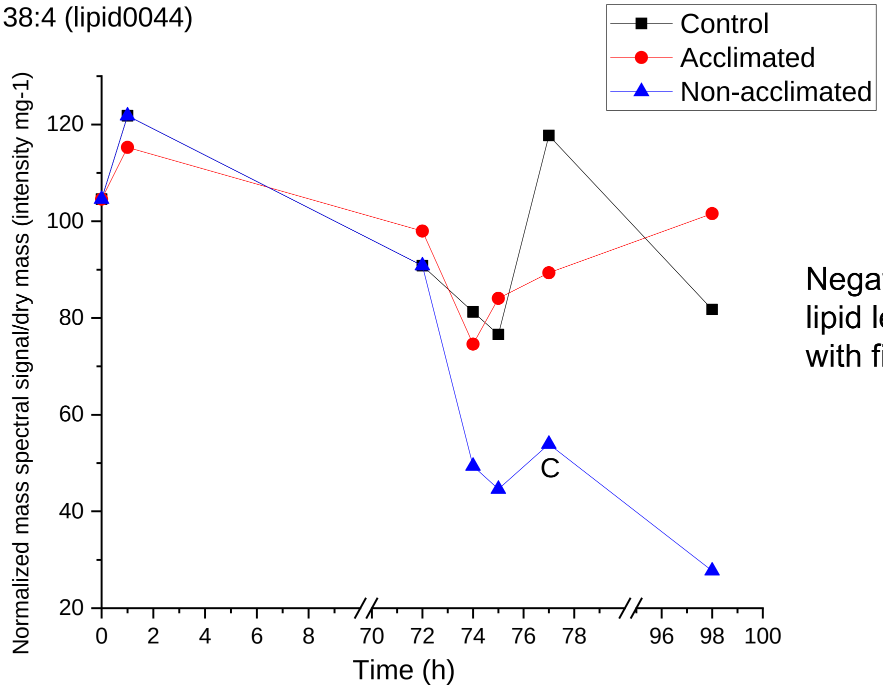

Negative correlation of lipid level at 74 and 75 h with final ion leakage

PC 38:5 (lipid0043)

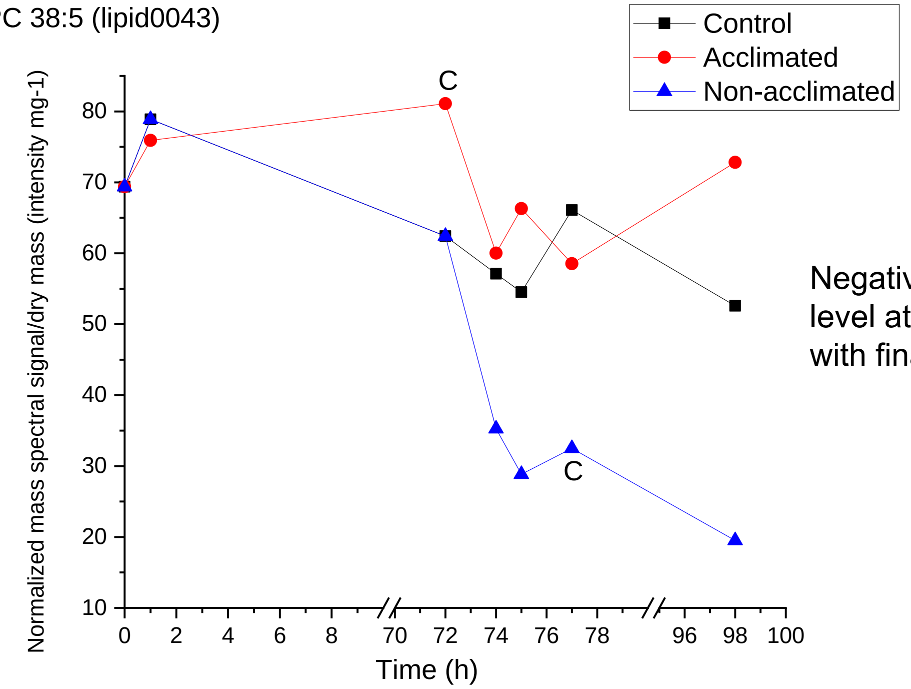

Negative correlation of lipid level at 74, 75, and 77 h with final ion leakage

PC 38:6 (lipid0042)

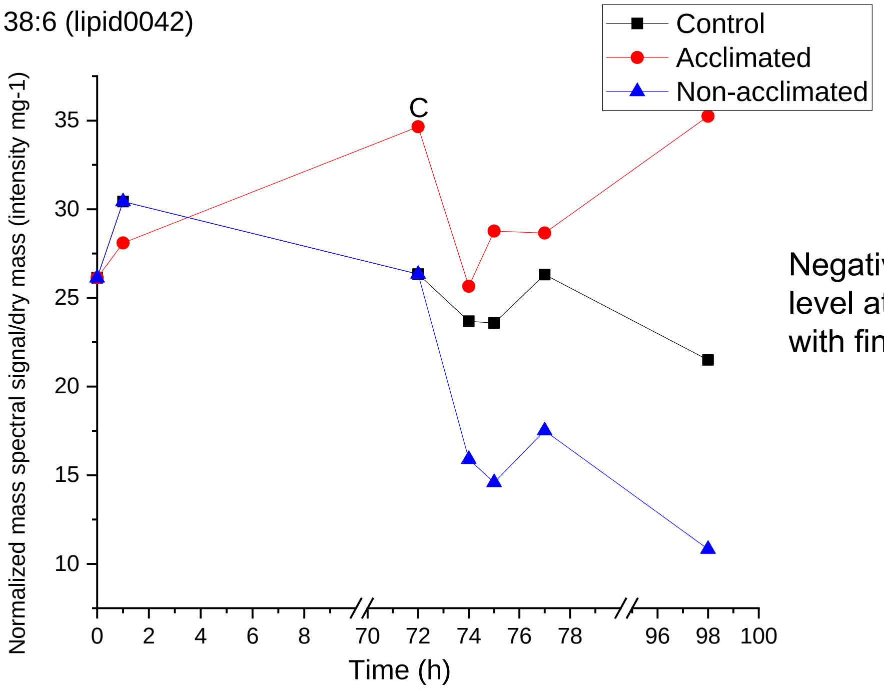

Negative correlation of lipid level at 74, 75, and 77 h with final ion leakage

PC 40:2* (lipid0050)

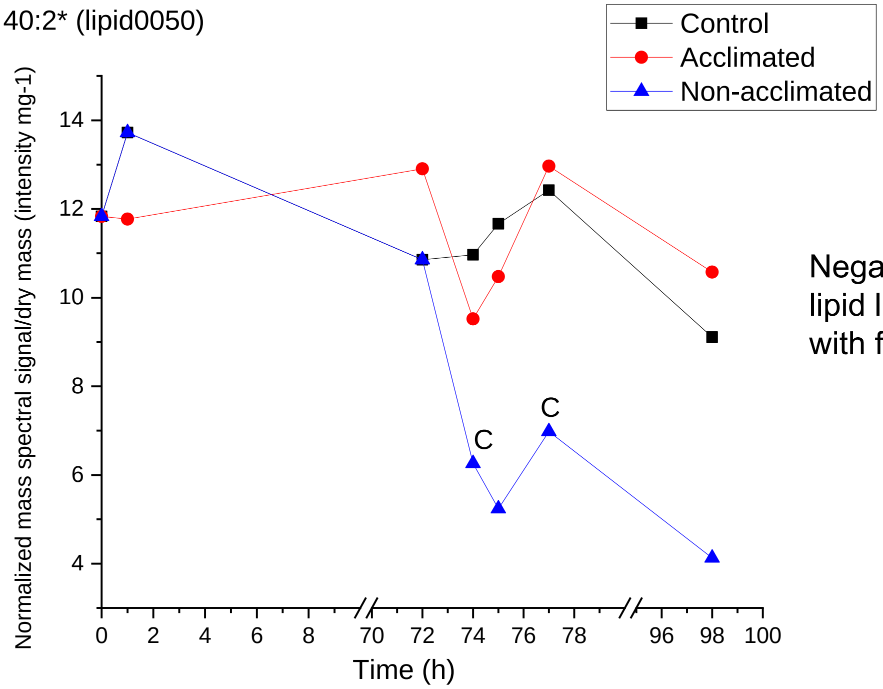

Negative correlation of lipid level at 75 and 77 h with final ion leakage

PC 40:3* (lipid0049)

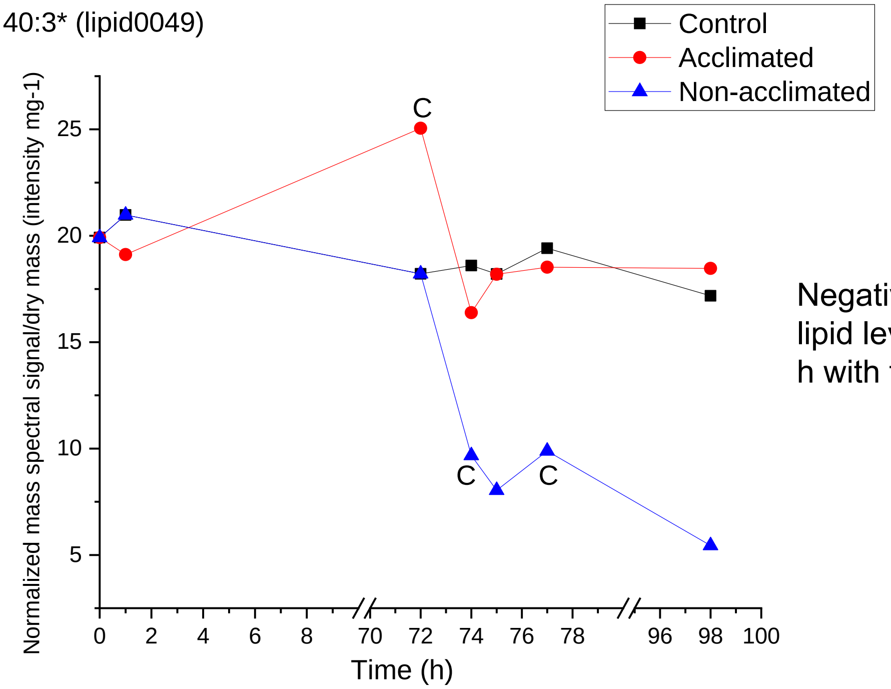

Negative correlation of lipid level at 74, 75, and 77 h with final ion leakage

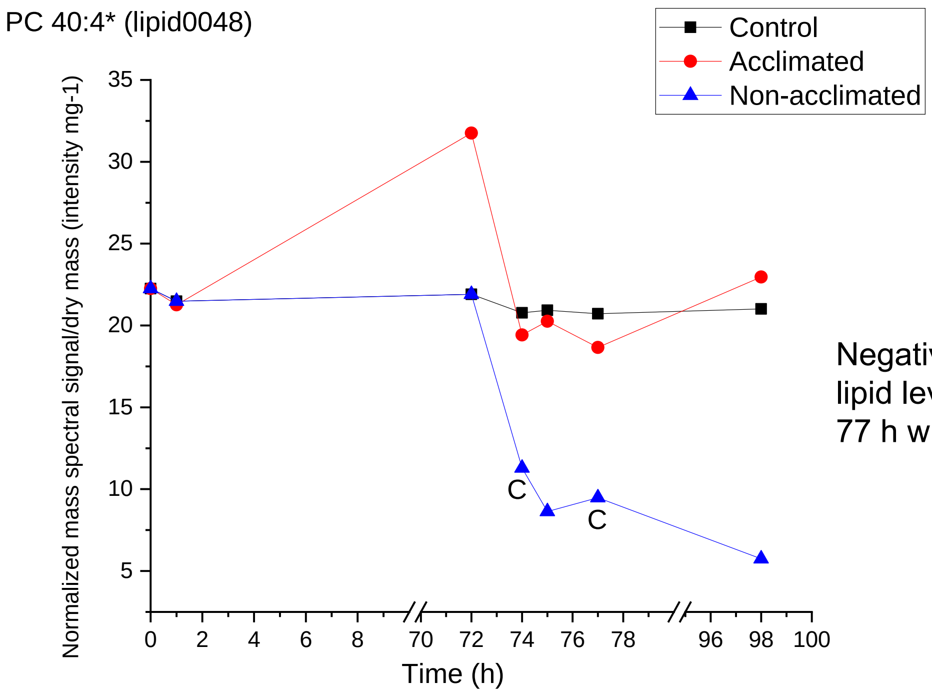

Negative correlation of lipid level at 74, 75, and 77 h with final ion leakage

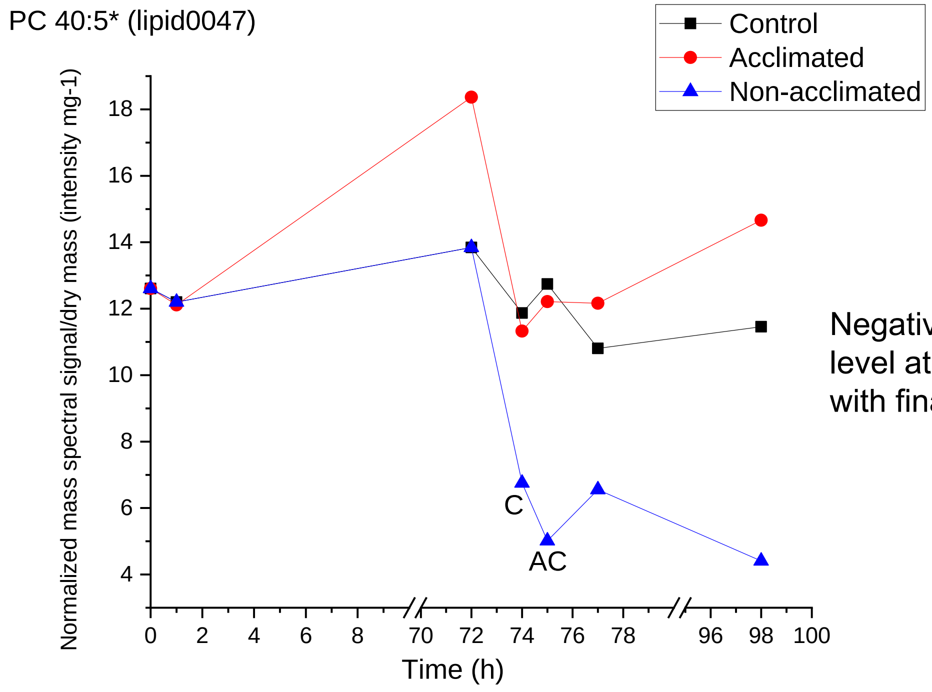

Negative correlation of lipid level at 74, 75, and 77 h with final ion leakage

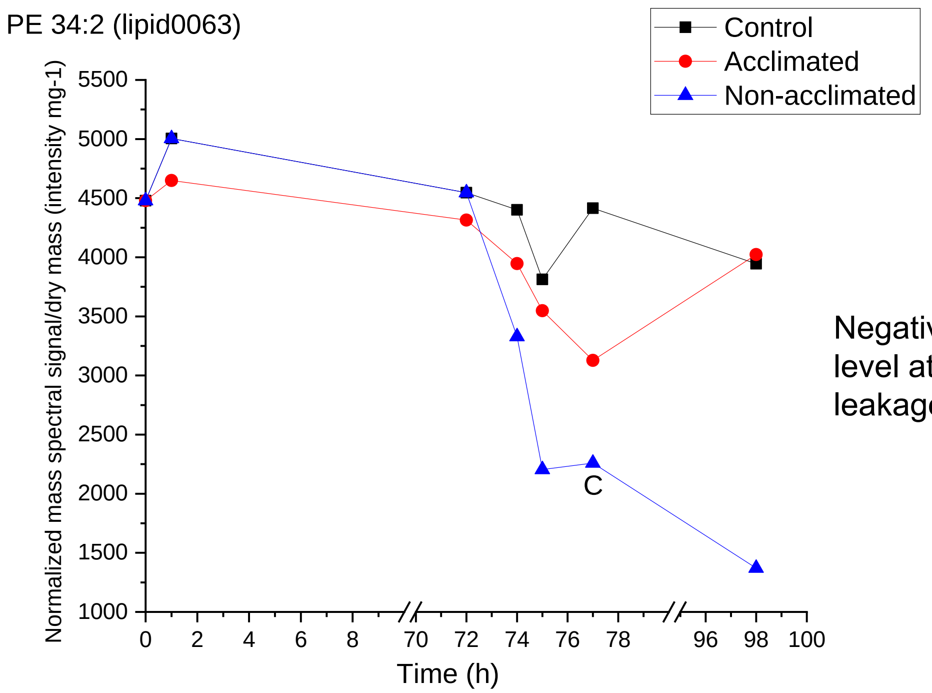

Negative correlation of lipid level at 75 h with final ion leakage

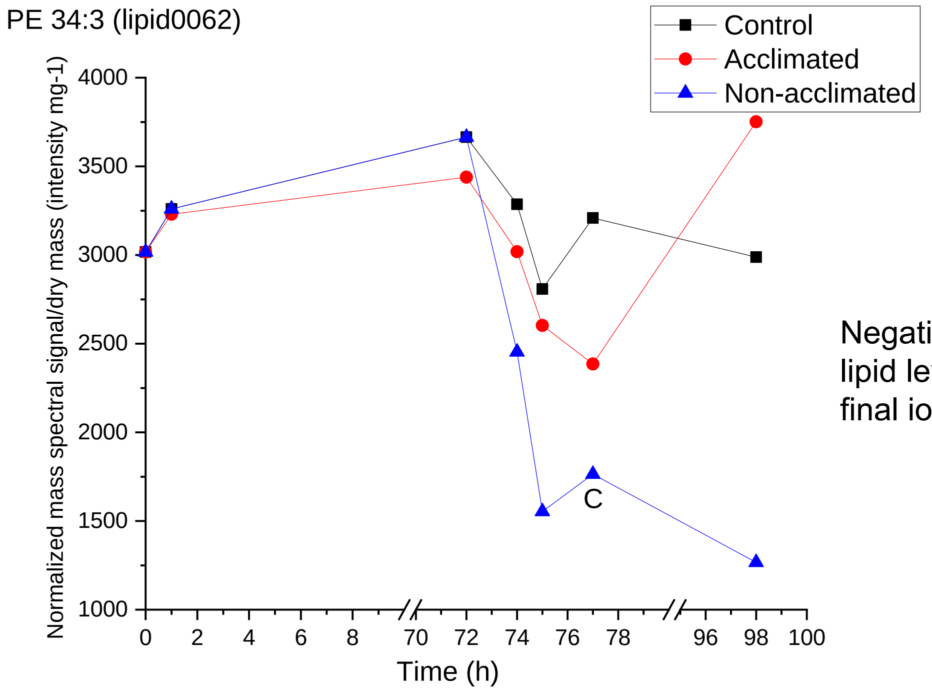

Negative correlation of lipid level at 75 h with final ion leakage

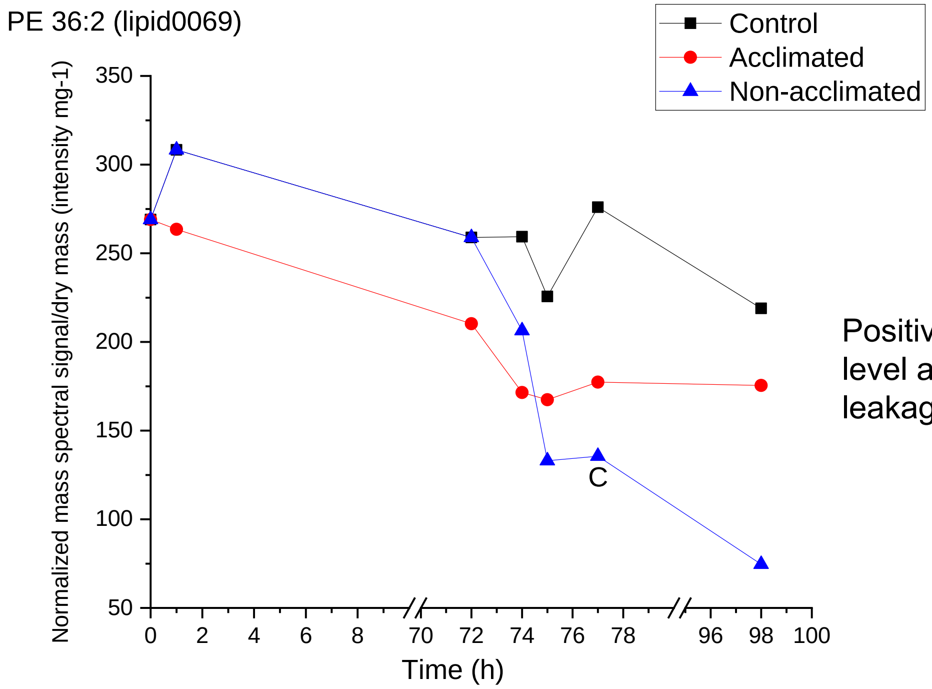

Positive correlation of lipid level at 74 h with final ion leakage

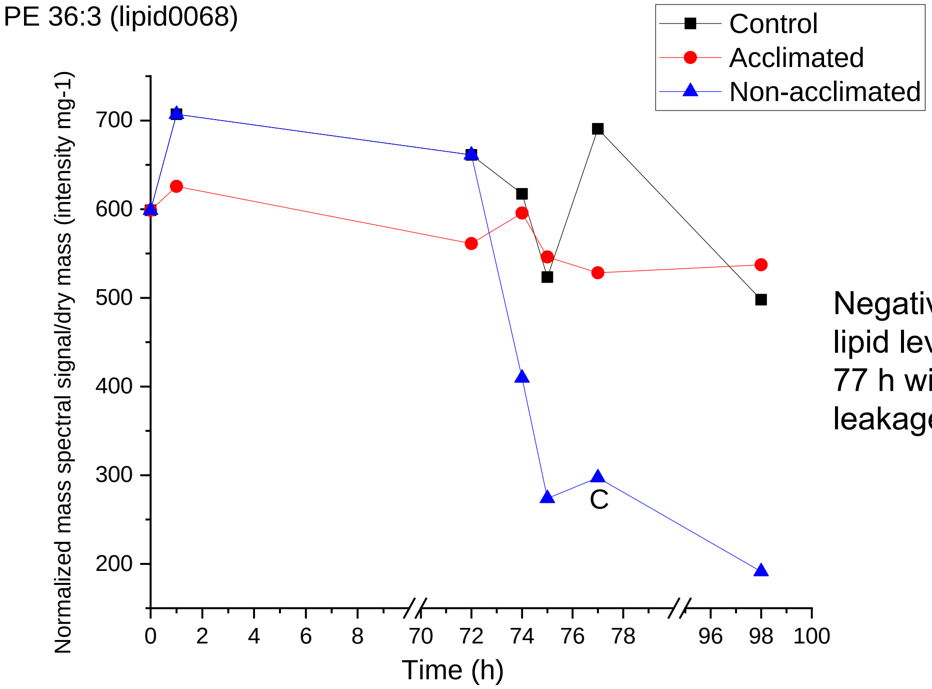

Negative correlation of lipid level at 74, 75, and 77 h with final ion leakage

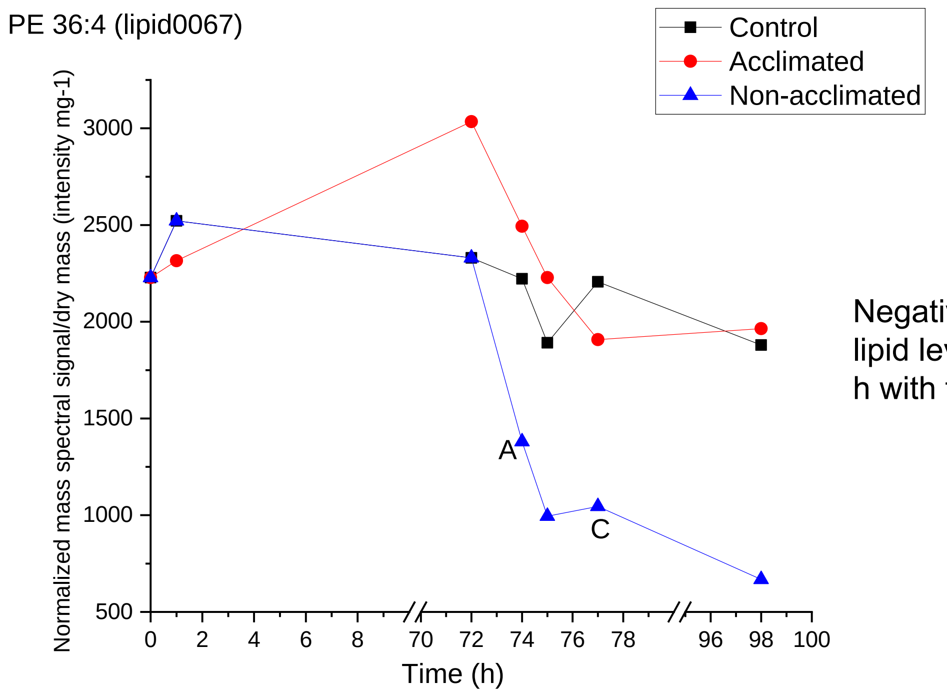

Negative correlation of lipid level at 74, 75, and 77 h with final ion leakage

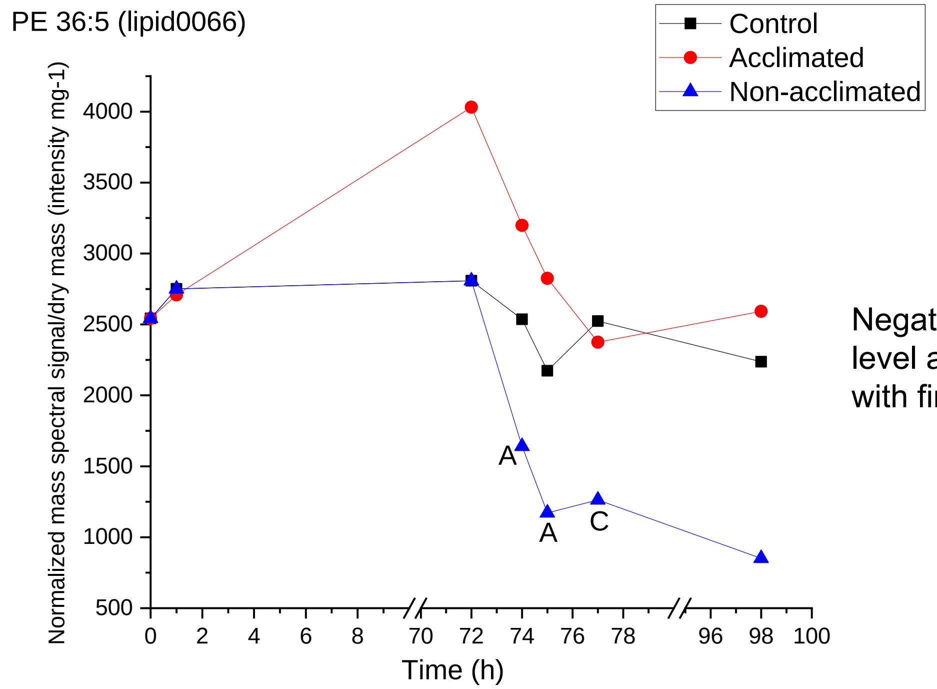

Negative correlation of lipid level at 74, 75, and 77 h with final ion leakage

PE 36:6 (lipid0065)

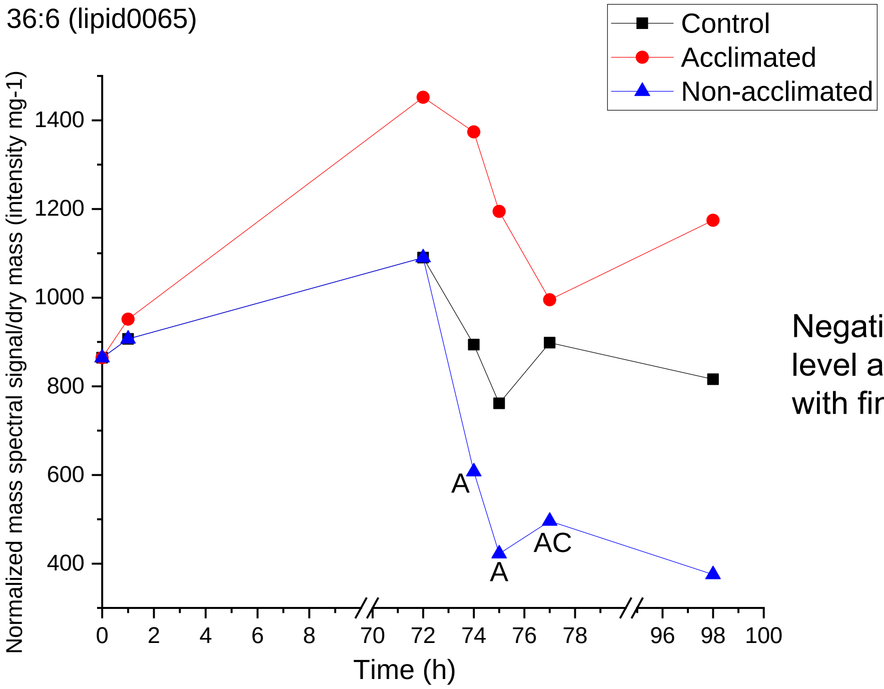

Negative correlation of lipid level at 74, 75, and 77 h with final ion leakage

PE 38:4 (lipid0073)

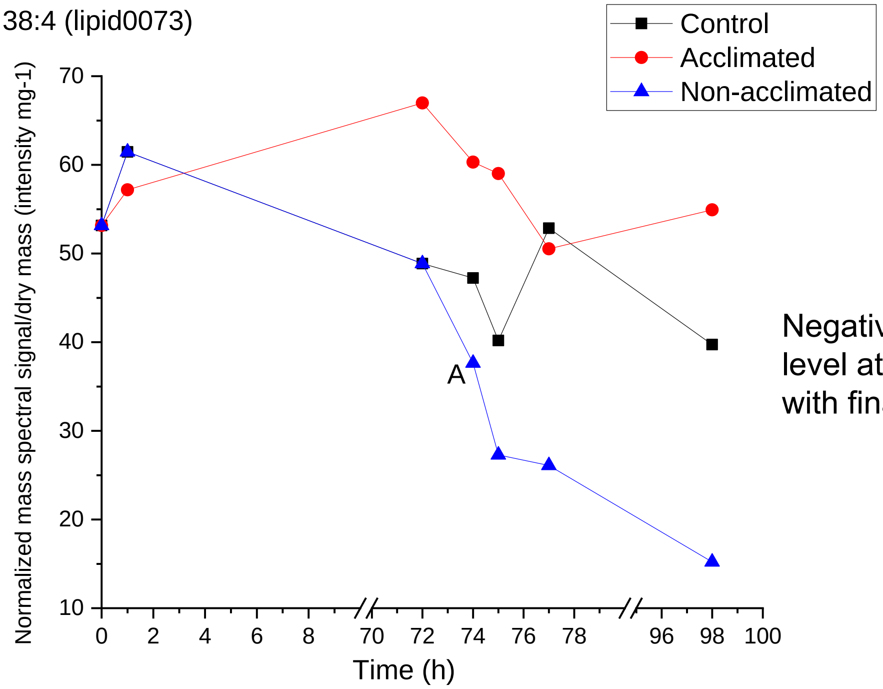

Negative correlation of lipid level at 74, 75, and 77 h with final ion leakage

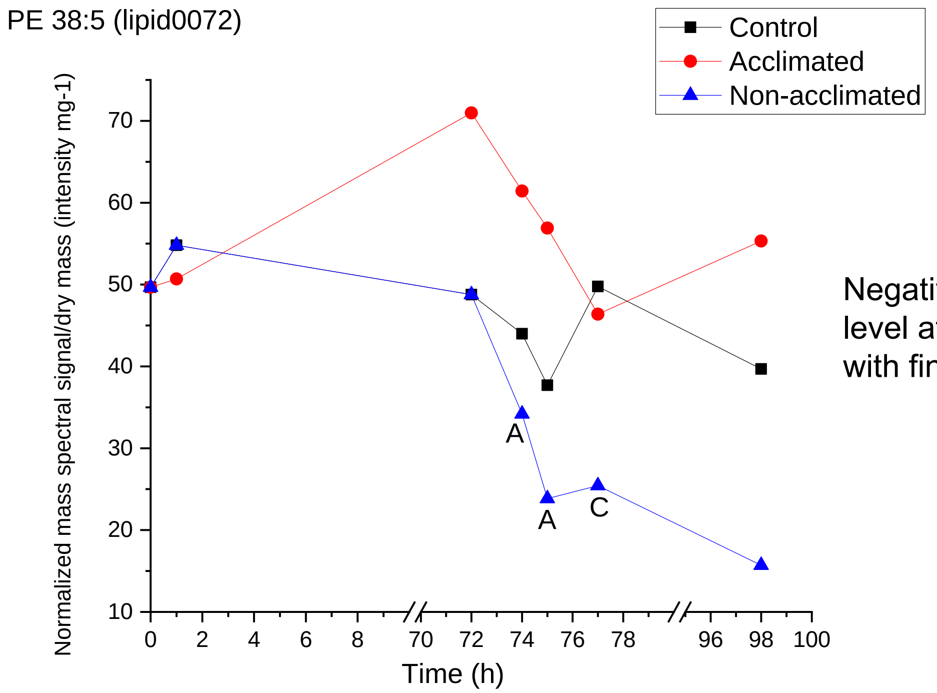

Negative correlation of lipid level at 74, 75, and 77 h with final ion leakage

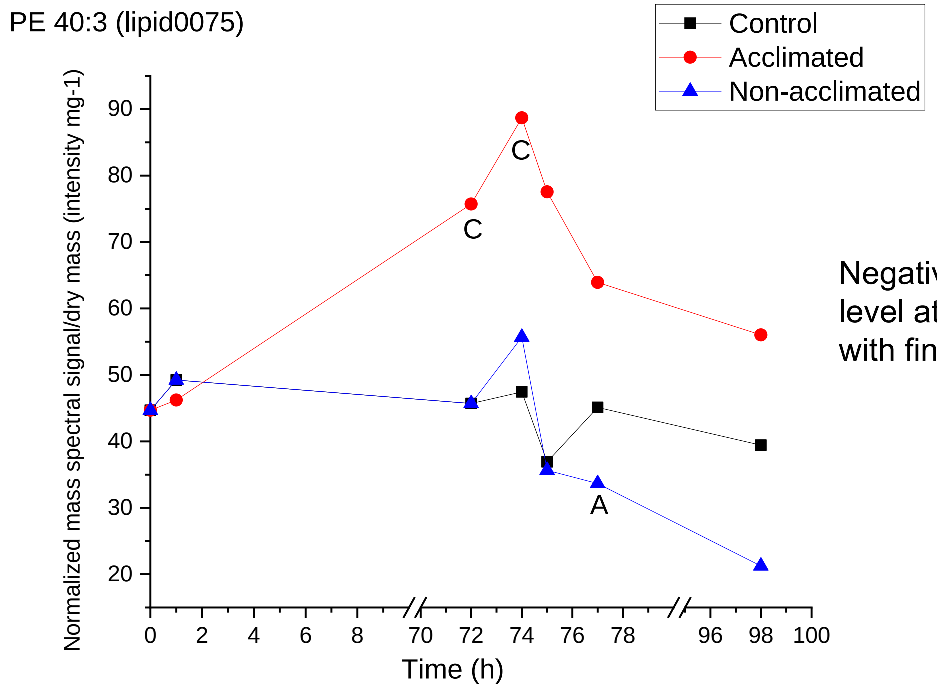

Negative correlation of lipid level at 74, 75, and 77 h with final ion leakage

PE 42:2 (lipid0079)

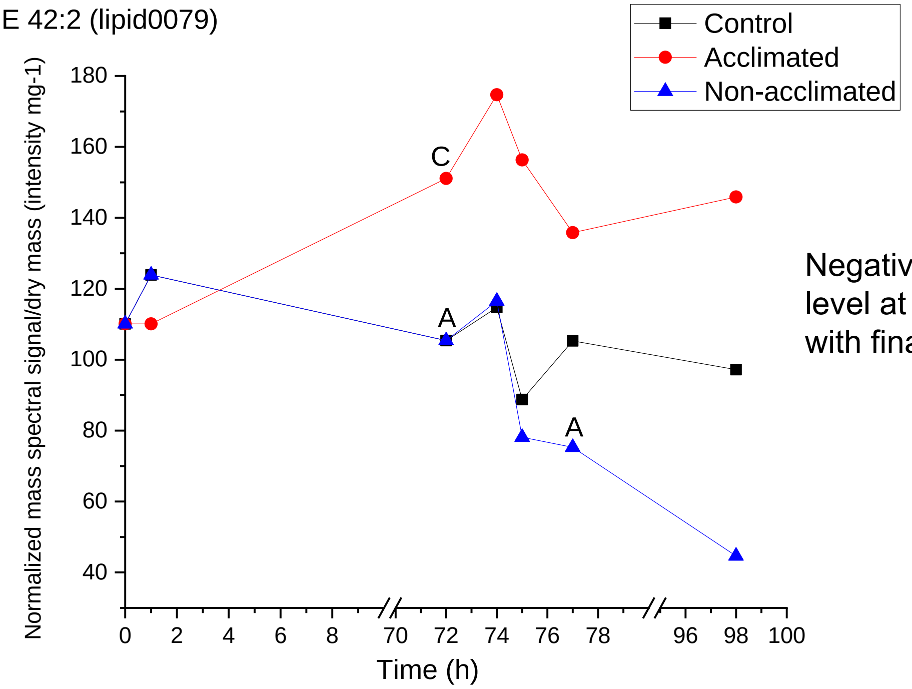

Negative correlation of lipid level at 74, 75, and 77 h with final ion leakage

PE 42:3 (measured in positive mode) (lipid0078)

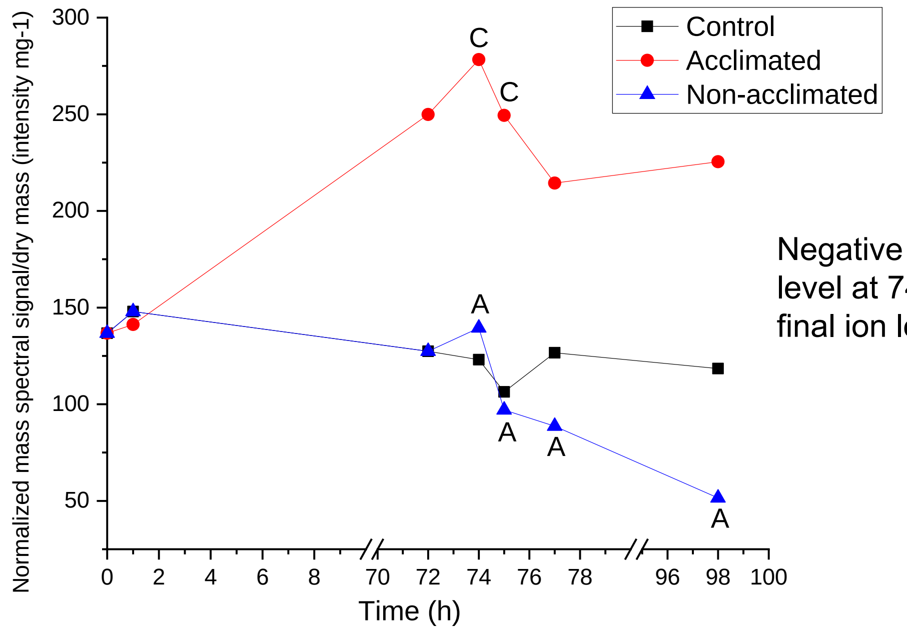

Negative correlation of lipid level at 74, 75, and 77 h with final ion leakage

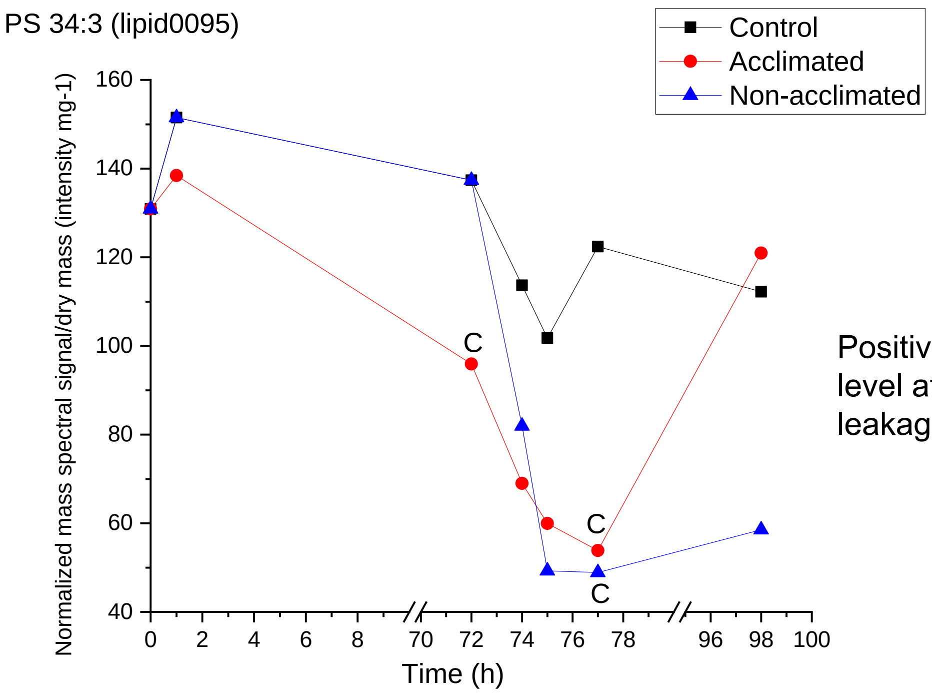

Positive correlation of lipid level at 74 h with final ion leakage

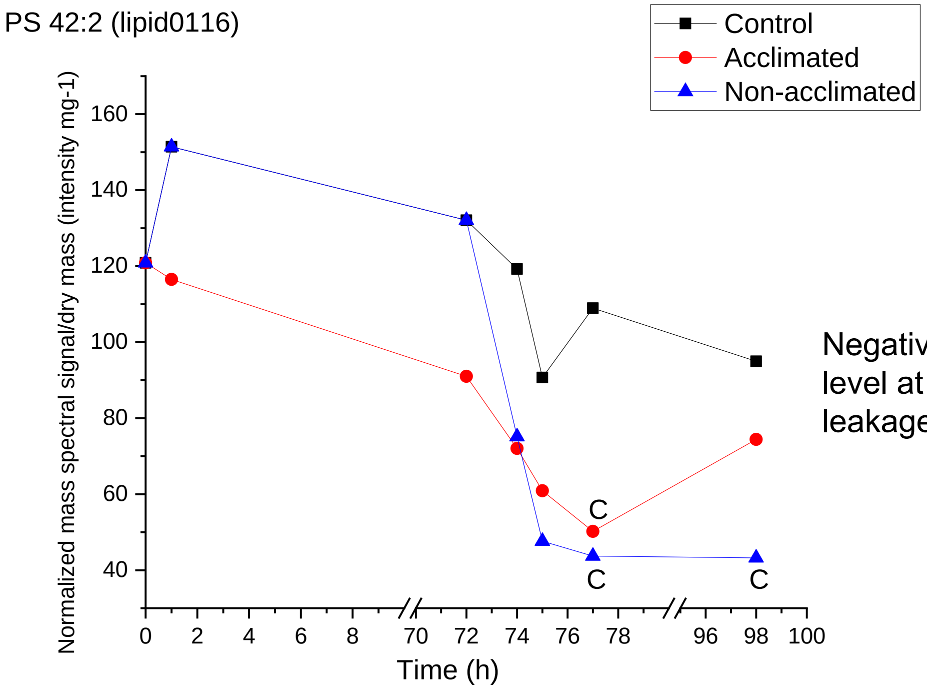

Negative correlation of lipid level at 75 h with final ion leakage

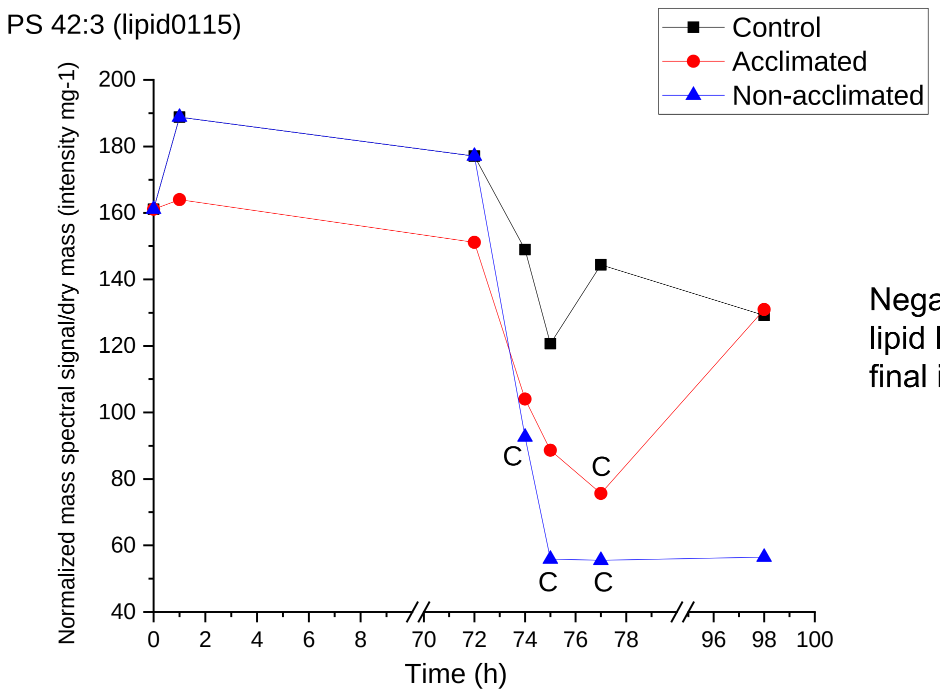

Negative correlation of lipid level at 75 h with final ion leakage

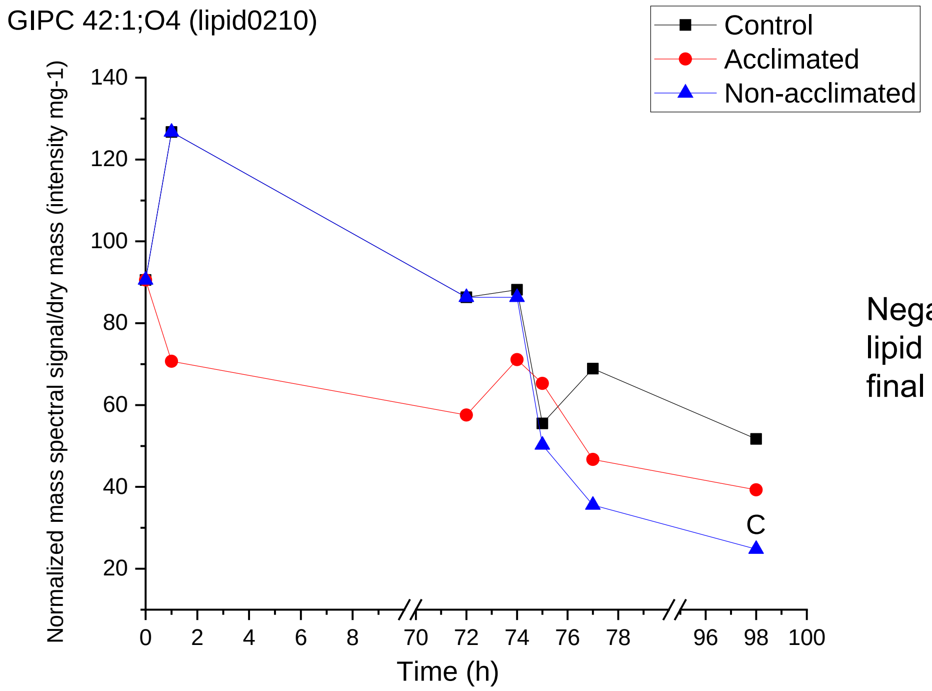

Negative correlation of lipid level at 75 h with final ion leakage

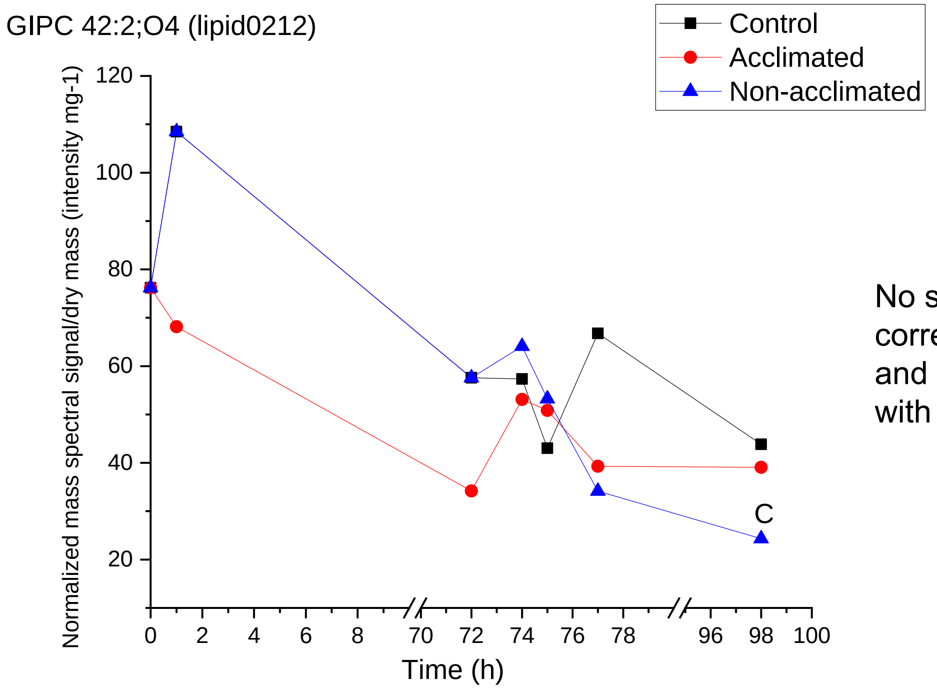

No significant correlation of 74, 75, and 77 h lipid levels with final ion leakage

Figure S3. Time courses of levels of polygalactosylated lipids in rosettes of control, non-acclimated, and acclimated plants. Treatments are shown in Figure 1. "C" indicates that the lipid level in non-acclimated or acclimated plants is significantly different than the control level, and "A" indicates that the lipid level in non-acclimated plants is significantly different than the level in acclimated plants (Table S2). Indicated on each plot is whether there is significant correlation of lipid level at the 74-, 75-, and 77-h time points with final (98 h) ion leakage.

**Lipid number**	**Panel**	**Class, oxidation**	**Lipid name**
lipid0305	3A	TeGDG, non-oxidized	TeGDG 34:6
lipid0309	3A	TeGDG, non-oxidized	TeGDG 36:6
lipid0294	3B	TrGDG, non-oxidized	TrGDG 34:6
lipid0298	3B	TrGDG, non-oxidized	TrGDG 36:6

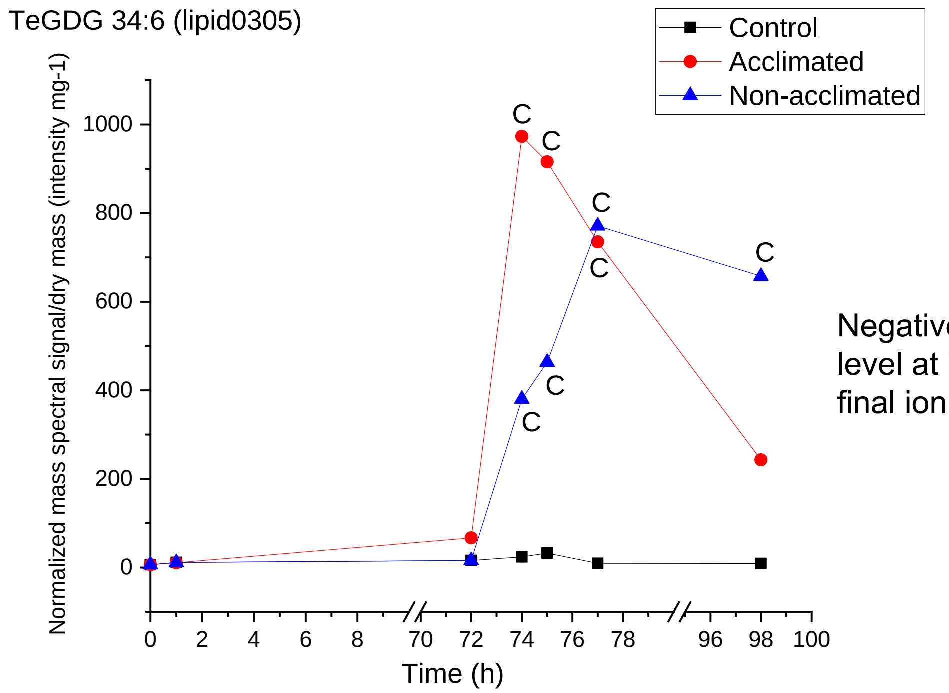

Negative correlation of lipid level at 74 and 75 h with final ion leakage

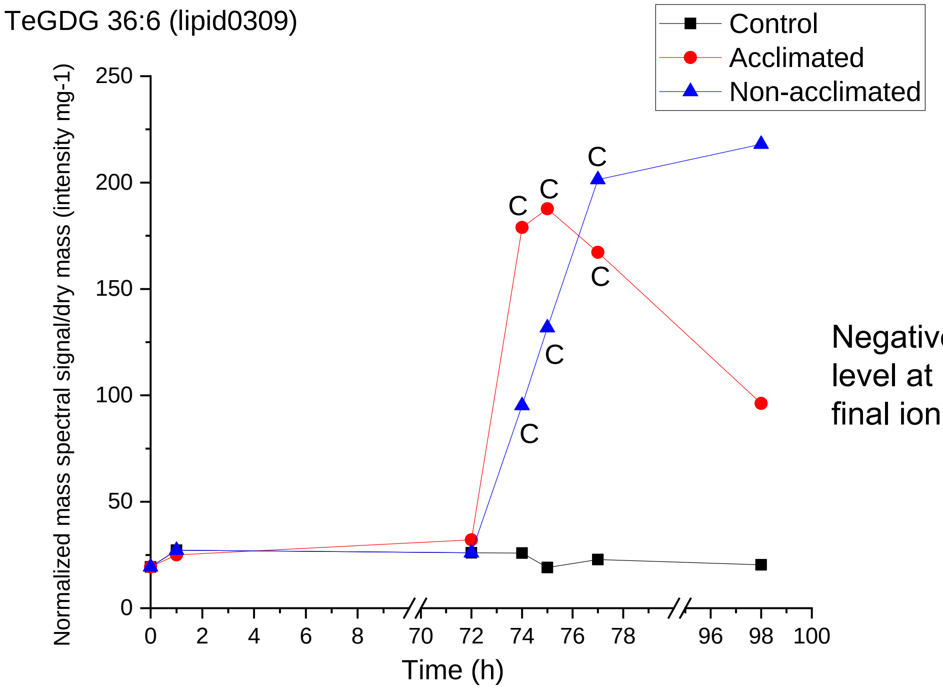

Negative correlation of lipid level at 74 and 75 h with final ion leakage

3A

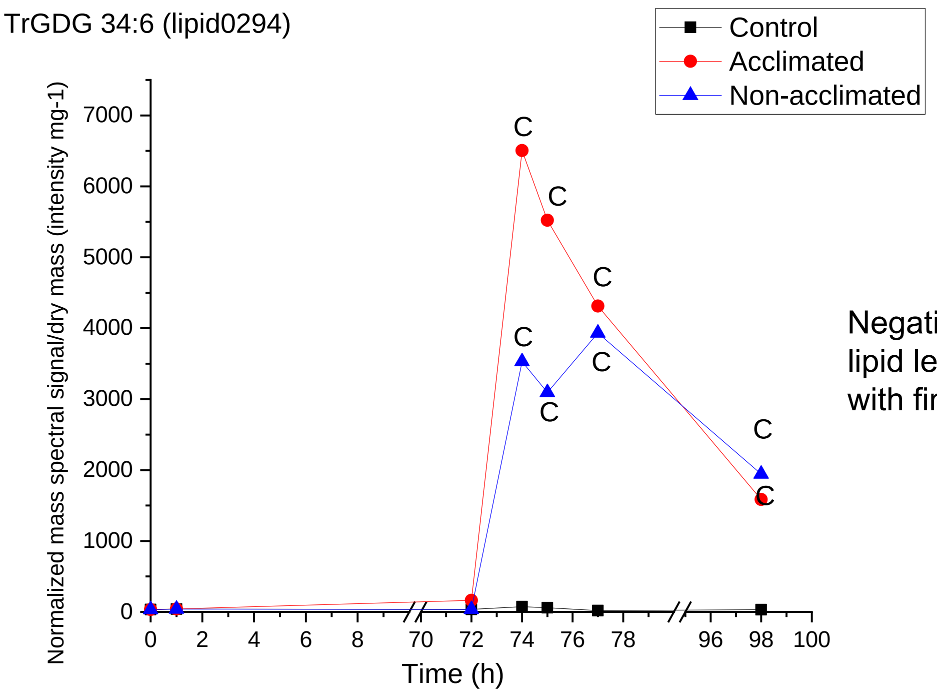

Negative correlation of lipid level at 74 and 75 h with final ion leakage

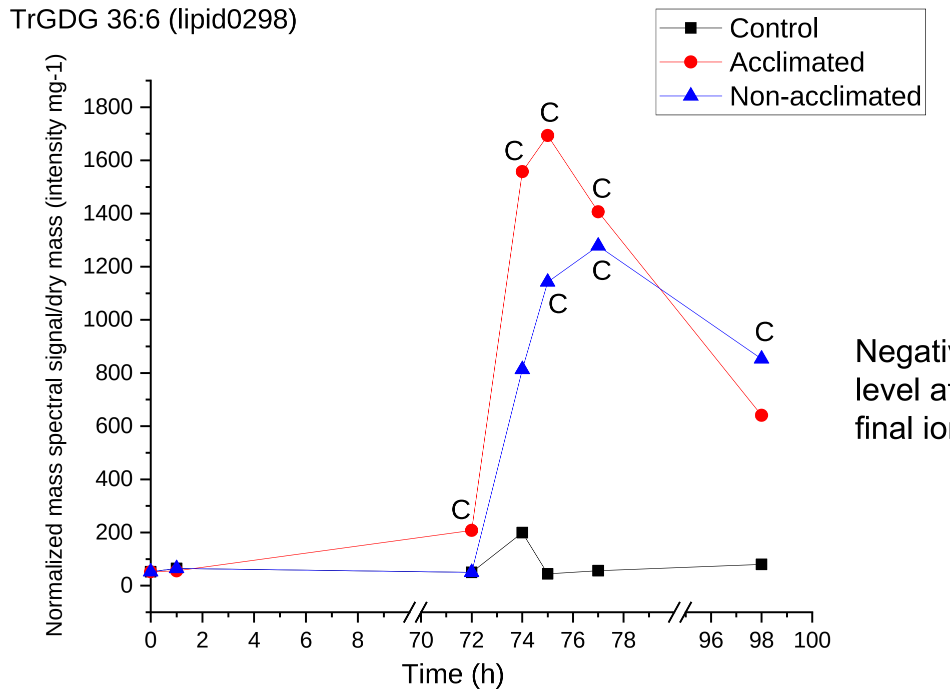

Negative correlation of lipid level at 74 and 75 h with final ion leakage

Figure S4. Time courses of levels of selected phosphatidic acids in rosettes of control, non-acclimated, and acclimated plants. Treatments are shown in Figure 1. Asterisks indicate lipids with quality control (pooled sample) levels less than 0.75 (*) or 0.25 (**) units of normalized mass spectral intensity, where 1 = intensity of 1 pmol of internal standard. "C" indicates that the lipid level in non-acclimated or acclimated plants is significantly different than the control level, and "A" indicates that the lipid level in non-acclimated plants is significantly different than the level in acclimated plants (Table S2). Indicated on each plot is whether there is significant correlation of lipid level at the 74-, 75-, and 77-h time points with final (98 h) ion leakage.

Lipid number	Panel	Class, oxidation	Lipid name
**extraplastidic PA**			
lipid0126	4A	PA, non-oxidized	PA 34:1
lipid0125	4A	PA, non-oxidized	PA 34:2
lipid0124	4B	PA, non-oxidized	PA 34:3
lipid0123	4B	PA, non-oxidized	PA 34:4
lipid0131	4C	PA, non-oxidized	PA 36:2
lipid0130	4C	PA, non-oxidized	PA 36:3
lipid0129	4D	PA, non-oxidized	PA 36:4
lipid0128	4D	PA, non-oxidized	PA 36:5
lipid0127	4E	PA, non-oxidized	PA 36:6
**plastidic PA**			
lipid0122	4E	PA, non-oxidized	PA 34:5*
lipid0121	4F	PA, non-oxidized	PA 34:6

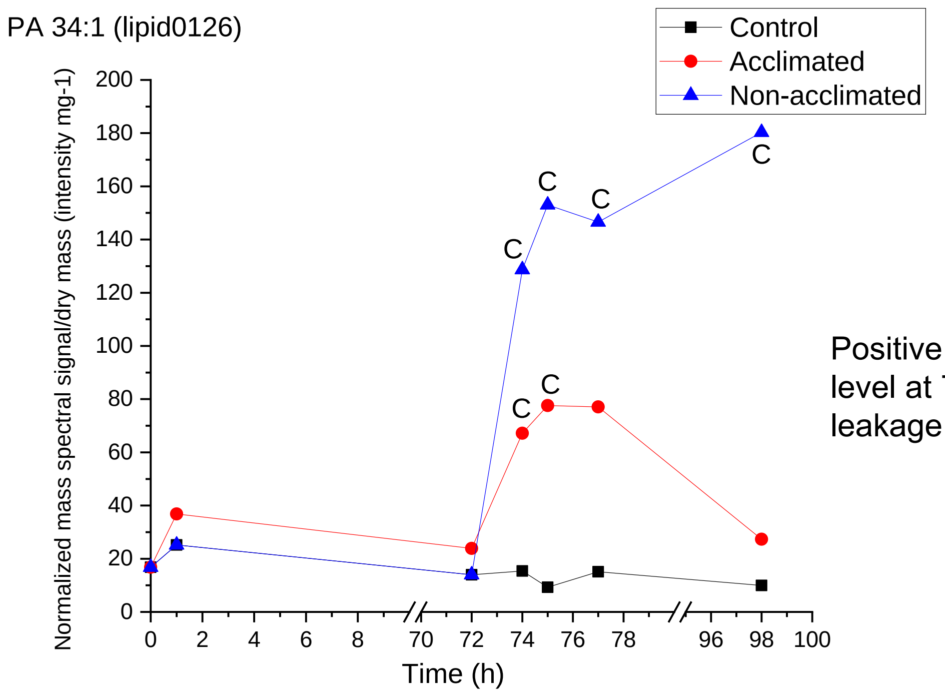

Positive correlation of lipid level at 74 h with final ion leakage

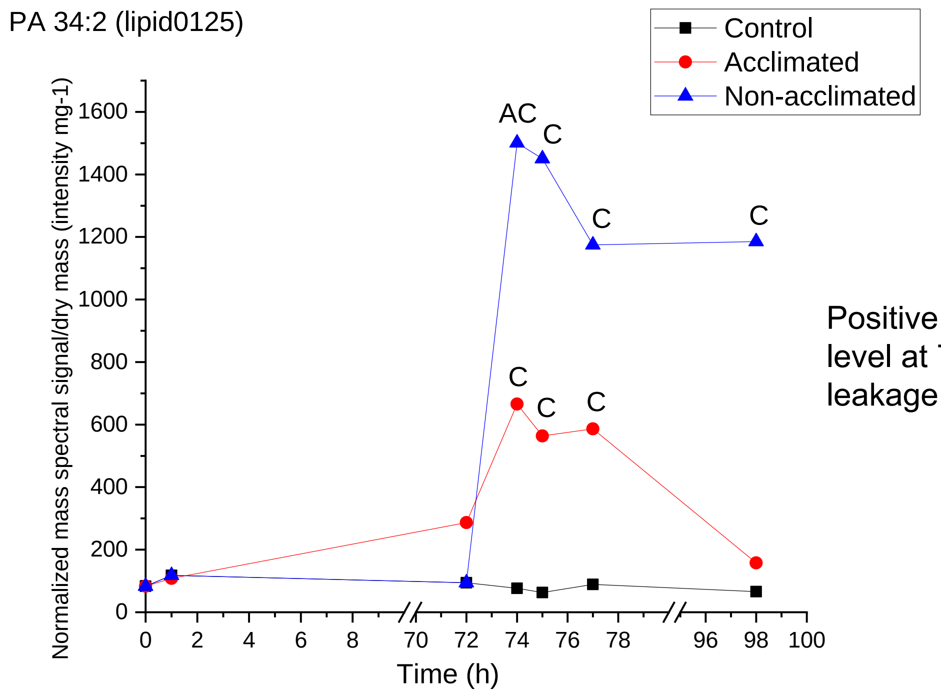

Positive correlation of lipid level at 74 h with final ion leakage

4A

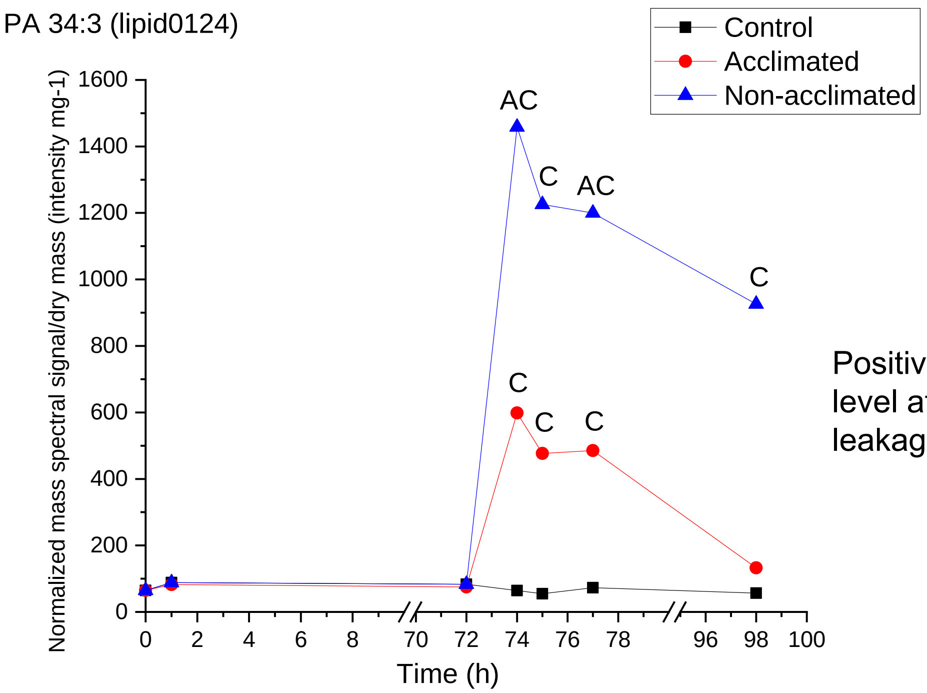

Positive correlation of lipid level at 74 h with final ion leakage

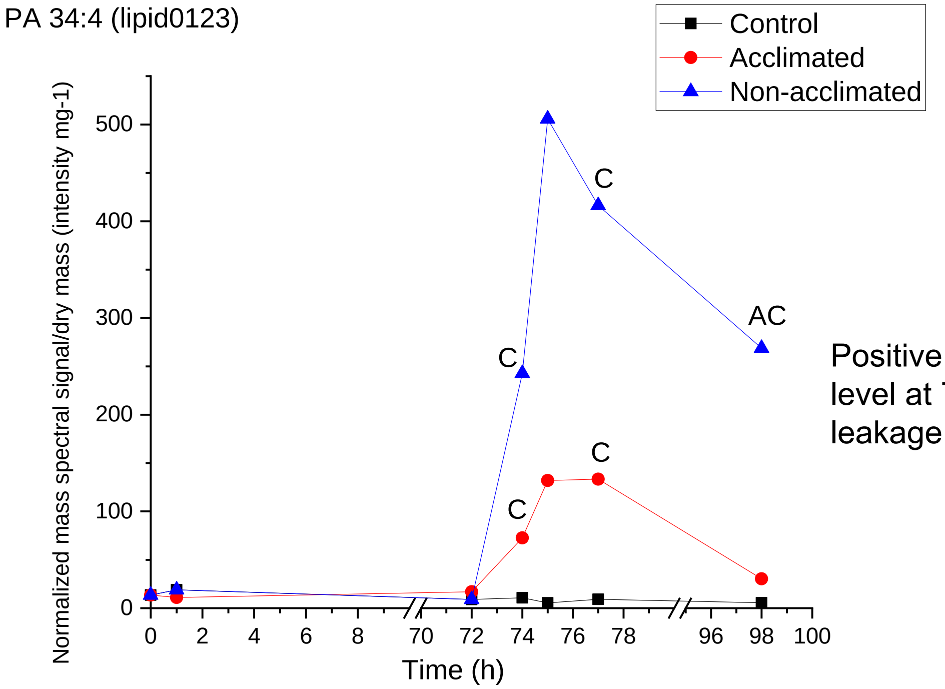

Positive correlation of lipid level at 74 h with final ion leakage

4B

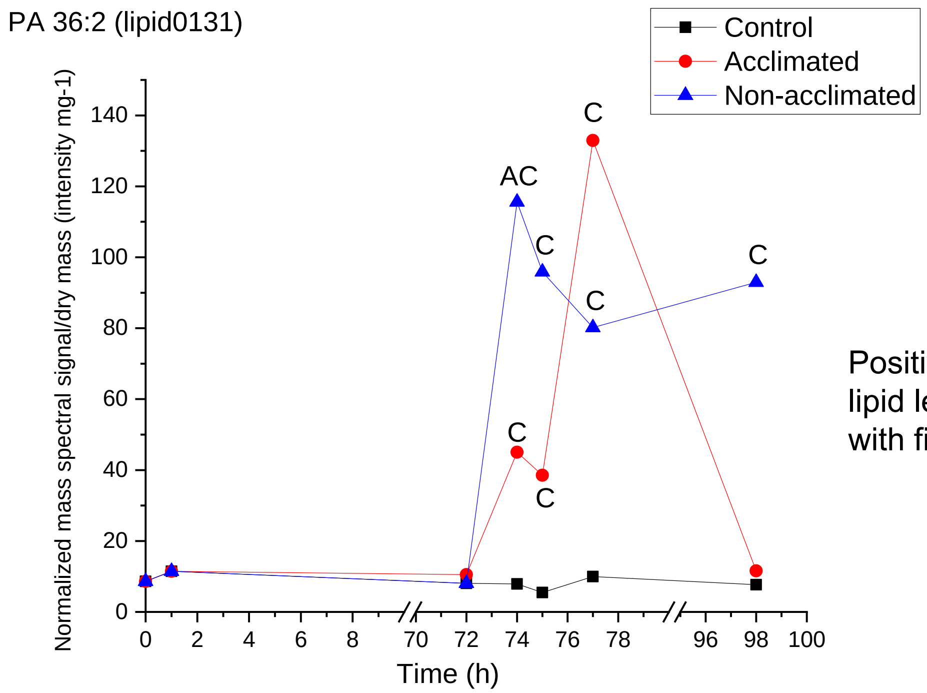

Positive correlation of lipid level at 74 and 75 h with final ion leakage

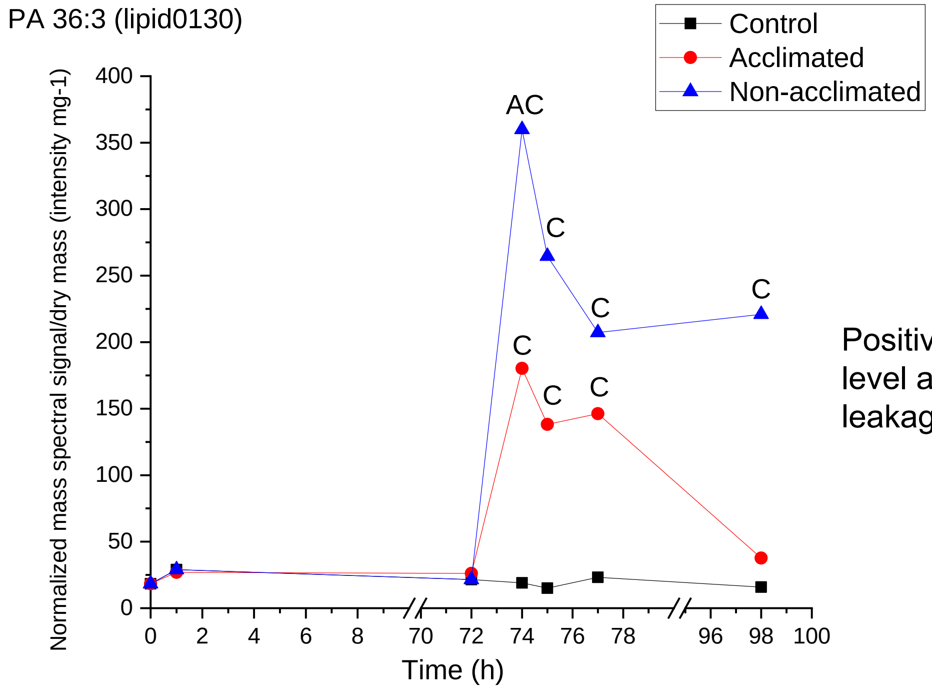

Positive correlation of lipid level at 74 h with final ion leakage

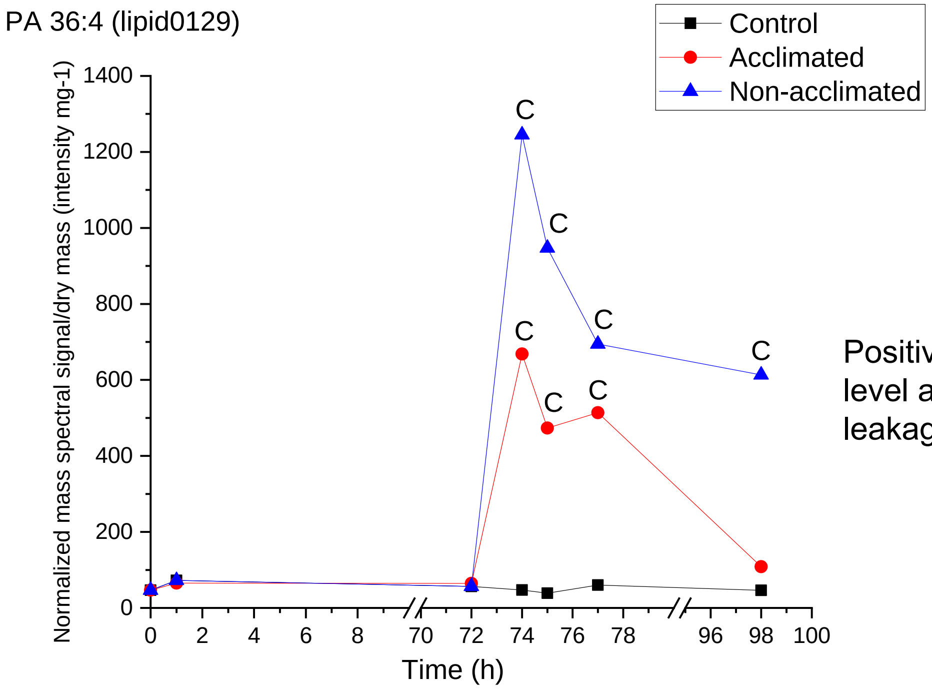

Positive correlation of lipid level at 74 h with final ion leakage

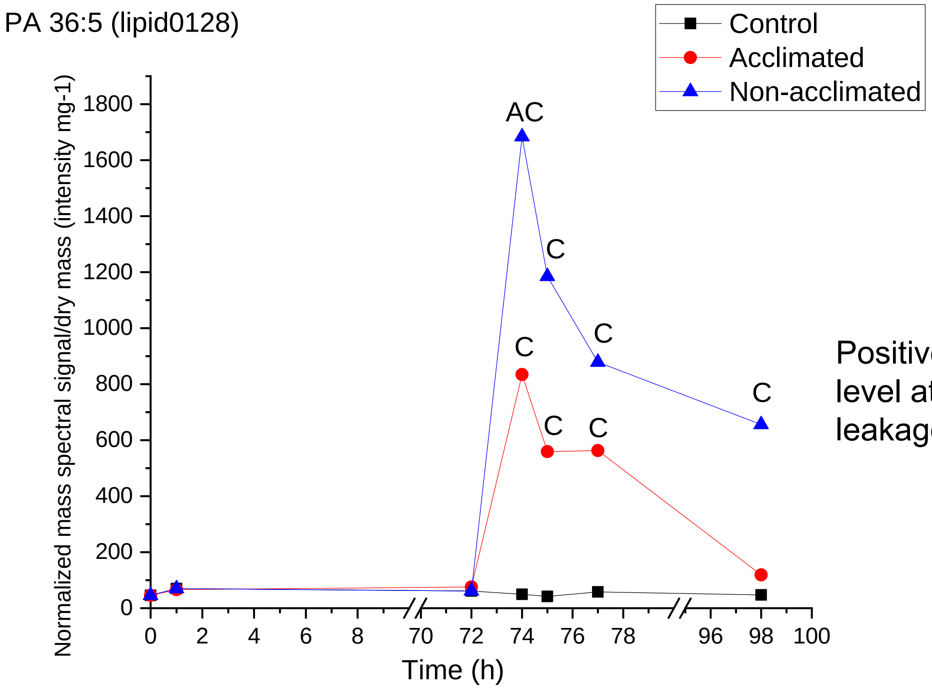

Positive correlation of lipid level at 74 h with final ion leakage

4D

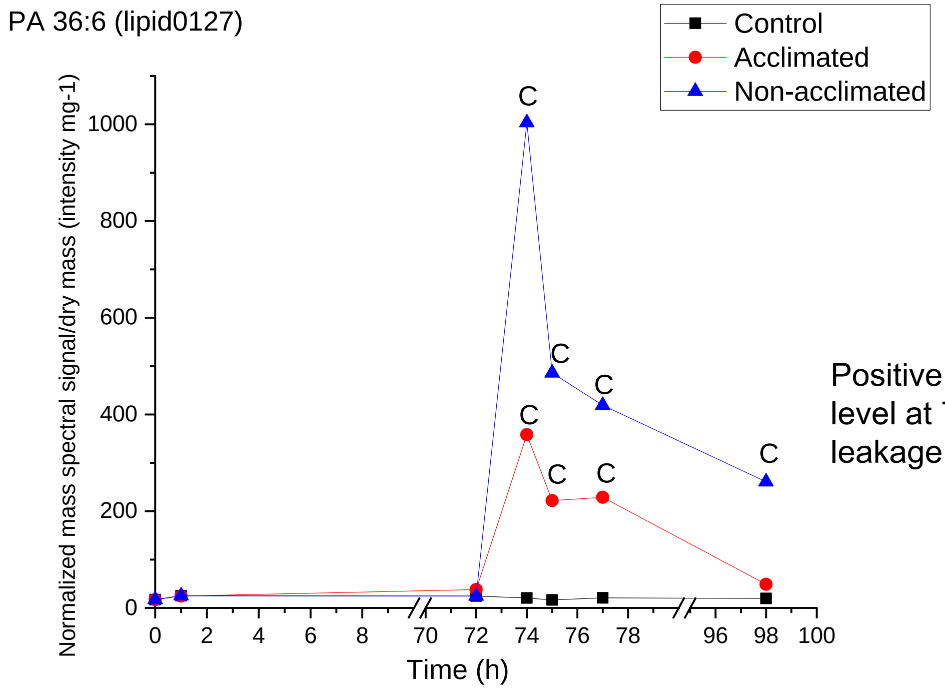

Positive correlation of lipid level at 74 h with final ion leakage

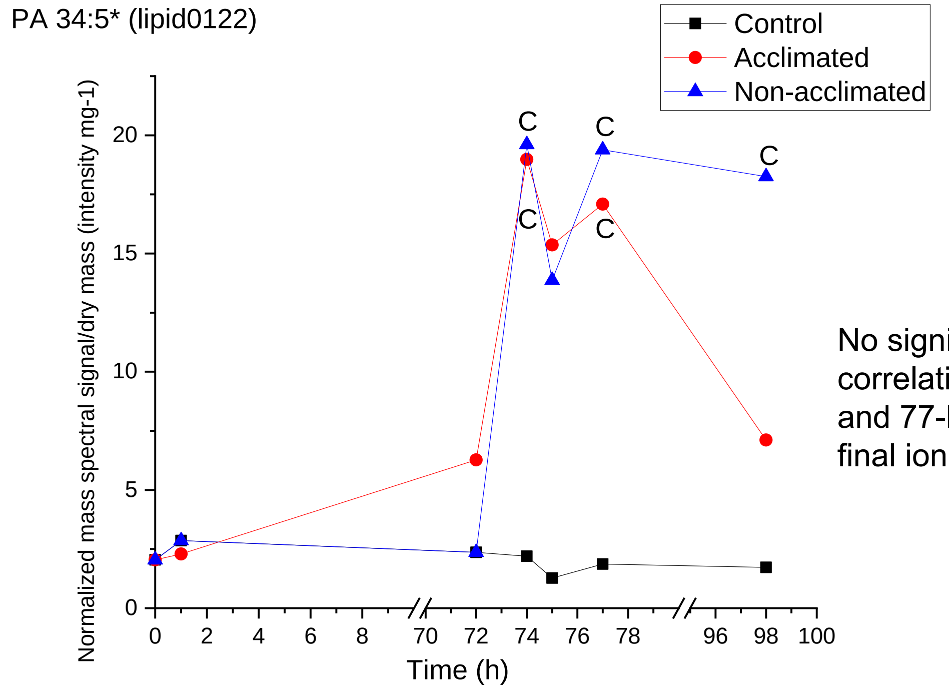

No significant correlation of 74-, 75-, and 77-h lipid levels with final ion leakage

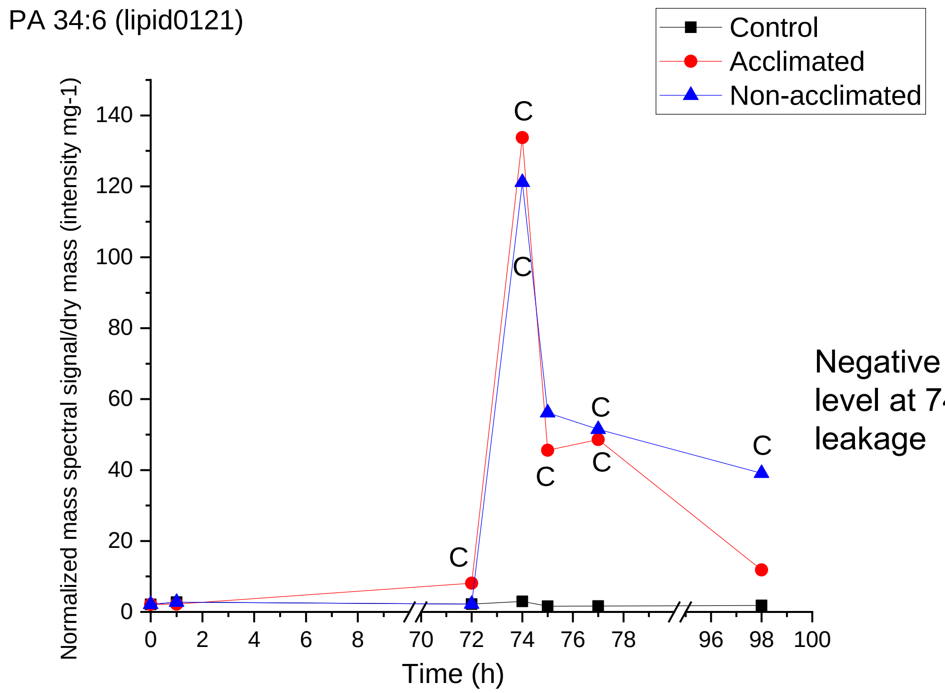

Negative correlation of lipid level at 74 h with final ion leakage

4F

Figure S5. Time courses of levels of selected head group-acylated chloroplast lipids in rosettes of control, non-acclimated, and acclimated plants. Treatments are shown in Figure 1. Asterisks indicate lipids with quality control (pooled sample) levels less than 0.75 (*) or 0.25 (**) units of normalized mass spectral intensity, where 1 = intensity of 1 pmol of internal standard. "C" indicates that the lipid level in non-acclimated or acclimated plants is significantly different than the control level, and "A" indicates that the lipid level in non-acclimated plants is significantly different than the level in acclimated plants (Table S2). Indicated on each plot is whether there is significant correlation of lipid level at the 74-, 75-, and 77-h time points with final (98 h) ion leakage.

Lipid number	Panel	Lipid name (new Lipid Maps nomenclature or similar)	Previous nomenclature
**Non-oxidized, acylated chloroplast lipids**			
lipid0473	5A	MGDG-O(FA 16:0) 34:6	acMGDG(16:0/34:6)
lipid0464	5A	MGDG-O(FA 16:1) 34:6	acMGDG(16:1/34:6)
lipid0459	5B	MGDG-O(FA 16:2) 34:6	acMGDG(16:2/34:6)
lipid0448	5B	MGDG-O(FA 16:3) 34:6	acMGDG(16:3/34:6)
lipid0526	5C	MGDG-O(FA 18:0) 34:6	acMGDG(18:0/34:6)
lipid0514	5C	MGDG-O(FA 18:2) 34:6	acMGDG(18:2/34:6)
lipid0501	5D	MGDG-O(FA 18:3) 34:6	acMGDG(18:3/34:6)
**Oxidized (at leat one acyl chain), acylated chloroplast lipids**			
lipid0601	5E	DGDG-O(FA 18:4;O) 36:6 or DGDG-O(FA 18:4;O) 34:8;O2	acDGDG(18:4-O/36:6) or acDGDG(18:4-O/34:8-2O)
lipid0603	5E	DGDG-O(FA 18:4;O) 36:8;O2	acDGDG(18:4-O/36:8-2O)
lipid0474	5F	MGDG-O(FA 16:0) 34:7;O	acMGDG(16:0/34:7-O)
lipid0483	5F	MGDG-O(FA 16:0) 36:8;O2	acMGDG(16:0/36:8-2O)
lipid0496	5G	MGDG-O(FA 16:3;O) 34:6	acMGDG(16:3-O/34:6)
lipid0498	5G	MGDG-O(FA 16:3;O) 34:8;O2 or MGDG-O(FA 16:3;O) 36:6	acMGDG(16:3-O/34:8-2O) or acMGDG(16:3-O/36:6)
lipid0500	5H	MGDG-O(FA 16:3;O) 36:8;O2	acMGDG(16:3-O/36:8-2O)

lipid0487	5H	MGDG-O(FA 16:4;O) 34:7;O	acMGDG(16:4-O/34:7-O)
lipid0489	5I	MGDG-O(FA 16:4;O) 34:8;O2 or MGDG-O(FA 16:4;O) 36:6	acMGDG(16:4-O/34:8-2O) or acMGDG(16:4-O/36:6)
lipid0495	5I	MGDG-O(FA 16:4;O) 36:8;O2	acMGDG(16:4-O/36:8-2O)
lipid0527	5J	MGDG-O(FA 18:0) 34:7;O	acMGDG(18:0/34:7-O)
lipid0515	5J	MGDG-O(FA 18:2) 34:7;O	acMGDG(18:2/34:7-O)
lipid0518	5K	MGDG-O(FA 18:2) 36:8;O2	acMGDG(18:2/36:8-2O)
lipid0551	5K	MGDG-O(FA 18:2;O) 36:6 or MGDG-O(FA 18:2;O) 34:8;O2	acMGDG(18:2-O/36:6) or acMGDG(18:2-O/34:8-2O)
lipid0596	5L	MGDG-O(FA 18:2;O3) 36:6 or MGDG-O(FA 18:2;O3) 34:8;O2	acMGDG(18:2-3O/36:6) or acMGDG(18:2-3O/34:8-2O)
lipid0553	5L	MGDG-O(FA 18:2;O) 36:8;O2	acMGDG(18:2-O/36:8-2O)
lipid0503	5M	MGDG-O(FA 18:3) 34:7;O	acMGDG(18:3/34:7-O)
lipid0508	5M	MGDG-O(FA 18:3) 36:7;O	acMGDG(18:3/36:7-O)
lipid0512	5N	MGDG-O(FA 18:3) 36:8;O2	acMGDG(18:3/36:8-2O)
lipid0542	5N	MGDG-O(FA 18:3;O) 34:6	acMGDG(18:3-O/34:6)
lipid0543	5O	MGDG-O(FA 18:3;O) 34:7;O	acMGDG(18:3-O/34:7-O)
lipid0544	5O	MGDG-O(FA 18:3;O) 36:6 or MGDG-O(FA 18:3;O) 34:8;O2	acMGDG(18:3-O/36:6) or acMGDG(18:3-O/34:8-2O)
lipid0546	5P	MGDG-O(FA 18:3;O) 36:6;O	acMGDG(18:3-O/36:6-O)
lipid0548	5P	MGDG-O(FA 18:3;O) 36:8;O2	acMGDG(18:3-O/36:8-2O)
lipid0566	5Q	MGDG-O(FA 18:3;O2) 34:6	acMGDG(18:3-2O/34:6)
lipid0570	5Q	MGDG-O(FA 18:3;O2) 36:6 or MGDG-O(FA 18:3;O2) 34:8;O2	acMGDG(18:3-2O/36:6) or acMGDG(18:3-2O/34:8-2O)
lipid0580	5R	MGDG-O(FA 18:3;O2) 36:8;O2	acMGDG(18:3-2O/36:8-2O)
lipid0591	5R	MGDG-O(FA 18:3;O3) 34:6	acMGDG(18:3-3O/34:6)
lipid0592	5S	MGDG-O(FA 18:3;O3) 34:7;O	acMGDG(18:3-3O/34:7-O)

lipid0595	5S	MGDG-O(FA 18:3;O3) 36:8;O2	acMGDG(18:3-3O/36:8-2O)
lipid0532	5T	MGDG-O(FA 18:4;O) 34:6	acMGDG(18:4-O/34:6)
lipid0533	5T	MGDG-O(FA 18:4;O) 34:7;O	acMGDG(18:4-O/34:7-O)
lipid0535	5U	MGDG-O(FA 18:4;O) 34:8;O2 (Arabidopside E or MGDG-O(FA 18:4;O)	acMGDG(18:4-O/34:8-2O) (Arabidopside E) or acMGDG(18:4-
lipid0538	5U	MGDG-O(FA 18:4;O) 36:6;O or MGDG-O(FA 18:4;O) 34:8;O3	acMGDG(18:4-O/36:6-O) or acMGDG(18:4-O/34:8-3O)
lipid0575	5V	MGDG-O(FA 18:4;O) 36:7;O2 (alternative fragmentation)	acMGDG(18:4-O/36:7-2O) (alternative fragmentation)
lipid0537	5V	MGDG-O(FA 18:4;O) 36:7;O	acMGDG(18:4-O/36:7-O)
lipid0574	5W	MGDG-O(FA 18:4;O) 36:8;O2 (alternative fragmentation; Arabidopside	acMGDG(18:4-O/36:8-2O) (alternative fragmentation;
lipid0540	5W	MGDG-O(FA 18:4;O) 36:8;O2 (Arabidopside G)	acMGDG(18:4-O/36:8-2O) (Arabidopside G)
lipid0559	5X	MGDG-O(FA 18:4;O2) 34:6	acMGDG(18:4-2O/34:6)
lipid0541	5X	MGDG-O(FA 18:4;O) 38:4 or MGDG-O(FA 18:4;O) 36:6;O2	acMGDG(18:4-O/38:4) or acMGDG(18:4-O/36:6-2O)
lipid0560	5Y	MGDG-O(FA 18:4;O2) 34:7;O	acMGDG(18:4-2O/34:7-O)
lipid0561	5Y	MGDG-O(FA 18:4;O2) 36:6 or MGDG-O(FA 18:4;O2) 34:8;O2	acMGDG(18:4-2O/36:6) or acMGDG(18:4-2O/34:8-2O)
lipid0565	5Z	MGDG-O(FA 18:4;O2) 36:8;O2	acMGDG(18:4-2O/36:8-2O)
lipid0586	5Z	MGDG-O(FA 18:4;O3) 34:6	acMGDG(18:4-3O/34:6)
lipid0587	5AA	MGDG-O(FA 18:4;O3) 34:7;O	acMGDG(18:4-3O/34:7-O)
lipid0588	5AA	MGDG-O(FA 18:4;O3) 36:6 or MGDG-O(FA 18:4;O3) 34:8;O2	acMGDG(18:4-3O/36:6) or acMGDG(18:4-3O/34:8-2O)
lipid0556	5BB	MGDG-O(FA 18:5;O2) 36:6 or MGDG-O(FA 18:5;O2) 34:8;O2	acMGDG(18:5-2O/36:6) or acMGDG(18:5-2O/34:8-2O)
lipid0558	5BB	MGDG-O(FA 18:5;O2) 36:8;O2	acMGDG(18:5-2O/36:8-2O)
lipid0293	5CC	PG-O(FA 16:1) 36:8;O2**	acPG(16:1/36:8-2O)**

Ambiguous (with regard to oxidation), acylated chloroplast lipids

lipid0476	5DD	MGDG-O(FA 16:0) 36:6 or MGDG-O(FA 16:0) 34:8;O2	acMGDG(16:0/36:6) or acMGDG(16:0/34:8-2O)

lipid0468	5DD	MGDG-O(FA 16:1) 36:6 or MGDG-O(FA 16:1) 34:8;O2	acMGDG(16:1/36:6) or acMGDG(16:1/34:8-2O)
lipid0461	5EE	MGDG-O(FA 16:2) 36:6 or MGDG-O(FA 16:2) 34:8;O2	acMGDG(16:2/36:6) or acMGDG(16:2/34:8-2O)
lipid0454	5EE	MGDG-O(FA 16:3) 36:6 or MGDG-O(FA 16:3) 34:8;O2	acMGDG(16:3/36:6) or acMGDG(16:3/34:8-2O)
lipid0519	5FF	MGDG-O(FA 16:3;O2) 34:6 or MGDG-O(FA 18:1) 34:6	acMGDG(16:3-2O/34:6) or acMGDG(18:1/34:6)
lipid0522	5FF	MGDG-O(FA 16:3;O2) 36:6 or MGDG-O(FA 16:3;O2) 34:8;O2 or MGDG-O(FA	acMGDG(16:3-2O/36:6) or acMGDG(16:3-2O/34:8-2O) or
lipid0525	5GG	MGDG-O(FA 16:3;O2) 36:8;O2 or MGDG-O(FA 18:1) 36:8;O2	acMGDG(16:3-2O/36:8-2O) or acMGDG(18:1/36:8-2O)
lipid0528	5GG	MGDG-O(FA 18:0) 34:8;O2 or MGDG-O(FA 18:0) 36:6	acMGDG(18:0/34:8-2O) or acMGDG(18:0/36:6)
lipid0516	5HH	MGDG-O(FA 18:2) 36:6 or MGDG-O(FA 18:2) 34:8;O2	acMGDG(18:2/36:6) or acMGDG(18:2/34:8-2O)
lipid0506	5HH	MGDG-O(FA 18:3) 34:7;O2 or MGDG-O(FA 18:3) 36:5	acMGDG(18:3/34:7-2O) or acMGDG(18:3/36:5)
lipid0505	5II	MGDG-O(FA 18:3) 36:6 or MGDG-O(FA 18:3) 34:8;O2	acMGDG(18:3/36:6) or acMGDG(18:3/34:8-2O)

Non-oxidized, acylated chloroplast lipids

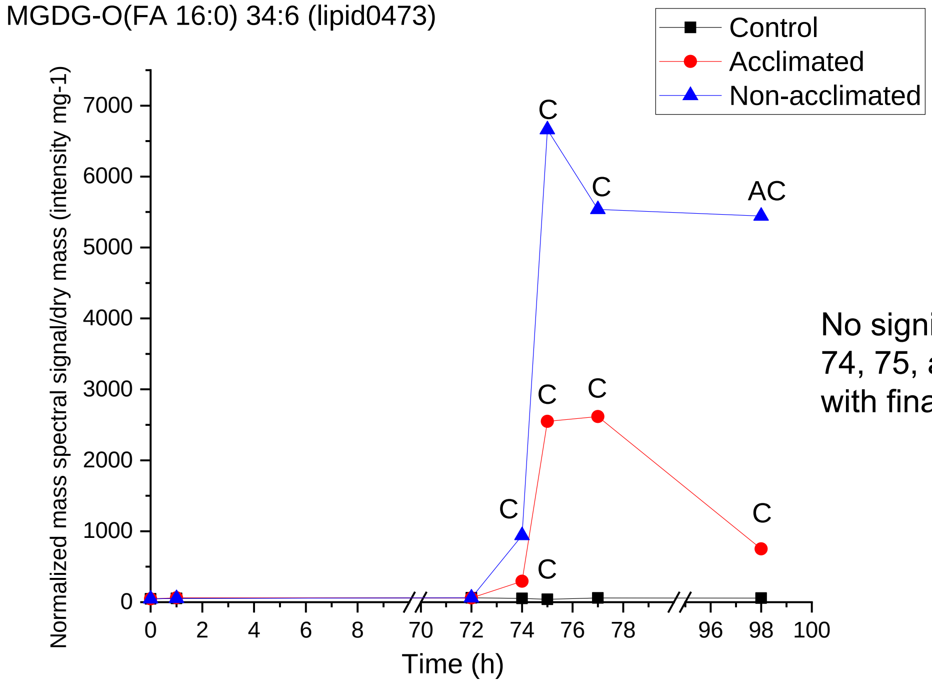

No significant correlation of 74, 75, and 77 h lipid levels with final ion leakage

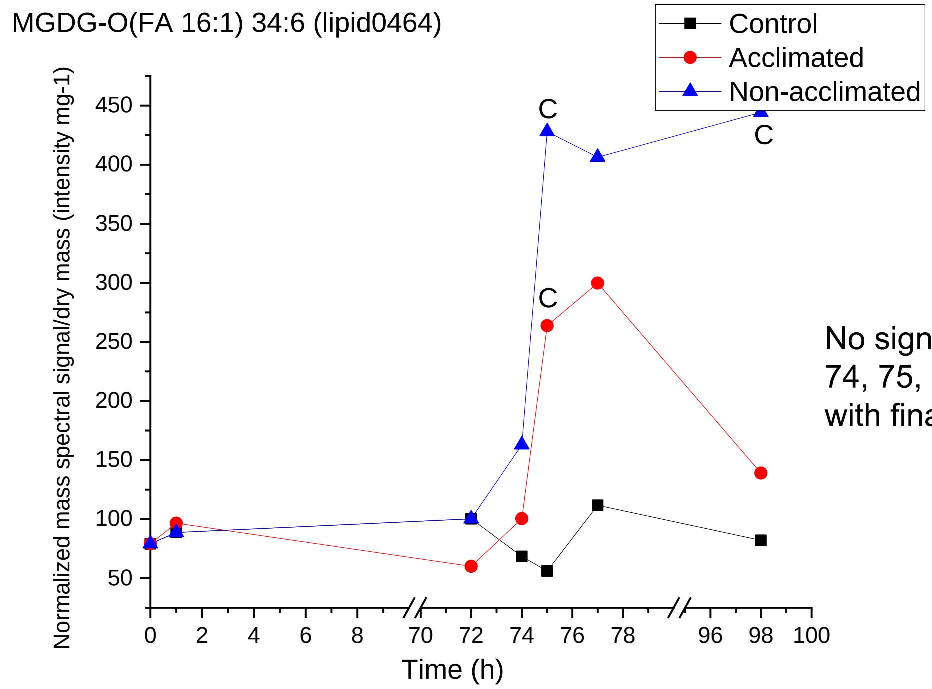

No significant correlation of 74, 75, and 77 h lipid levels with final ion leakage

MGDG-O(FA 16:2) 34:6 (lipid0459)

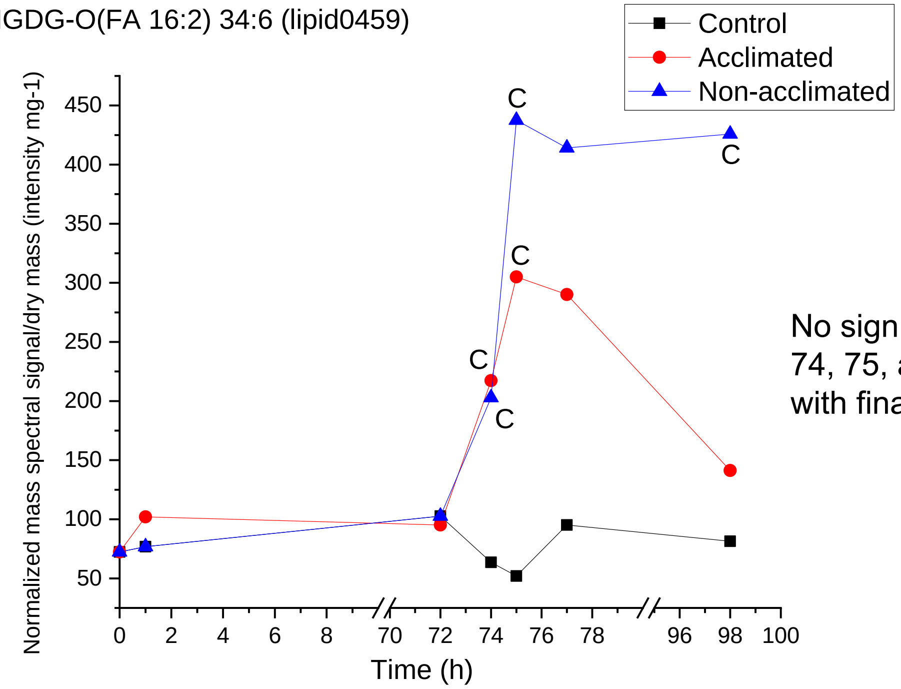

No significant correlation of 74, 75, and 77 h lipid levels with final ion leakage

MGDG-O(FA 16:3) 34:6 (lipid0448)

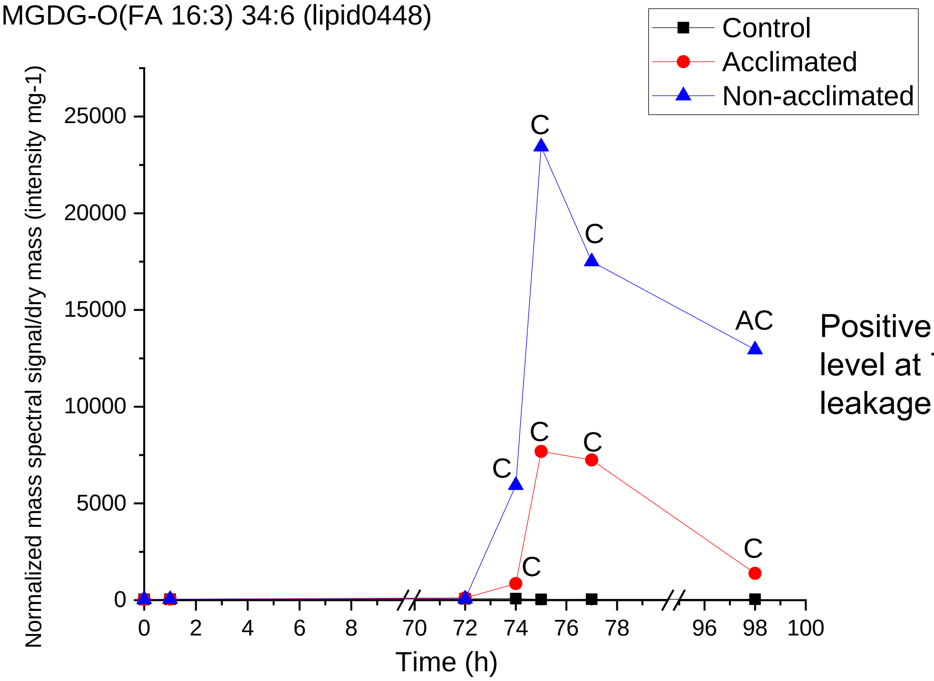

Positive correlation of lipid level at 74 h with final ion leakage

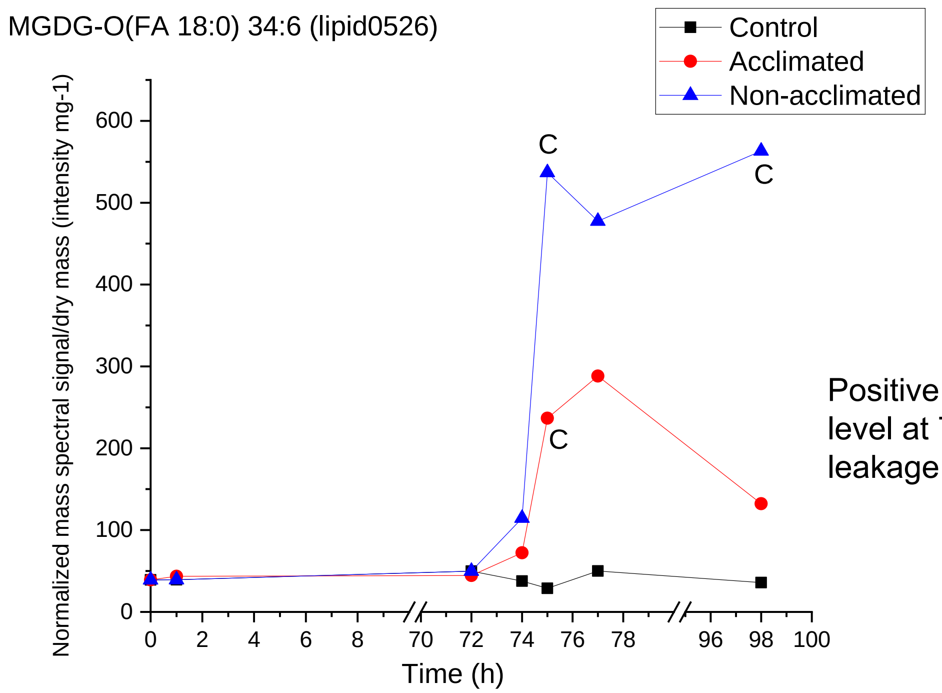

Positive correlation of lipid level at 75 h with final ion leakage

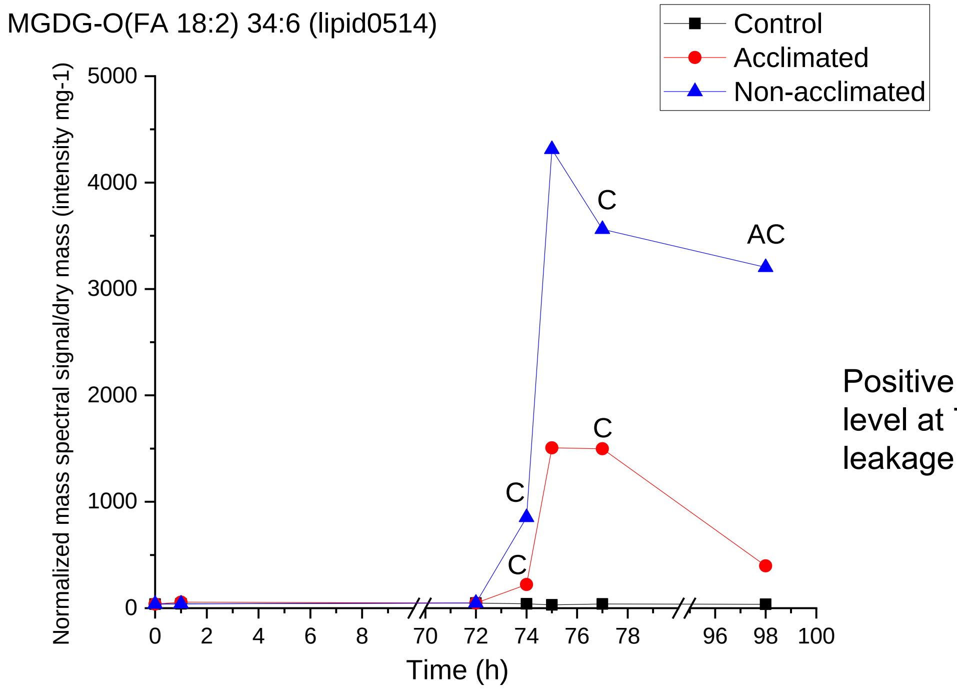

Positive correlation of lipid level at 74 h with final ion leakage

5C

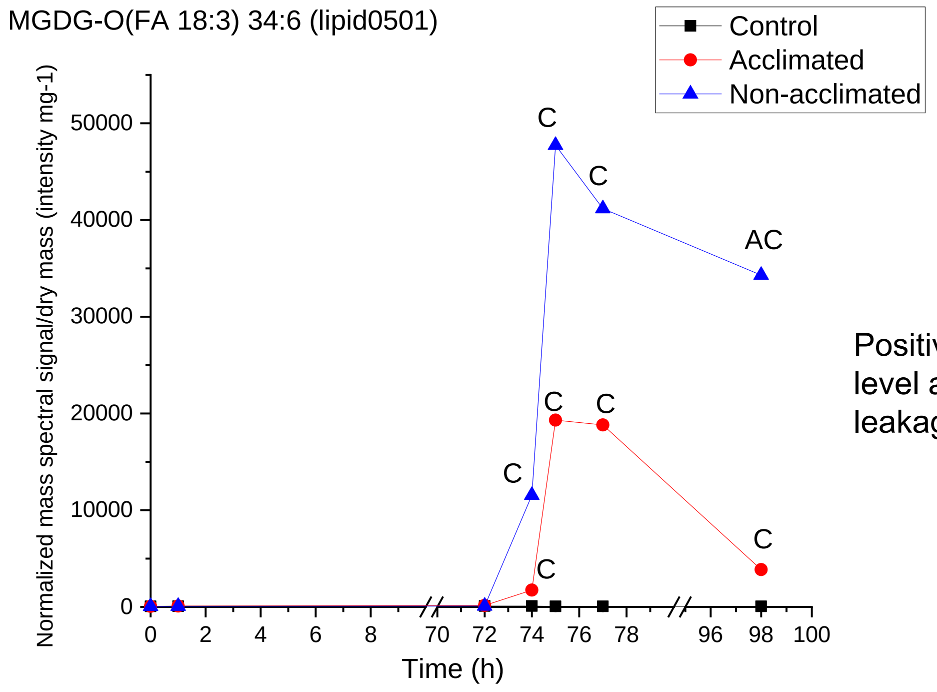

Positive correlation of lipid level at 74 h with final ion leakage

5D

Oxidized (at least one oxidized acyl chain), acylated chloroplast lipids

DGDG-O(FA 18:4;O) 36:6 or DGDG-O(FA 18:4;O) 34:8;O2 (lipid0601)

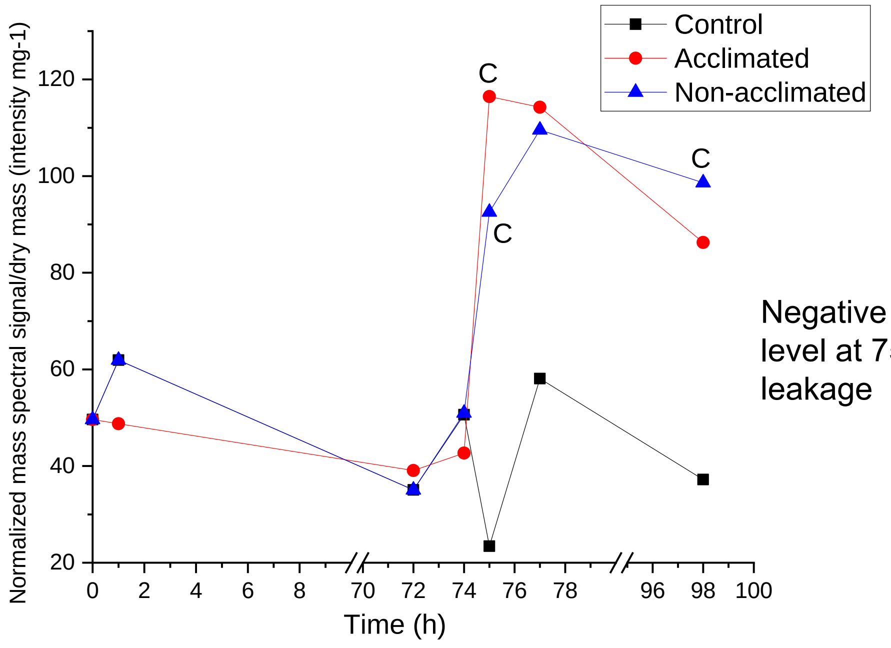

Negative correlation of lipid level at 75 h with final ion leakage

DGDG-O(FA 18:4;O) 36:8;O2 (lipid0603)

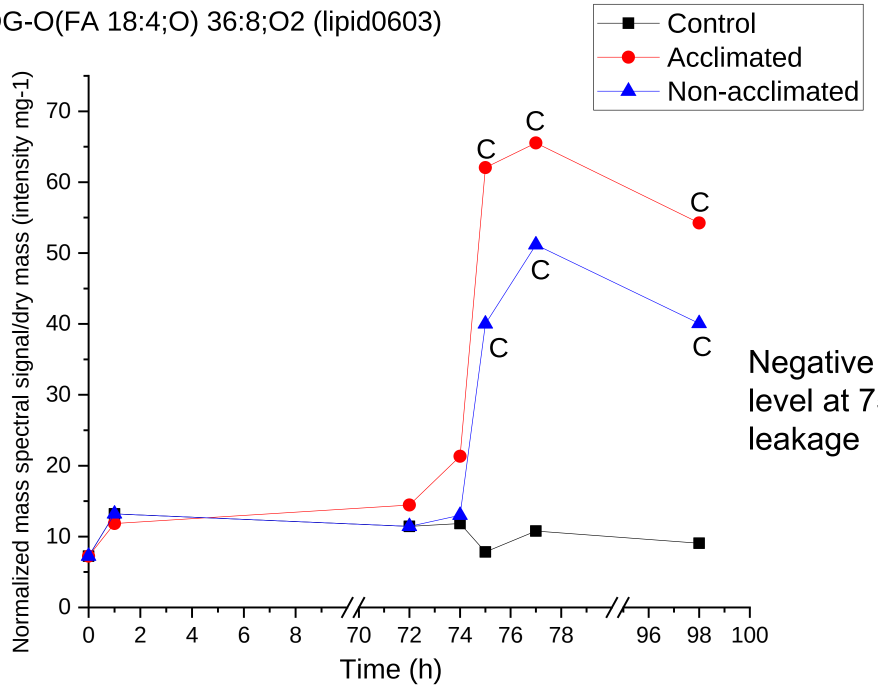

Negative correlation of lipid level at 75 h with final ion leakage

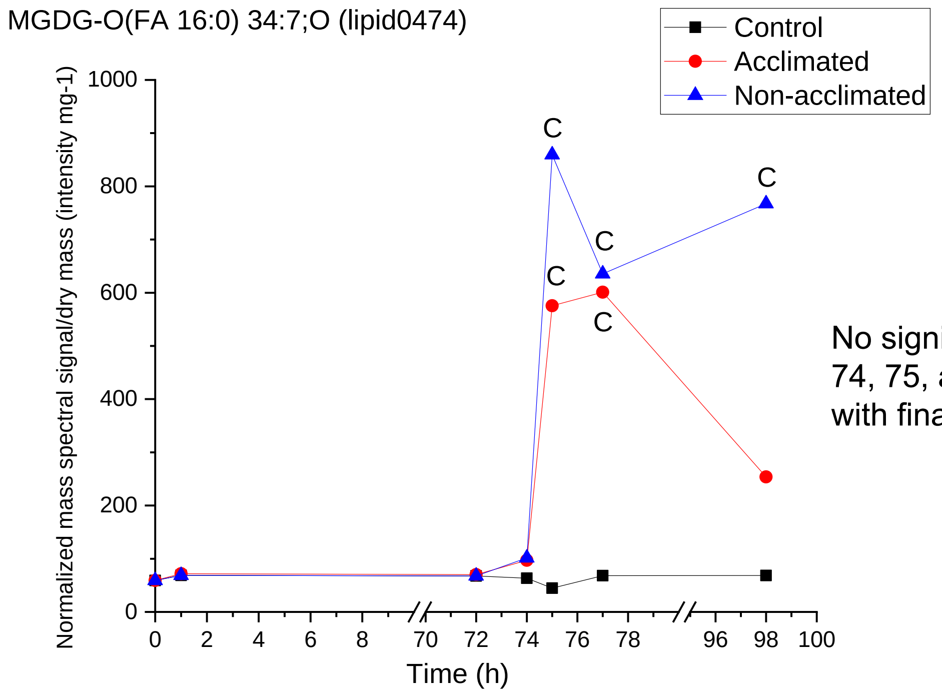

No significant correlation of 74, 75, and 77 h lipid levels with final ion leakage

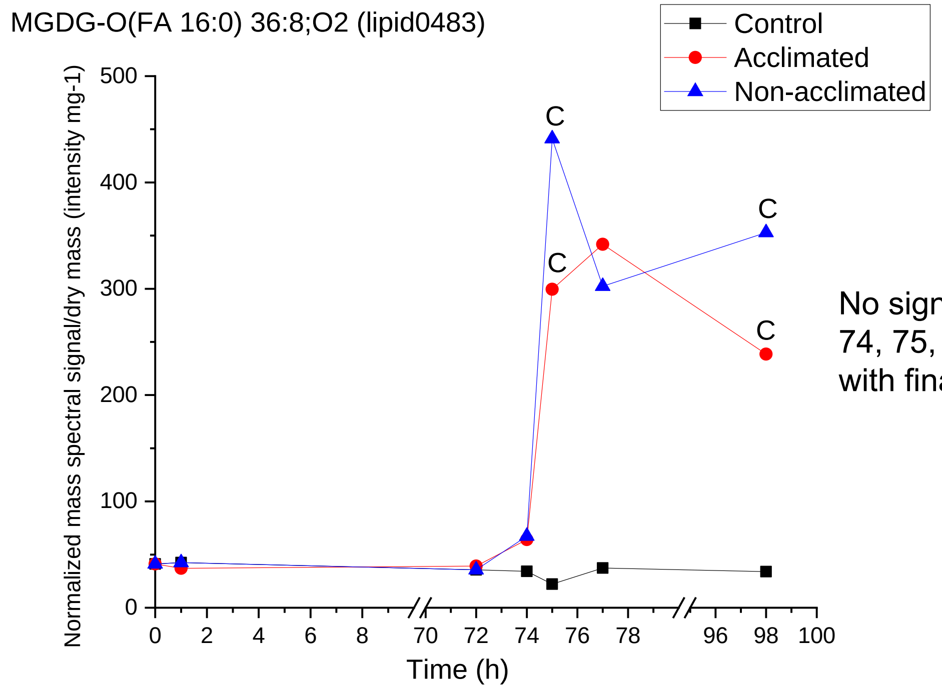

No significant correlation of 74, 75, and 77 h lipid levels with final ion leakage

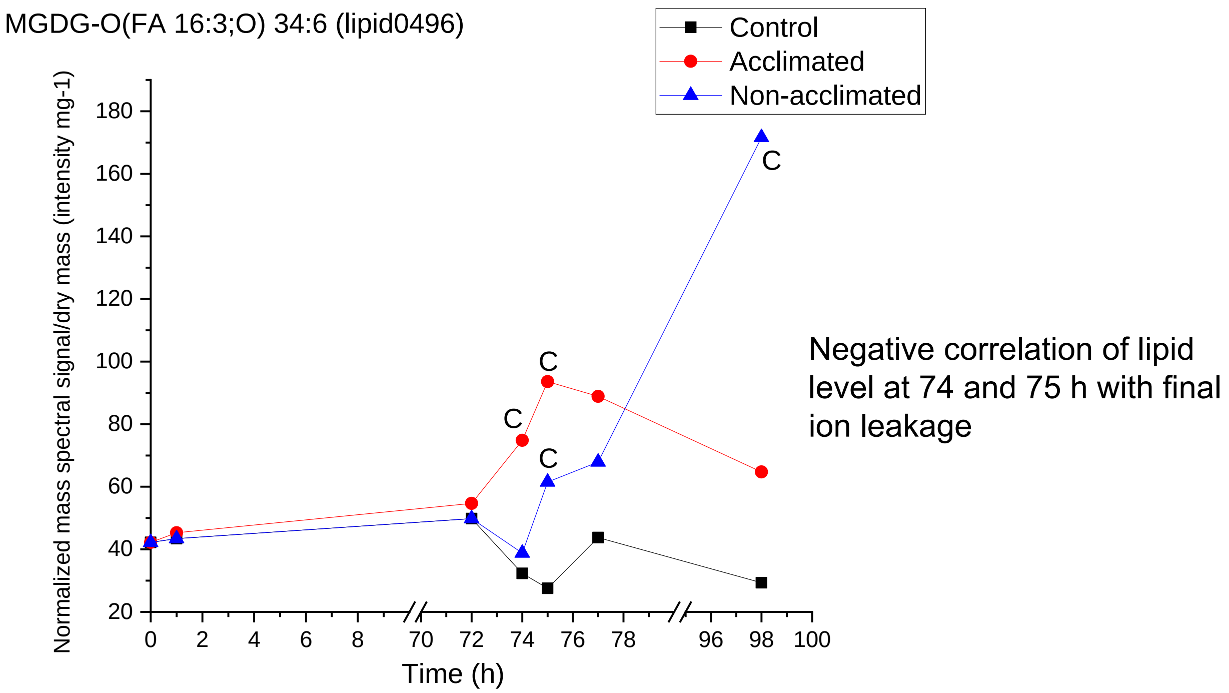
MGDG-O(FA 16:3;O) 34:6 (lipid0496)
Control
Acclimated
Non-acclimated
Normalized mass spectral signal/dry mass (intensity mg-1)
Time (h)
C
C
C
C
Negative correlation of lipid level at 74 and 75 h with final ion leakage

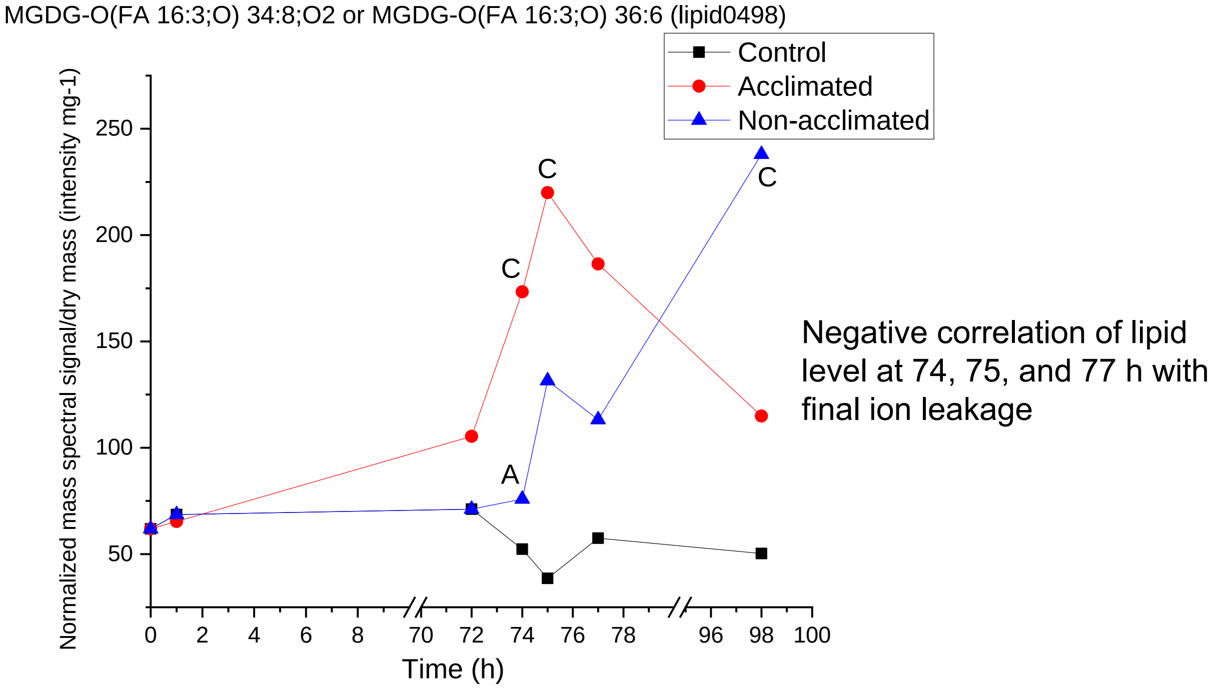
MGDG-O(FA 16:3;O) 34:8;O2 or MGDG-O(FA 16:3;O) 36:6 (lipid0498)
Control
Acclimated
Non-acclimated
Normalized mass spectral signal/dry mass (intensity mg-1)
Time (h)
C
C
A
C
Negative correlation of lipid level at 74, 75, and 77 h with final ion leakage

5G

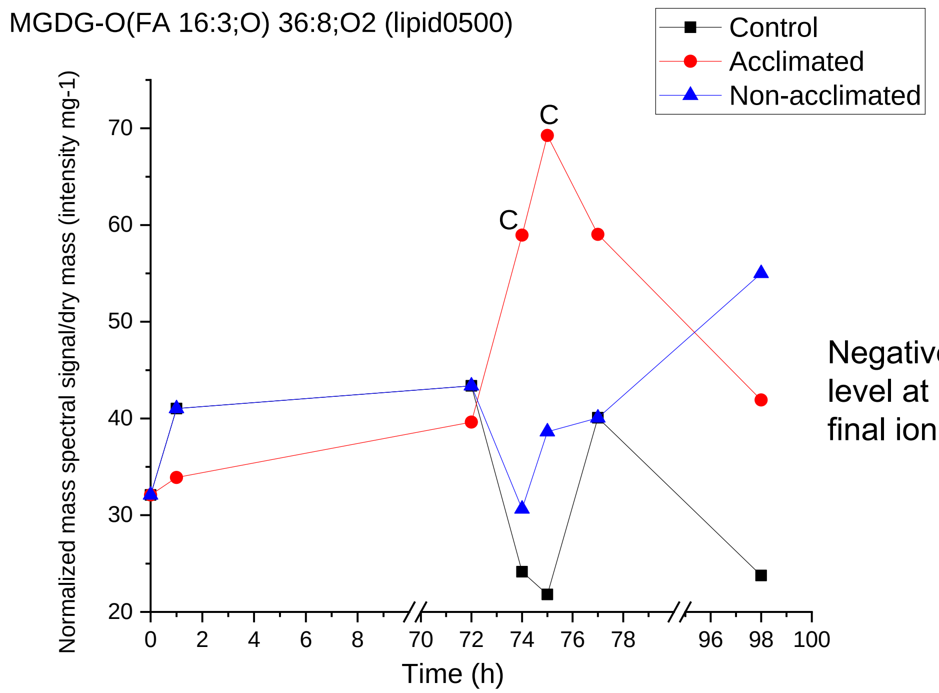

Negative correlation of lipid level at 74 and 75 h with final ion leakage

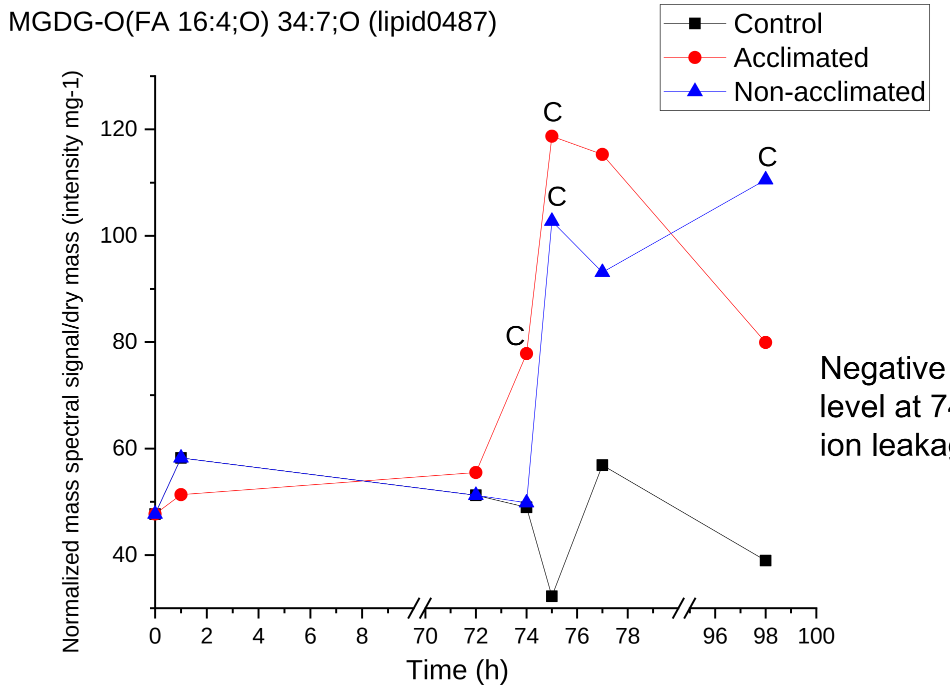

Negative correlation of lipid level at 74 and 75 h with final ion leakage

MGDG-O(FA 16:4;O) 34:8;O2 or MGDG-O(FA 16:4;O) 36:6 (lipid0489)

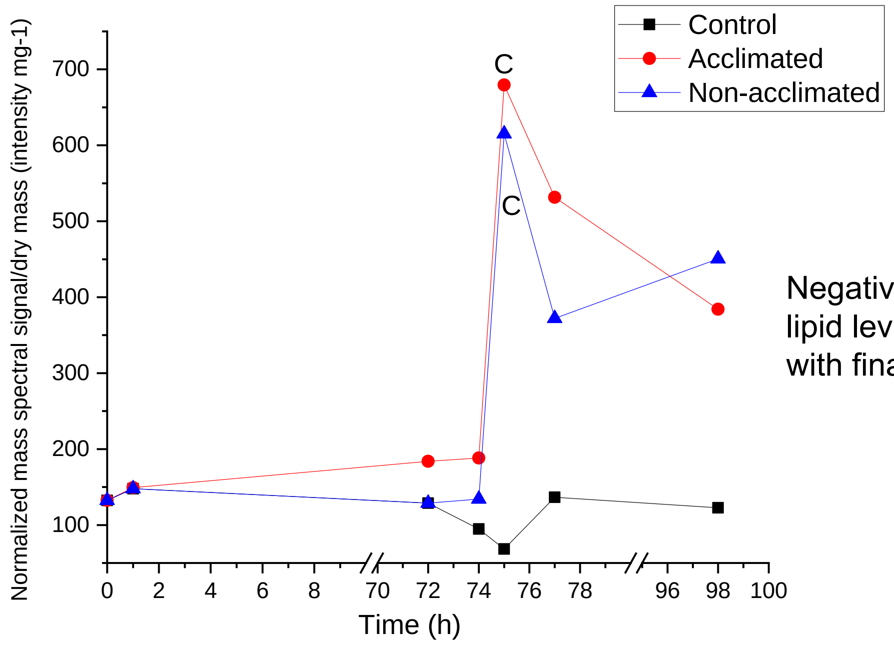

Negative correlation of lipid level at 74 and 77 h with final ion leakage

MGDG-O(FA 16:4;O) 36:8;O2 (lipid0495)

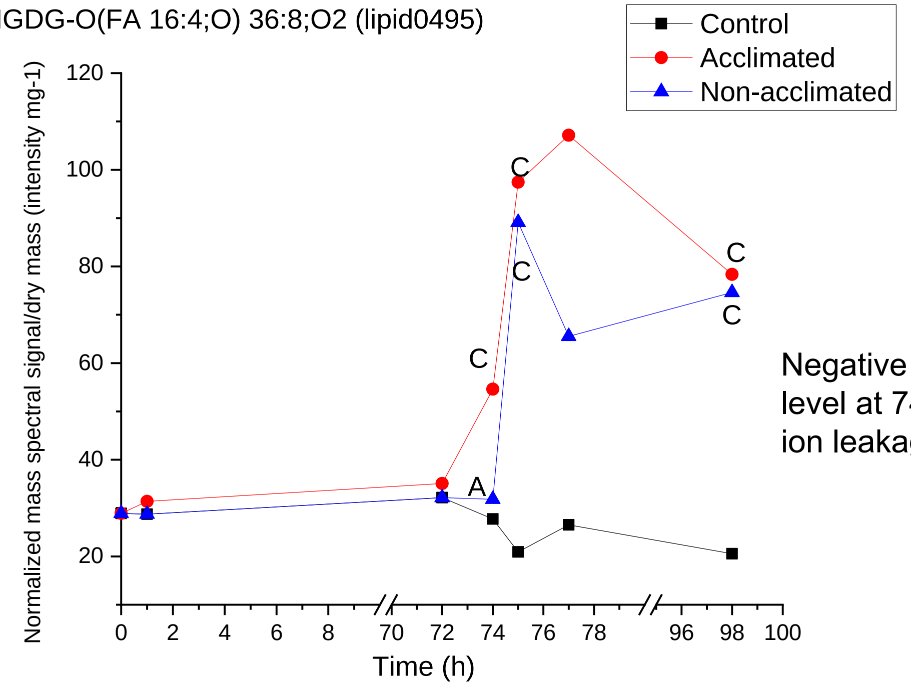

Negative correlation of lipid level at 74 and 77 h with final ion leakage

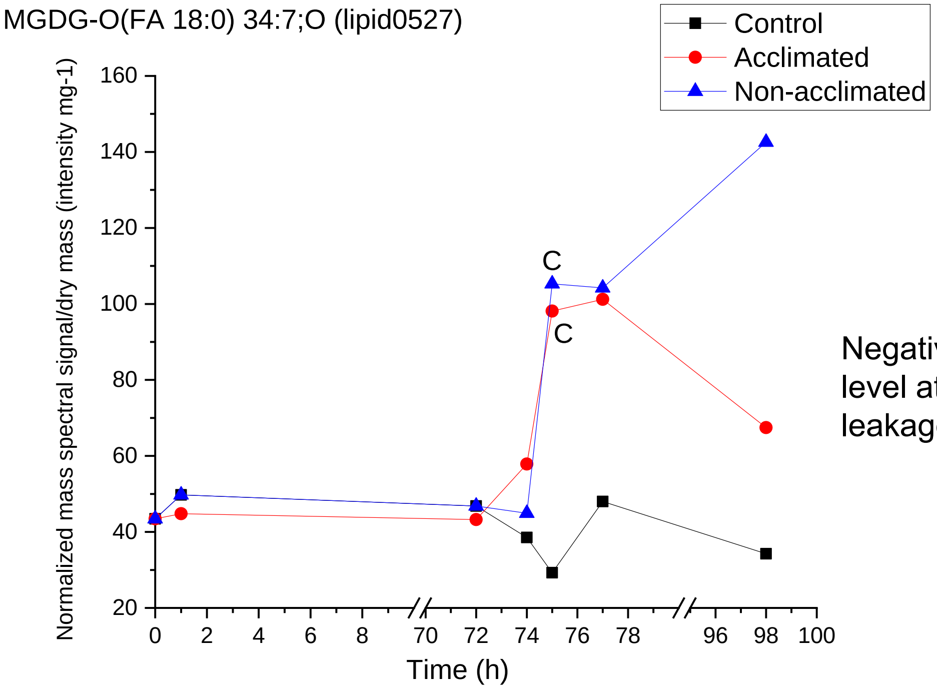

Negative correlation of lipid level at 74 h with final ion leakage

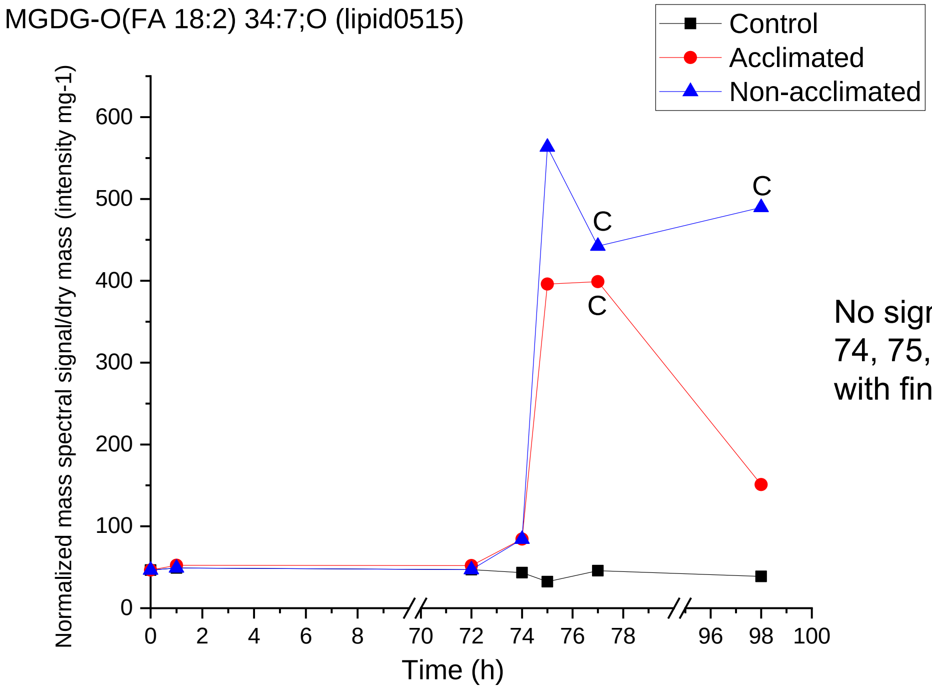

No significant correlation of 74, 75, and 77 h lipid levels with final ion leakage

5J

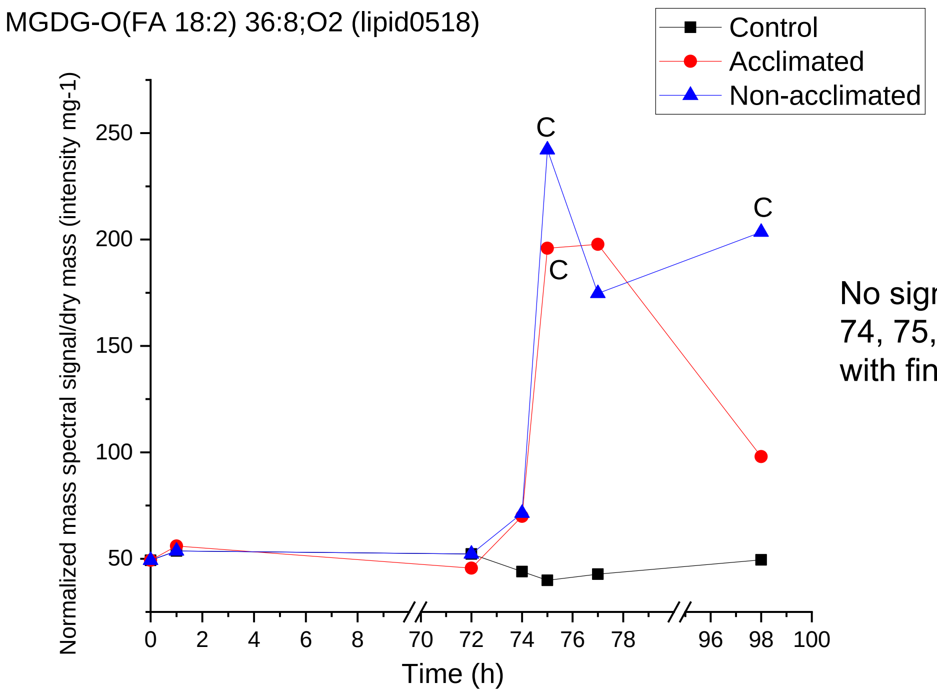

No significant correlation of 74, 75, and 77 h lipid levels with final ion leakage

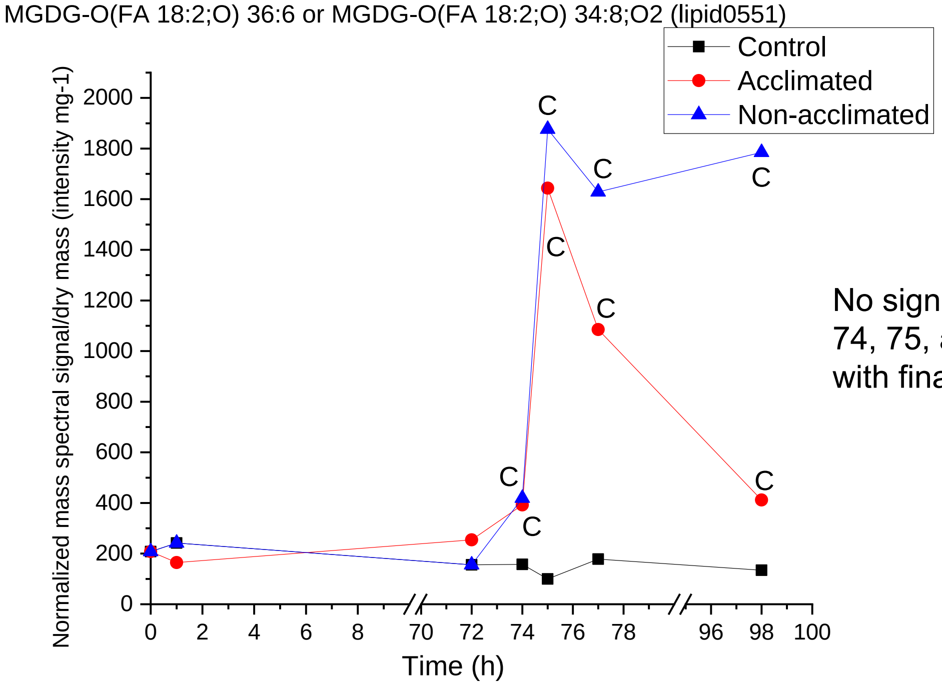

No significant correlation of 74, 75, and 77 h lipid levels with final ion leakage

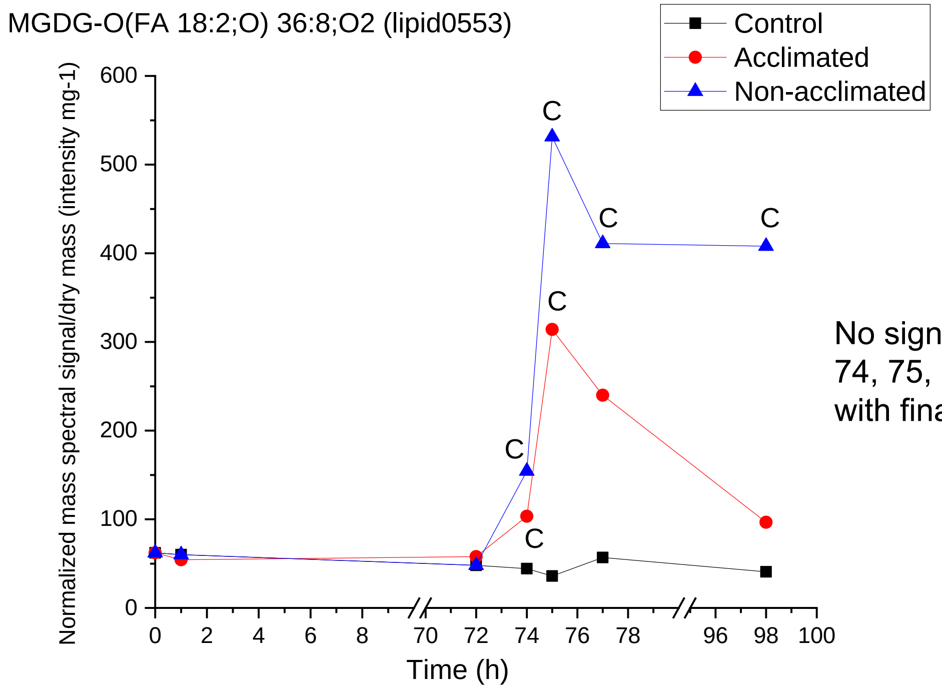

No significant correlation of 74, 75, and 77 h lipid levels with final ion leakage

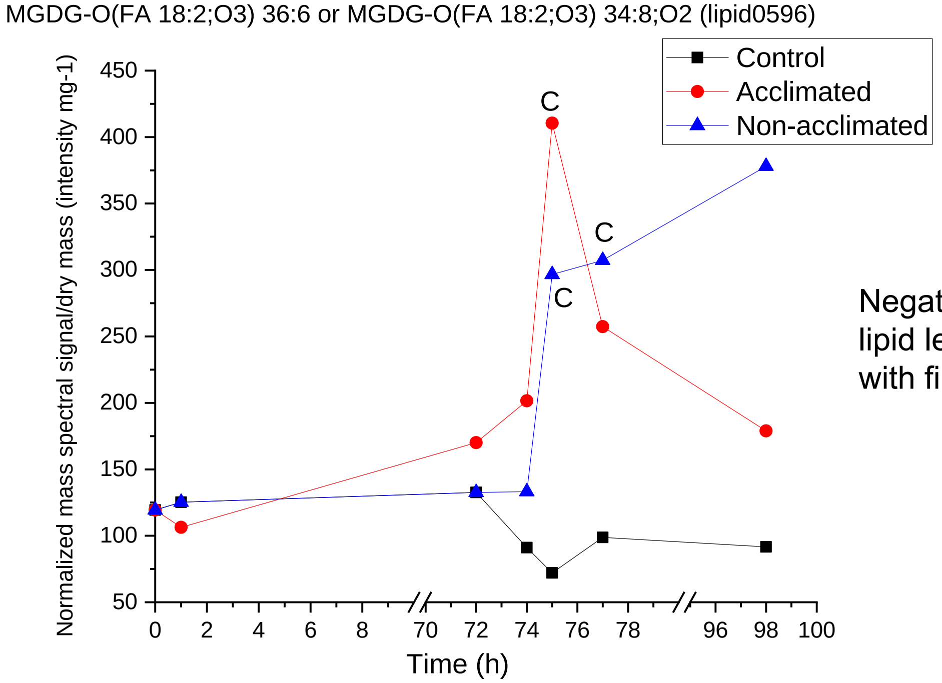

Negative correlation of lipid level at 74 and 75 h with final ion leakage

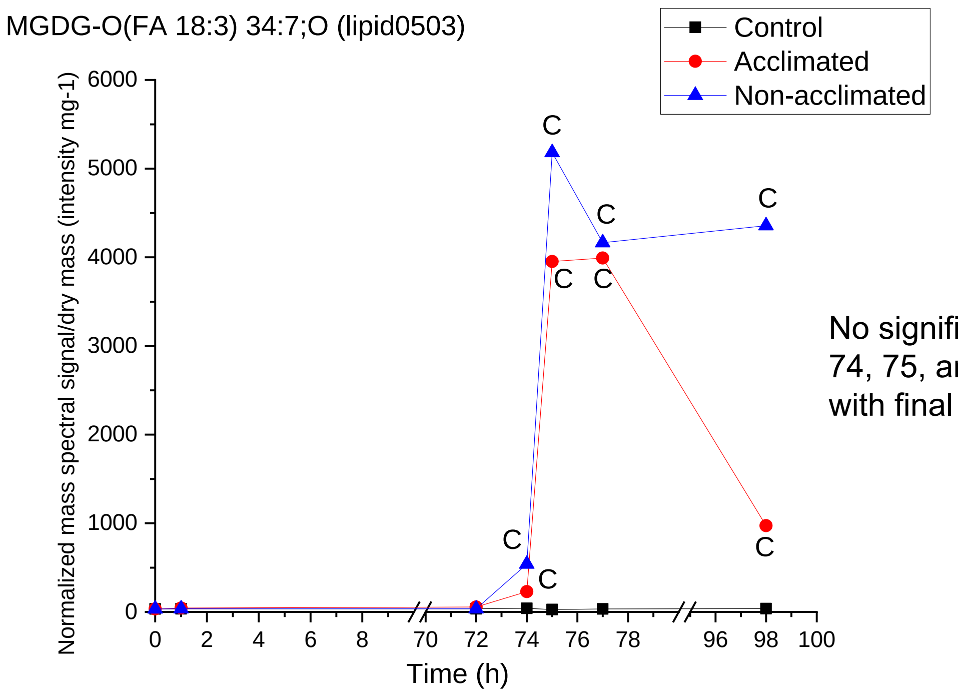

No significant correlation of 74, 75, and 77 h lipid levels with final ion leakage

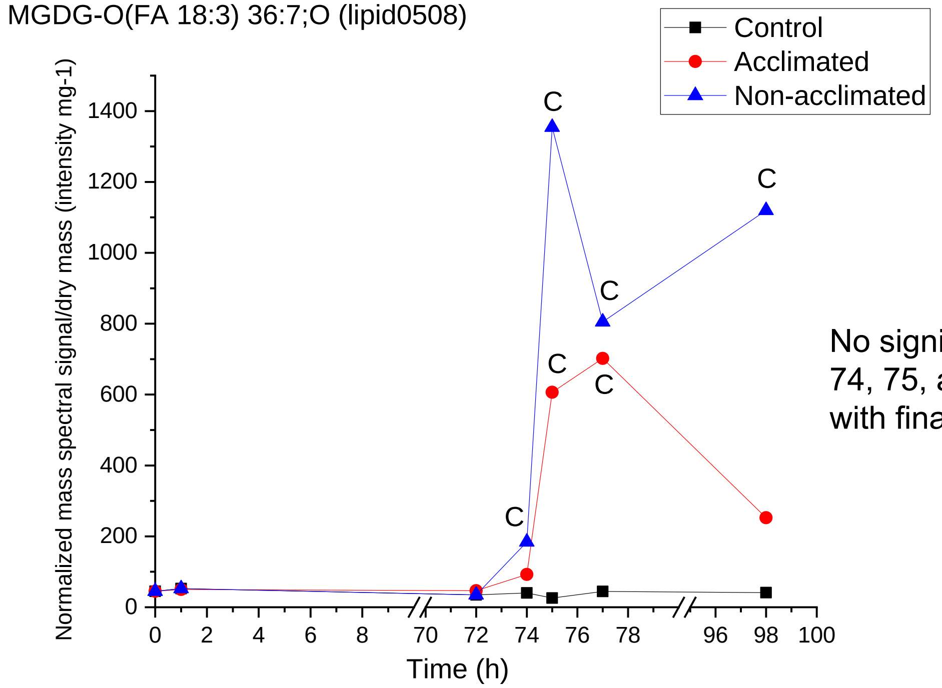

No significant correlation of 74, 75, and 77 h lipid levels with final ion leakage

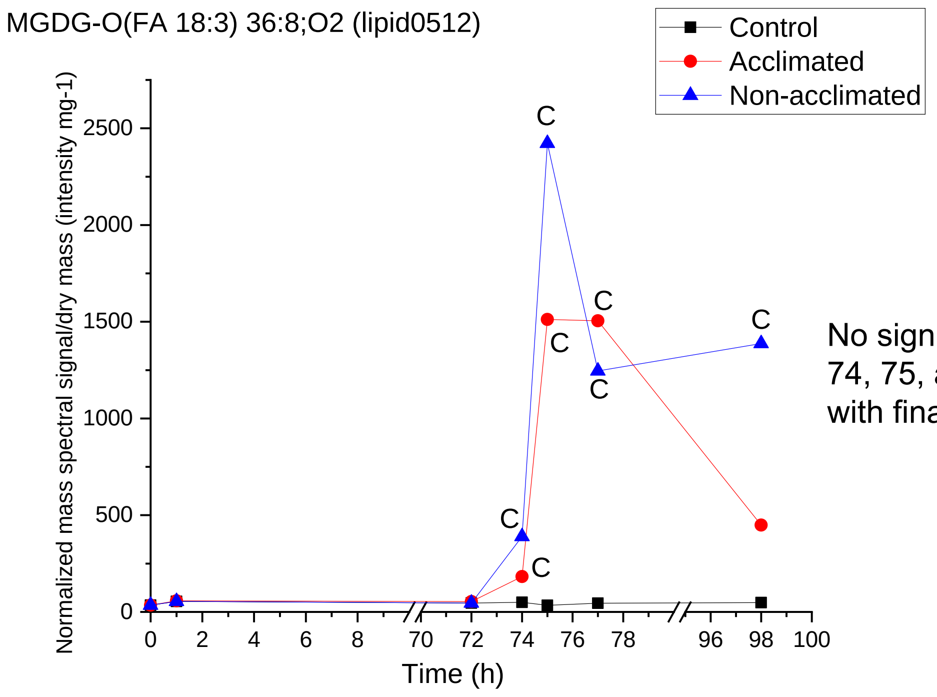

No significant correlation of 74, 75, and 77 h lipid levels with final ion leakage

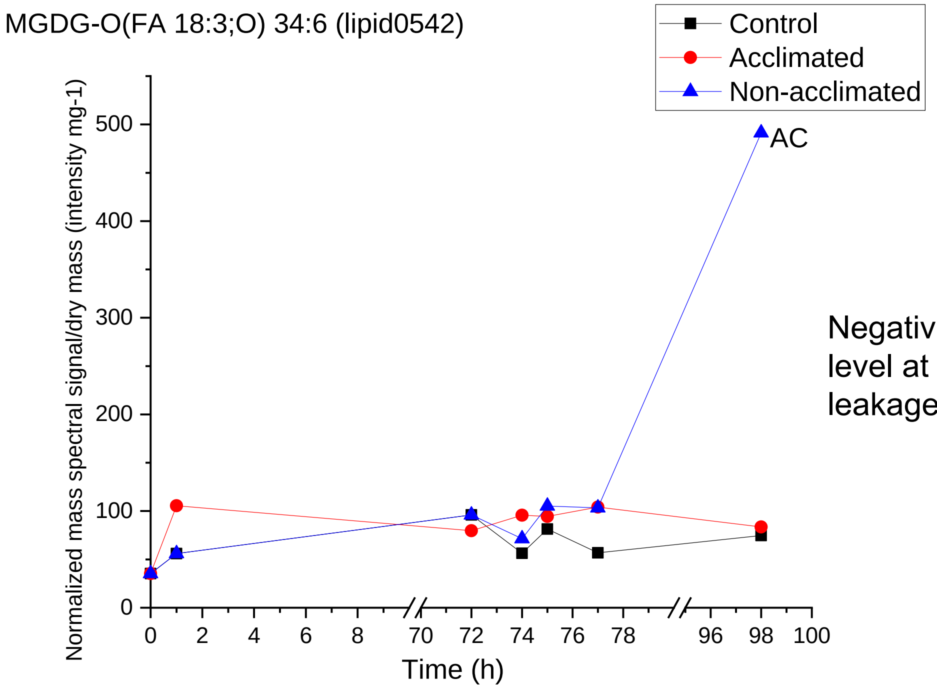

Negative correlation of lipid level at 74 h with final ion leakage

MGDG-O(FA 18:3;O) 34:7;O (lipid0543)

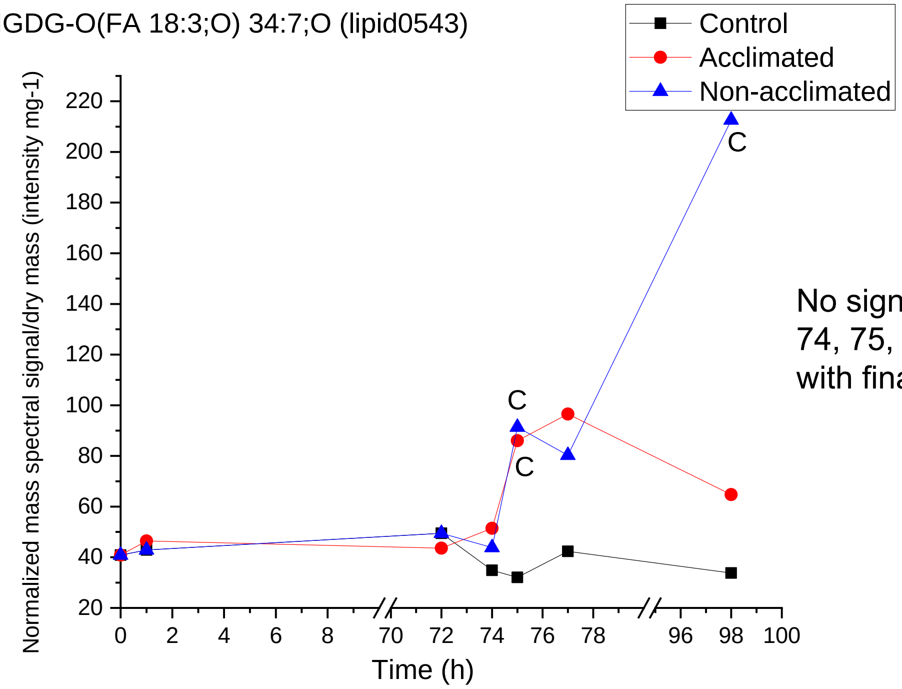

No significant correlation of 74, 75, and 77 h lipid levels with final ion leakage

MGDG-O(FA 18:3;O) 36:6 or MGDG-O(FA 18:3;O) 34:8;O2 (lipid0544)

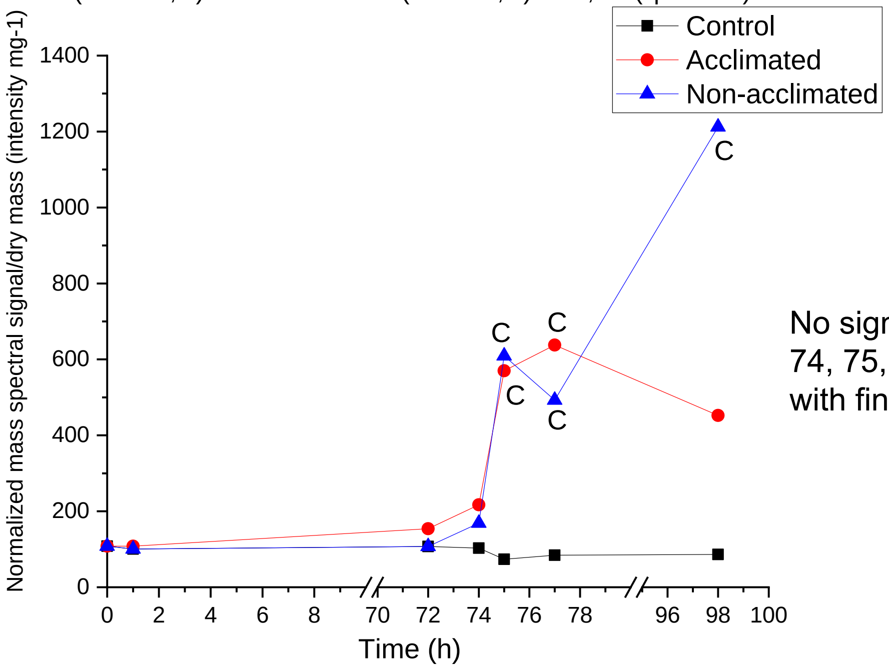

No significant correlation of 74, 75, and 77 h lipid levels with final ion leakage

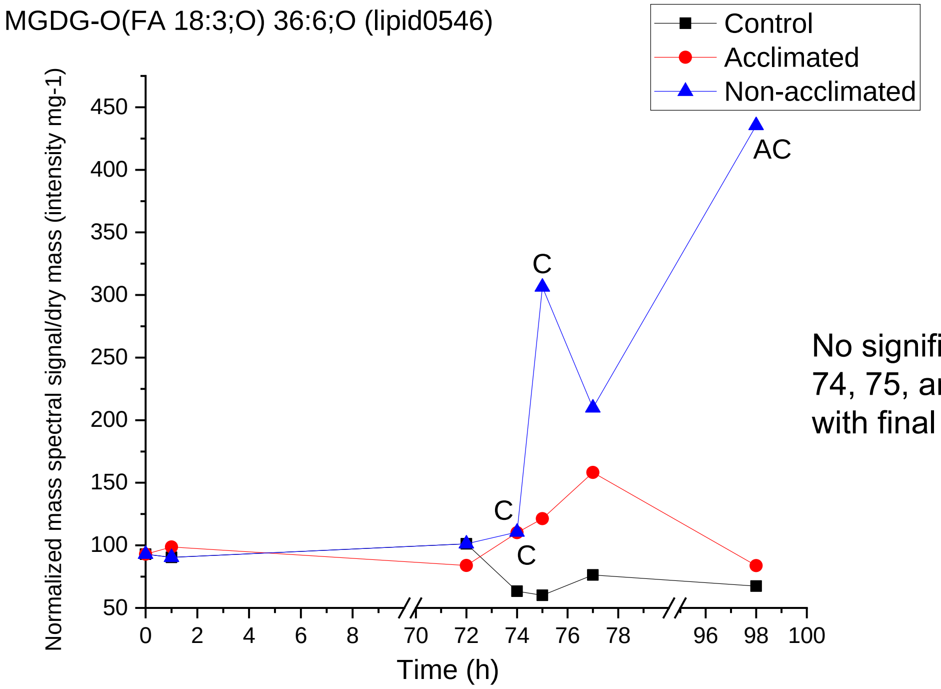

No significant correlation of 74, 75, and 77 h lipid levels with final ion leakage

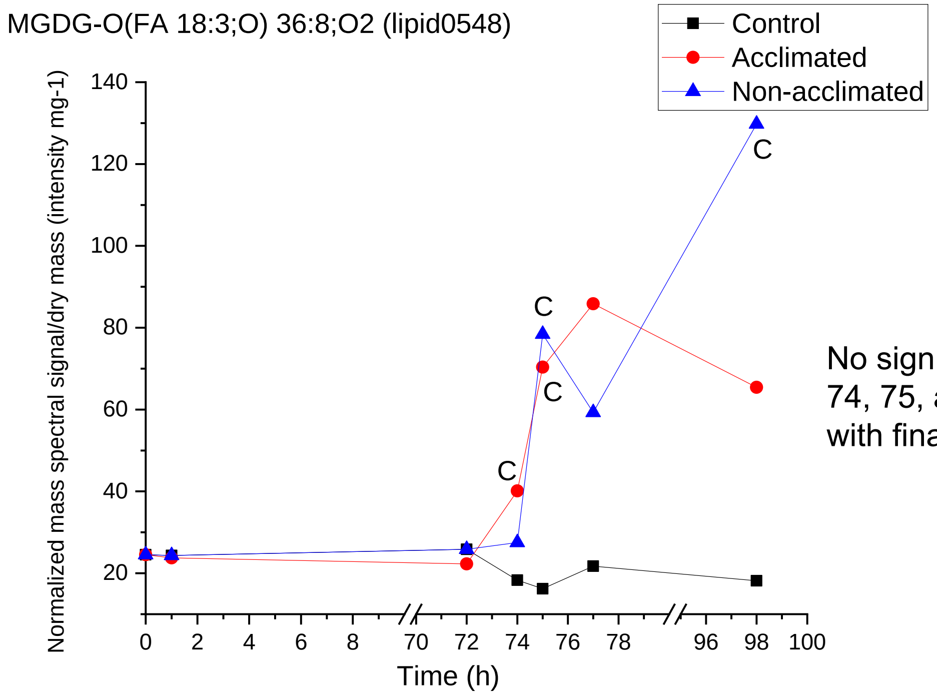

No significant correlation of 74, 75, and 77 h lipid levels with final ion leakage

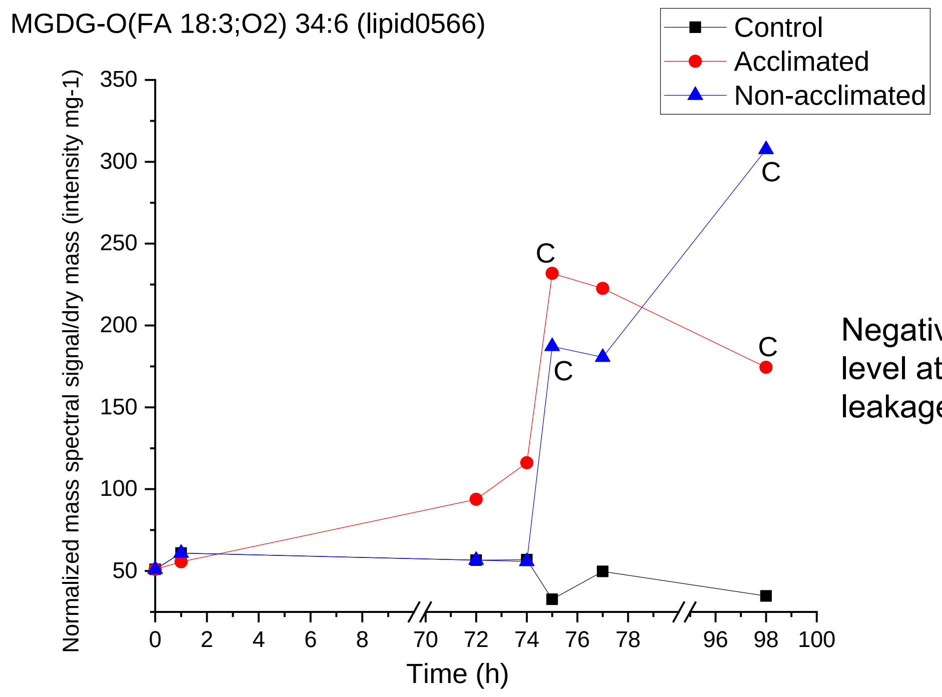

Negative correlation of lipid level at 74 h with final ion leakage

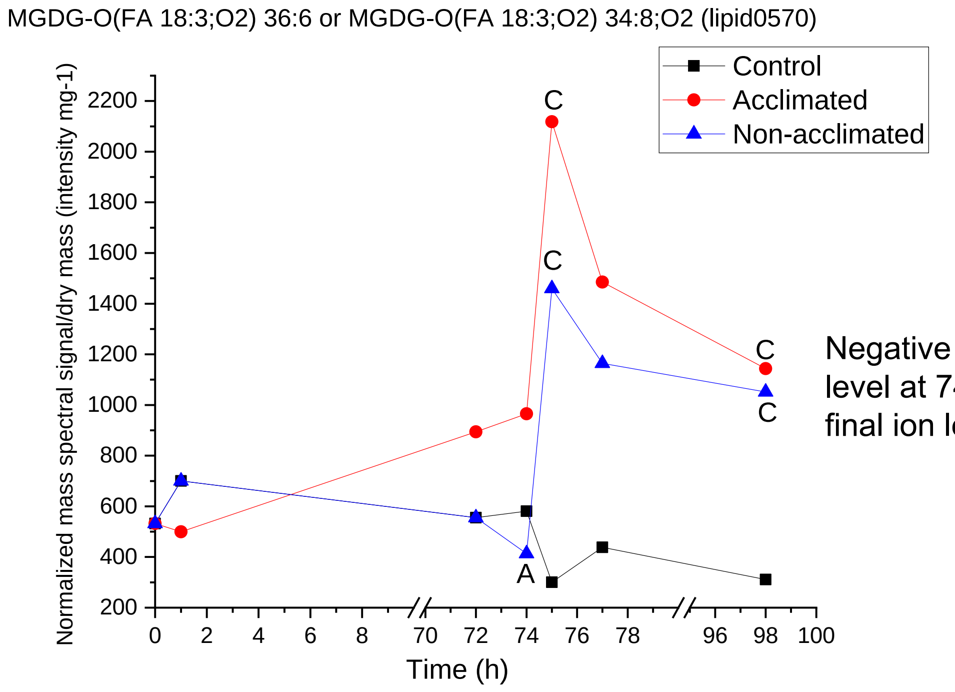

Negative correlation of lipid level at 74, 75, and 77 h with final ion leakage

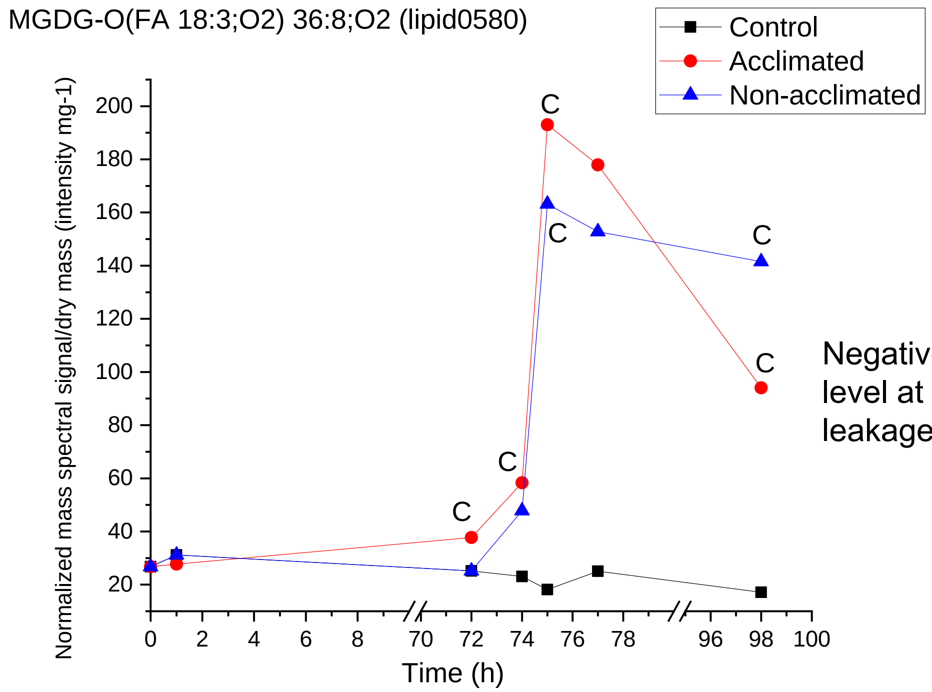

Negative correlation of lipid level at 74 h with final ion leakage

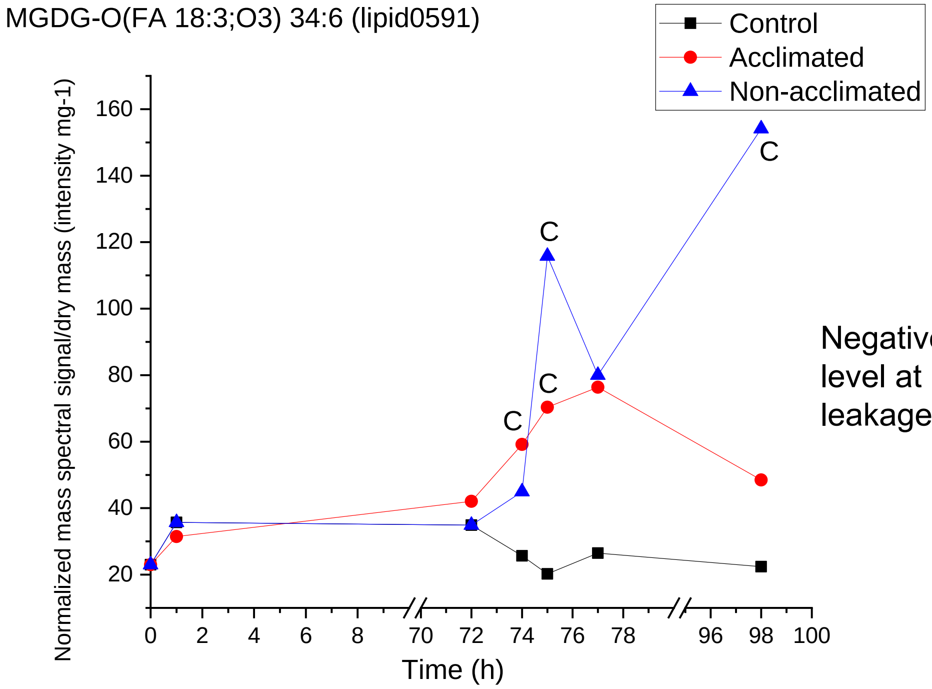

Negative correlation of lipid level at 74 h with final ion leakage

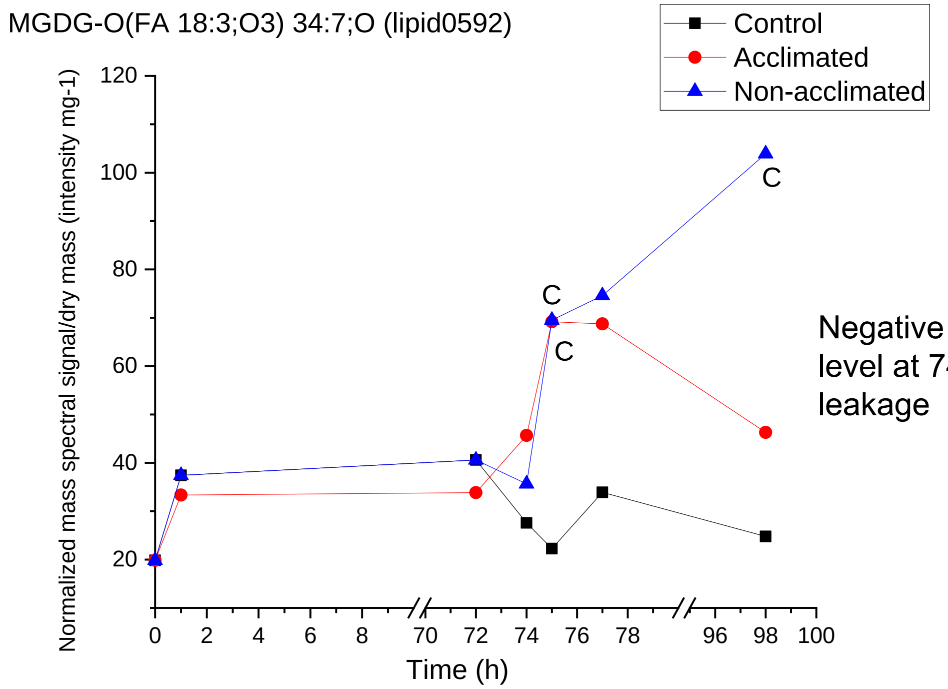

Negative correlation of lipid level at 74 h with final ion leakage

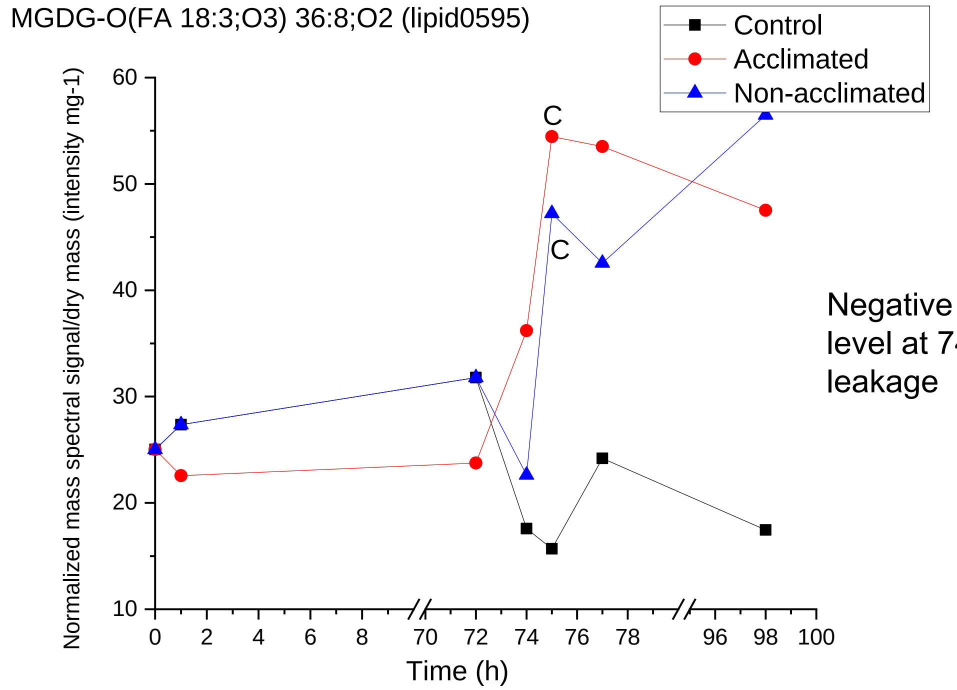

Negative correlation of lipid level at 74 h with final ion leakage

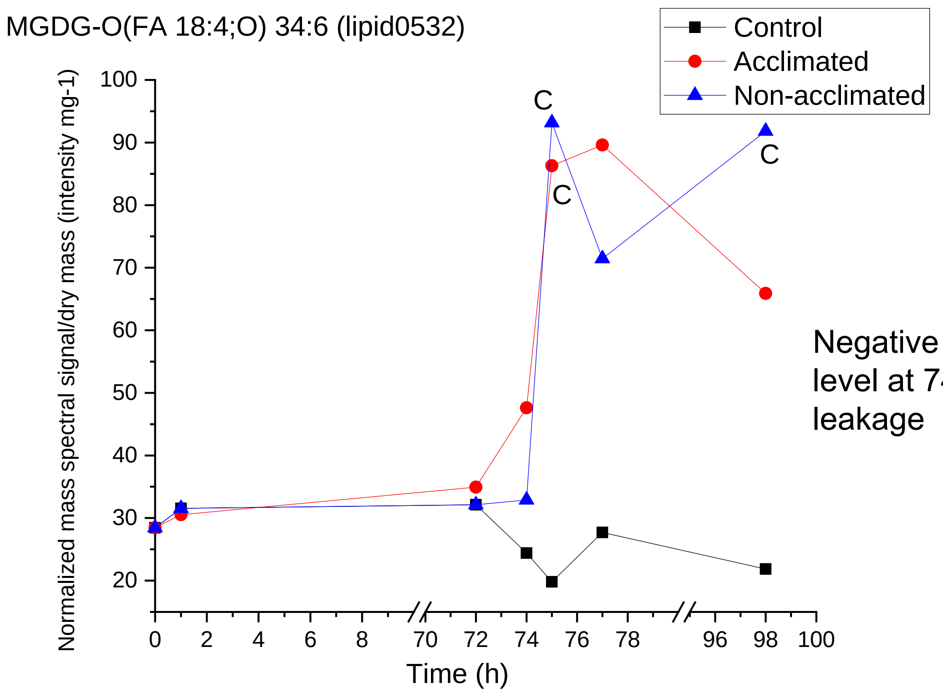

Negative correlation of lipid level at 74 h with final ion leakage

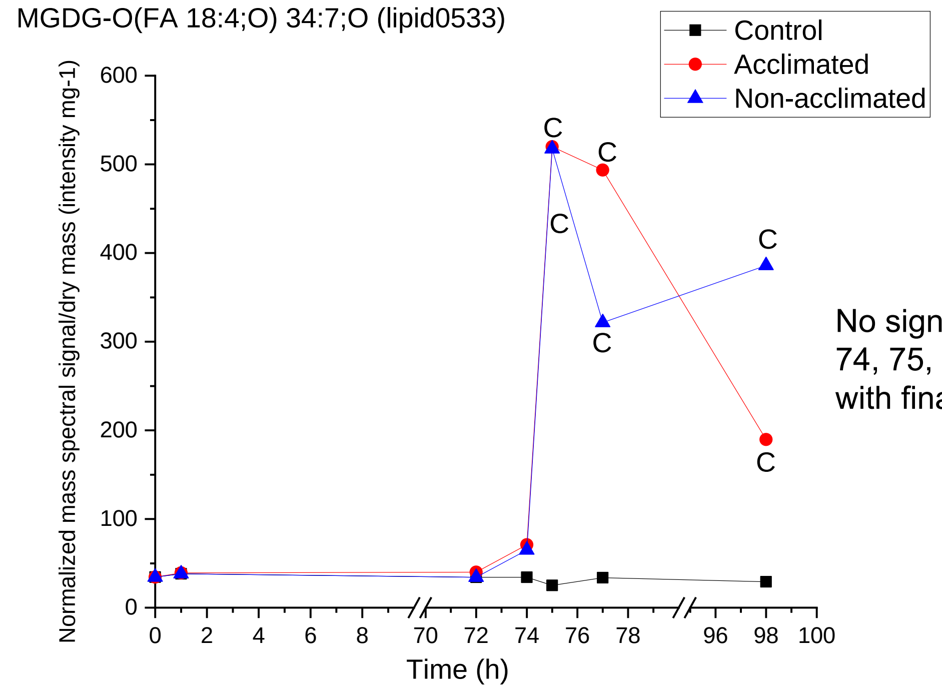

No significant correlation of 74, 75, and 77 h lipid levels with final ion leakage

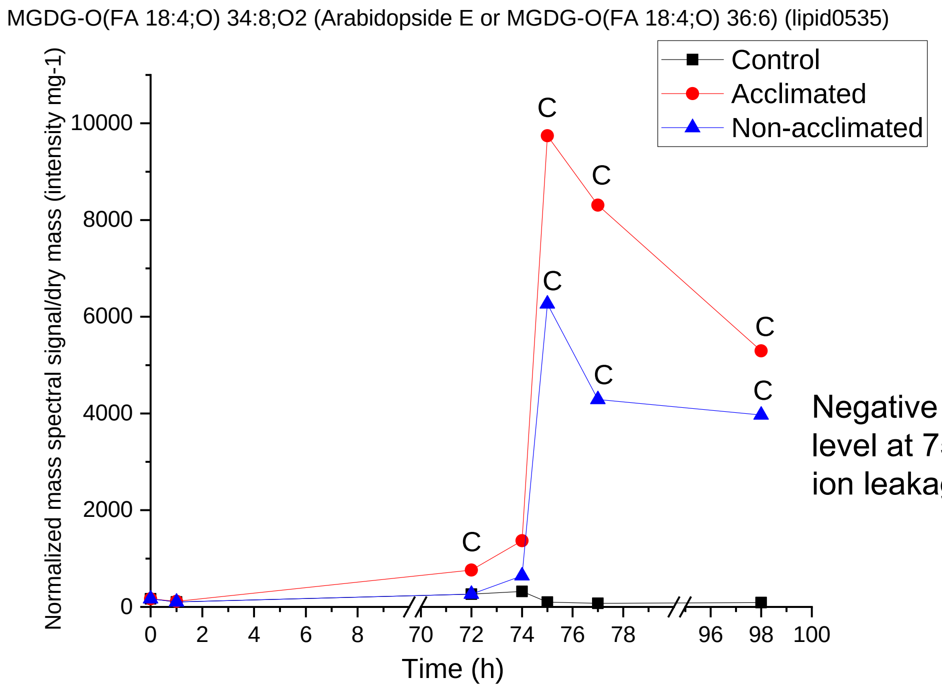

Negative correlation of lipid level at 75 and 77 h with final ion leakage

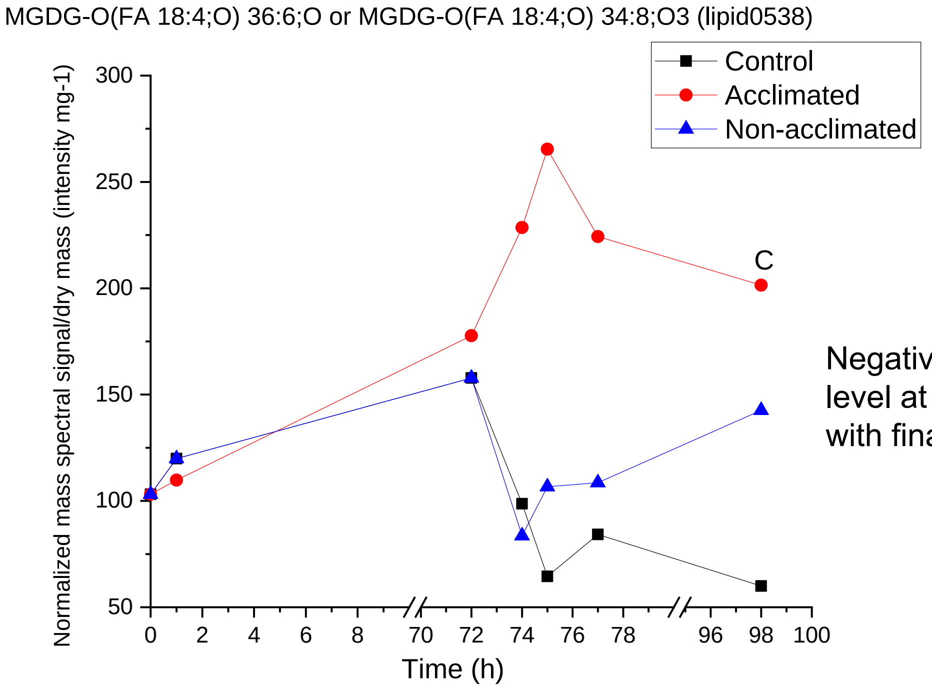

Negative correlation of lipid level at 74, 75, and 77 h with final ion leakage

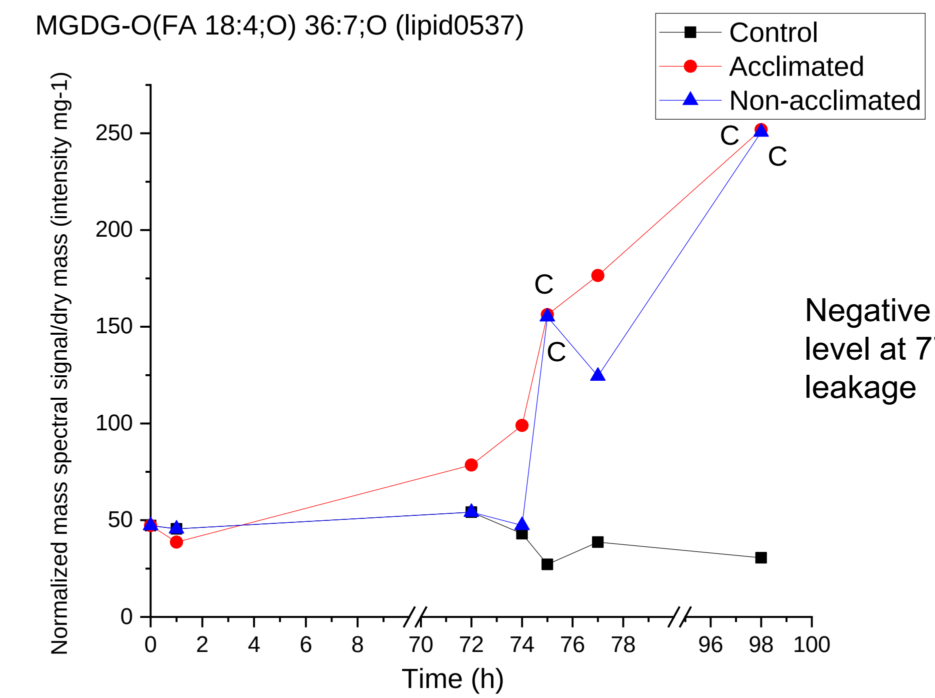

Negative correlation of lipid level at 77 h with final ion leakage

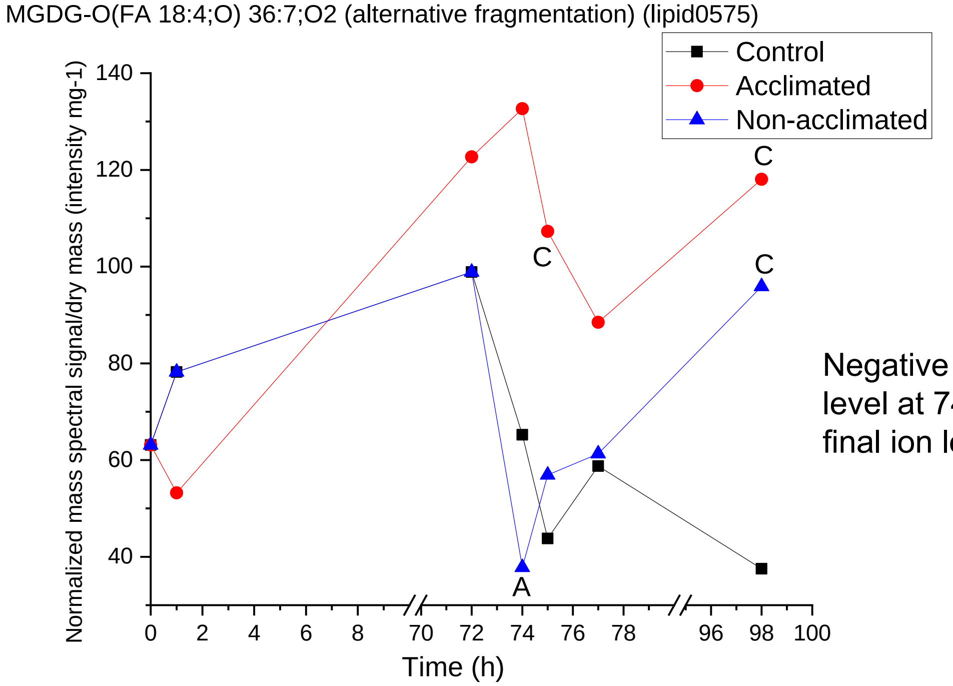

Negative correlation of lipid level at 74, 75, and 77 h with final ion leakage

MGDG-O(FA 18:4;O) 36:8;O2 (alternative fragmentation; Arabidopside G) (lipid0574)

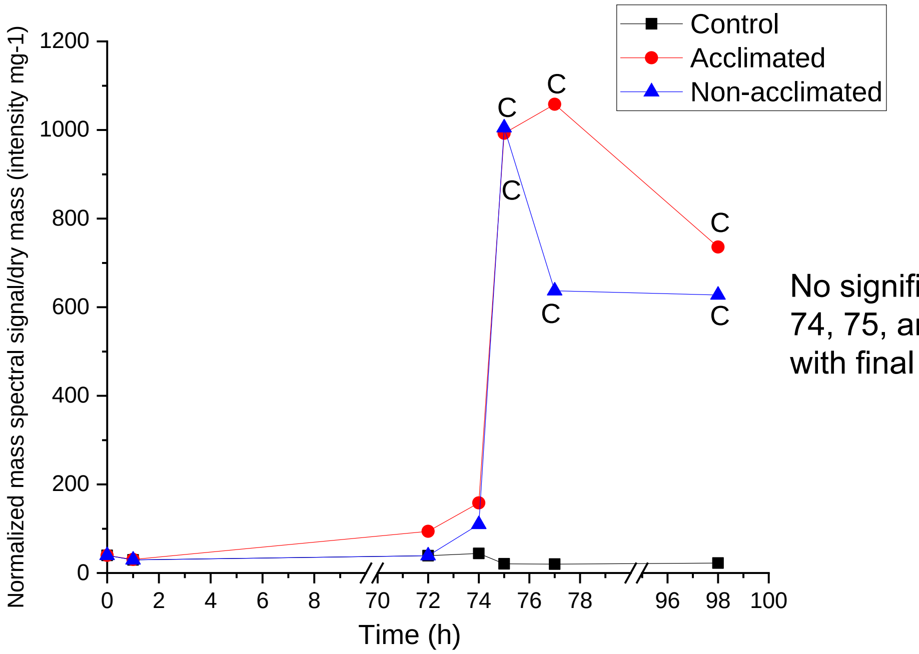

No significant correlation of 74, 75, and 77 h lipid levels with final ion leakage

MGDG-O(FA 18:4;O) 36:8;O2 (Arabidopside G) (lipid0540)

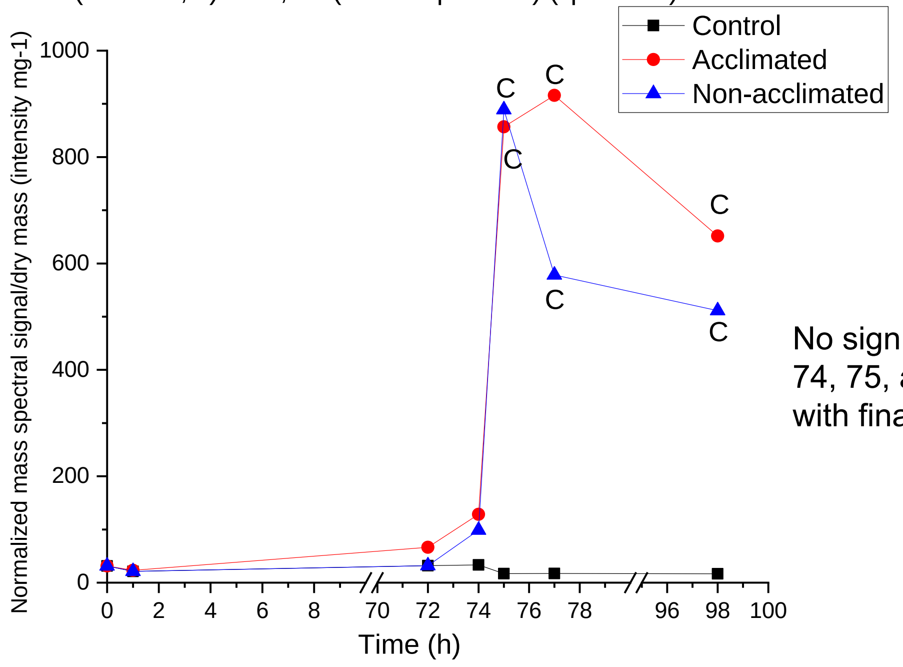

No significant correlation of 74, 75, and 77 h lipid levels with final ion leakage

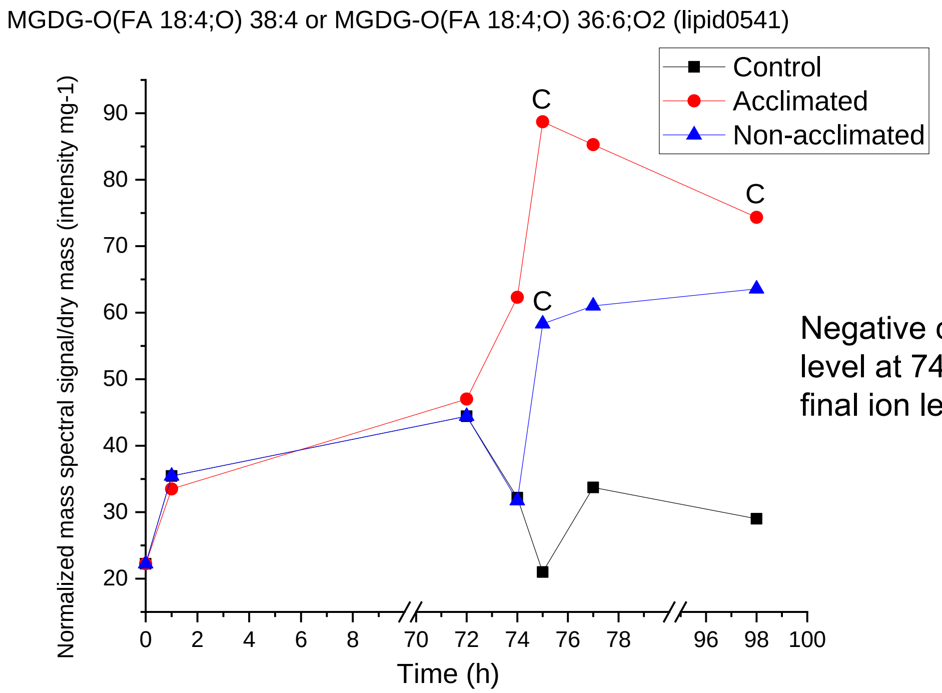

Negative correlation of lipid level at 74, 75, and 77 h with final ion leakage

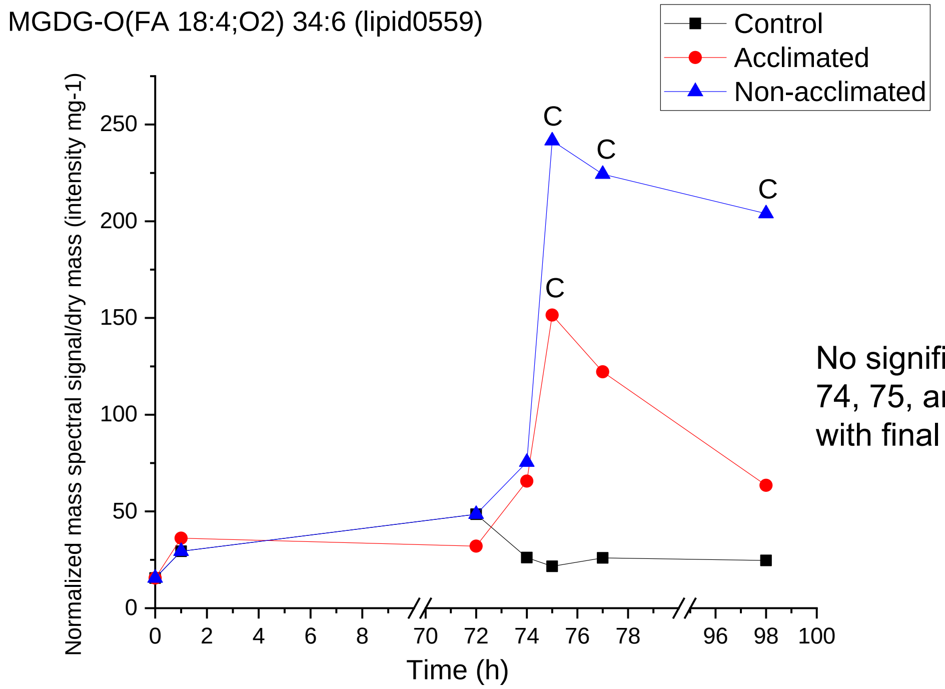

No significant correlation of 74, 75, and 77 h lipid levels with final ion leakage

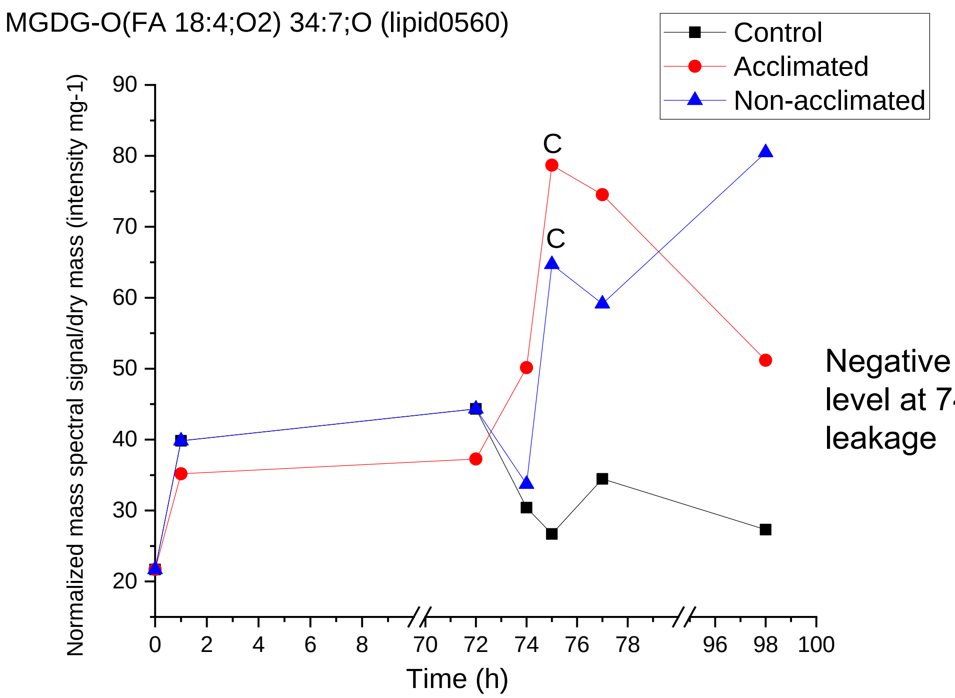

Negative correlation of lipid level at 74 h with final ion leakage

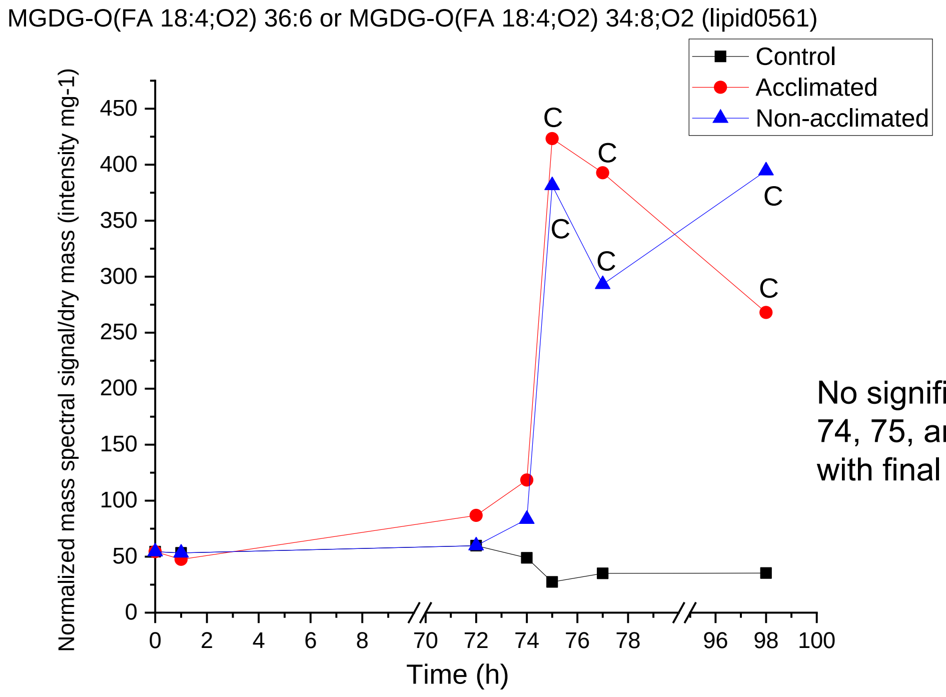

No significant correlation of 74, 75, and 77 h lipid levels with final ion leakage

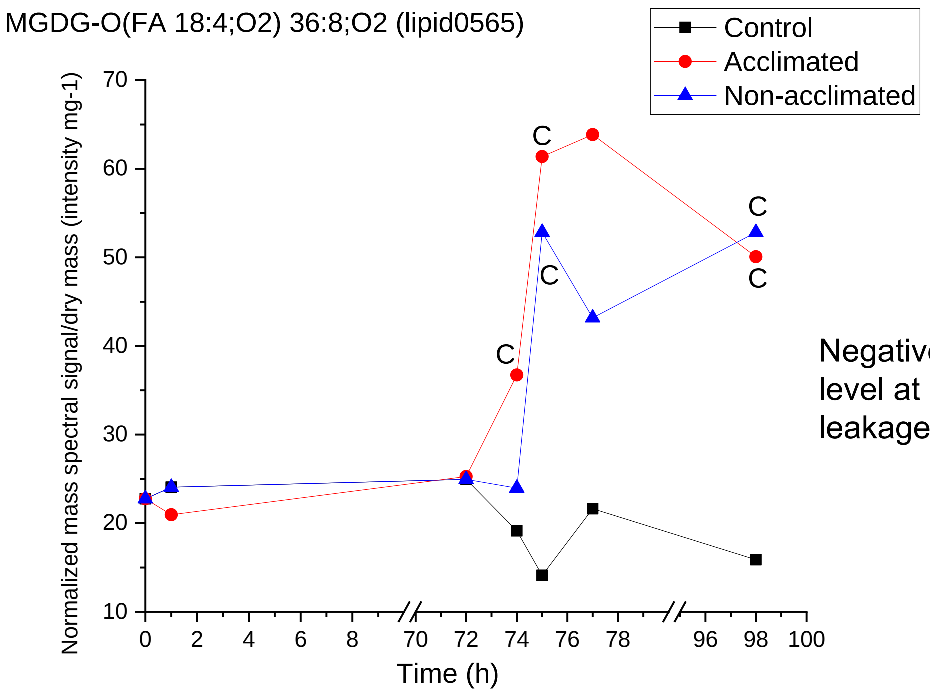

Negative correlation of lipid level at 75 h with final ion leakage

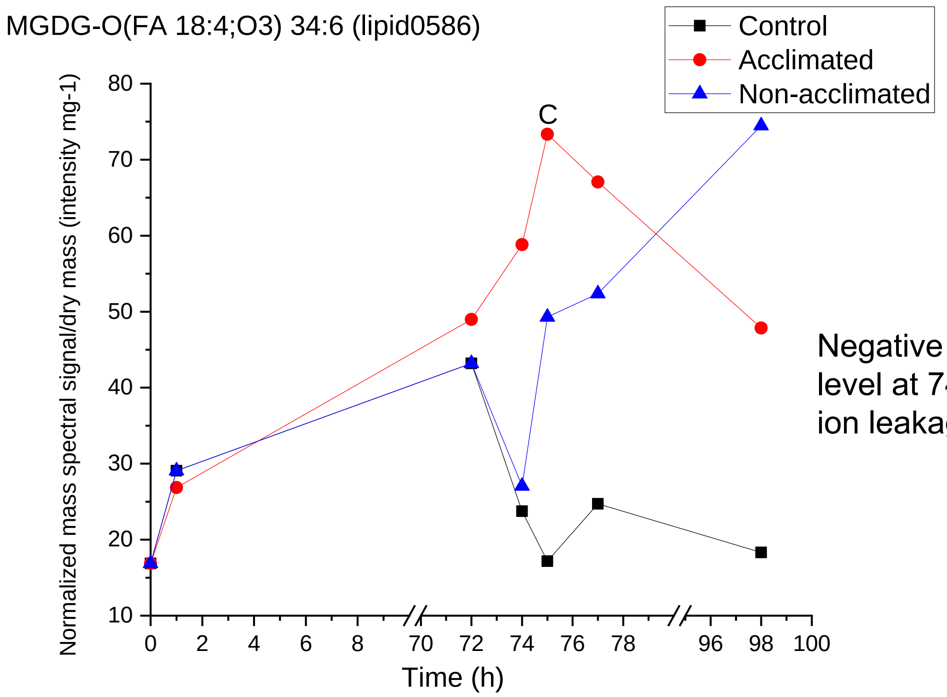

Negative correlation of lipid level at 74 and 75 h with final ion leakage

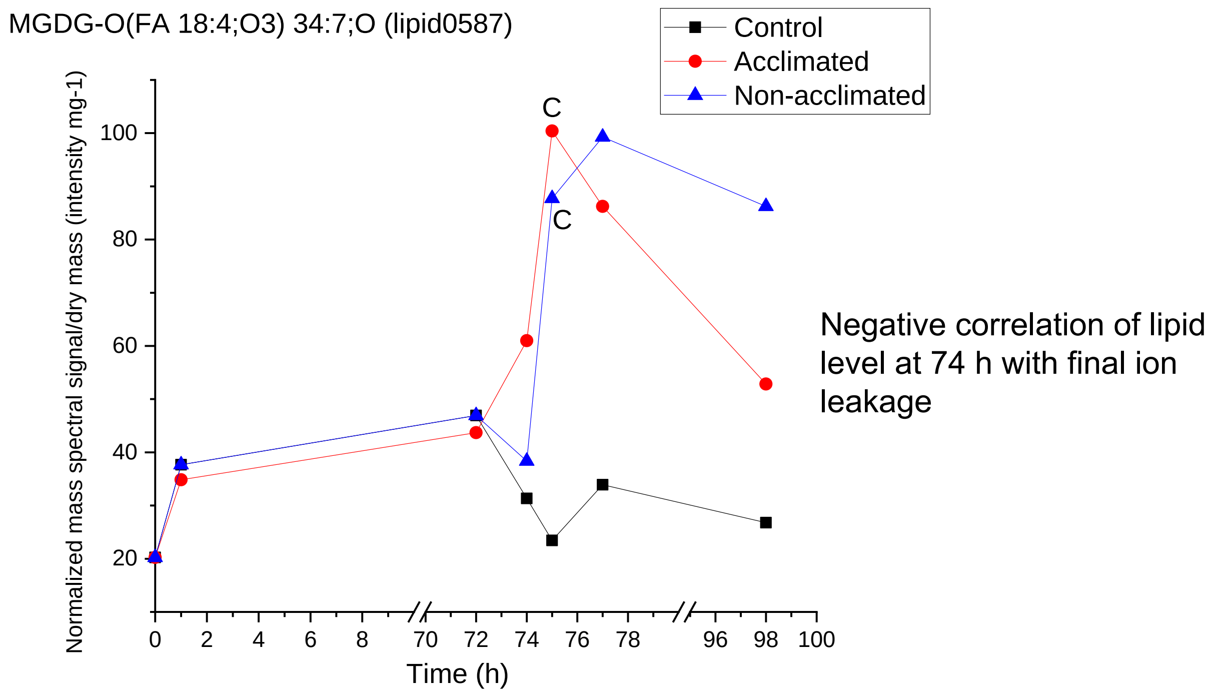
MGDG-O(FA 18:4;O3) 34:7;O (lipid0587)
Control
Acclimated
Non-acclimated
Normalized mass spectral signal/dry mass (intensity mg-1)
Time (h)
C
C
Negative correlation of lipid level at 74 h with final ion leakage

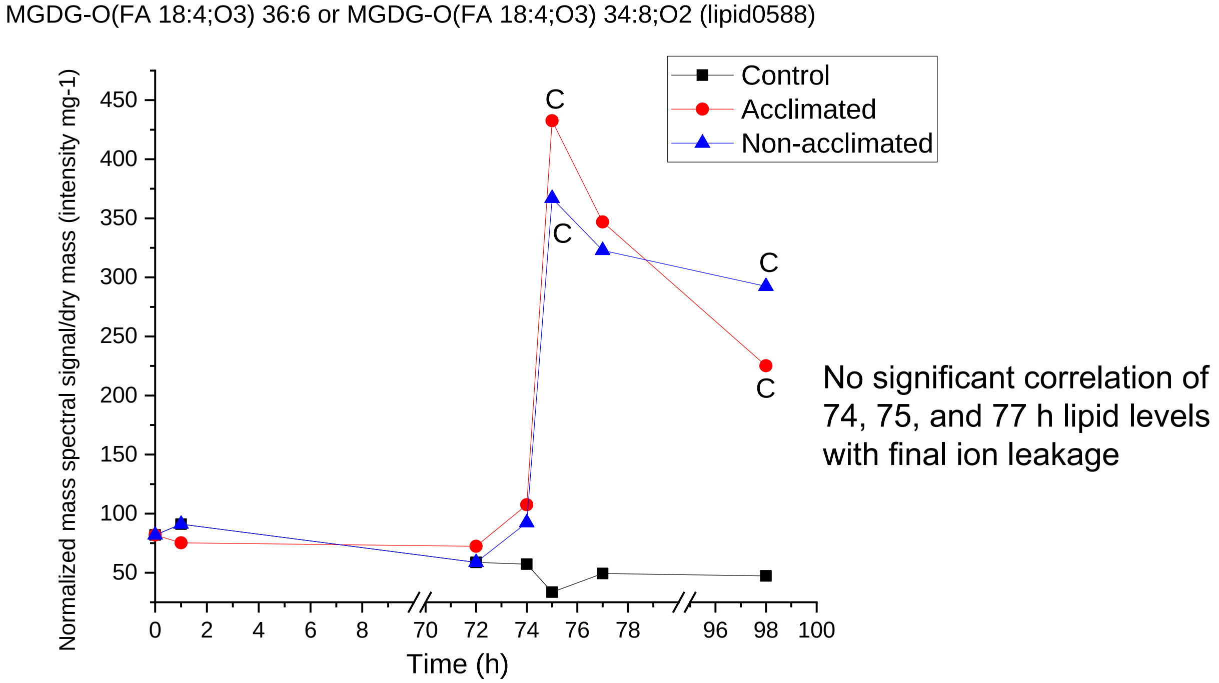
MGDG-O(FA 18:4;O3) 36:6 or MGDG-O(FA 18:4;O3) 34:8;O2 (lipid0588)
Control
Acclimated
Non-acclimated
Normalized mass spectral signal/dry mass (intensity mg-1)
Time (h)
C
C
C
C
No significant correlation of 74, 75, and 77 h lipid levels with final ion leakage

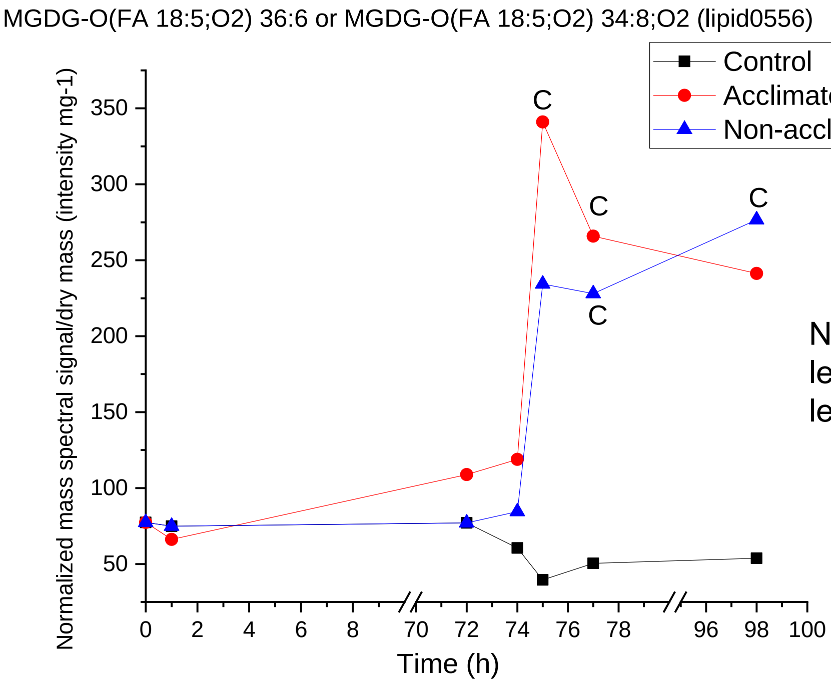

Negative correlation of lipid level at 75 h with final ion leakage

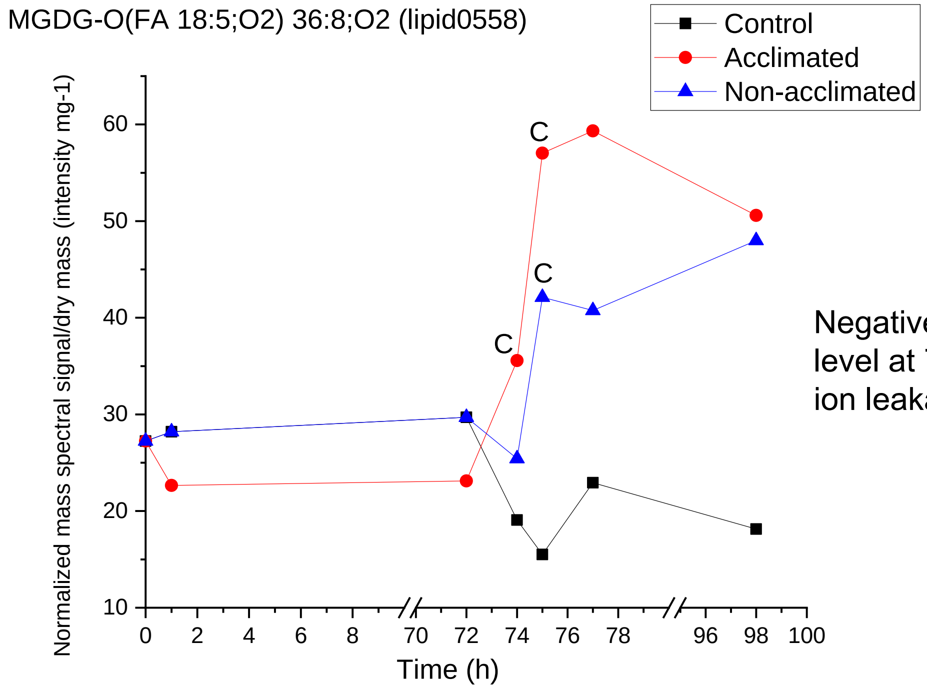

Negative correlation of lipid level at 74 and 75 h with final ion leakage

5BB

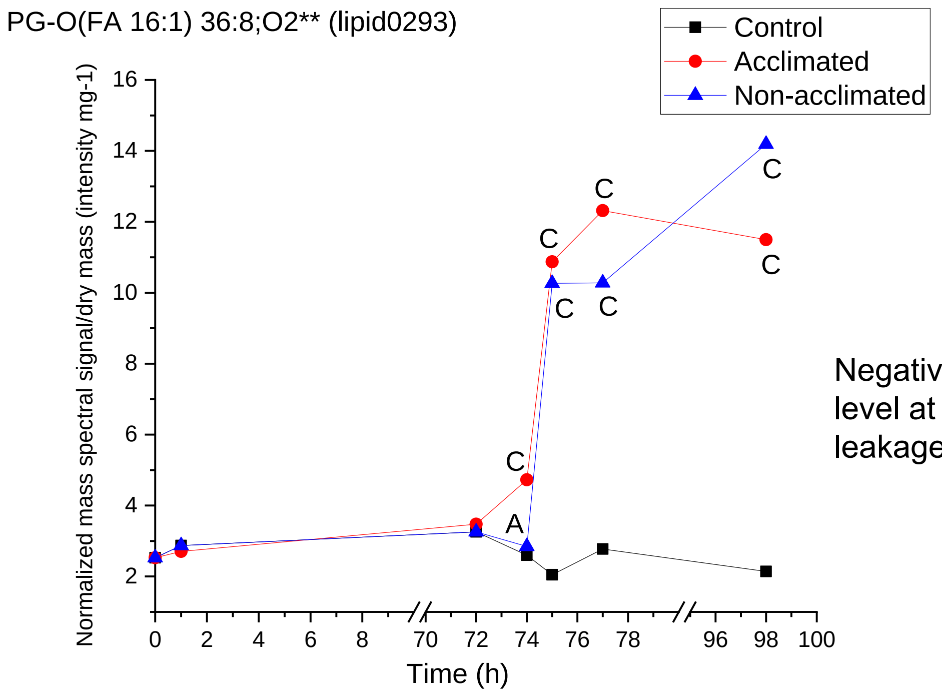

Negative correlation of lipid level at 74 h with final ion leakage

Ambiguous (with respect to oxidation), acylated chloroplast lipids

MGDG-O(FA 16:0) 36:6 or MGDG-O(FA 16:0) 34:8;O2 (lipid0476)

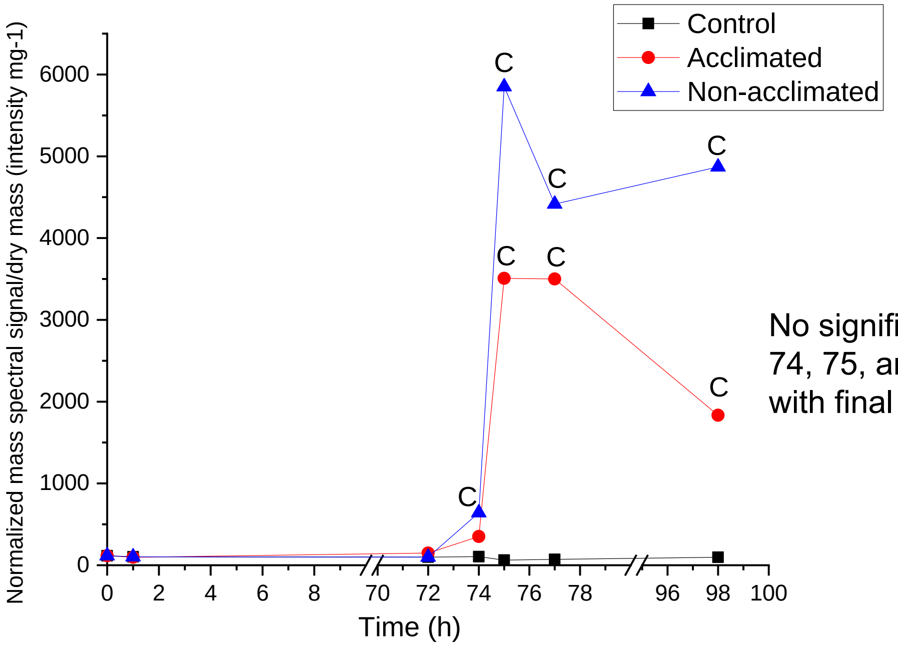

No significant correlation of 74, 75, and 77 h lipid levels with final ion leakage

MGDG-O(FA 16:1) 36:6 or MGDG-O(FA 16:1) 34:8;O2 (lipid0468)

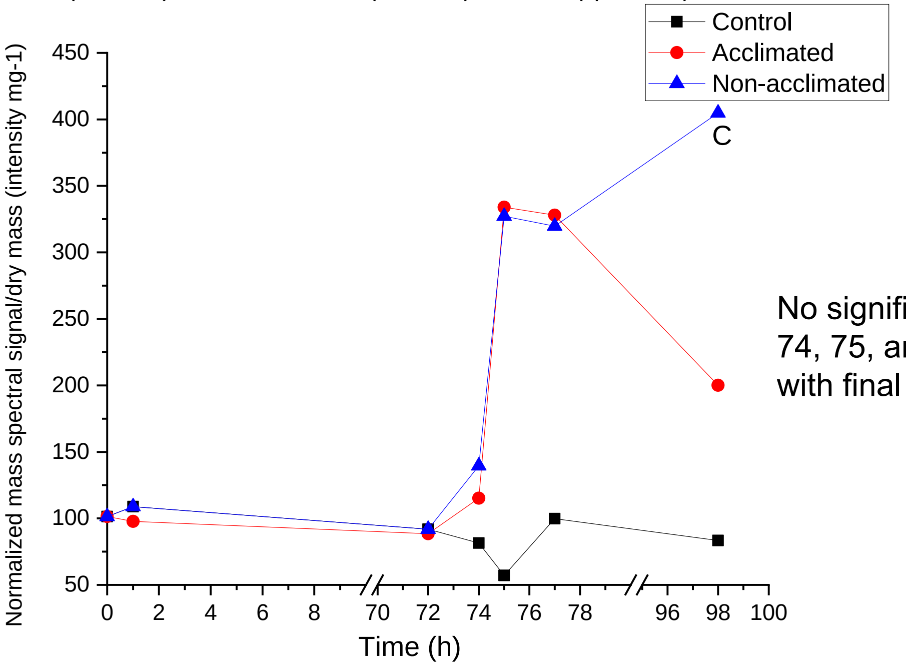

No significant correlation of 74, 75, and 77 h lipid levels with final ion leakage

MGDG-O(FA 16:2) 36:6 or MGDG-O(FA 16:2) 34:8;O2 (lipid0461)

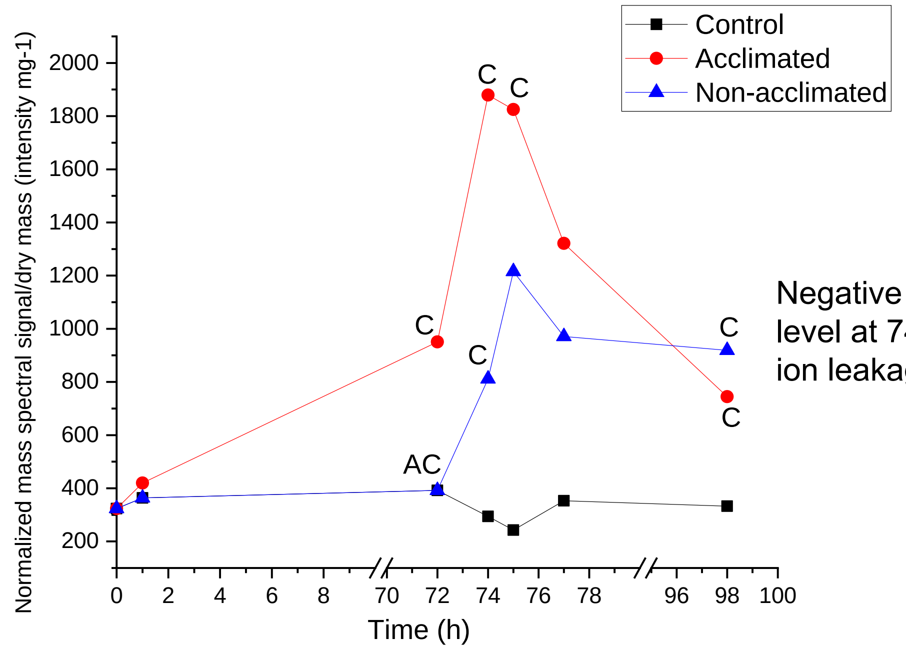

Negative correlation of lipid level at 74 and 75 h with final ion leakage

MGDG-O(FA 16:3) 36:6 or MGDG-O(FA 16:3) 34:8;O2 (lipid0454)

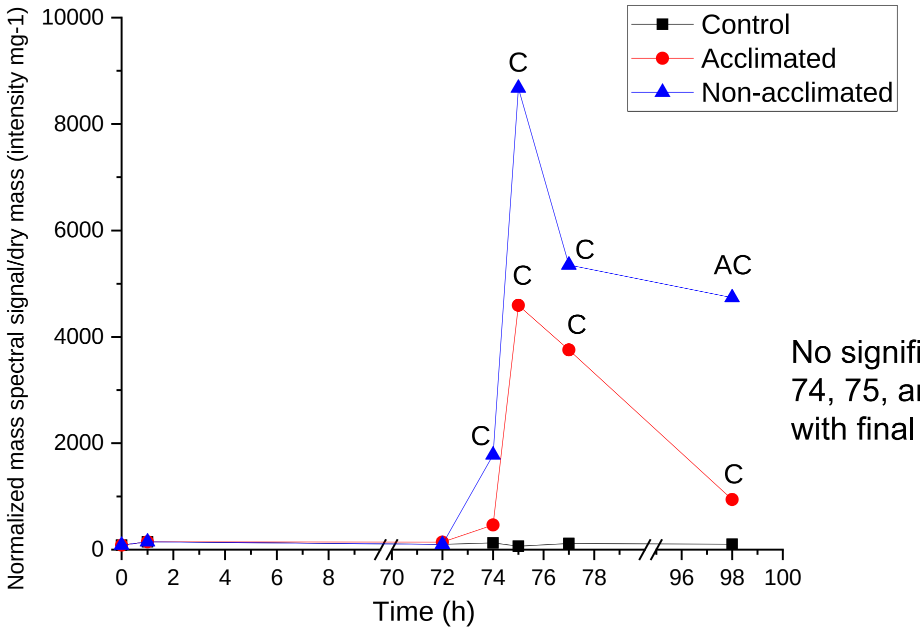

No significant correlation of 74, 75, and 77 h lipid levels with final ion leakage

MGDG-O(FA 16:3;O2) 34:6 or MGDG-O(FA 18:1) 34:6 (lipid0519)

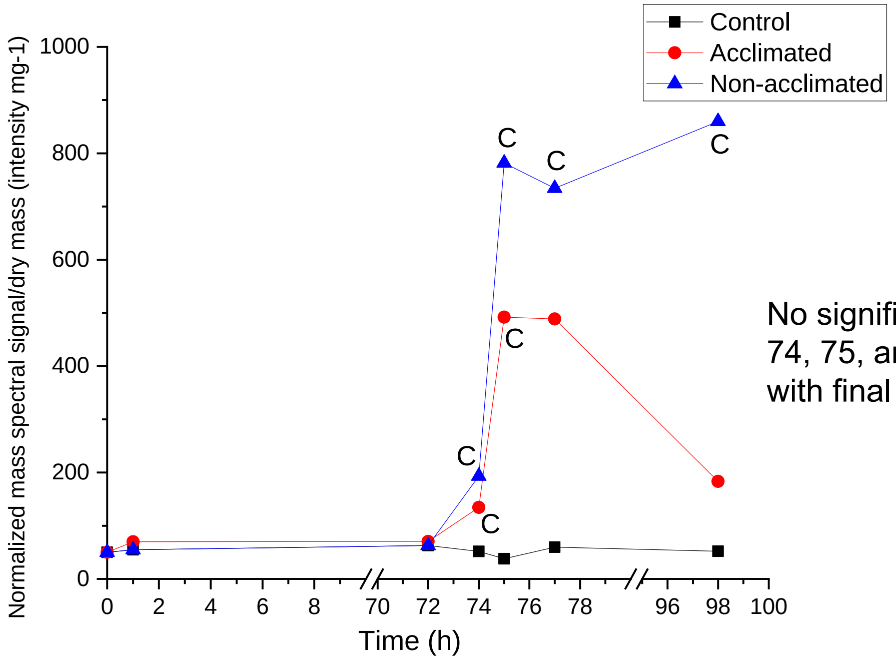

No significant correlation of 74, 75, and 77 h lipid levels with final ion leakage

MGDG-O(FA 16:3;O2) 36:6 or MGDG-O(FA 16:3;O2) 34:8;O2
or MGDG-O(FA 18:1) 36:6 or MGDG-O(FA 18:1) 34:8;O2 (lipid0522)

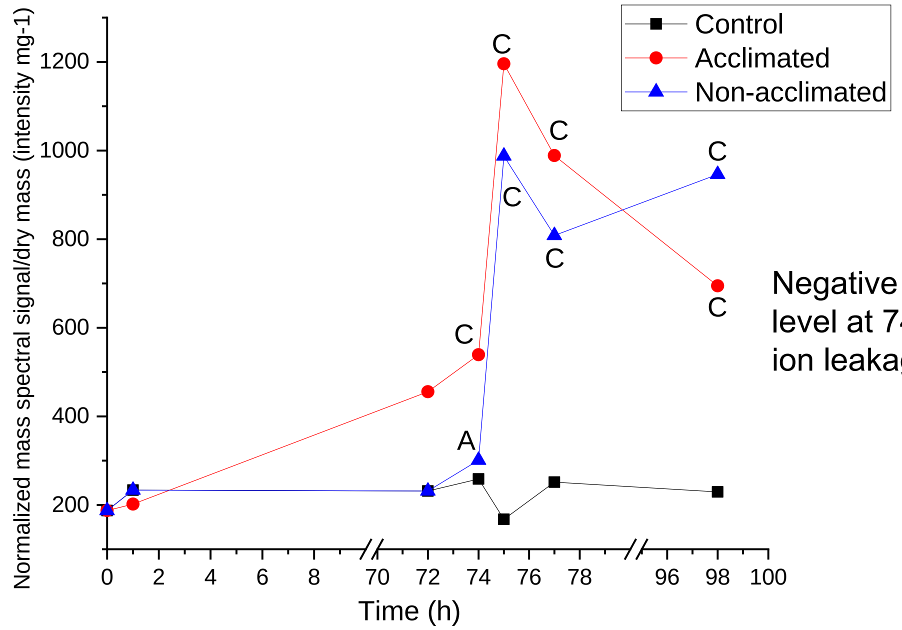

Negative correlation of lipid level at 74 and 75 h with final ion leakage

MGDG-O(FA 16:3;O2) 36:8;O2 or MGDG-O(FA 18:1) 36:8;O2 (lipid0525)

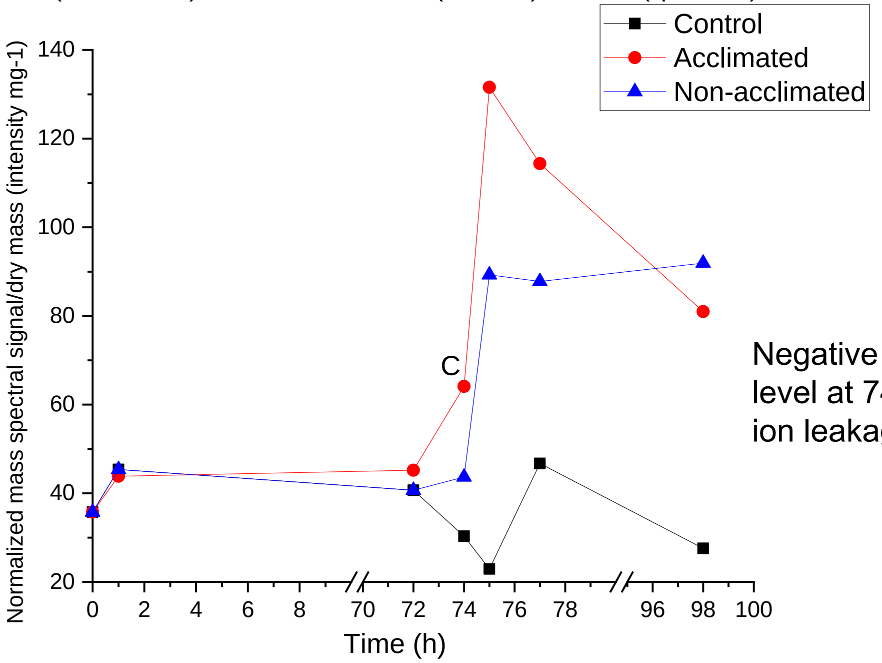

Negative correlation of lipid level at 74 and 75 h with final ion leakage

MGDG-O(FA 18:0) 34:8;O2 or MGDG-O(FA 18:0) 36:6 (lipid0528)

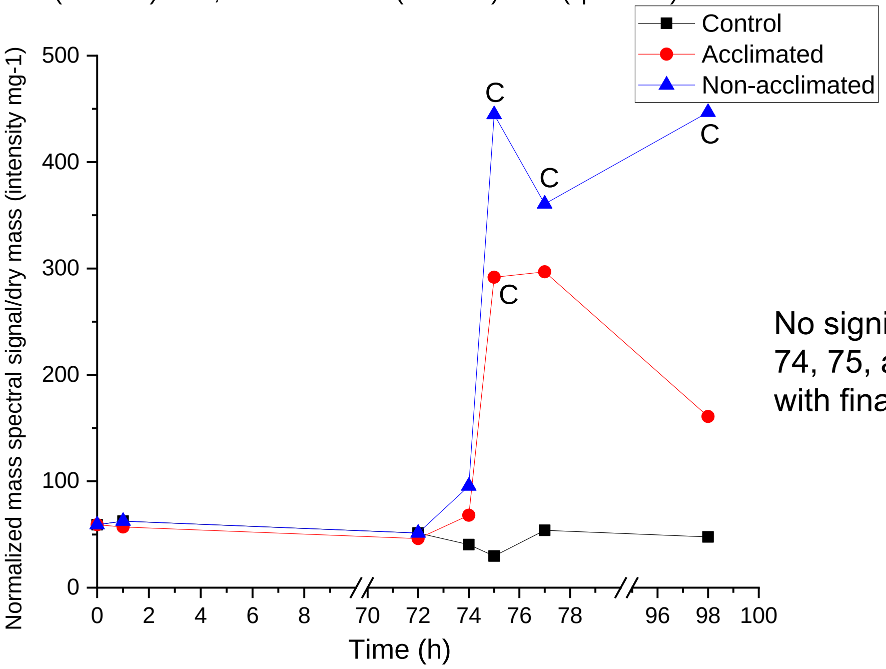

No significant correlation of 74, 75, and 77 h lipid levels with final ion leakage

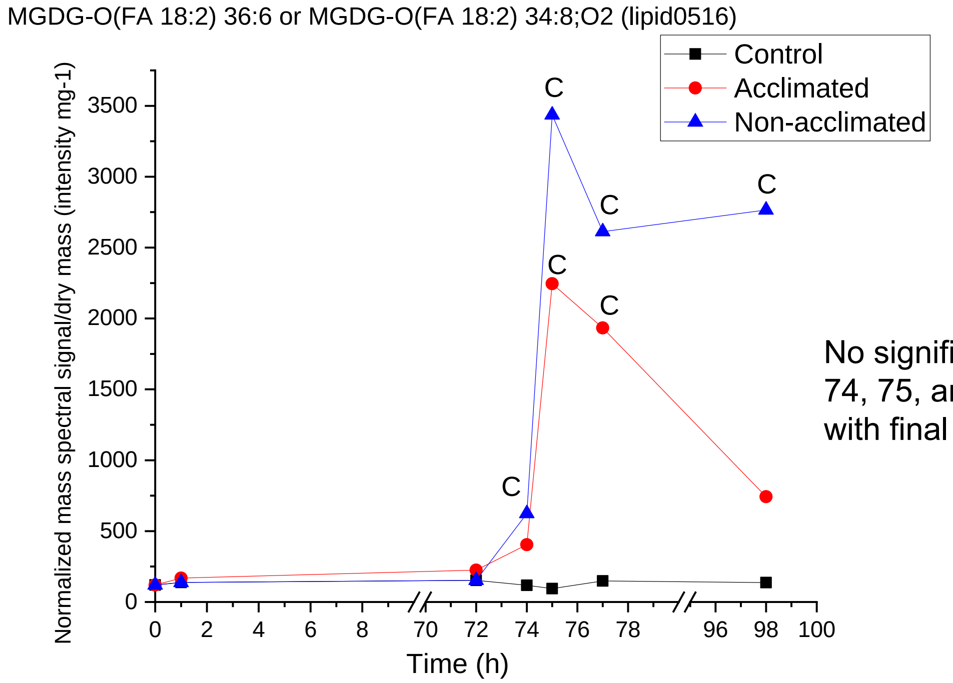

No significant correlation of 74, 75, and 77 h lipid levels with final ion leakage

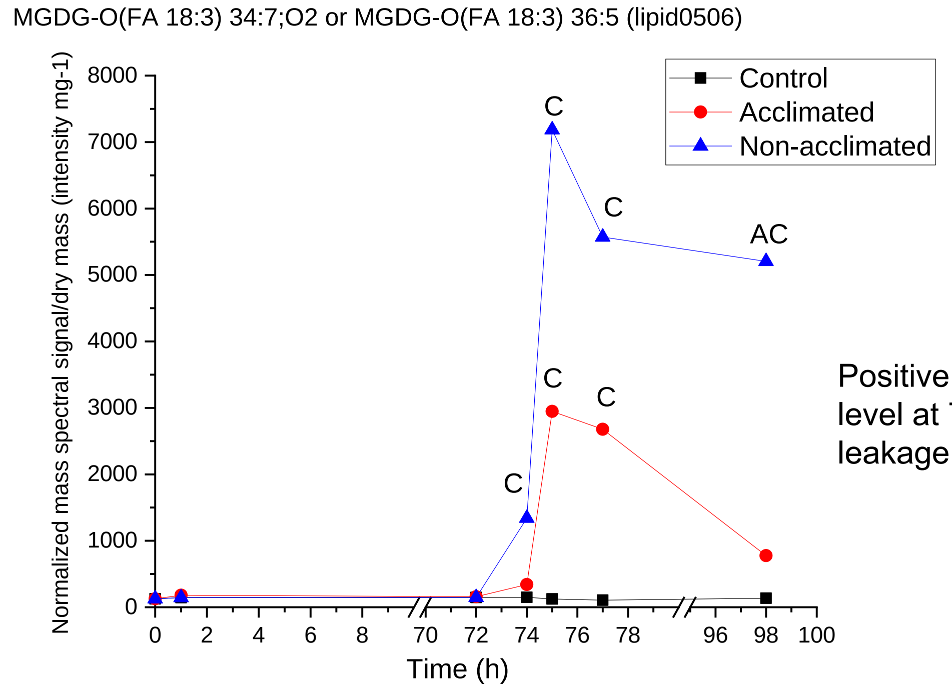

Positive correlation of lipid level at 74 h with final ion leakage

MGDG-O(FA 18:3) 36:6 or MGDG-O(FA 18:3) 34:8;O2 (lipid0505)

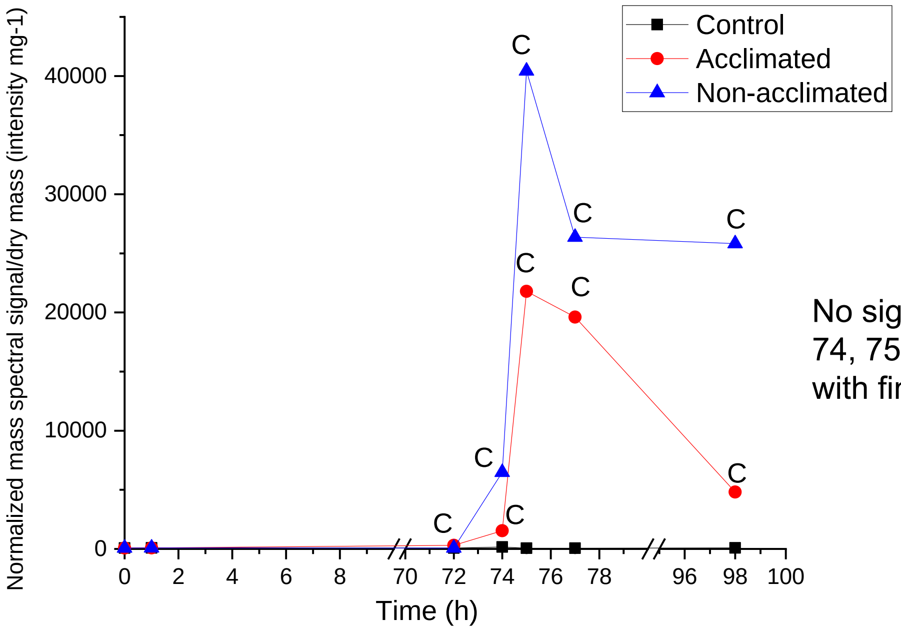

No significant correlation of 74, 75, and 77 h lipid levels with final ion leakage

Figure S6. Time courses of levels of selected oxidized polar lipids in rosettes of control, non-acclimated, and acclimated plants. Treatments are shown in Figure 1. Asterisks indicate lipids with quality control (pooled sample) levels less than 0.75 (*) or 0.25 (**) units of normalized mass spectral intensity, where 1 = intensity of 1 pmol of internal standard. "C" indicates that the lipid level in non-acclimated or acclimated plants is significantly different than the control level, and "A" indicates that the lipid level in non-acclimated plants is significantly different than the level in acclimated plants (Table S2). Indicated on each plot is whether there is significant correlation of lipid level at the 74-, 75-, and 77-h time points with final (98 h) ion leakage.

**Lipid number**	**Panel**	**Class, oxidation**	**Lipid name**
lipid0632	6A	DGDG, oxidized	DGDG 16:0_18:3;O*
lipid0392	6A	DGDG, oxidized	DGDG 16:0_18:4;O*
lipid0339	6B	DGDG, oxidized	DGDG 18:3;O_16:4;O**
lipid0394	6B	DGDG, oxidized	DGDG 18:3;O2_16:3*
lipid0400	6C	DGDG, oxidized	DGDG 18:3;O2_18:3 or DGDG 18:4;O_18:2;O**
lipid0336	6C	DGDG, oxidized	DGDG 18:3_16:4;O*
lipid0354	6D	DGDG, oxidized	DGDG 18:3_18:5;O2
lipid0387	6D	DGDG, oxidized	DGDG 18:4;O_16:3*
lipid0390	6E	DGDG, oxidized	DGDG 18:4;O_16:3;O*
lipid0396	6E	DGDG, oxidized	DGDG 18:4;O_18:3
lipid0399	6F	DGDG, oxidized	DGDG 18:4;O_18:3;O**
lipid0398	6F	DGDG, oxidized	DGDG 18:4;O_18:4;O (Arabidopside D)*
lipid0402	6G	DGDG, oxidized	DGDG 18:4;O_18:4;O2 or DGDG 18:4;O_20:2*
lipid0175	6G	DGDG, ambiguous	DGDG 38:4 or DGDG 36:6;O2
lipid0173	6H	DGDG, ambiguous	DGDG 38:6 or DGDG 36:8;O2
lipid0435	6H	MGDG, oxidized	MGDG 18:2;O_16:3*
lipid0623	6I	MGDG, oxidized	MGDG 18:3;O_16:3*
lipid0370	6I	MGDG, oxidized	MGDG 18:3;O2_16:3*
lipid0383	6J	MGDG, oxidized	MGDG 18:3;O2_18:3;O**
lipid0318	6J	MGDG, oxidized	MGDG 18:3;O3_16:3**
lipid0637	6K	MGDG, oxidized	MGDG 18:3_16:3;O*
lipid0327	6K	MGDG, oxidized	MGDG 18:3_16:3;O2*

lipid0323	6L	MGDG, oxidized	MGDG 18:3_16:4;O
lipid0837	6L	MGDG, oxidized	MGDG 18:3_7:1;O*
lipid0358	6M	MGDG, oxidized	MGDG 18:4;O_16:3
lipid0362	6M	MGDG, oxidized	MGDG 18:4;O_16:3;O*
lipid0325	6N	MGDG, oxidized	MGDG 18:4;O_16:4;O (16:4;O as fragment; Arabidopside A)
lipid0361	6N	MGDG, oxidized	MGDG 18:4;O_16:4;O (18:4;O as fragment; Arabidopside A)
lipid0372	6O	MGDG, oxidized	MGDG 18:4;O_18:3
lipid0385	6O	MGDG, oxidized	MGDG 18:4;O_18:3;O3**
lipid0386	6P	MGDG, oxidized	MGDG 18:4;O_18:3;O4**
lipid0375	6P	MGDG, oxidized	MGDG 18:4;O_18:4;O (Arabidopside B)*
lipid0384	6Q	MGDG, oxidized	MGDG 18:4;O_18:4;O3 or MGDG 18:4;O_19:3;O2**
lipid0317	6Q	MGDG, oxidized	MGDG 18:4;O3_16:3**
lipid0138	6R	MGDG, oxidized	MGDG 30:6;O
lipid0137	6R	MGDG, oxidized	MGDG 30:6;O2
lipid0831	6S	MGDG, oxidized	MGDG 9:1;O_16:3*
lipid0834	6S	MGDG, oxidized	MGDG 9:1;O2_16:3*
lipid0154	6T	MGDG, ambiguous	MGDG 38:4 or MGDG 36:6;O2
lipid0152	6T	MGDG, ambiguous	MGDG 38:6 or MGDG 36:8;O2
lipid0357	6U	PG, oxidized	PG 18:4;O_16:0
lipid0356	6U	PG, oxidized	PG 18:4;O_16:1
lipid0364	6V	PG, oxidized	PG 18:4;O_18:2*
lipid0363	6V	PG, oxidized	PG 18:4;O_18:3*
lipid0022	6W	PG, oxidized	PG 34:3;O
lipid0021	6W	PG, oxidized	PG 34:4;O
lipid0628	6X	PC, oxidized	PC 18:2_18:3;O*
lipid0379	6X	PC, oxidized	PC 18:2_18:3;O2*
lipid0421	6Y	PC, oxidized	PC 18:3;O3_18:2*
lipid0378	6Y	PC, oxidized	PC 18:3_18:3;O2*
lipid0604	6Z	PE, oxidized	PE 16:0_18:4;O2*
lipid0429	6Z	PE, oxidized	PE 18:2;O_18:3

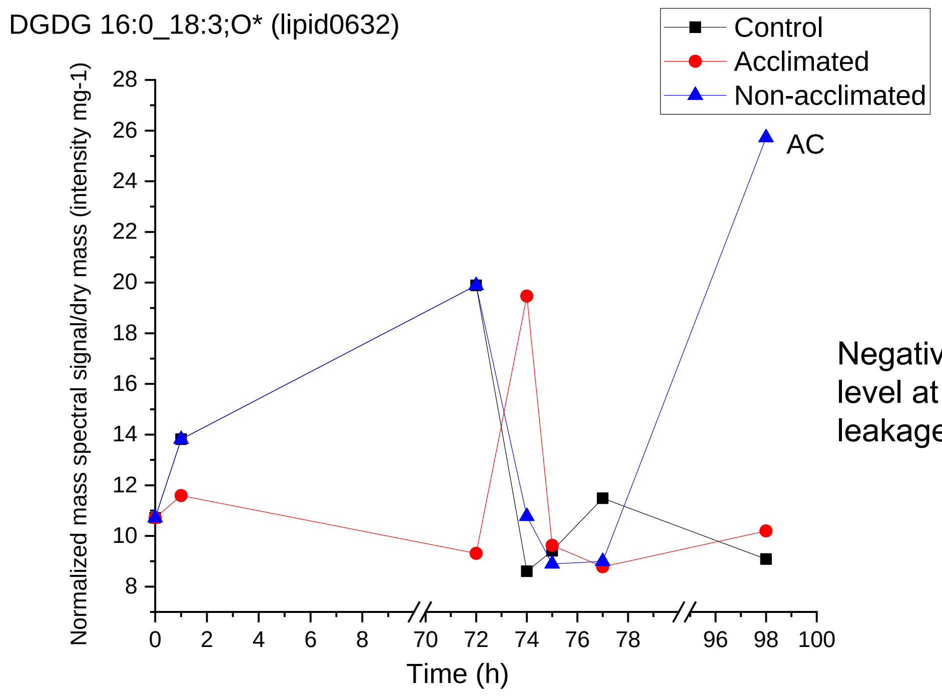

Negative correlation of lipid level at 74 h with final ion leakage

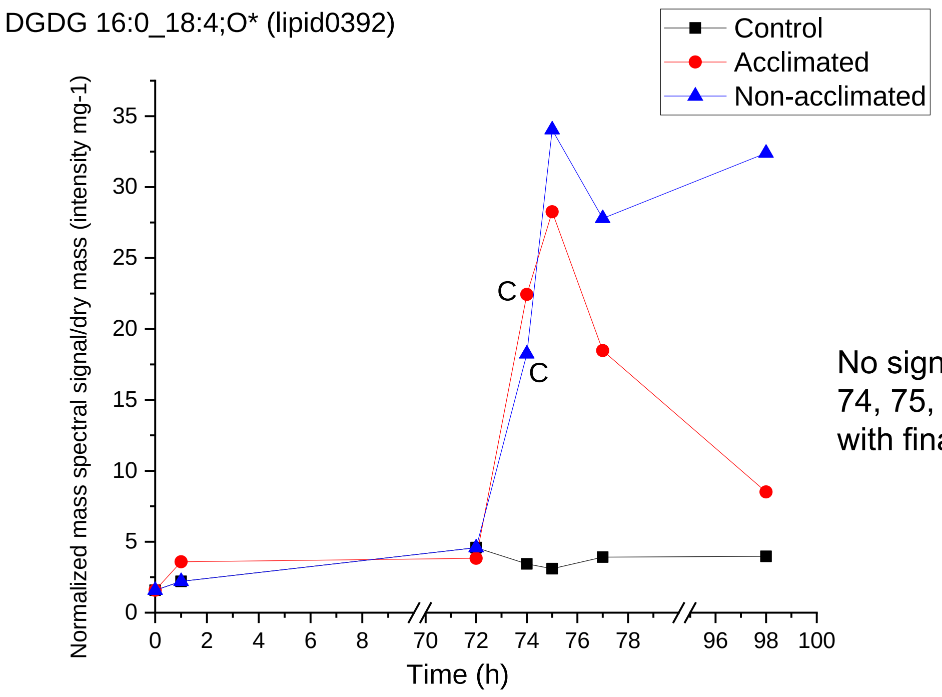

No significant correlation of 74, 75, and 77 h lipid levels with final ion leakage

6A

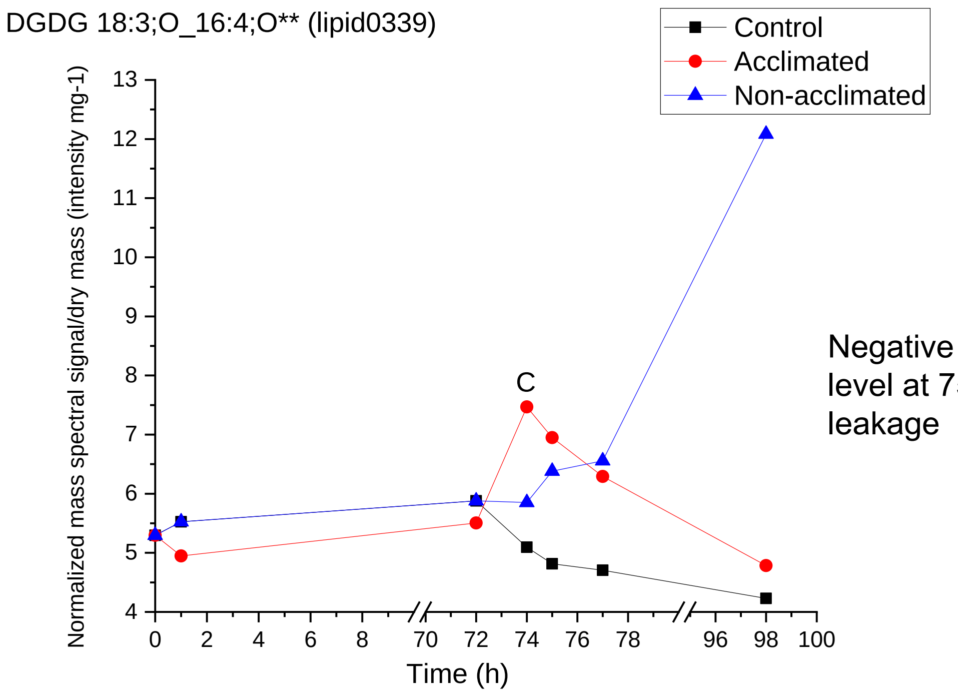

Negative correlation of lipid level at 75 h with final ion leakage

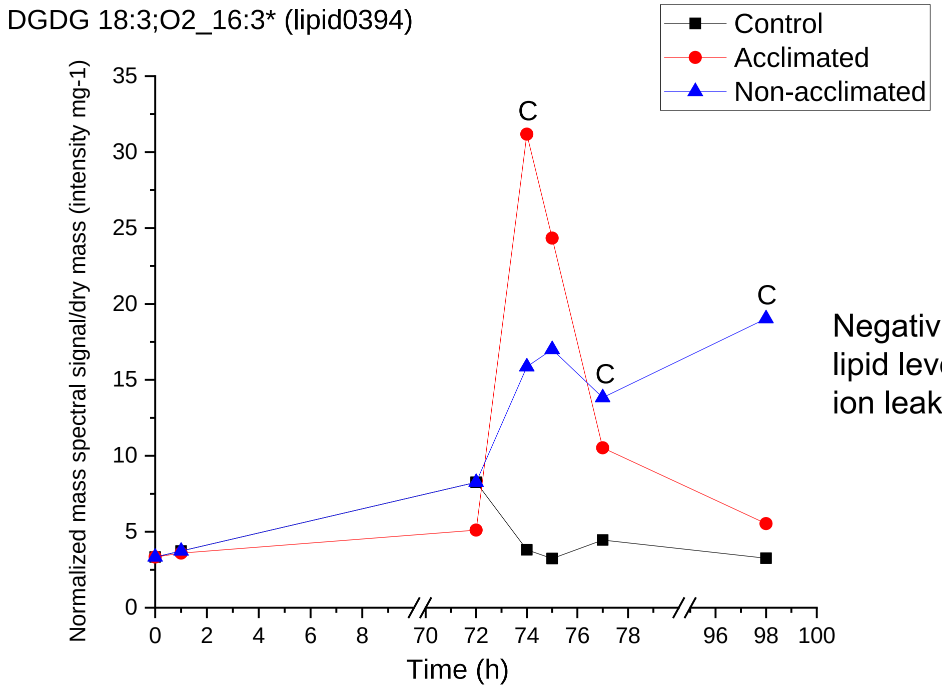

Negative correlation of lipid level at 74 h with final ion leakage

6B

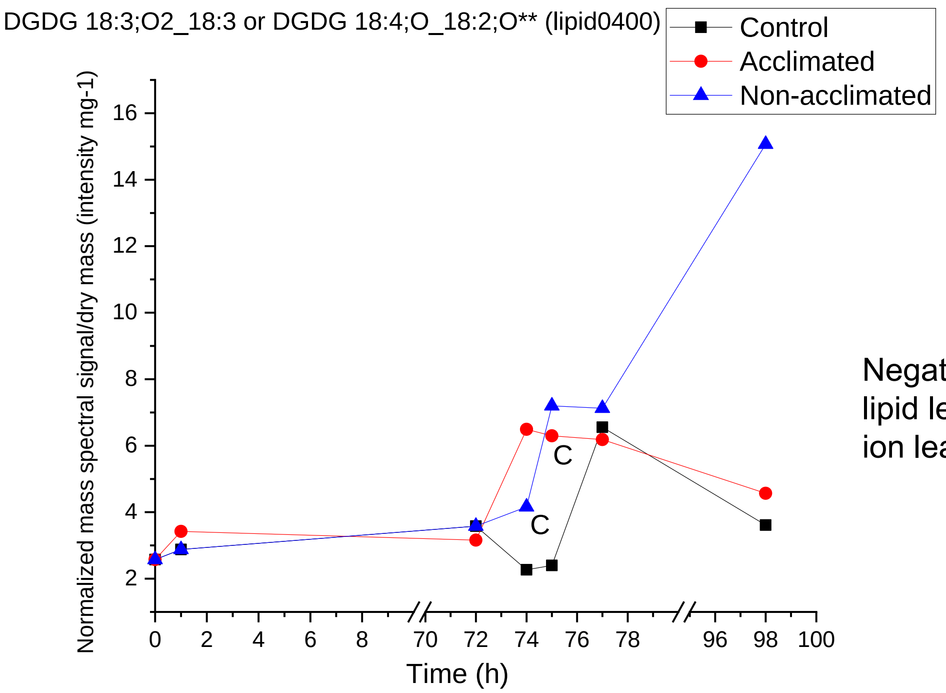

Negative correlation of lipid level at 74 h with final ion leakage

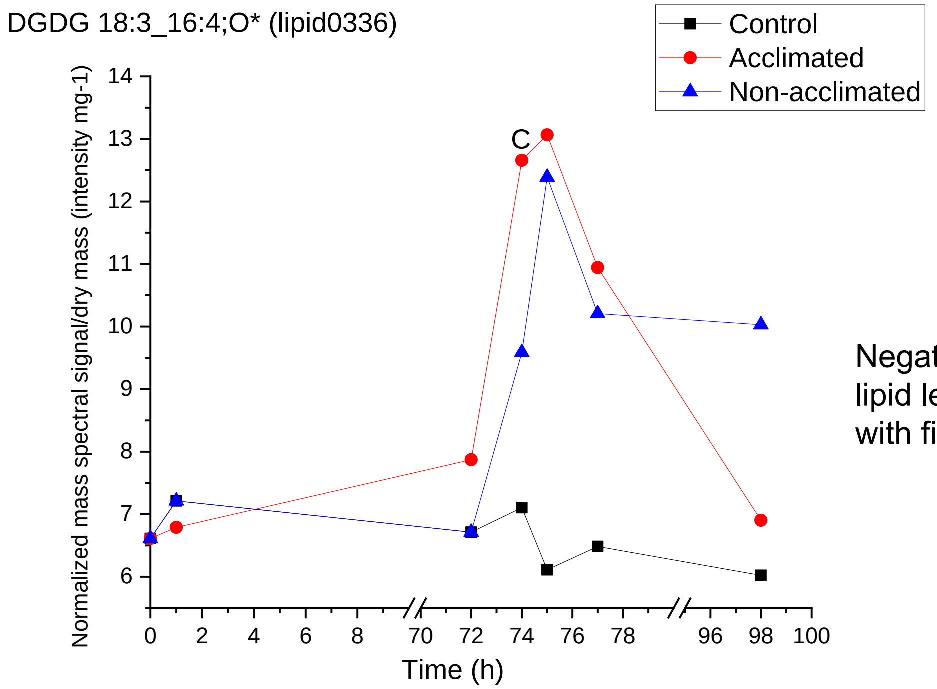

Negative correlation of lipid level at 74 and 75 h with final ion leakage

6C

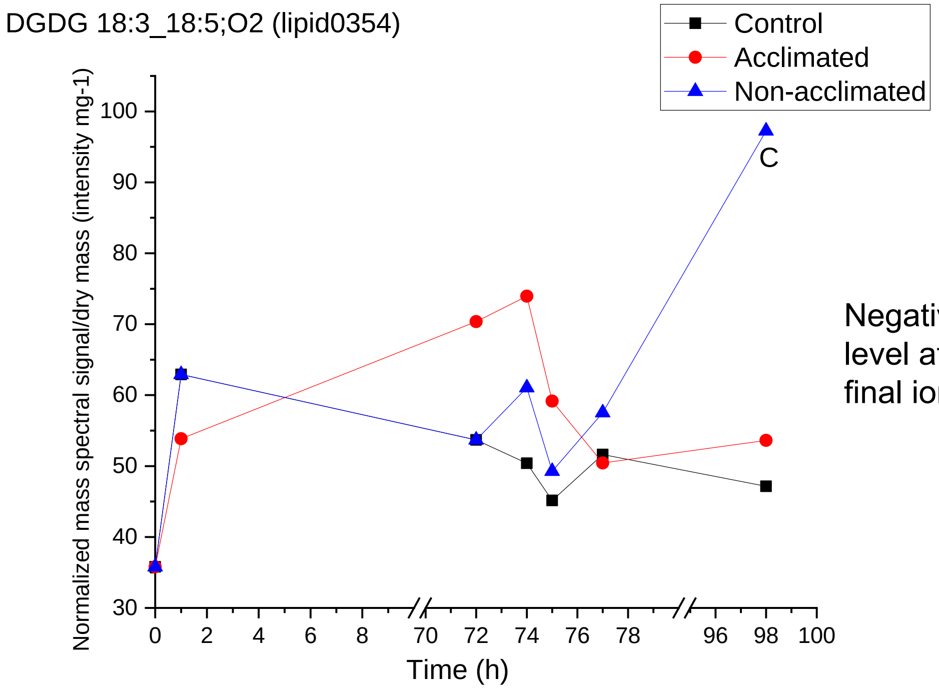

Negative correlation of lipid level at 74 and 75 h with final ion leakage

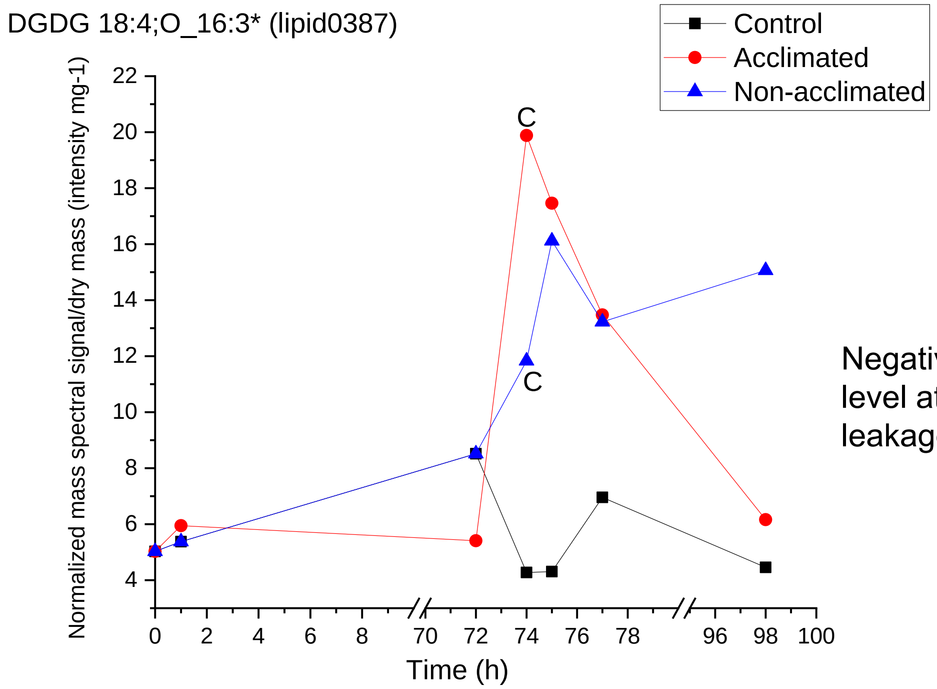

Negative correlation of lipid level at 74 h with final ion leakage

6D

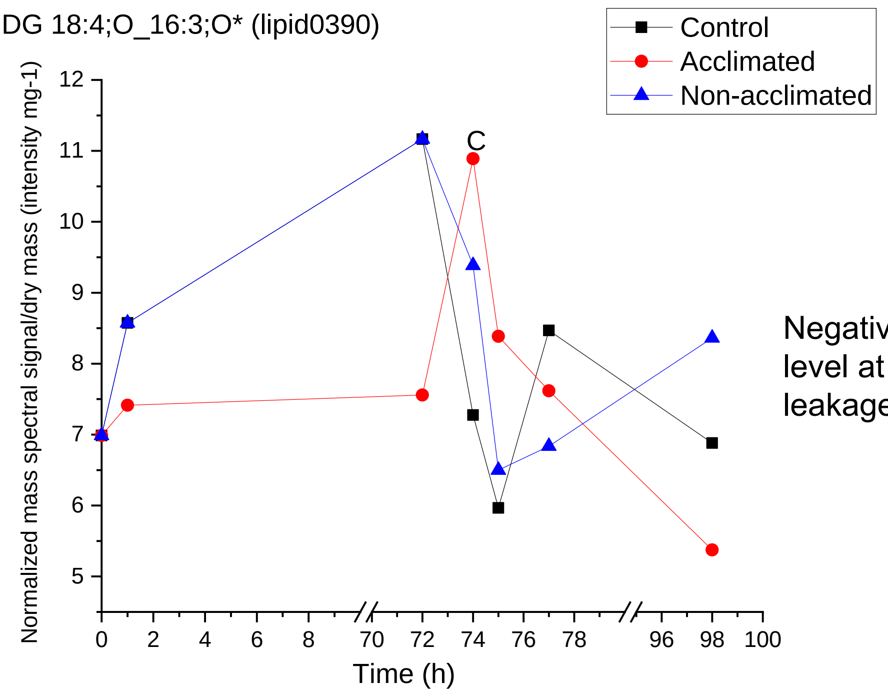

Negative correlation of lipid level at 75 h with final ion leakage

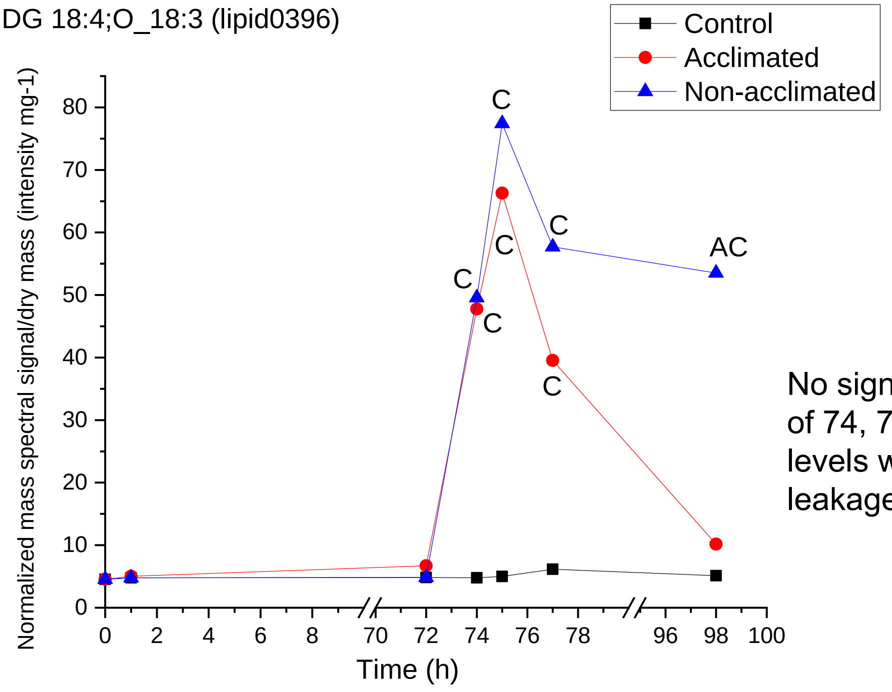

No significant correlation of 74, 75, and 77 h lipid levels with final ion leakage

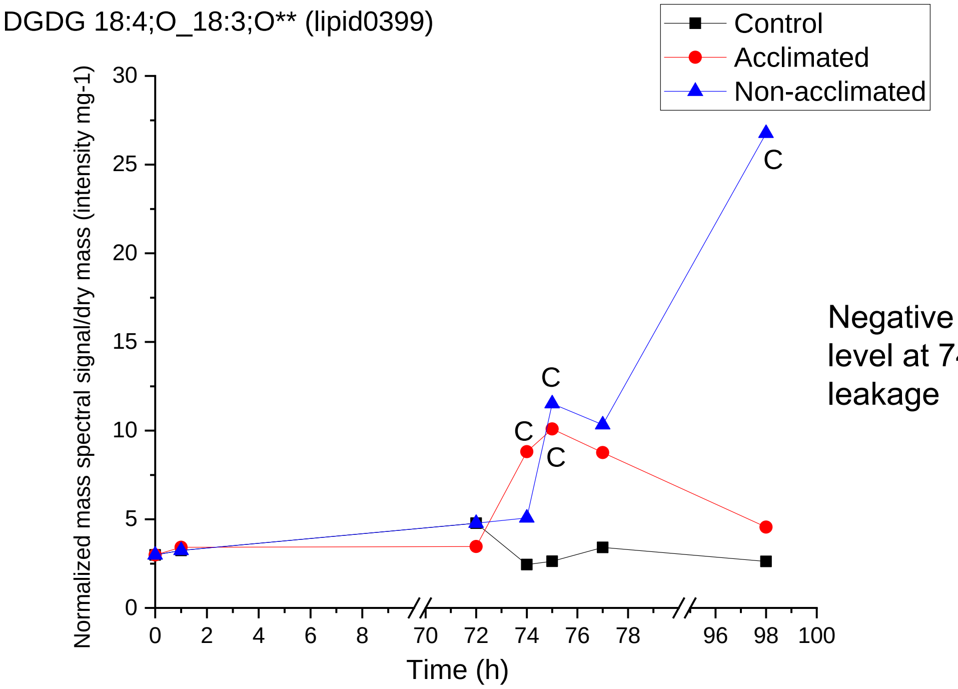

Negative correlation of lipid level at 74 h with final ion leakage

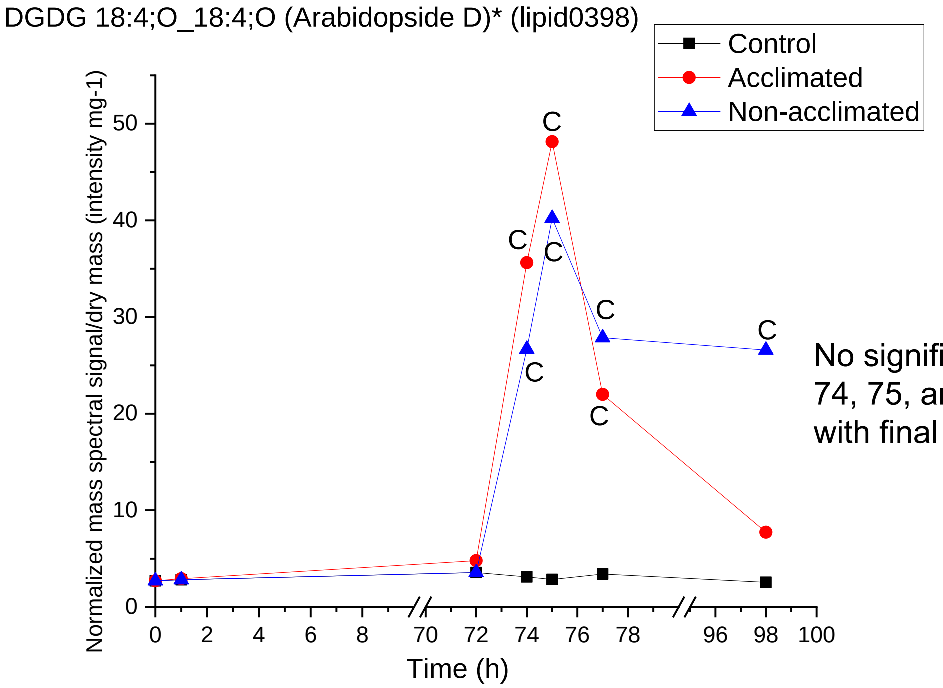

No significant correlation of 74, 75, and 77 h lipid levels with final ion leakage

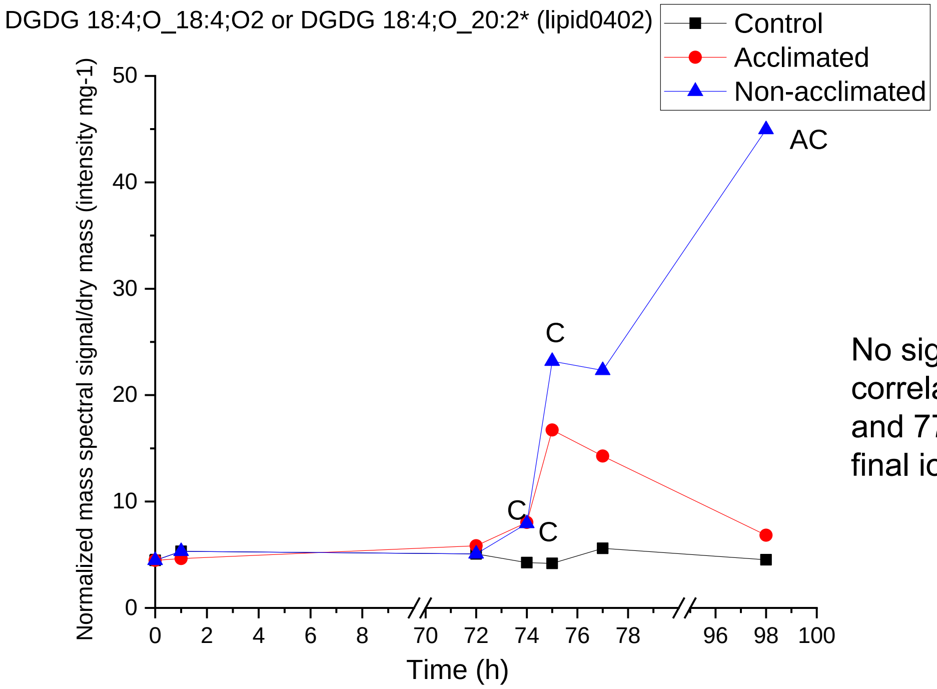

No significant correlation of 74-, 75-, and 77-h lipid levels with final ion leakage

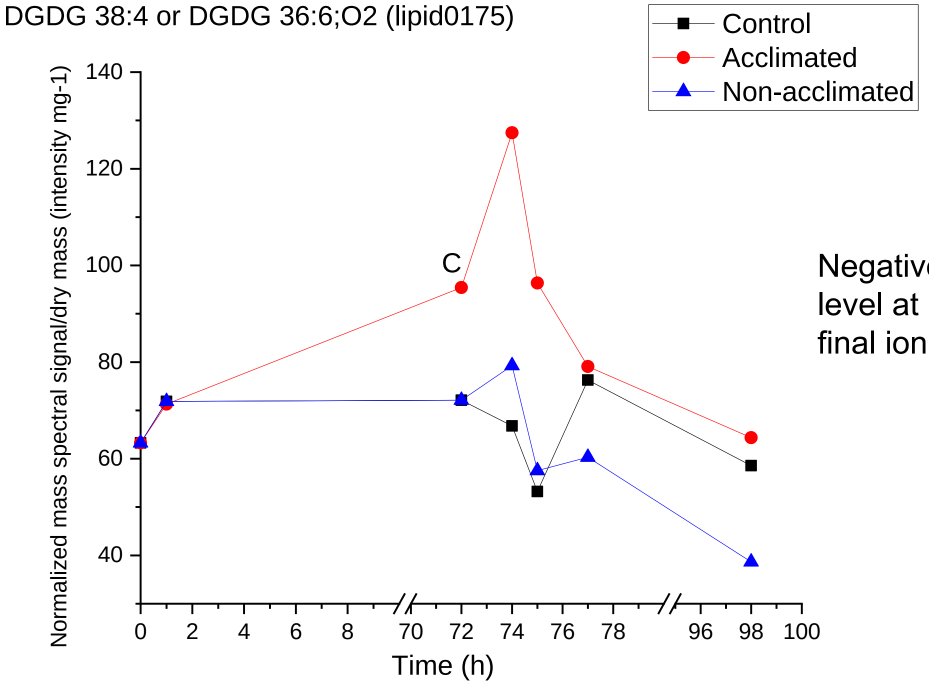

Negative correlation of lipid level at 74 and 75 h with final ion leakage

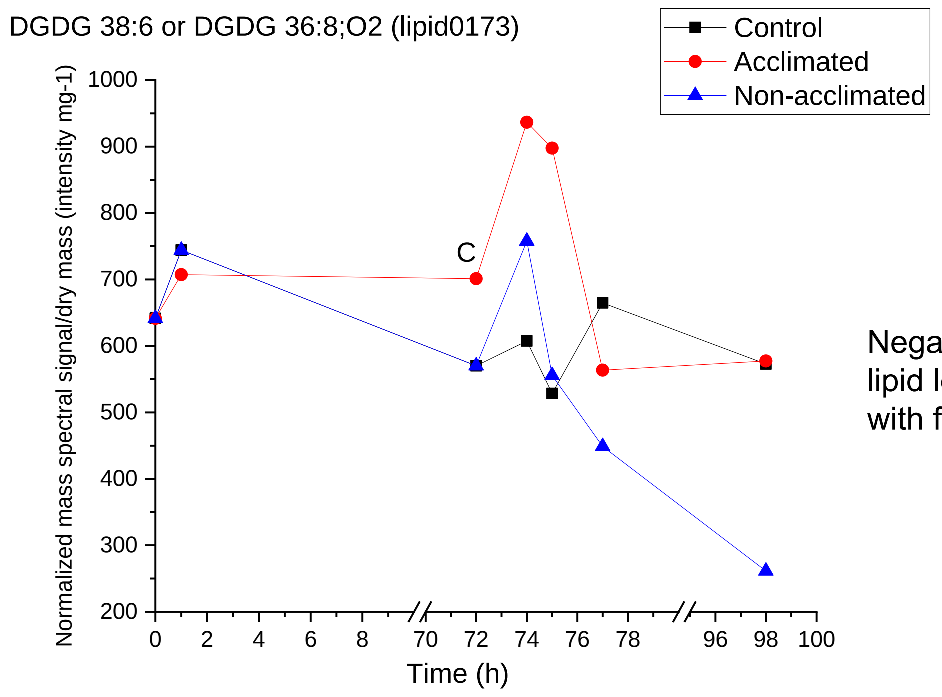

Negative correlation of lipid level at 74 and 75 h with final ion leakage

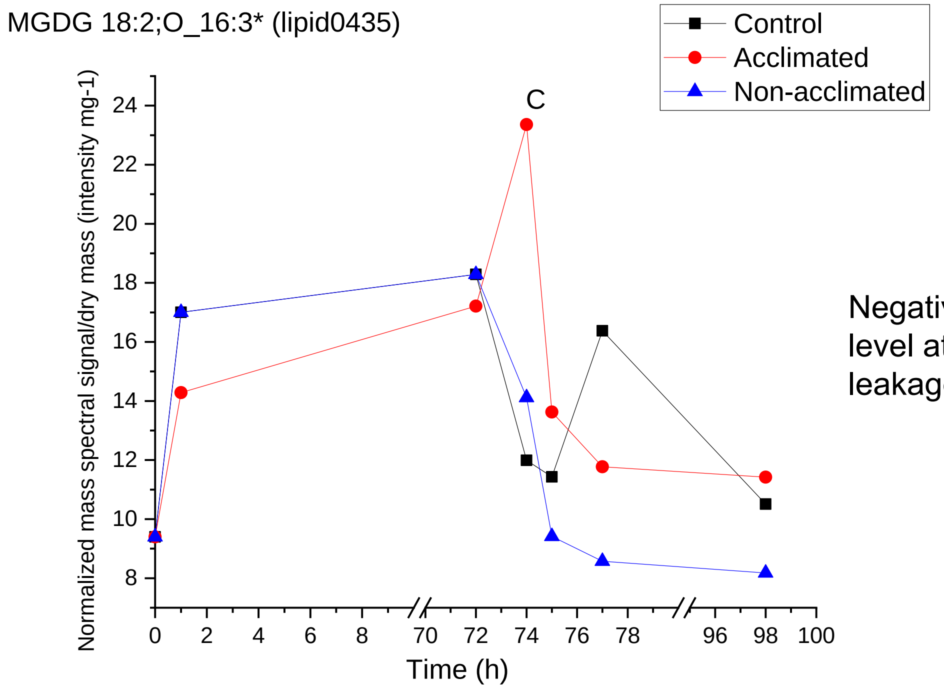

Negative correlation of lipid level at 74 h with final ion leakage

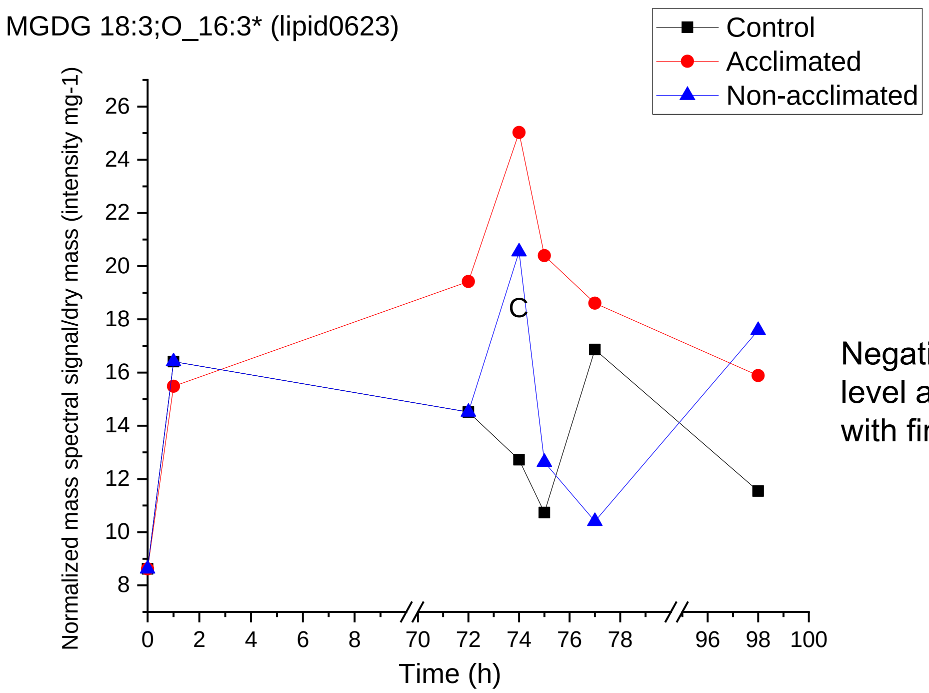

Negative correlation of lipid level at 74, 75, and 77 h with final ion leakage

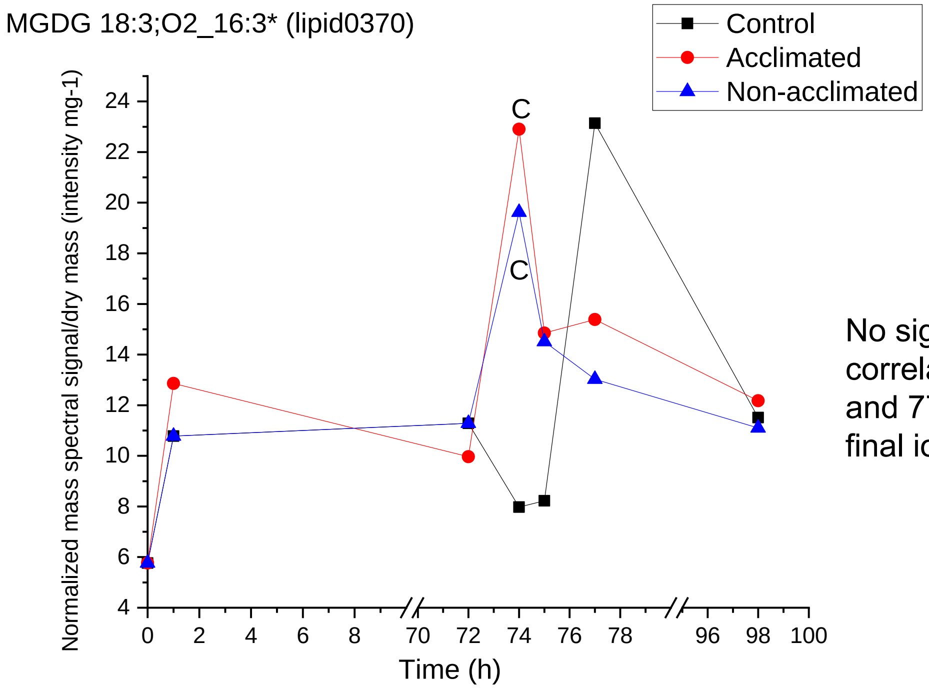

No significant correlation of 74-, 75-, and 77-h lipid levels with final ion leakage

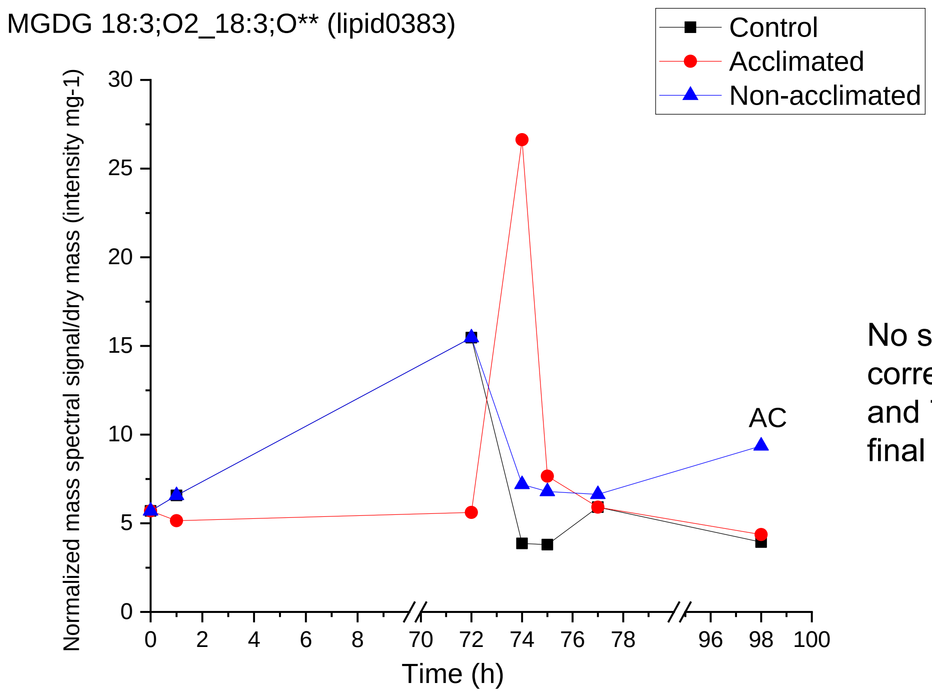

No significant correlation of 74-, 75-, and 77-h lipid levels with final ion leakage

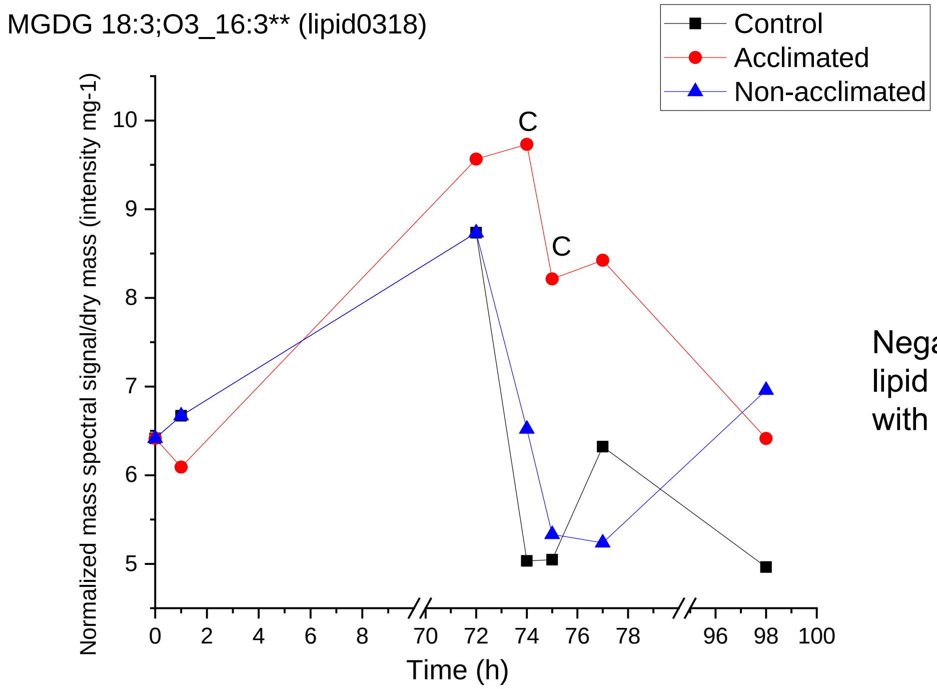

Negative correlation of lipid level at 74 and 75 h with final ion leakage

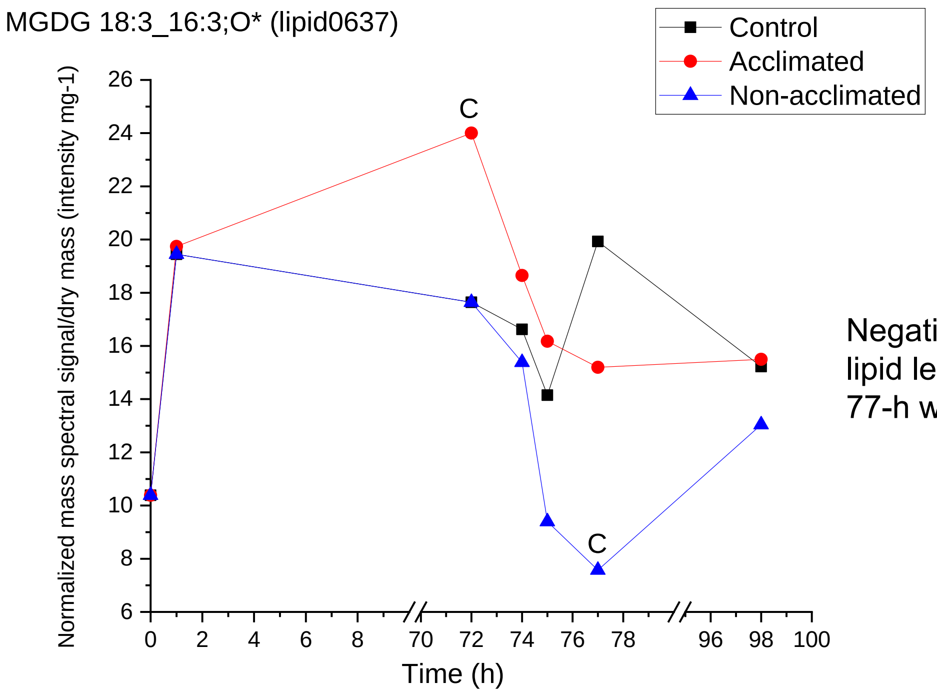

Negative correlation of lipid level at 74-, 75-, and 77-h with final ion leakage

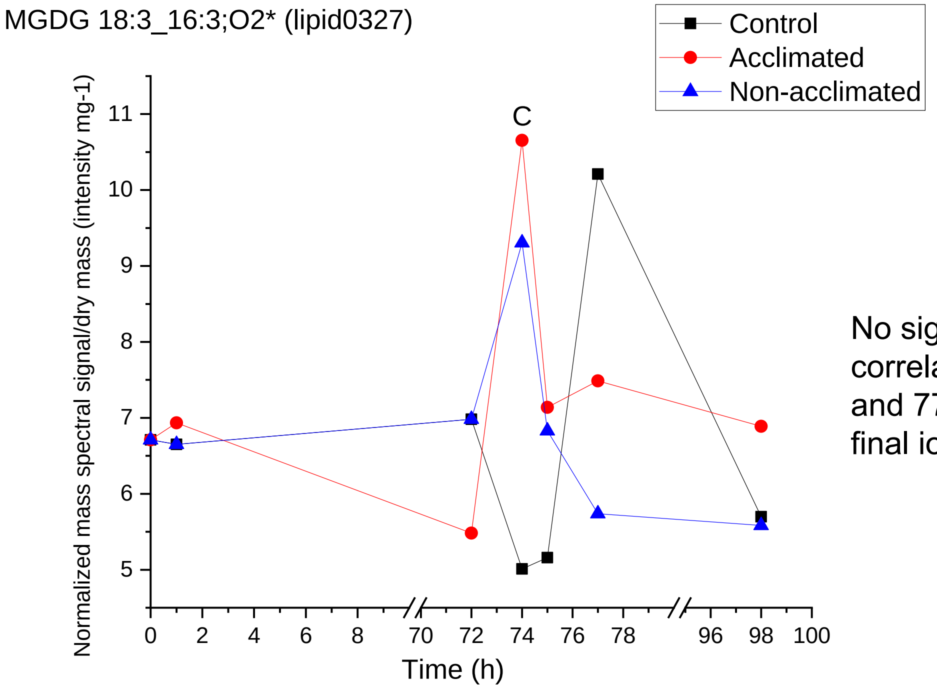

No significant correlation of 74-, 75-, and 77-h lipid levels with final ion leakage

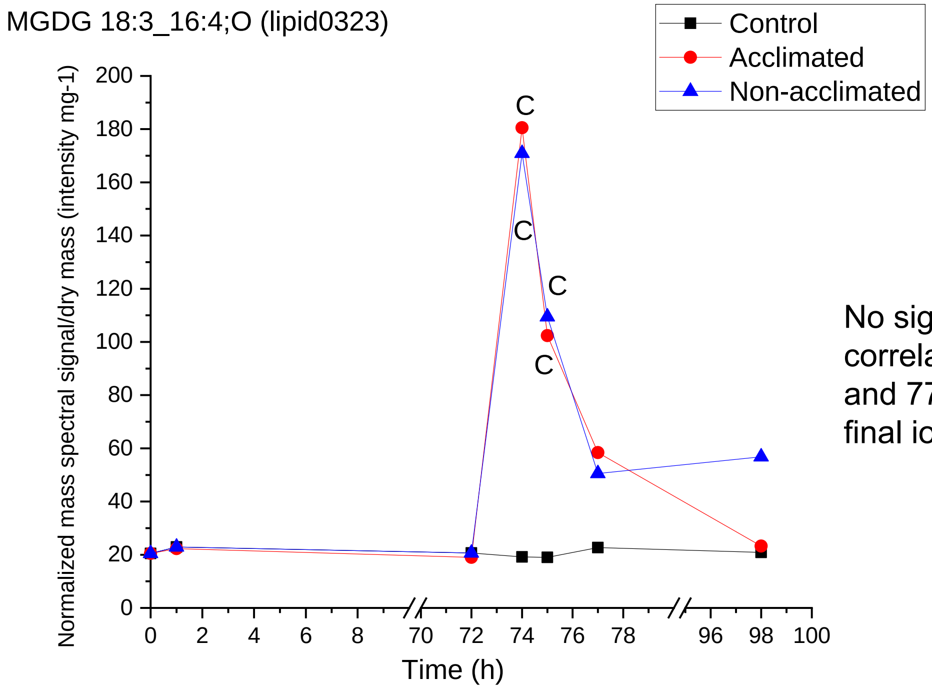

No significant correlation of 74-, 75-, and 77-h lipid levels with final ion leakage

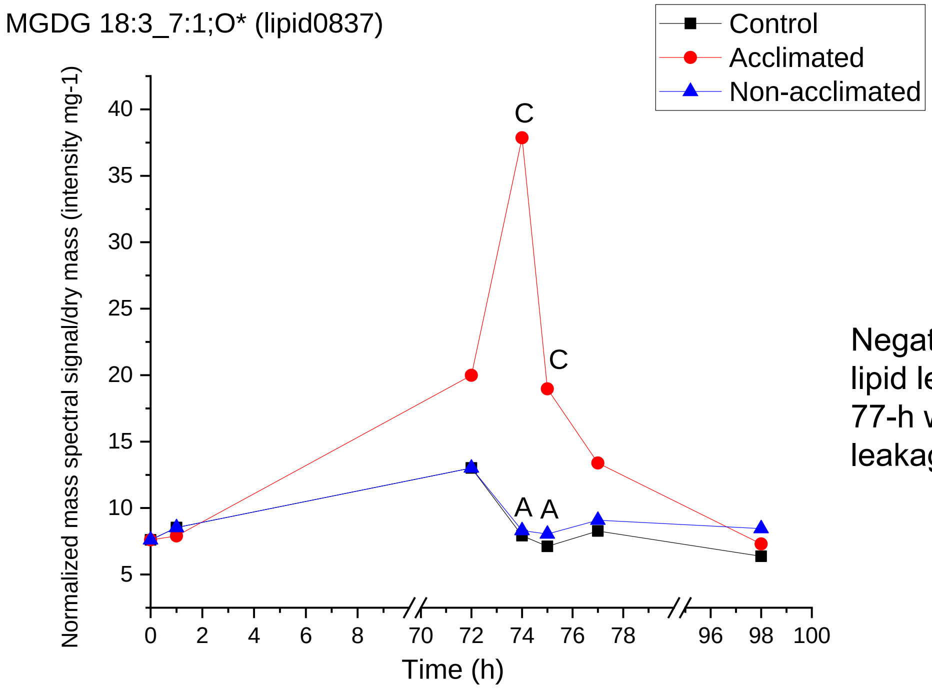

Negative correlation of lipid level at 74-, 75-, and 77-h with final ion leakage

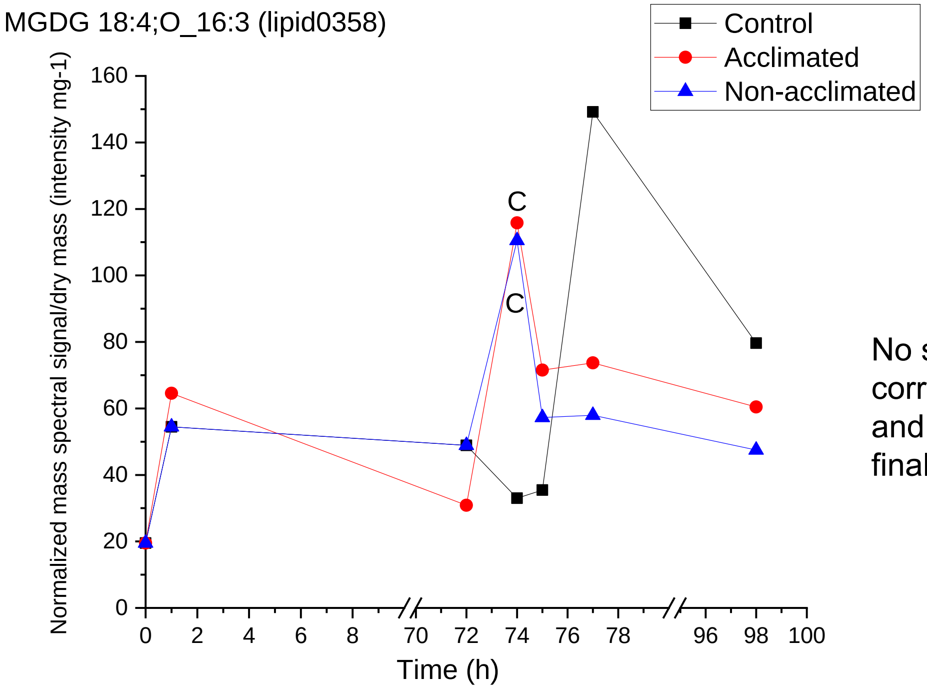

No significant correlation of 74-, 75-, and 77-h lipid levels with final ion leakage

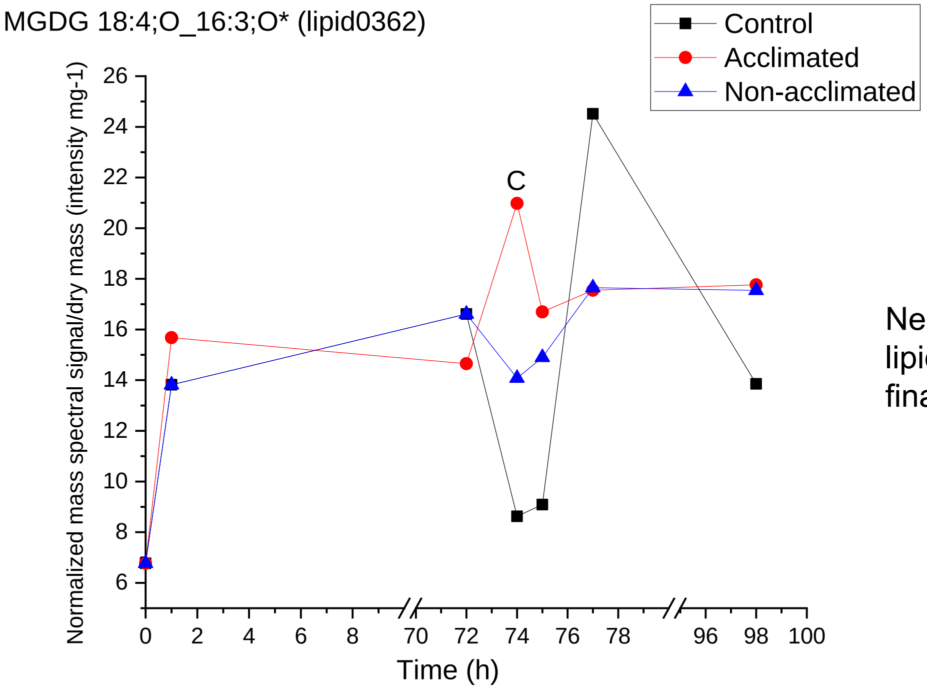

Negative correlation of lipid level at 74 h with final ion leakage

MGDG 18:4;O_16:4;O (16:4;O as fragment; Arabidopside A) (lipid0325)

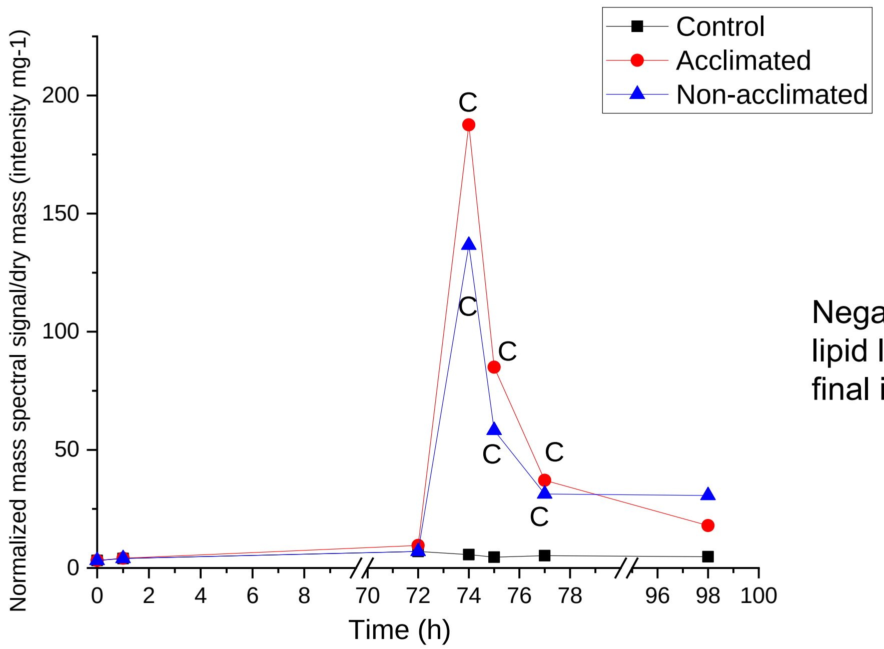

Negative correlation of lipid level at 75 h with final ion leakage

MGDG 18:4;O_16:4;O (18:4;O as fragment; Arabidopside A) (lipid0361)

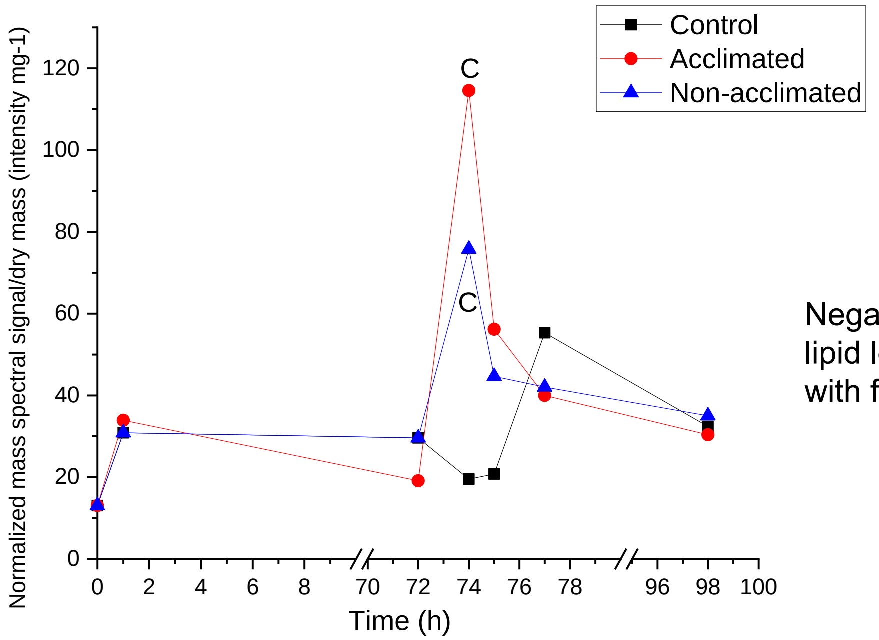

Negative correlation of lipid level at 74 and 75 h with final ion leakage

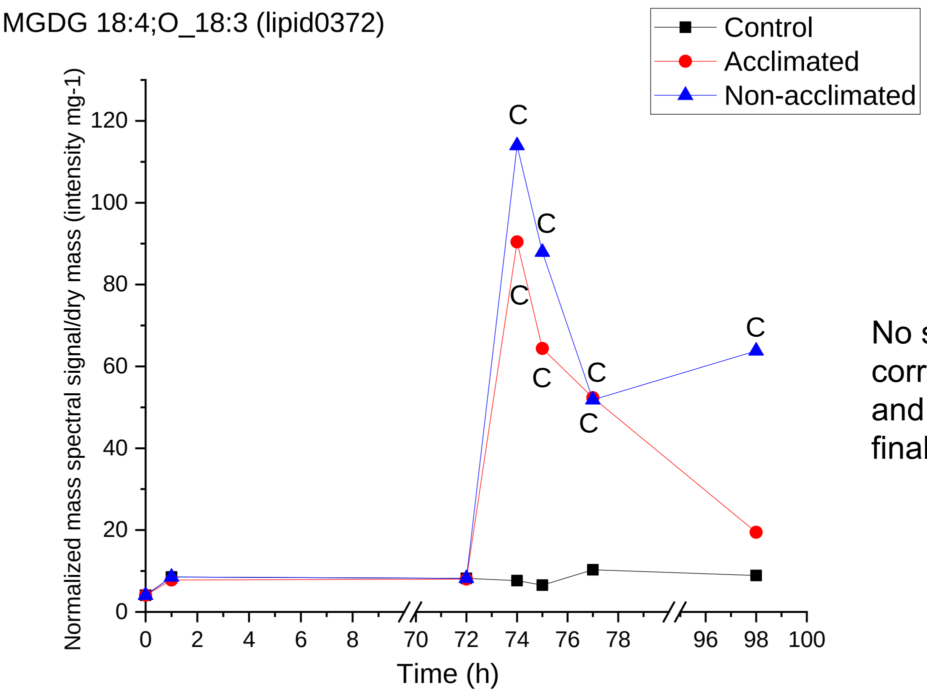

No significant correlation of 74-, 75-, and 77-h lipid levels with final ion leakage

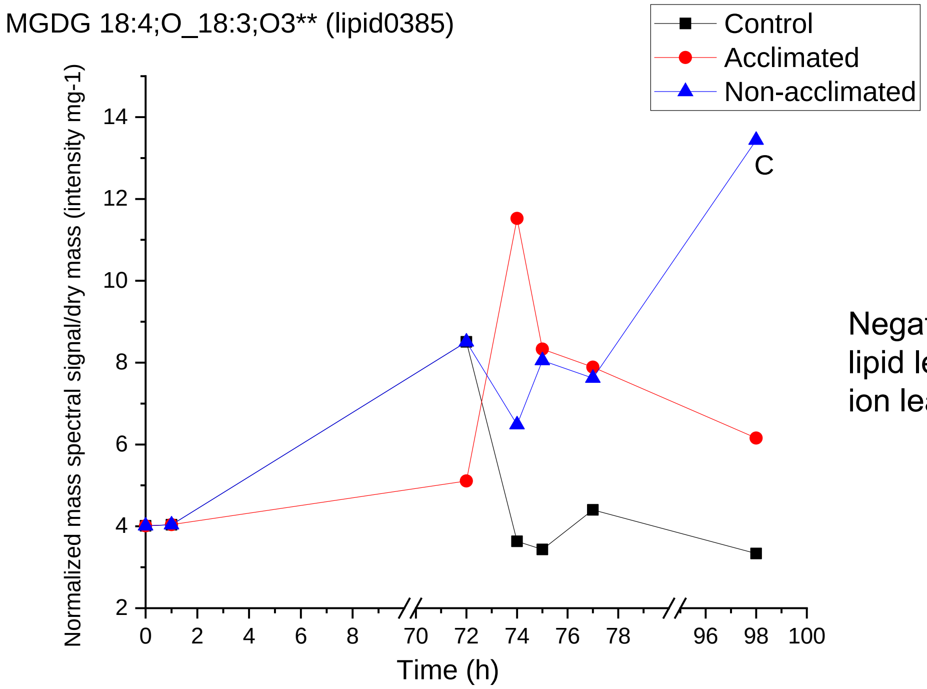

Negative correlation of lipid level at 74 h with final ion leakage

MGDG 18:4;O_18:3;O4** (lipid0386)

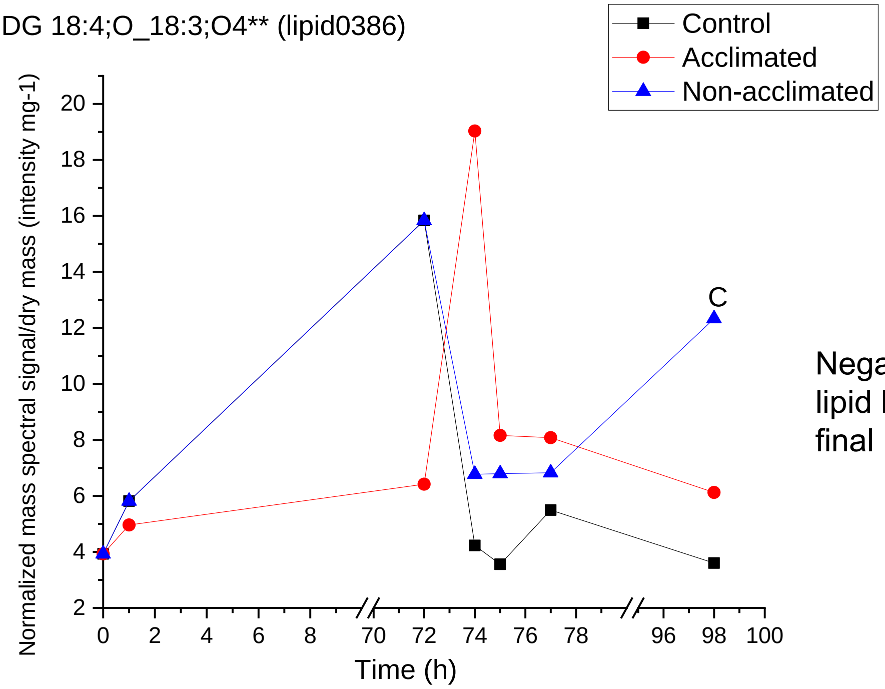

Negative correlation of lipid level at 74 h with final ion leakage

MGDG 18:4;O_18:4;O (Arabidopside B)* (lipid0375)

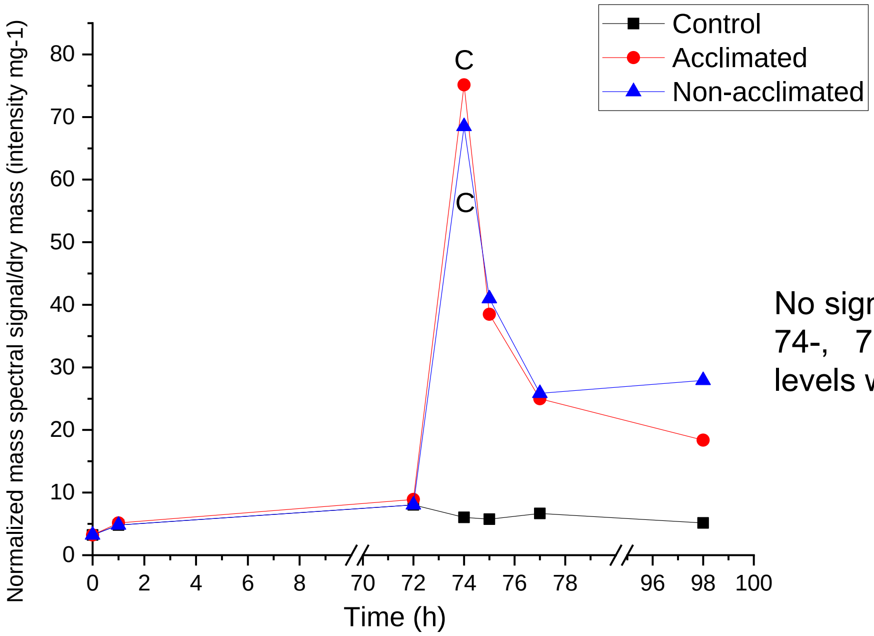

No significant correlation of 74-, 75-, and 77-h lipid levels with final ion leakage

MGDG 18:4;O_18:4;O3 or MGDG 18:4;O_19:3;O2** (lipid0384)

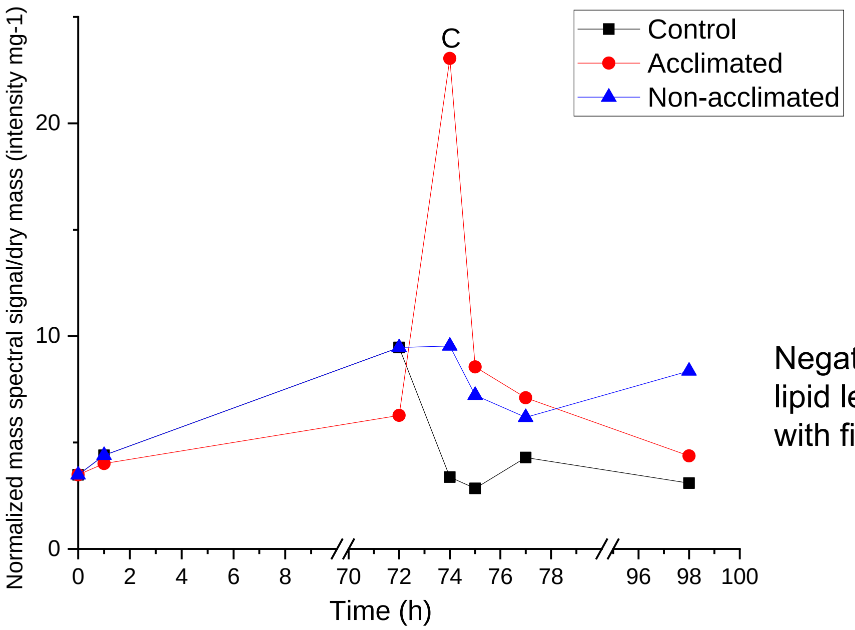

Negative correlation of lipid level at 74 and 75 h with final ion leakage

MGDG 18:4;O3_16:3** (lipid0317)

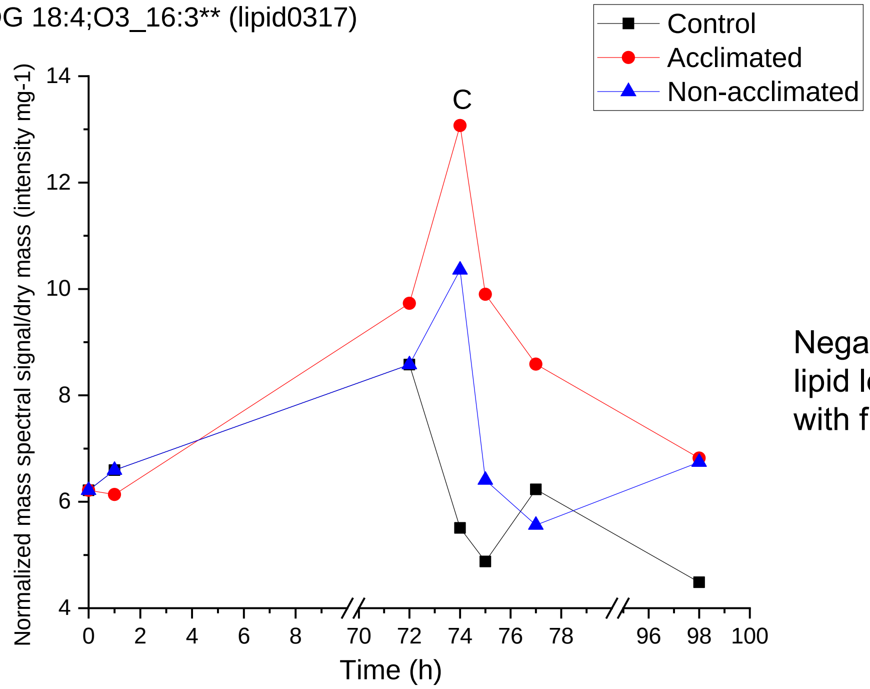

Negative correlation of lipid level at 74 and 75 h with final ion leakage

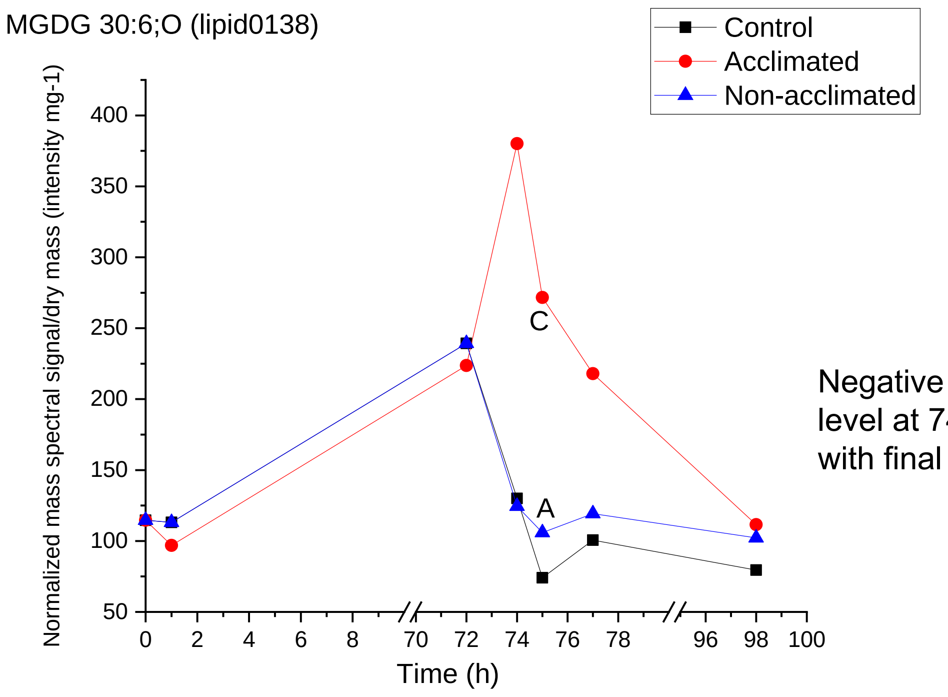

Negative correlation of lipid level at 74-, 75-, and 77-h with final ion leakage

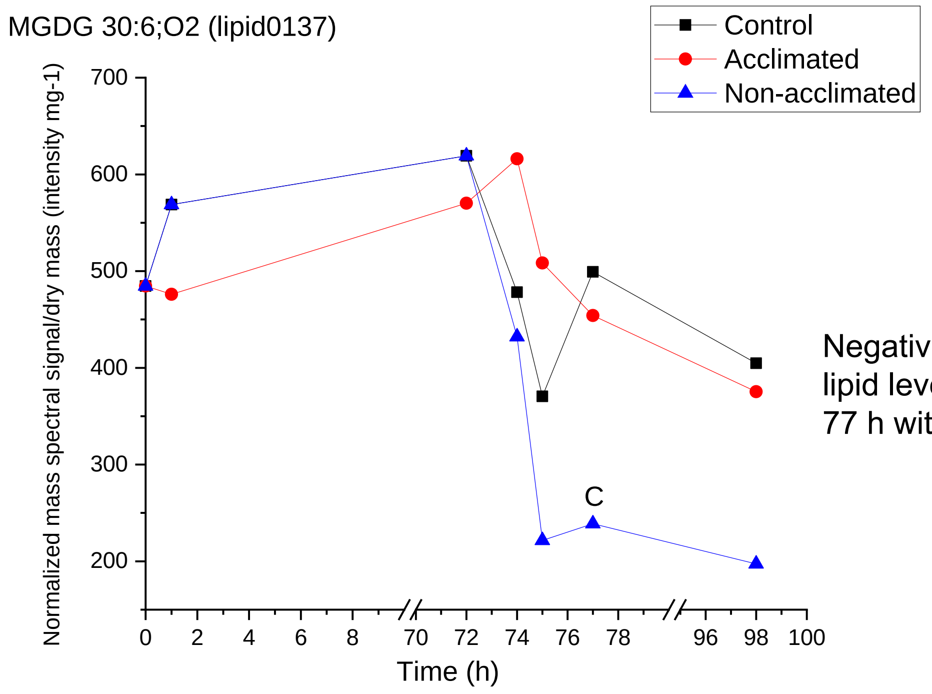

Negative correlation of lipid level at 74, 75, and 77 h with final ion leakage

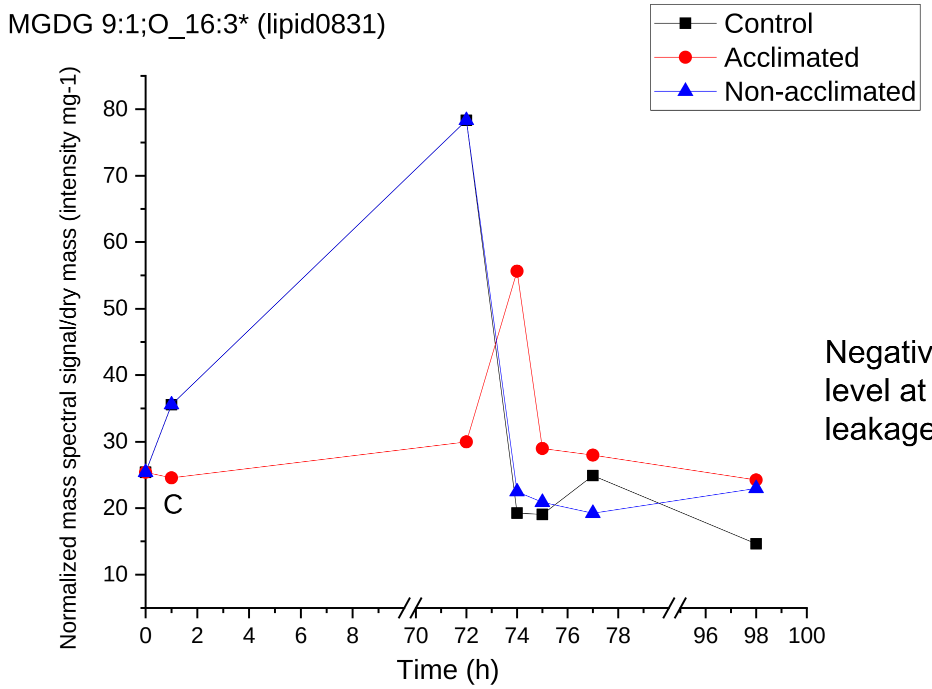

Negative correlation of lipid level at 74 h with final ion leakage

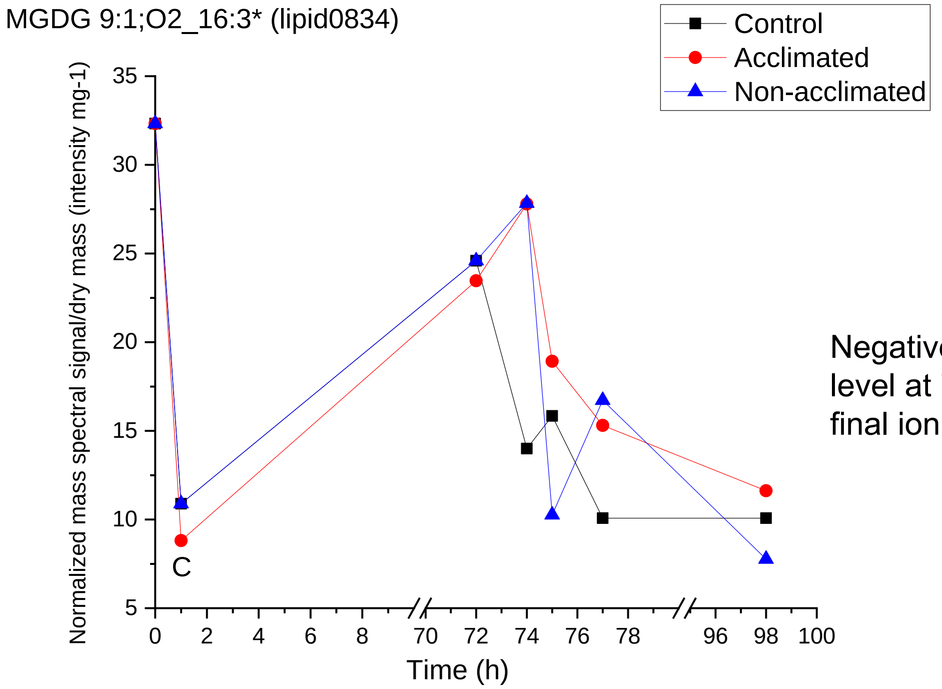

Negative correlation of lipid level at 74 and 75 h with final ion leakage

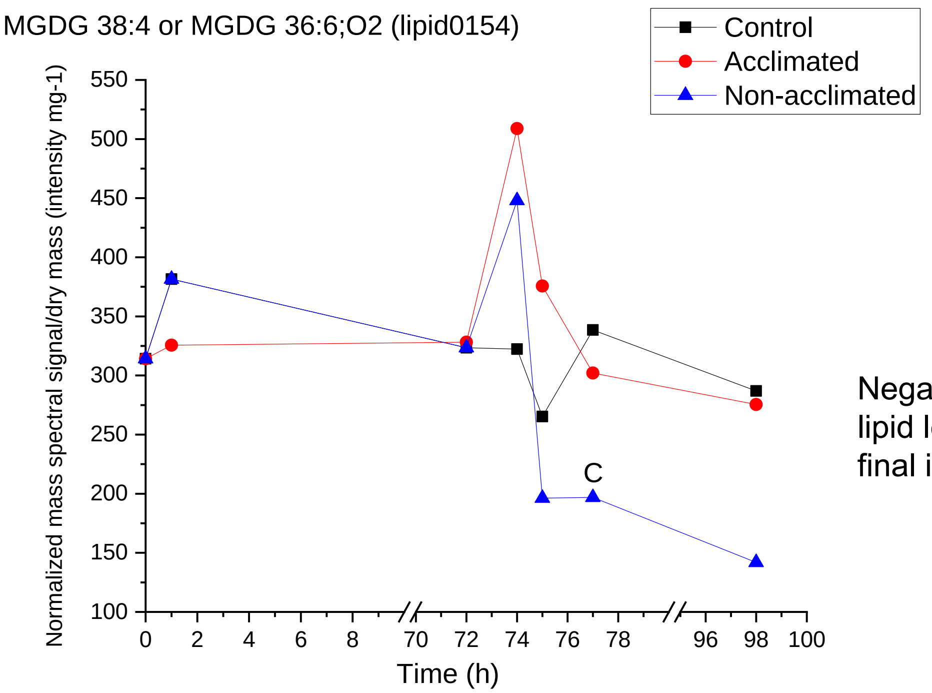

Negative correlation of lipid level at 75 h with final ion leakage

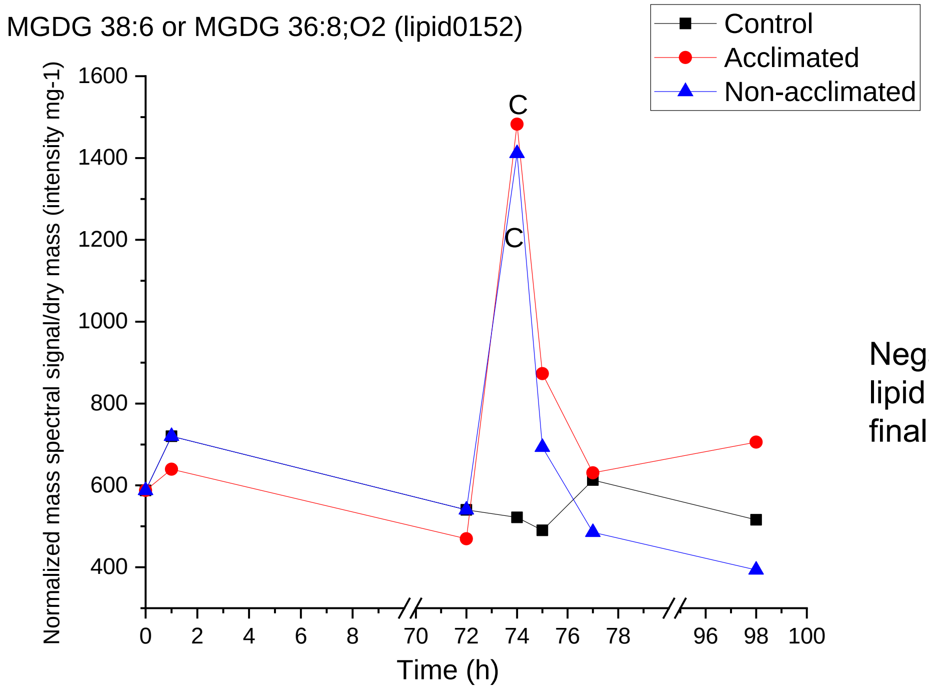

Negative correlation of lipid level at 75 h with final ion leakage

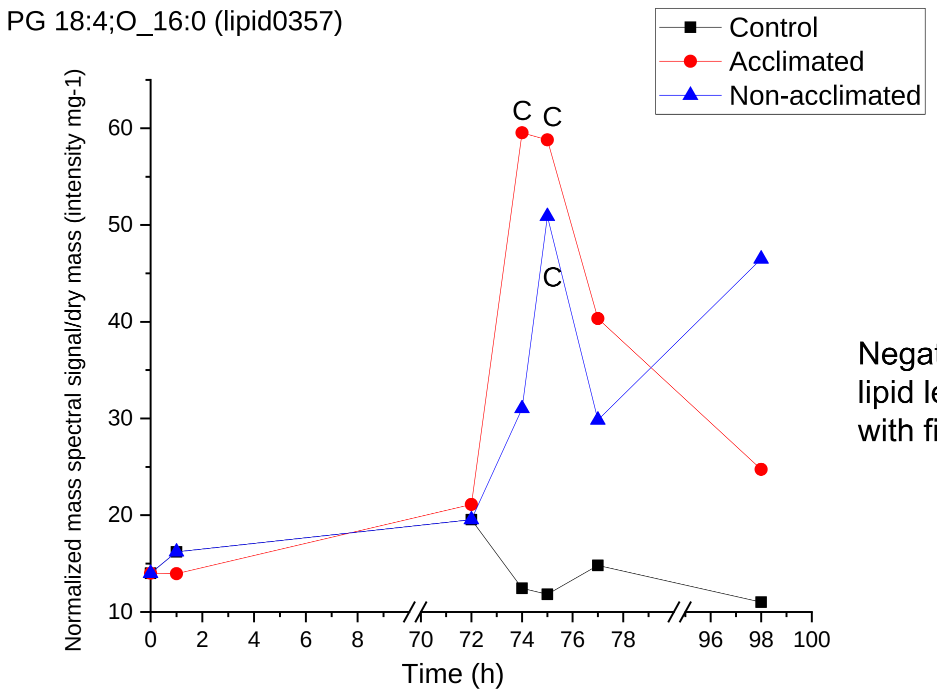

Negative correlation of lipid level at 74 and 75 h with final ion leakage

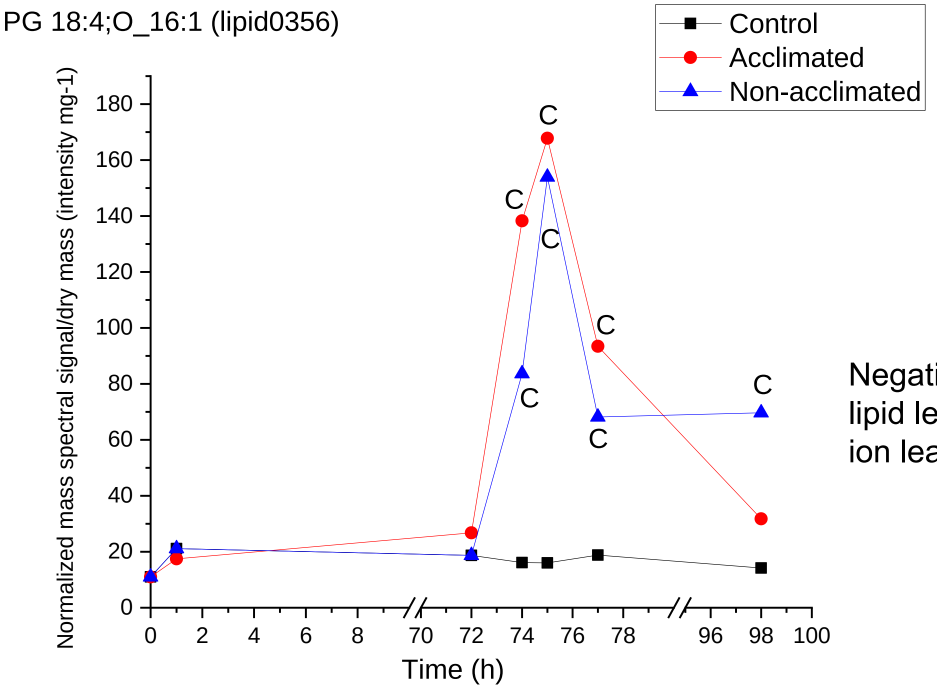

Negative correlation of lipid level at 74 h with final ion leakage

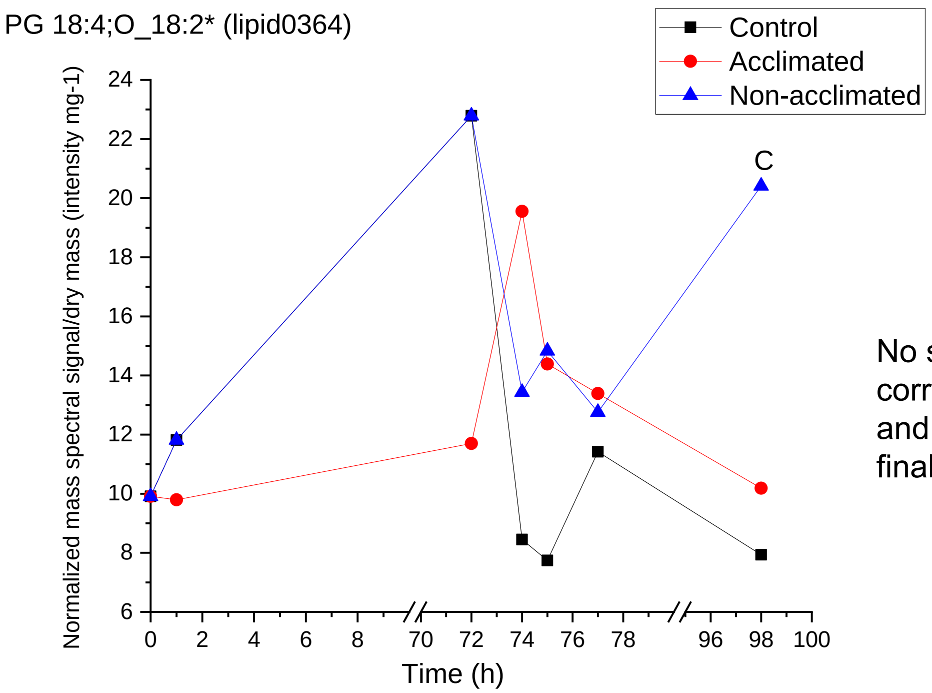

No significant correlation of 74-, 75-, and 77-h lipid levels with final ion leakage

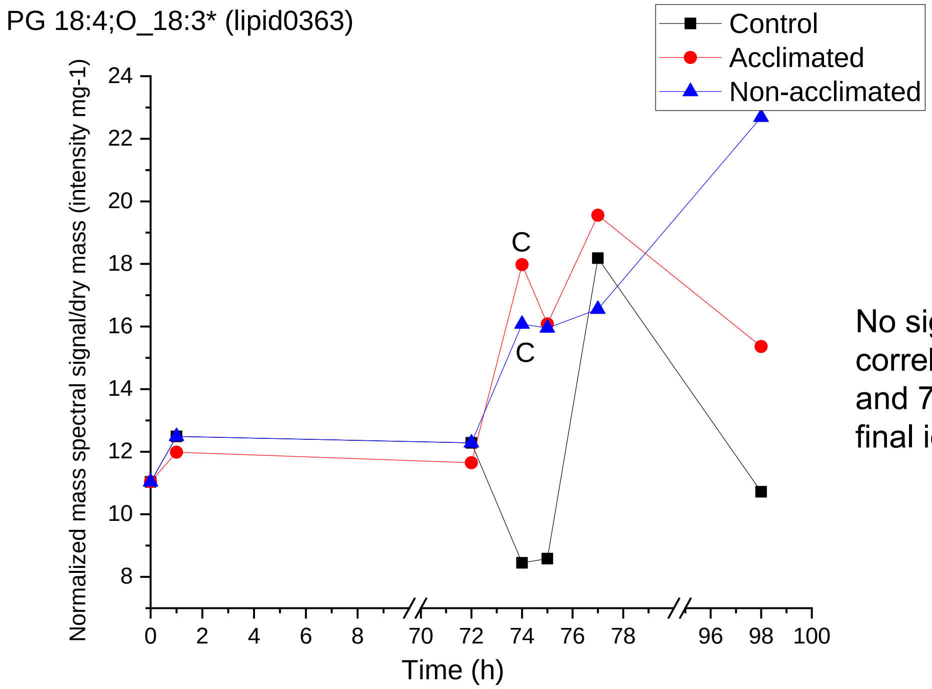

No significant correlation of 74-, 75-, and 77-h lipid levels with final ion leakage

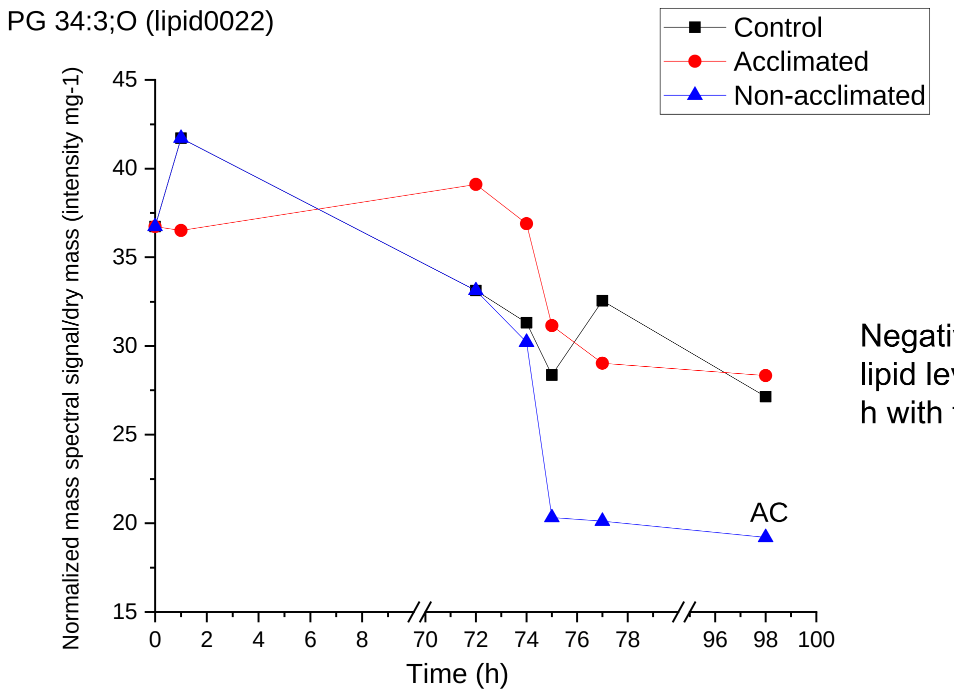

Negative correlation of lipid level at 74, 75, and 77 h with final ion leakage

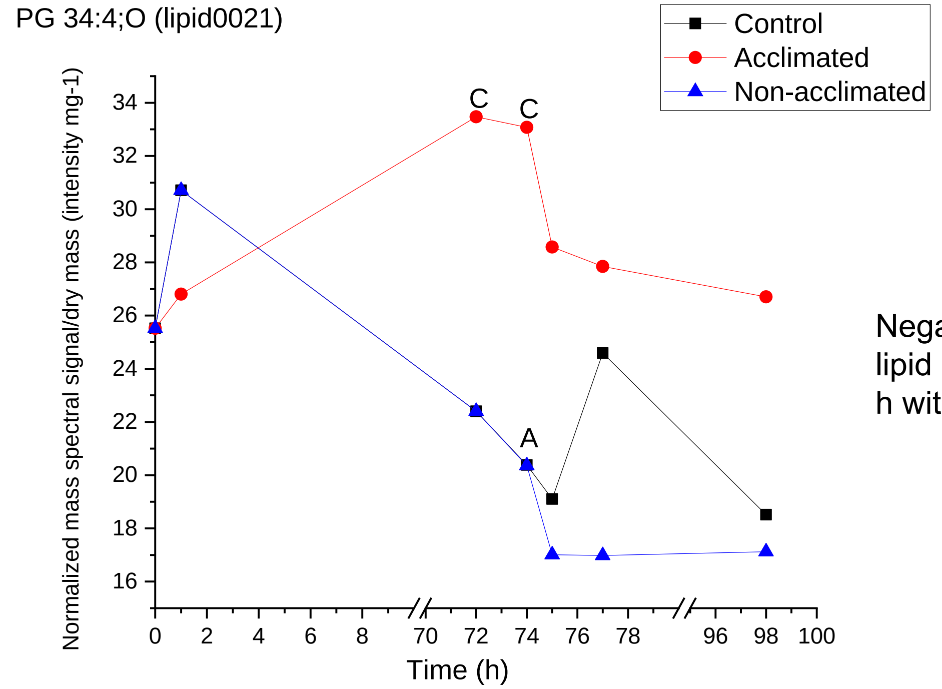

Negative correlation of lipid level at 74, 75, and 77 h with final ion leakage

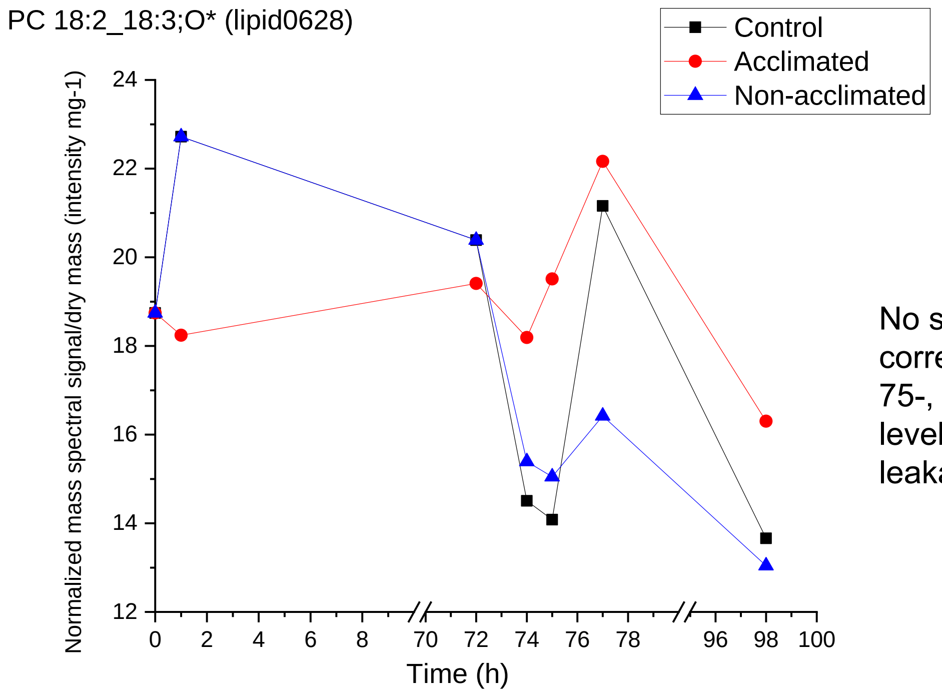

No significant correlation of 74-, 75-, and 77-h lipid levels with final ion leakage

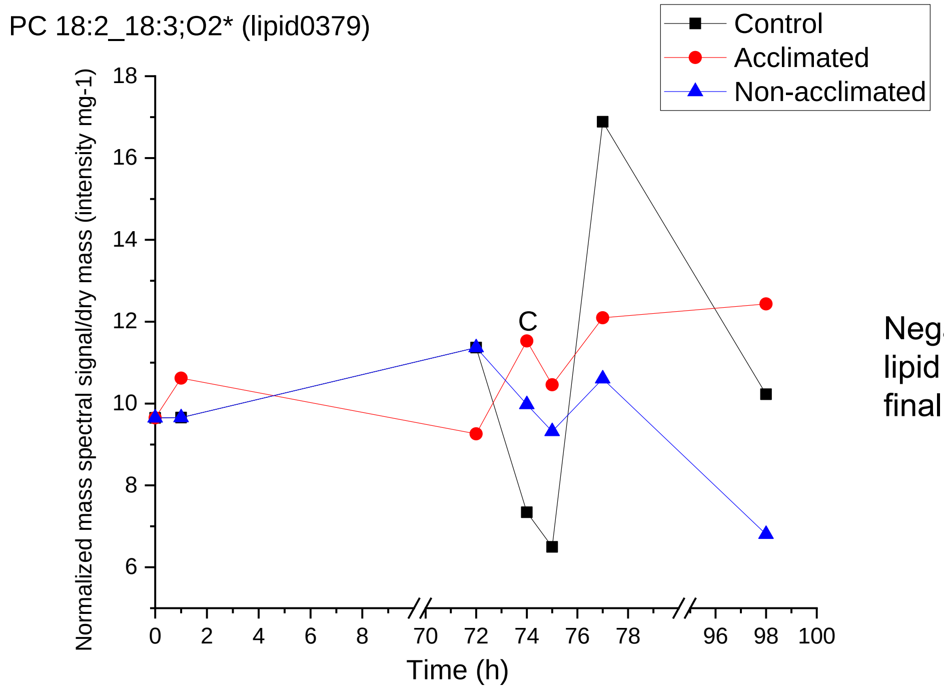

Negative correlation of lipid level at 77 h with final ion leakage

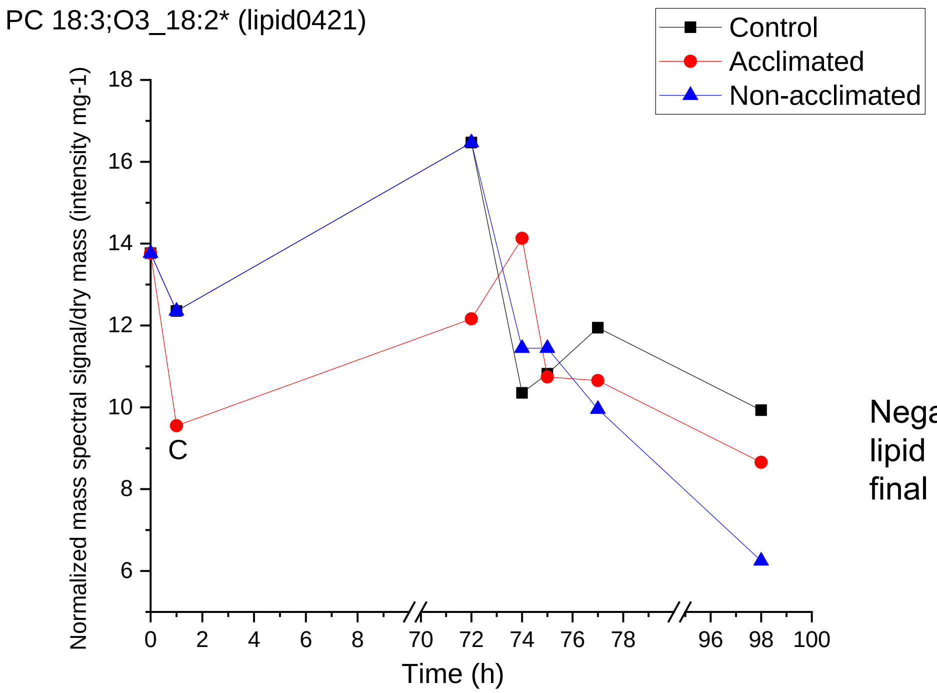

Negative correlation of lipid level at 74 h with final ion leakage

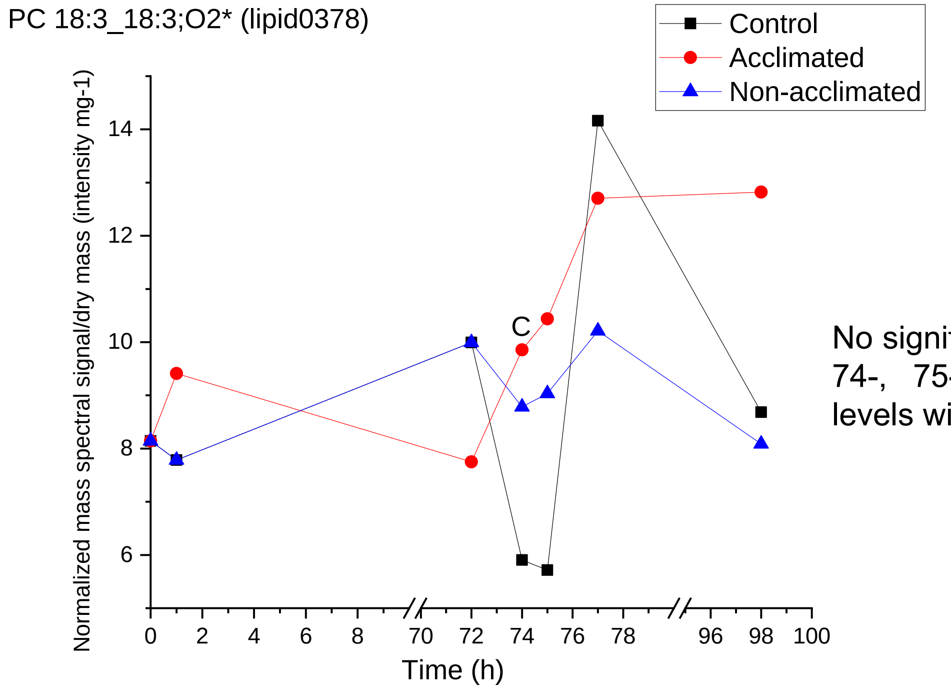

No significant correlation of 74-, 75-, and 77-h lipid levels with final ion leakage

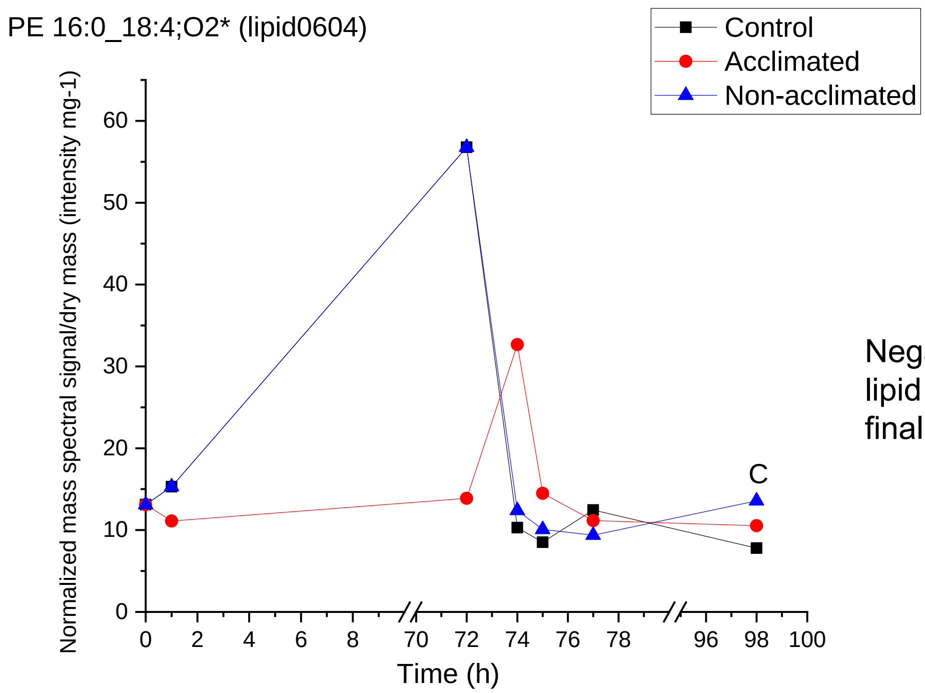

Negative correlation of lipid level at 74 h with final ion leakage

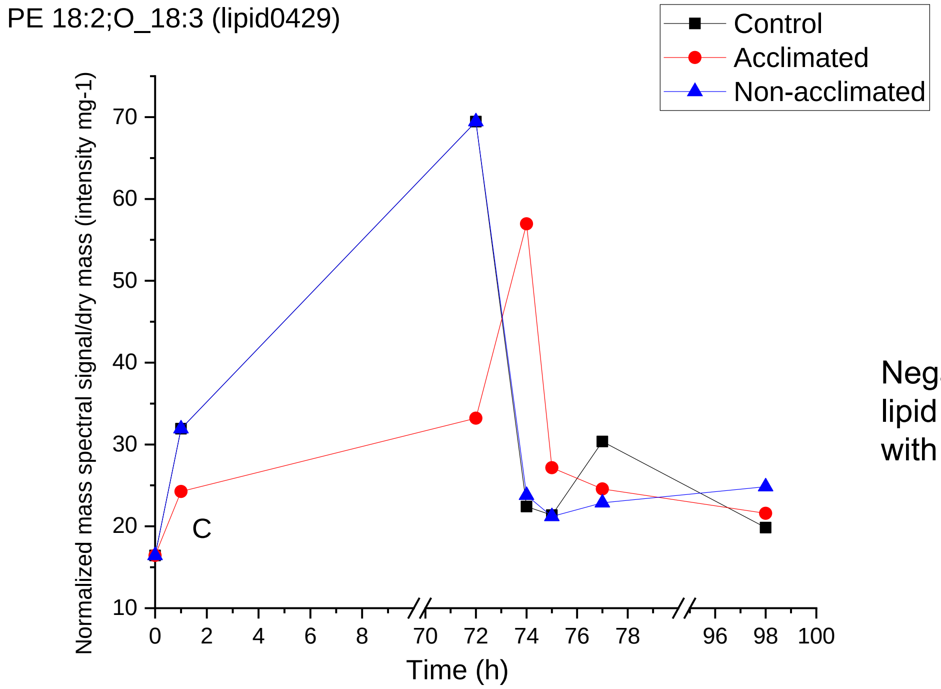

Negative correlation of lipid level at 74 and 75 h with final ion leakage

Figure S7. Time courses of levels of selected monoacyl polar lipids in rosettes of control, non-acclimated, and acclimated plants. Treatments are shown in Figure 1. Asterisks indicate lipids with quality control (pooled sample) levels less than 0.75 (*) or 0.25 (**) units of normalized mass spectral intensity, where 1 = intensity of 1 pmol of internal standard. "C" indicates that the lipid level in non-acclimated or acclimated plants is significantly different than the control level, and "A" indicates that the lipid level in non-acclimated plants is significantly different than the level in acclimated plants (Table S2). Indicated on each plot is whether there is significant correlation of lipid level at the 74-, 75-, and 77-h time points with final (98 h) ion leakage. Abbreviation: lysoPS (LPS).

Lipid number	Panel	Class, oxidation	Lipid name
lipid0157	7A	DGMG, non-oxidized	DGMG 16:0
lipid0156	7A	DGMG, non-oxidized	DGMG 16:3
lipid0159	7B	DGMG, non-oxidized	DGMG 18:3
lipid0158	7B	DGMG, oxidized	DGMG 16:4;O
lipid0160	7C	DGMG, oxidized	DGMG 18:4;O
lipid0132	7C	MGMG, non-oxidized	MGMG 16:3
lipid0177	7D	SQMG, non-oxidized	SQMG 16:0
lipid0178	7D	SQMG, non-oxidized	SQMG 18:3
lipid0001	7E	LPG, non-oxidized	LPG 16:1
lipid0025	7E	LPC, non-oxidized	LPC 16:0
lipid0029	7F	LPC, non-oxidized	LPC 18:0**
lipid0028	7F	LPC, non-oxidized	LPC 18:1*
lipid0027	7G	LPC, non-oxidized	LPC 18:2
lipid0026	7G	LPC, non-oxidized	LPC 18:3
lipid0052	7H	LPE, non-oxidized	LPE 16:0
lipid0055	7H	LPE, non-oxidized	LPE 18:1*
lipid0054	7I	LPE, non-oxidized	LPE 18:2
lipid0053	7I	LPE, non-oxidized	LPE 18:3
lipid0792	7J	LPI, non-oxidized	LPI 16:0
lipid0793	7J	LPI, non-oxidized	LPI 18:3
lipid0796	7K	LPS, non-oxidized	LPS 16:0**

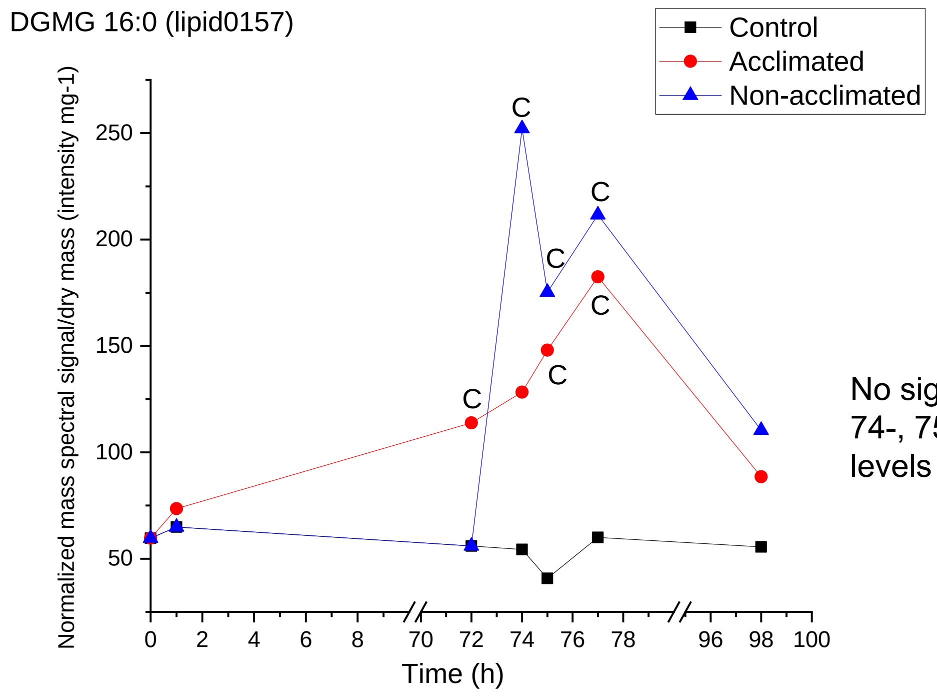

No significant correlation of 74-, 75-, and 77-h lipid levels with final ion leakage

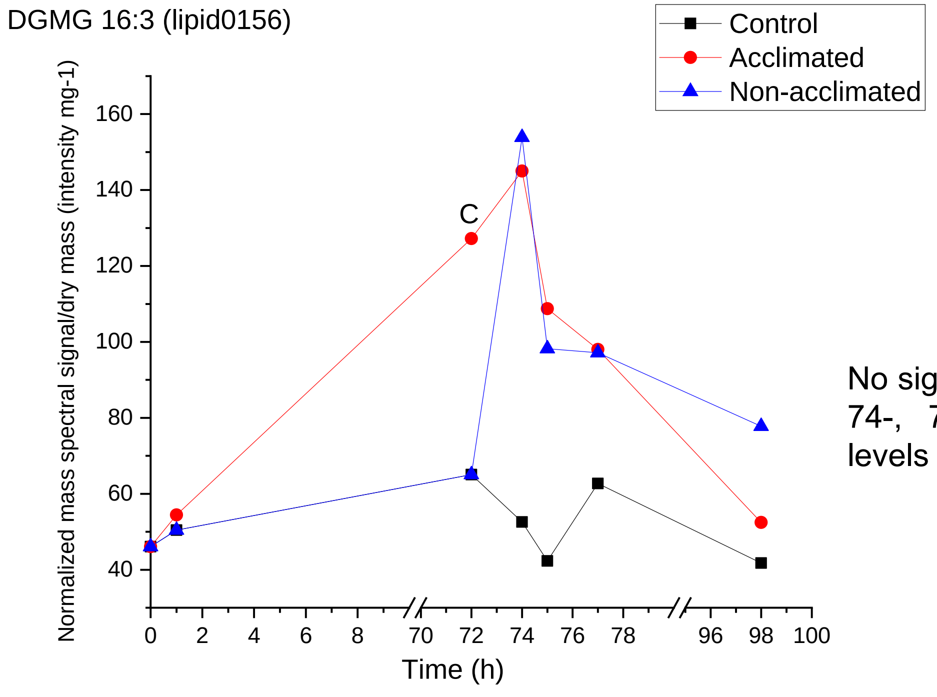

No significant correlation of 74-, 75-, and 77-h lipid levels with final ion leakage

7A

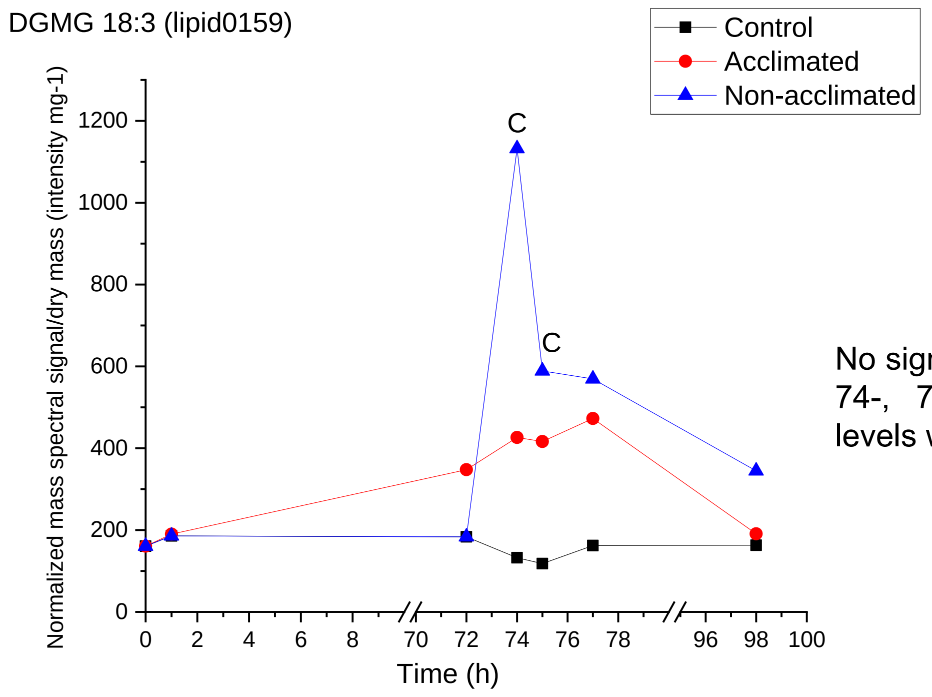

No significant correlation of 74-, 75-, and 77-h lipid levels with final ion leakage

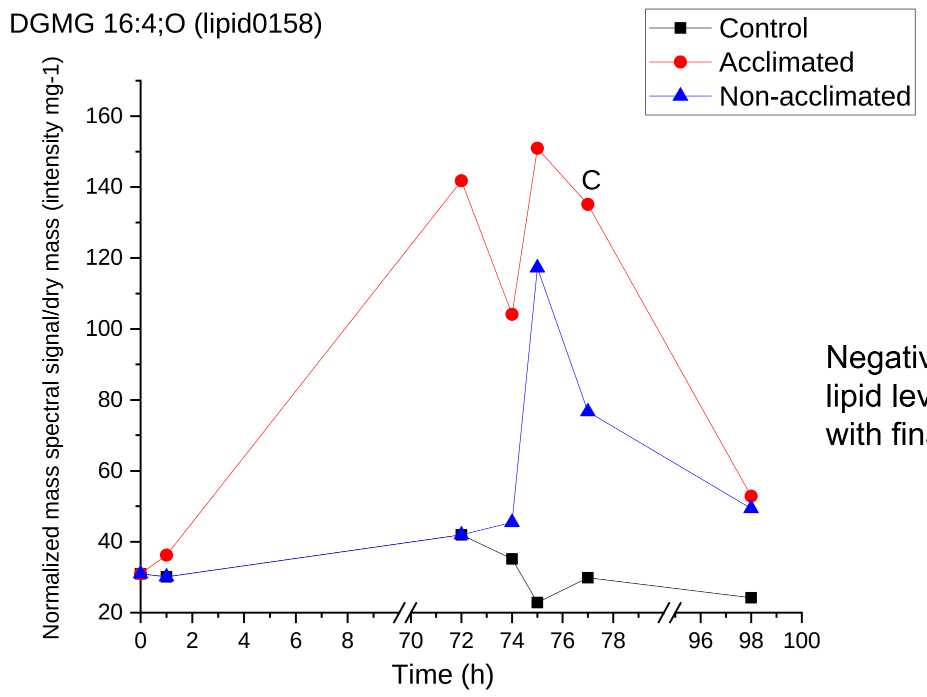

Negative correlation of lipid level at 75 and 77 h with final ion leakage

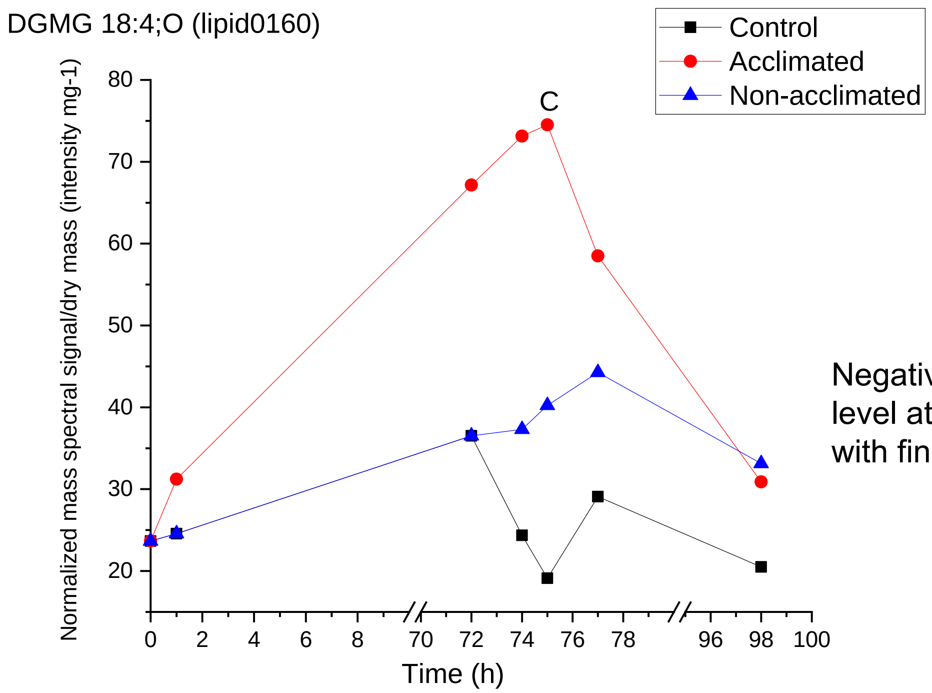

Negative correlation of lipid level at 74, 75, and 77 h with final ion leakage

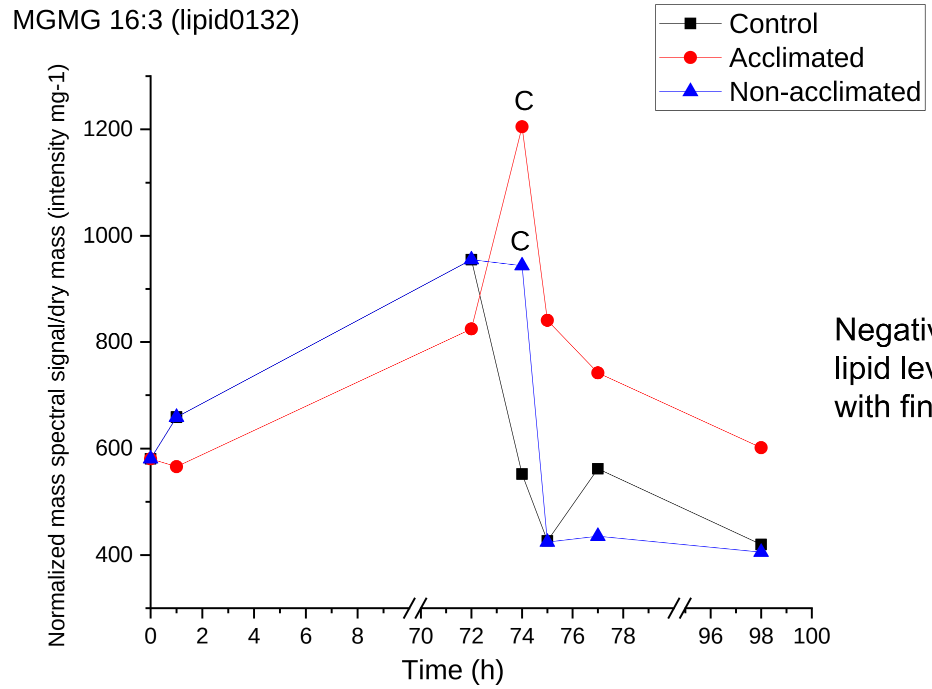

Negative correlation of lipid level at 75 and 77 h with final ion leakage

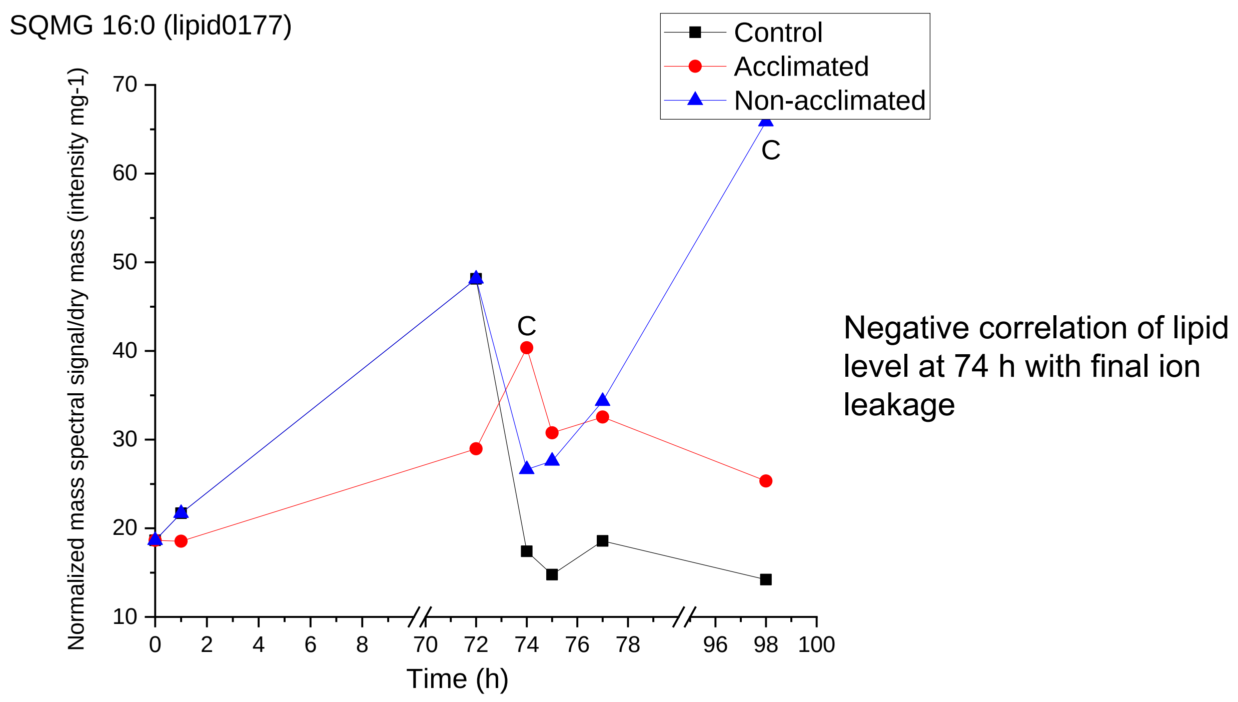
SQMG 16:0 (lipid0177)
Control
Acclimated
Non-acclimated
C
C
Normalized mass spectral signal/dry mass (intensity mg-1)
Time (h)
Negative correlation of lipid level at 74 h with final ion leakage

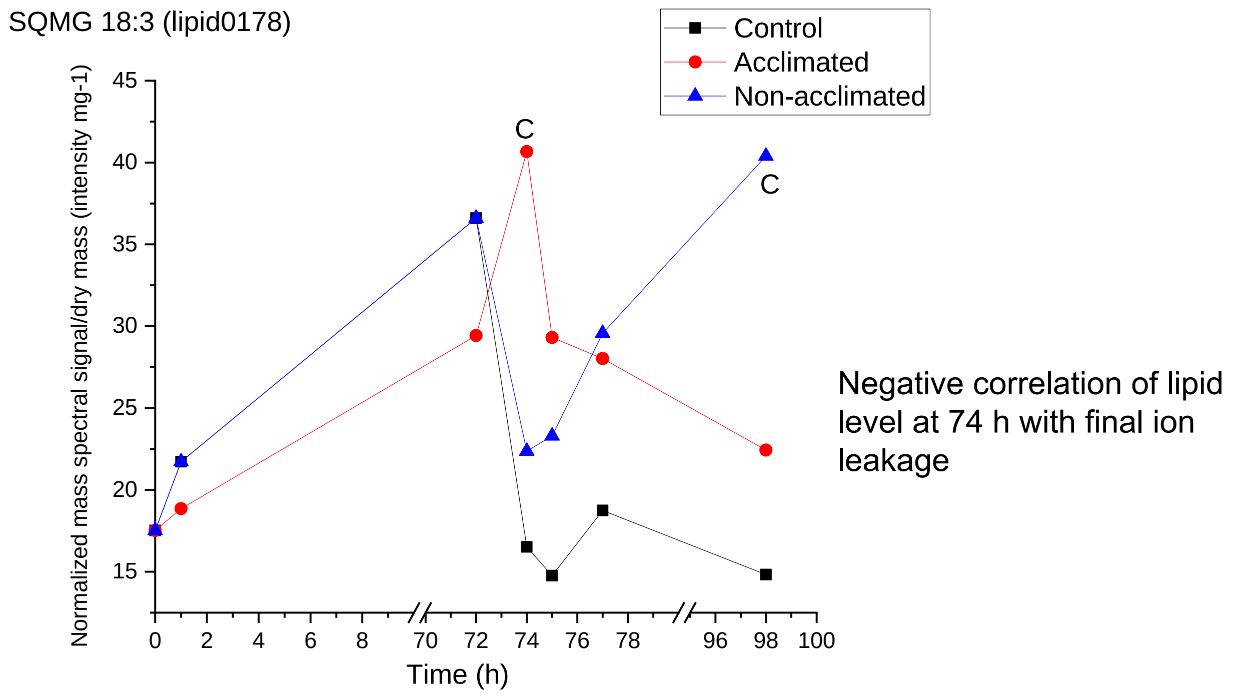
SQMG 18:3 (lipid0178)
Control
Acclimated
Non-acclimated
C
C
Normalized mass spectral signal/dry mass (intensity mg-1)
Time (h)
Negative correlation of lipid level at 74 h with final ion leakage

7D

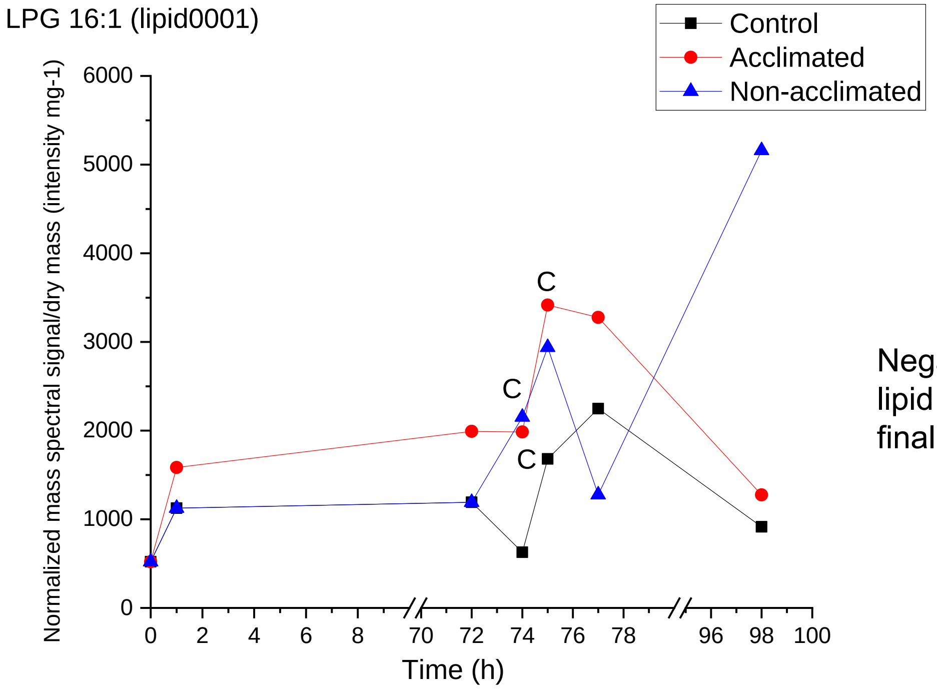

Negative correlation of lipid level at 77 h with final ion leakage

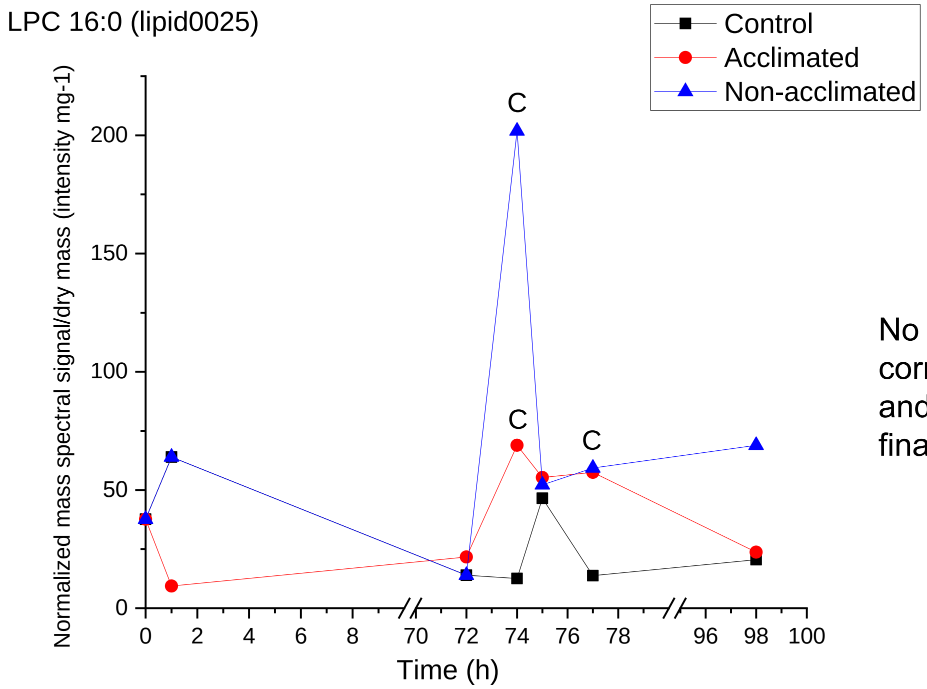

No significant correlation of 74-, 75-, and 77-h lipid levels with final ion leakage

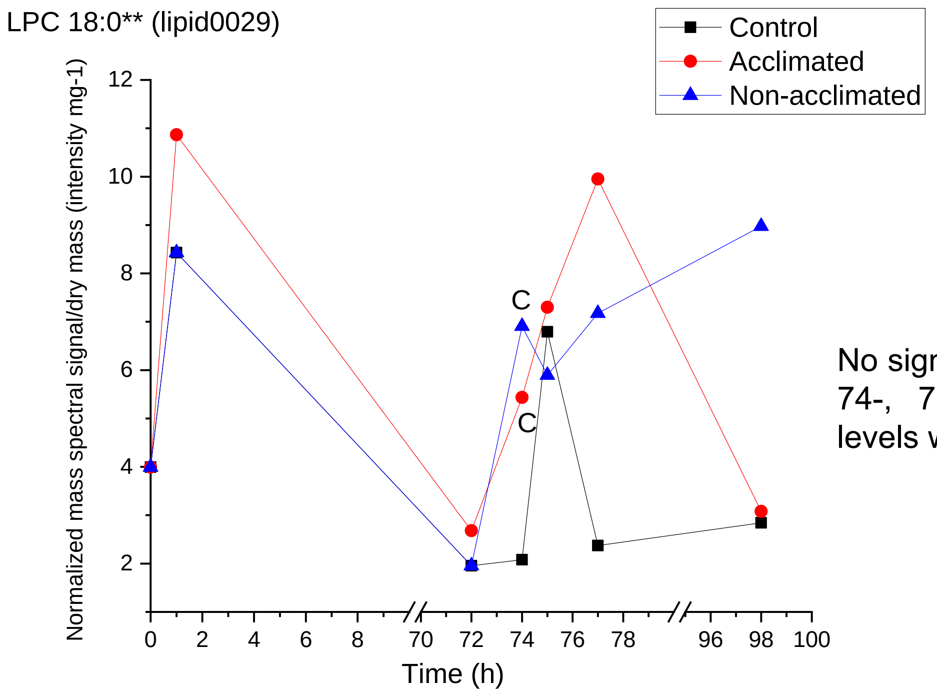

No significant correlation of 74-, 75-, and 77-h lipid levels with final ion leakage

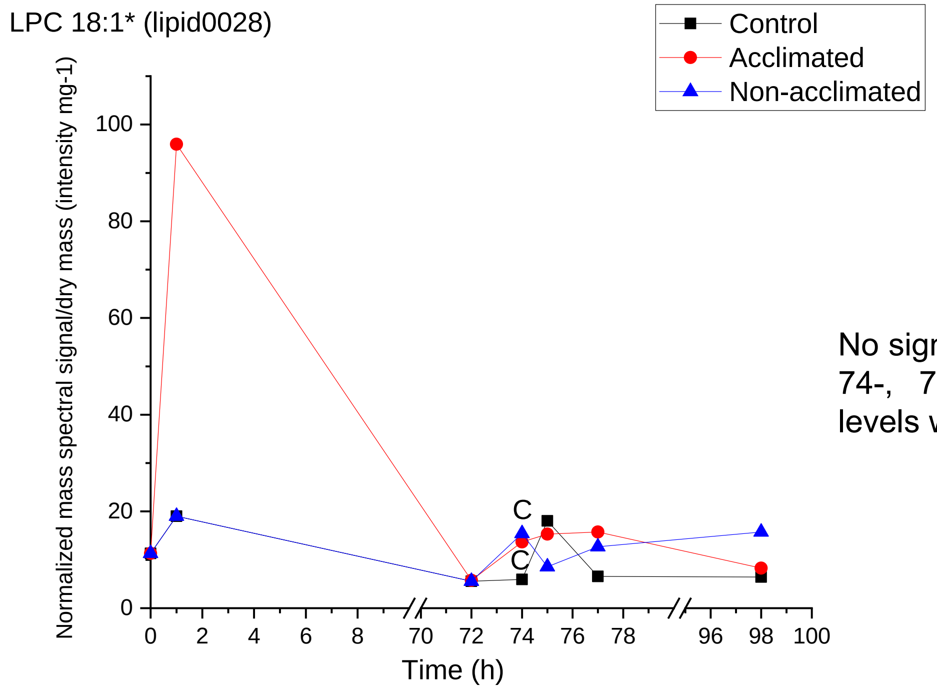

No significant correlation of 74-, 75-, and 77-h lipid levels with final ion leakage

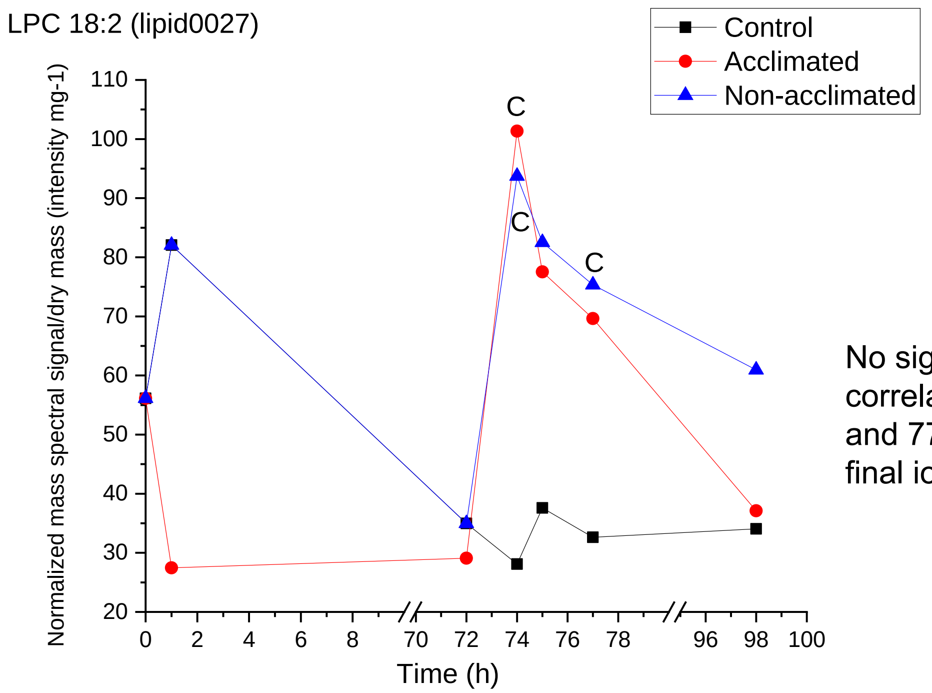

No significant correlation of 74-, 75-, and 77-h lipid levels with final ion leakage

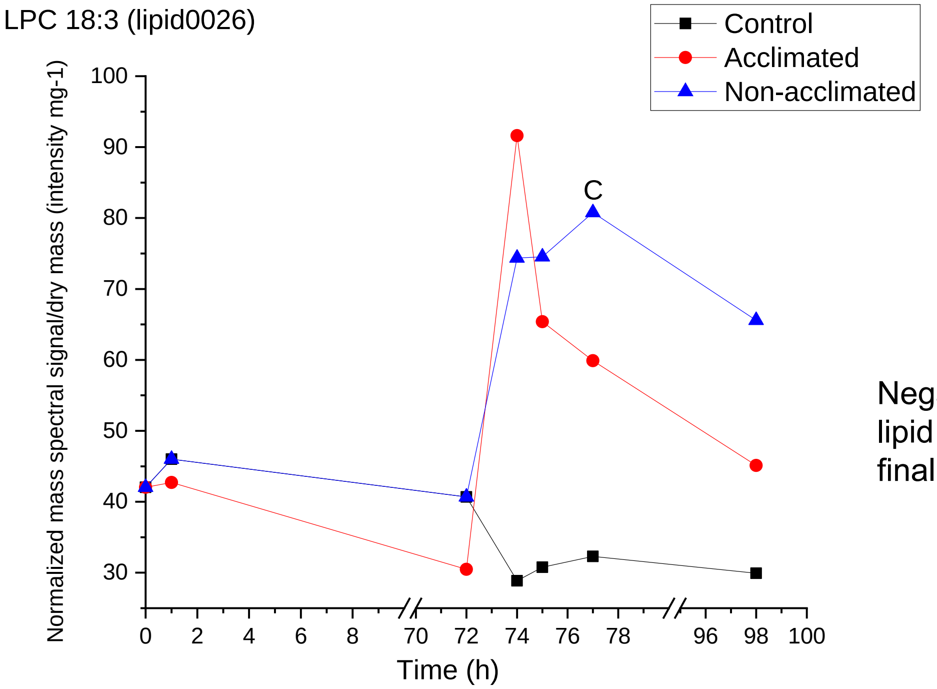

Negative correlation of lipid level at 74 h with final ion leakage

7G

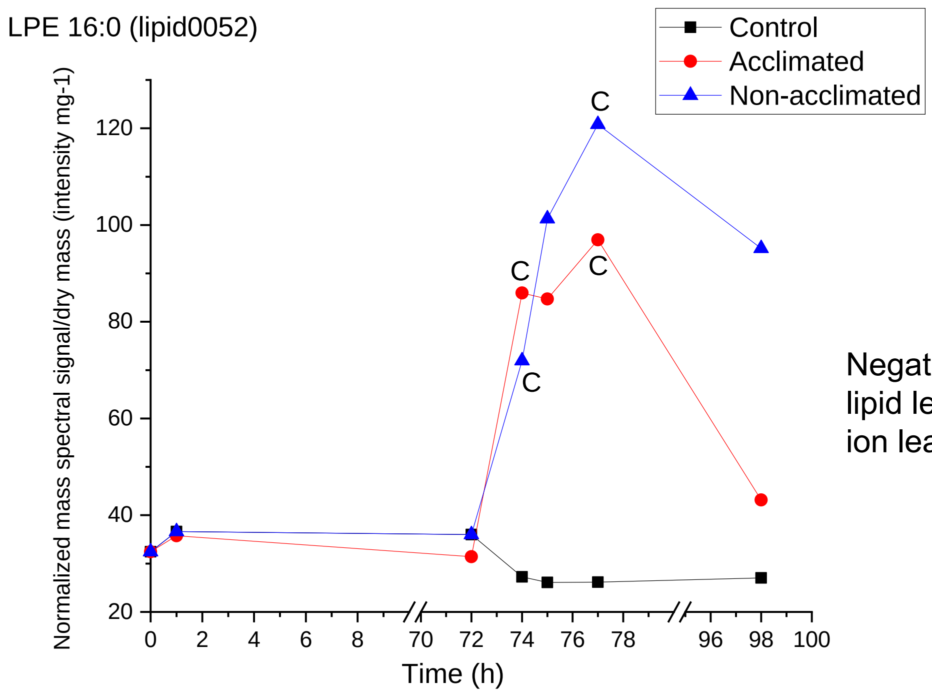

Negative correlation of lipid level at 74 h with final ion leakage

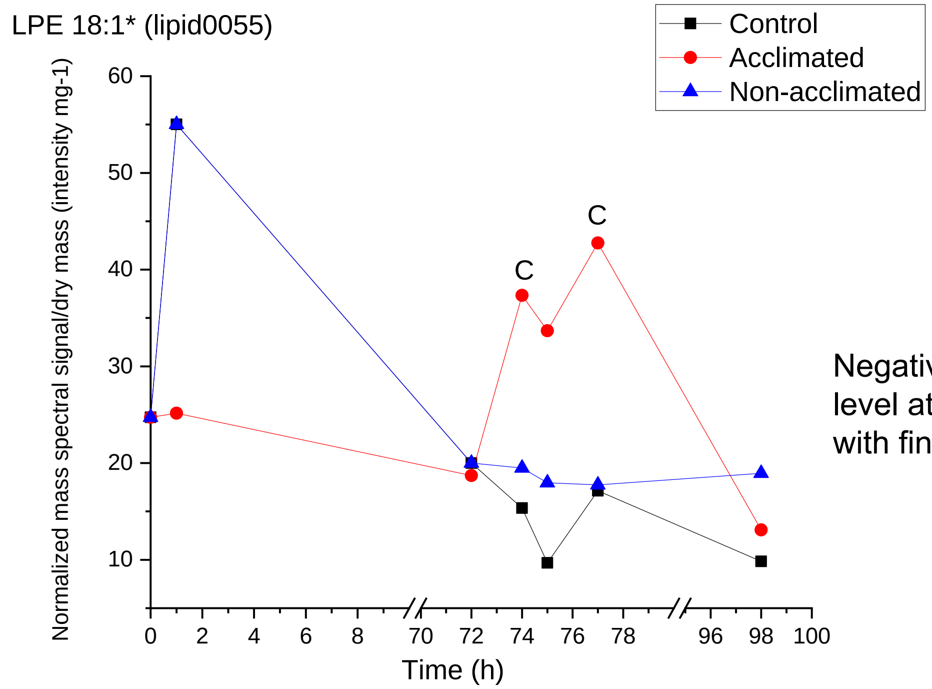

Negative correlation of lipid level at 74, 75, and 77 h with final ion leakage

7H

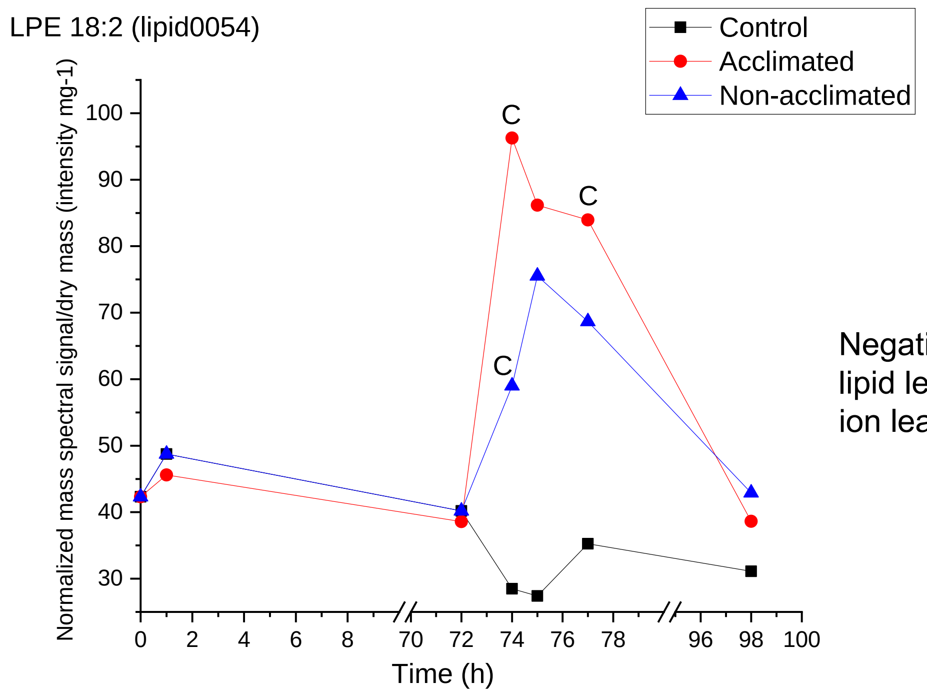

Negative correlation of lipid level at 74 h with final ion leakage

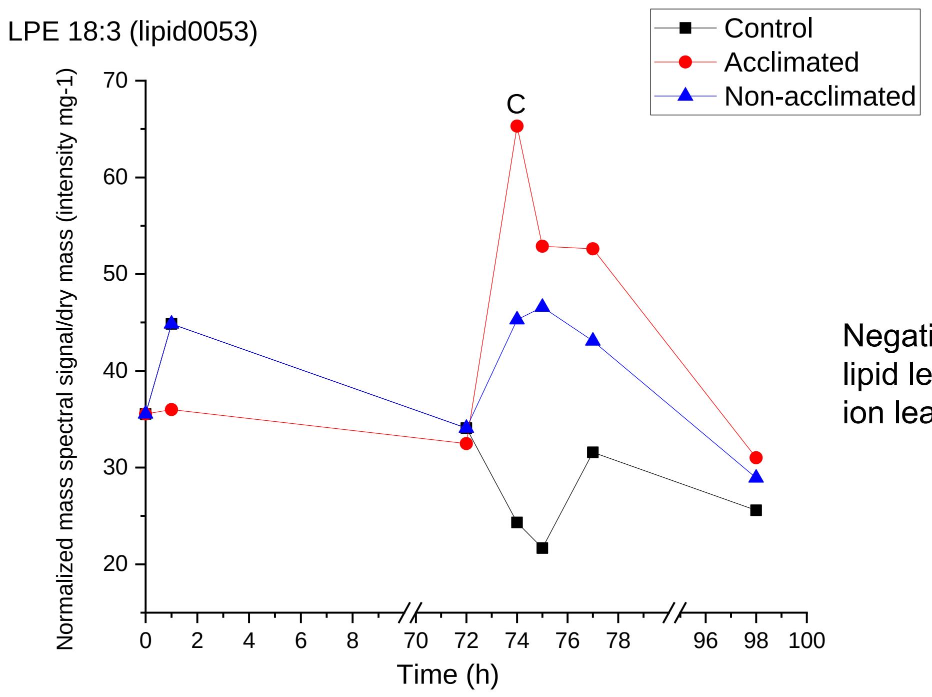

Negative correlation of lipid level at 74 h with final ion leakage

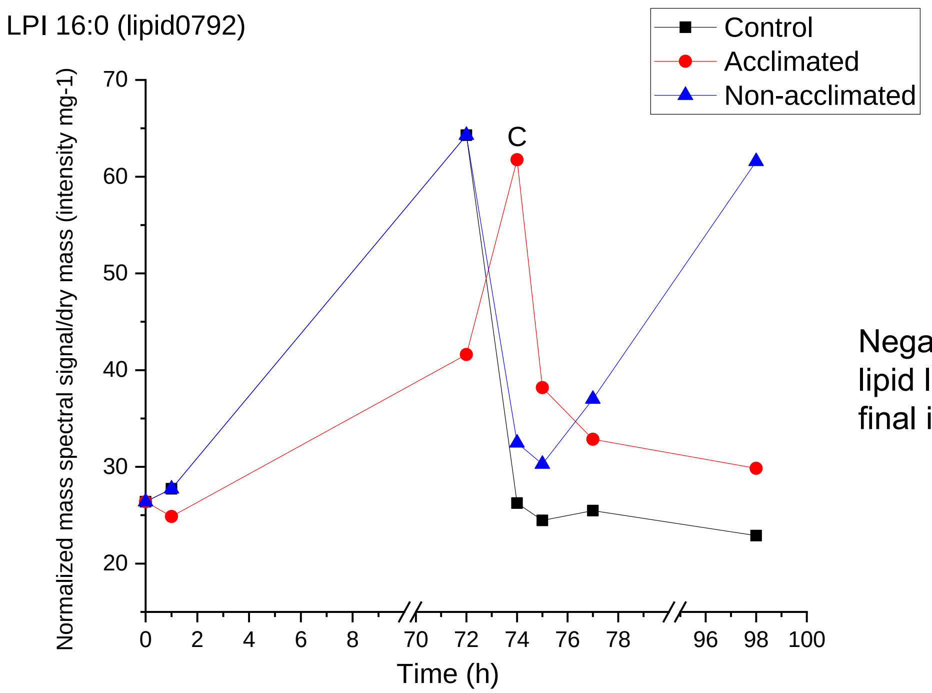

Negative correlation of lipid level at 74 h with final ion leakage

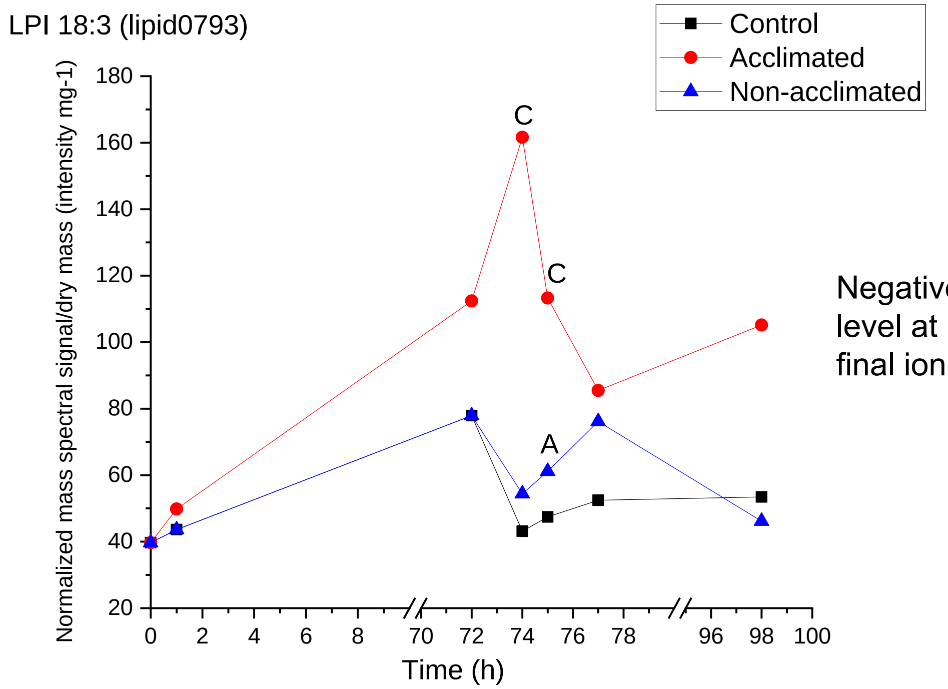

Negative correlation of lipid level at 74 and 75 h with final ion leakage

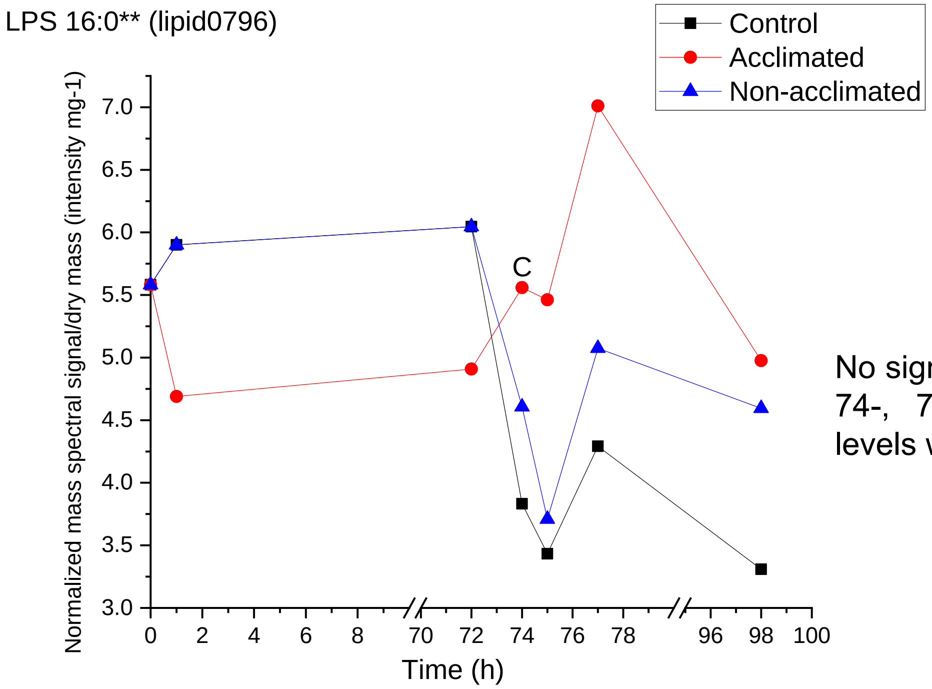

No significant correlation of 74-, 75-, and 77-h lipid levels with final ion leakage

7K

Figure S8. Time courses of levels of neutral glycerolipids in rosettes of control, non-acclimated, and acclimated plants. Treatments are shown in Figure 1. Asterisks indicate lipids with quality control (pooled sample) levels less than 0.75 (*) or 0.25 (**) units of normalized mass spectral intensity, where 1 = intensity of 1 pmol of internal standard. "C" indicates that the lipid level in non-acclimated or acclimated plants is significantly different than the control level, and "A" indicates that the lipid level in non-acclimated plants is significantly different than the level in acclimated plants (Table S2). Indicated on each plot is whether there is significant correlation of lipid level at the 74-, 75-, and 77-h time points with final (98 h) ion leakage.

Lipid number	Panel	Class, oxidation	Lipid name
lipid0283	8A	DG, non-oxidized	DG 16:0_18:3*
lipid0282	8A	DG, non-oxidized	DG 16:1_18:3**
lipid0285	8B	DG, non-oxidized	DG 18:2_18:3*
lipid0281	8B	DG, non-oxidized	DG 18:3_16:3**
lipid0286	8C	DG, non-oxidized	DG 18:3_18:1**
lipid0284	8C	DG, non-oxidized	DG 18:3_18:3*
lipid0280	8D	TG, non-oxidized	TG 18:2_36:5
lipid0276	8D	TG, non-oxidized	TG 18:3_34:2*
lipid0278	8E	TG, non-oxidized	TG 18:3_36:5
lipid0277	8E	TG, non-oxidized	TG 18:3_36:6

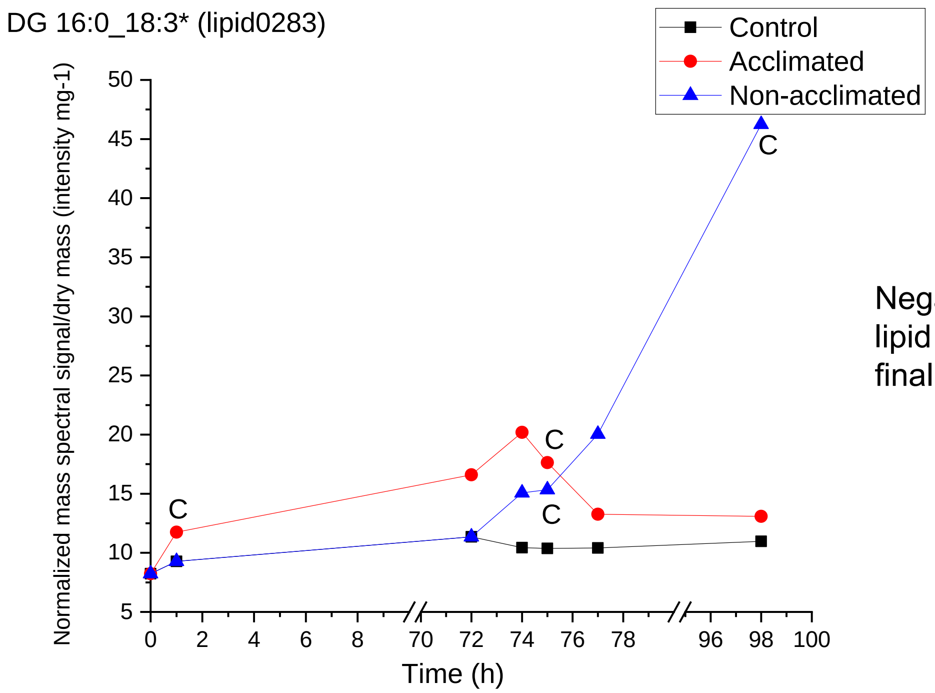

Negative correlation of lipid level at 75 h with final ion leakage

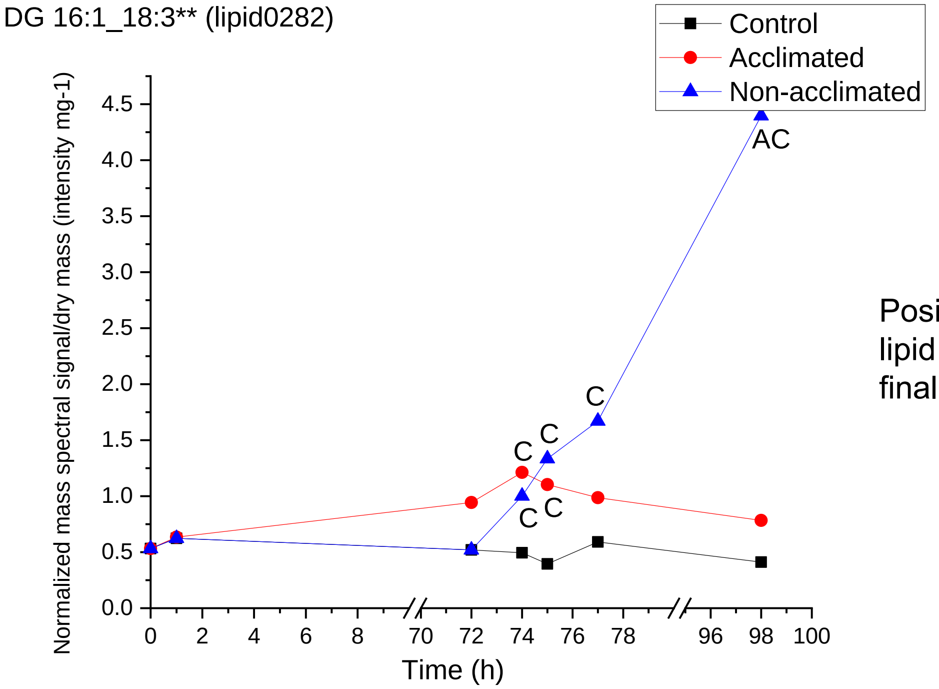

Positive correlation of lipid level at 77 h with final ion leakage

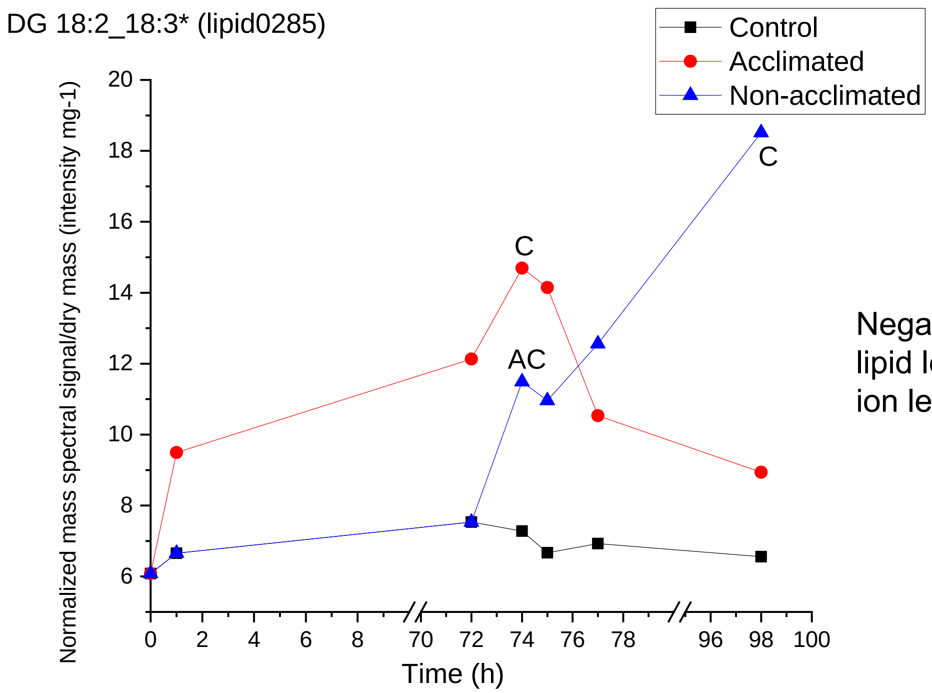

Negative correlation of lipid level at 75 h with final ion leakage

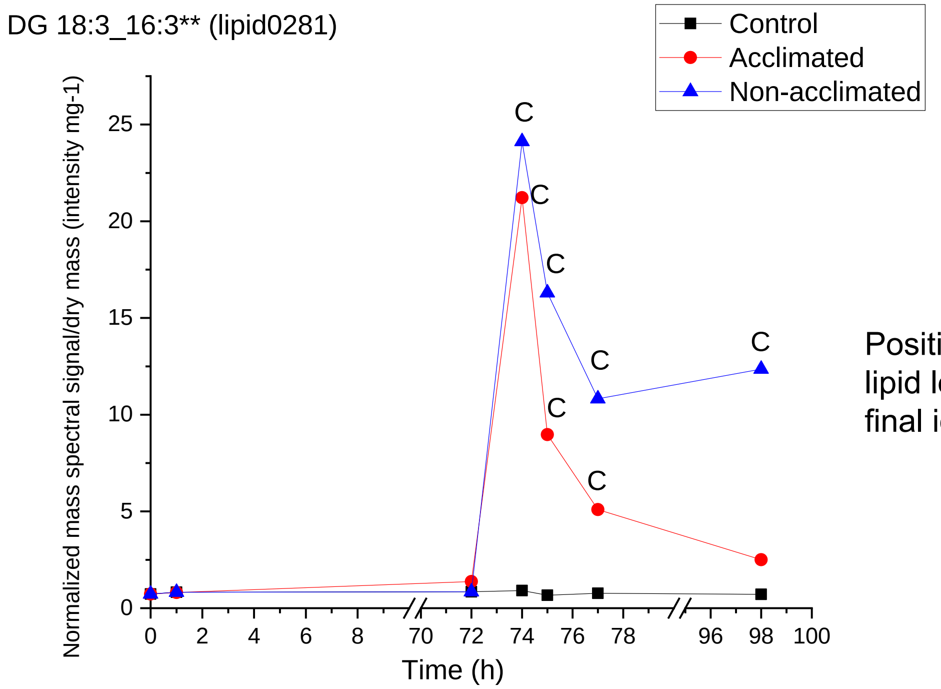

Positive correlation of lipid level at 77 h with final ion leakage

8B

DG 18:3_18:1** (lipid0286)

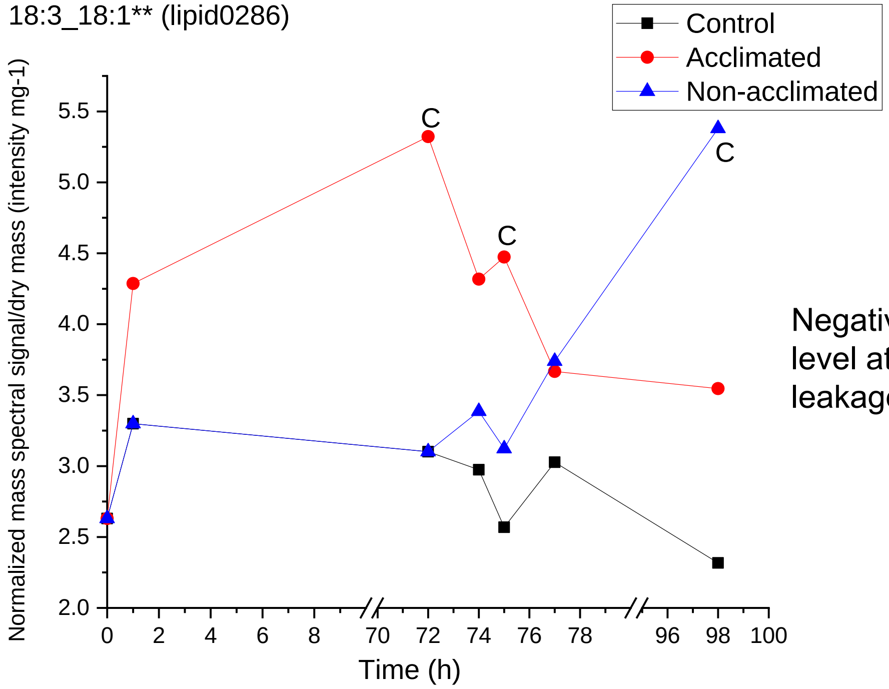

Negative correlation of lipid level at 75 h with final ion leakage

DG 18:3_18:3* (lipid0284)

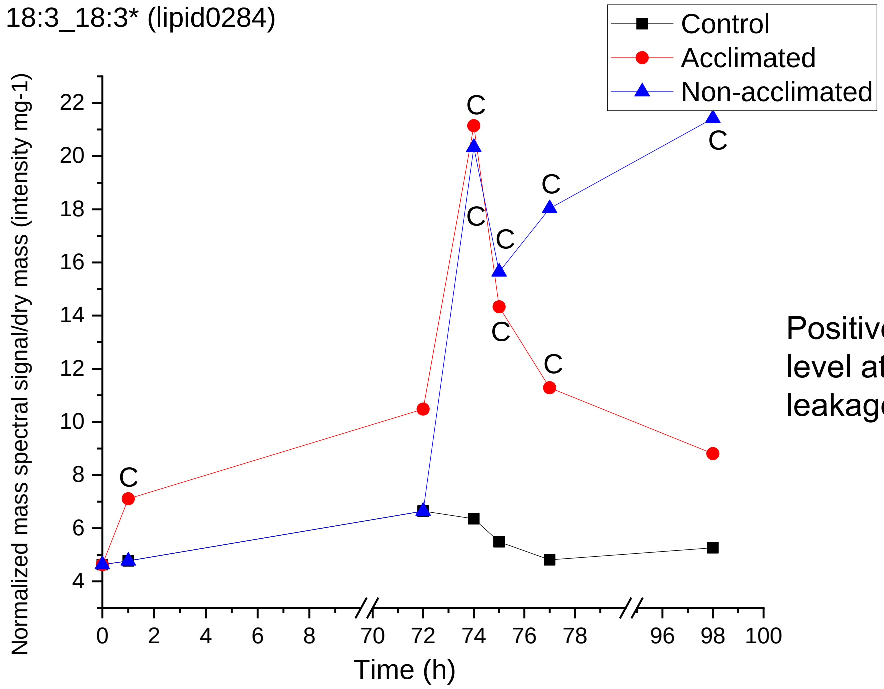

Positive correlation of lipid level at 77 h with final ion leakage

8C

TG 18:2_36:5 (lipid0280)

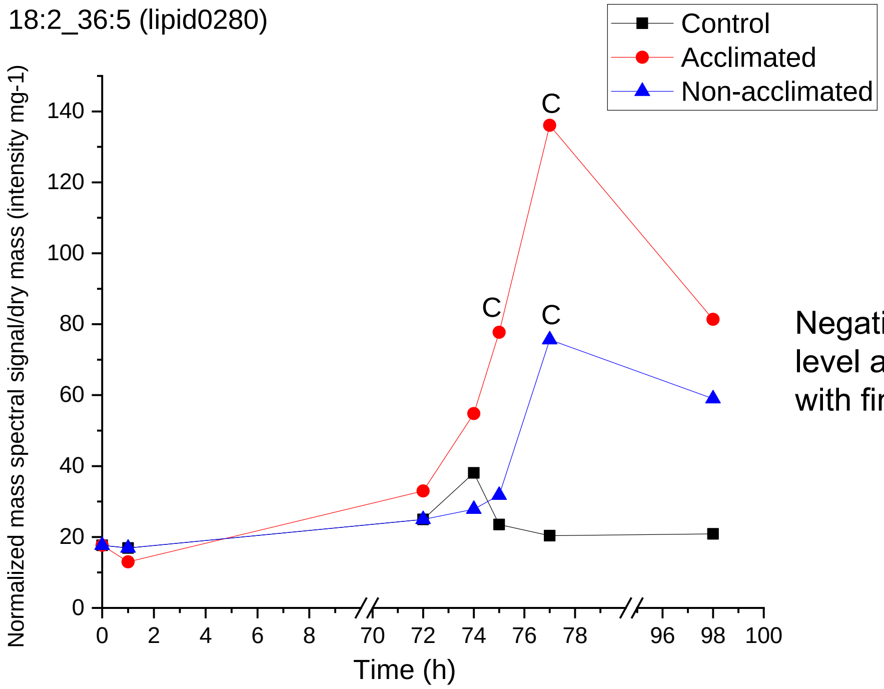

Negative correlation of lipid level at 74, 75, and 77 h with final ion leakage

TG 18:3_34:2* (lipid0276)

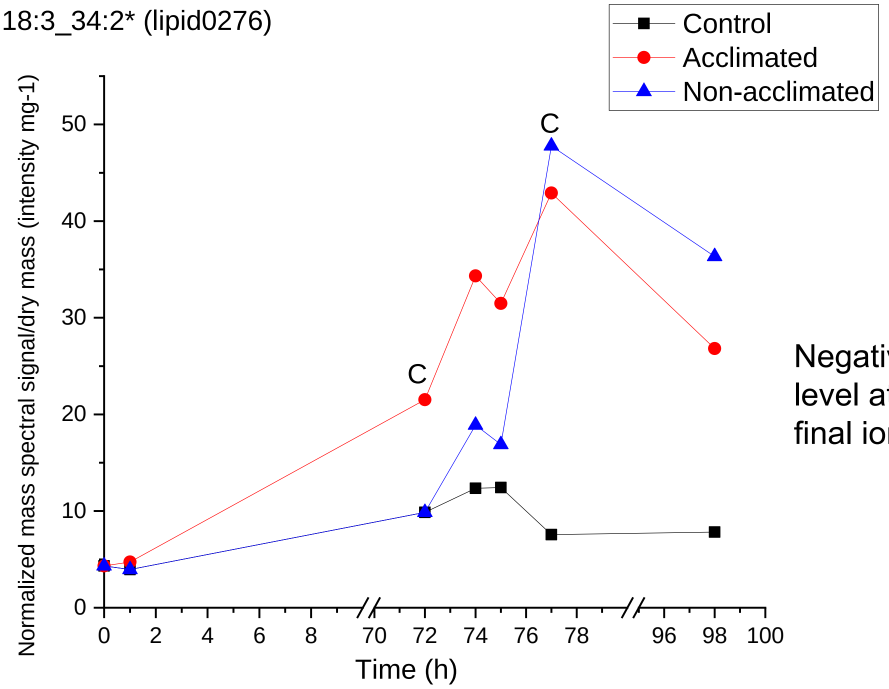

Negative correlation of lipid level at 74 and 75 h with final ion leakage

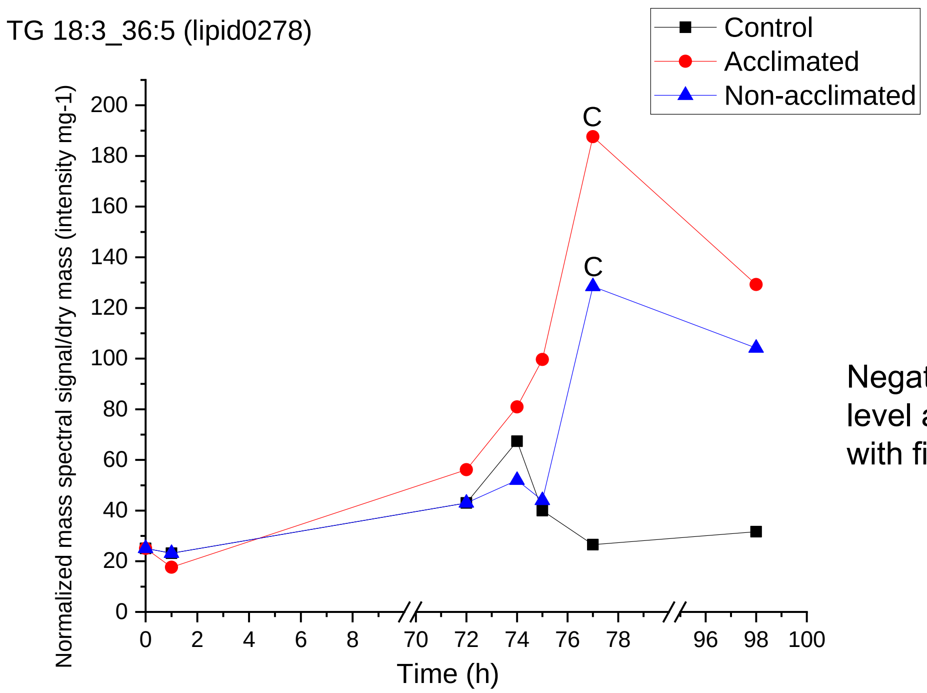

Negative correlation of lipid level at 74, 75, and 77 h with final ion leakage

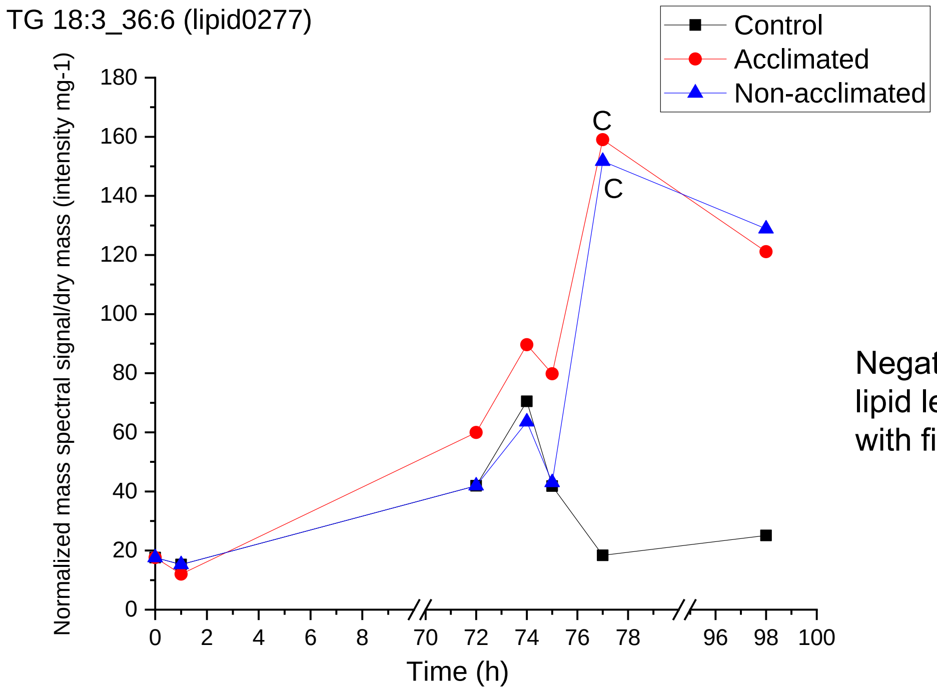

Negative correlation of lipid level at 74 and 75 h with final ion leakage

8E

Figure S9. Time courses of levels of selected sterol derivatives in rosettes of control, non-acclimated, and acclimated plants. Treatments are shown in Figure 1. Asterisks indicate lipids with quality control (pooled sample) levels less than 0.75 (*) or 0.25 (**) units of normalized mass spectral intensity, where 1 = intensity of 1 pmol of internal standard. "C" indicates that the lipid level in non-acclimated or acclimated plants is significantly different than the control level, and "A" indicates that the lipid level in non-acclimated plants is significantly different than the level in acclimated plants (Table S2). Indicated on each plot is whether there is significant correlation of lipid level at the 74-, 75-, and 77-h time points with final (98 h) ion leakage.

Lipid number	Panel	Class, oxidation	Lipid name
lipid0255	9A	Acyl sterol hexoside, non-oxidized	Campesterol-Hex 16:0
lipid0258	9A	Acyl sterol hexoside, non-oxidized	Campesterol-Hex 18:3
lipid0257	9B	Acyl sterol hexoside, non-oxidized	Sitosterol-Hex 16:0
lipid0263	9B	Acyl sterol hexoside, non-oxidized	Sitosterol-Hex 18:2
lipid0260	9C	Acyl sterol hexoside, non-oxidized	Sitosterol-Hex 18:3
lipid0256	9C	Acyl sterol hexoside, non-oxidized	Stigmasterol-Hex 16:0
lipid0259	9D	Acyl sterol hexoside, non-oxidized	Stigmasterol-Hex 18:3
lipid0816	9D	Acyl sterol hexoside, oxidized	Campesterol-Hex 18:4;O
lipid0818	9E	Acyl sterol hexoside, oxidized	Sitosterol-Hex 18:4;O
lipid0817	9E	Acyl sterol hexoside, oxidized	Stigmasterol-Hex 18:4;O
lipid0287	9F	Sterol ester, non-oxidized	Campesterol 18:3**
lipid0821	9F	Sterol ester, non-oxidized	Sitosterol 18:2*
lipid0289	9G	Sterol ester, non-oxidized	Sitosterol 18:3*
lipid0825	9G	Sterol ester, oxidized	Campesterol 18:4;O**
lipid0827	9H	Sterol ester, oxidized	Sitosterol 18:4;O**
lipid0826	9H	Sterol ester, oxidized	Stigmasterol 18:4;O**

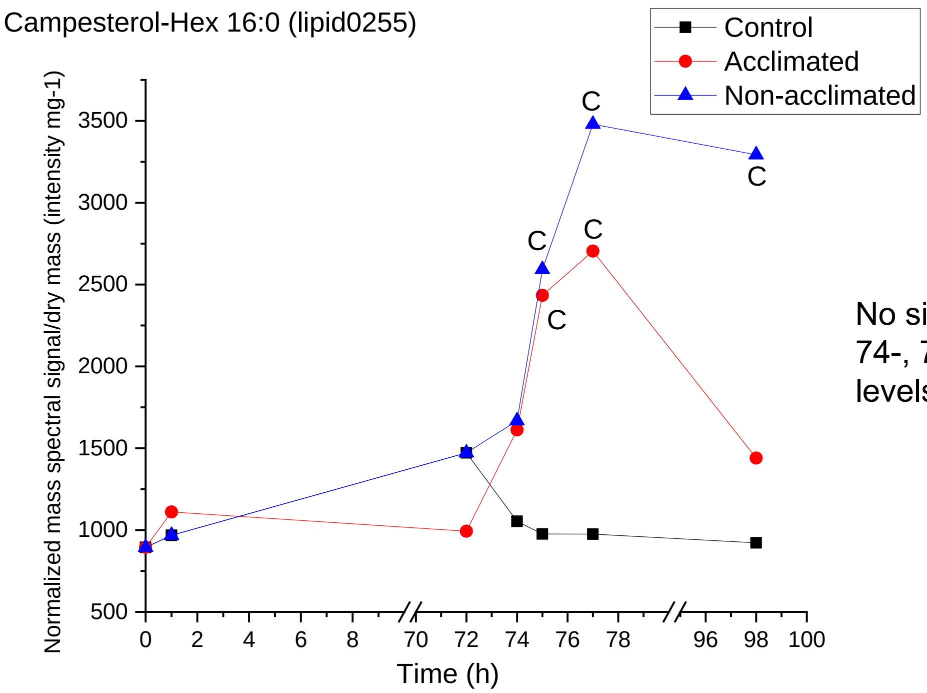

No significant correlation of 74-, 75-, and 77-h lipid levels with final ion leakage

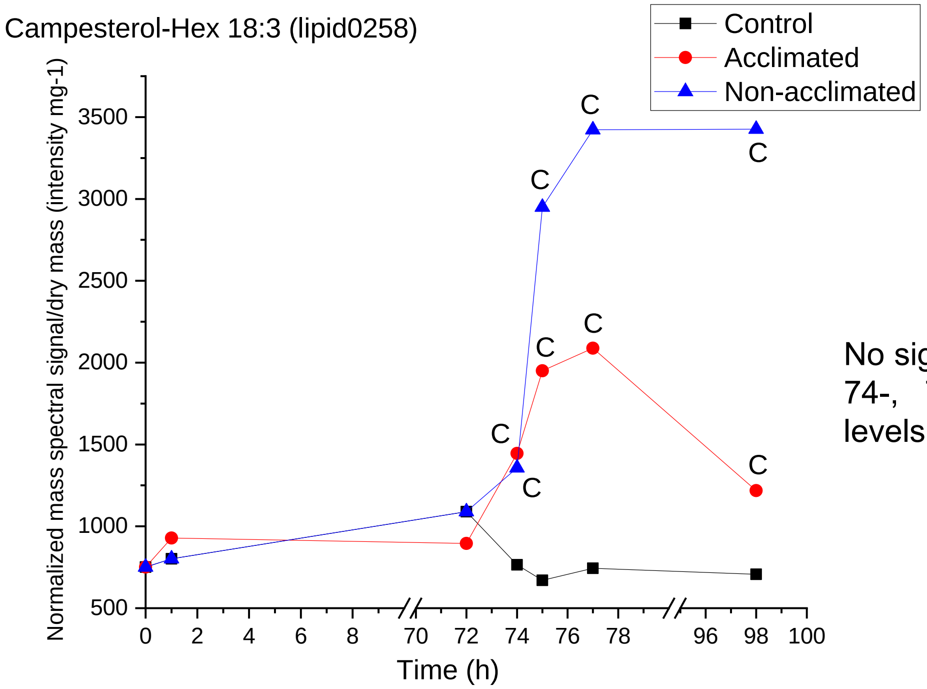

No significant correlation of 74-, 75-, and 77-h lipid levels with final ion leakage

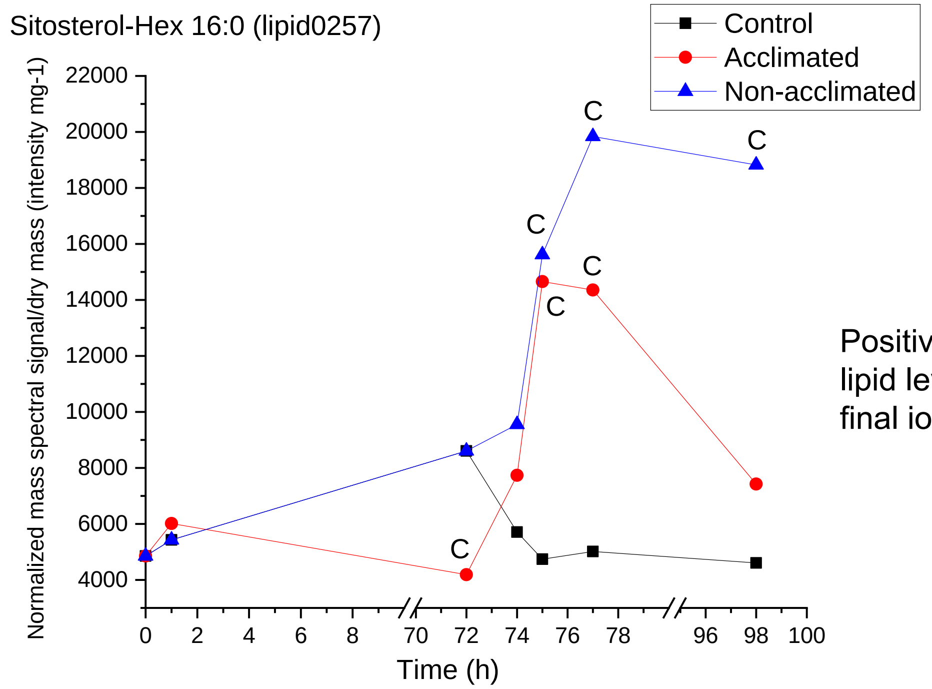

Positive correlation of lipid level at 74 h with final ion leakage

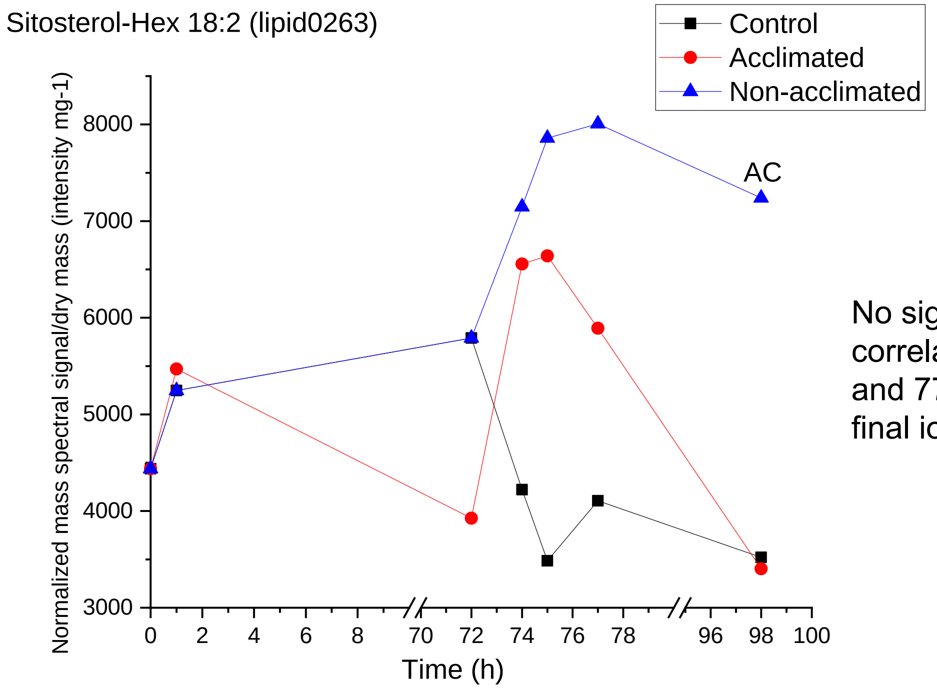

No significant correlation of 74-, 75-, and 77-h lipid levels with final ion leakage

9B

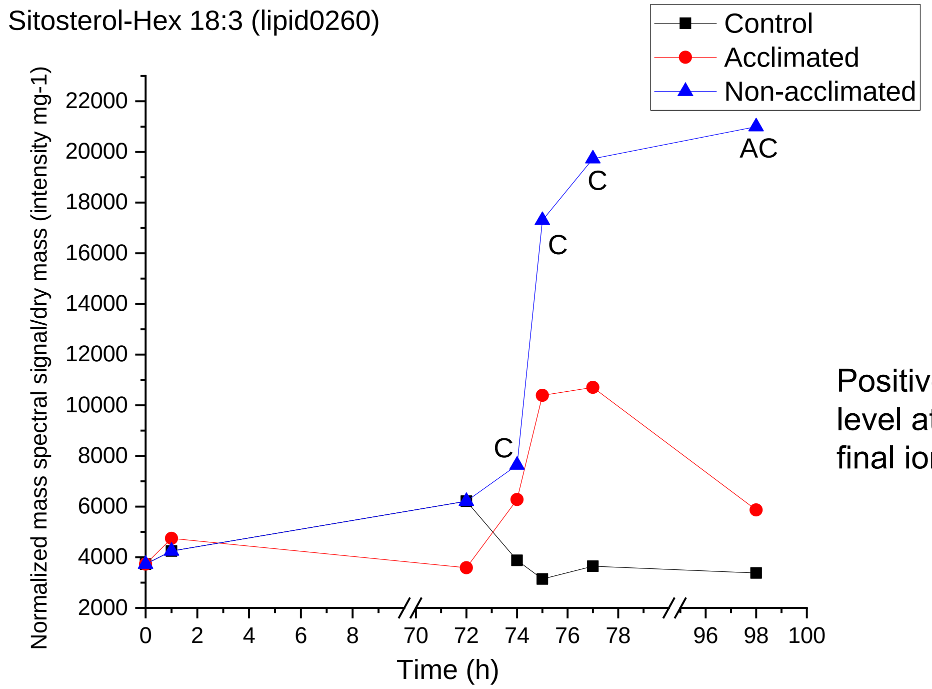

Positive correlation of lipid level at 74 and 77 h with final ion leakage

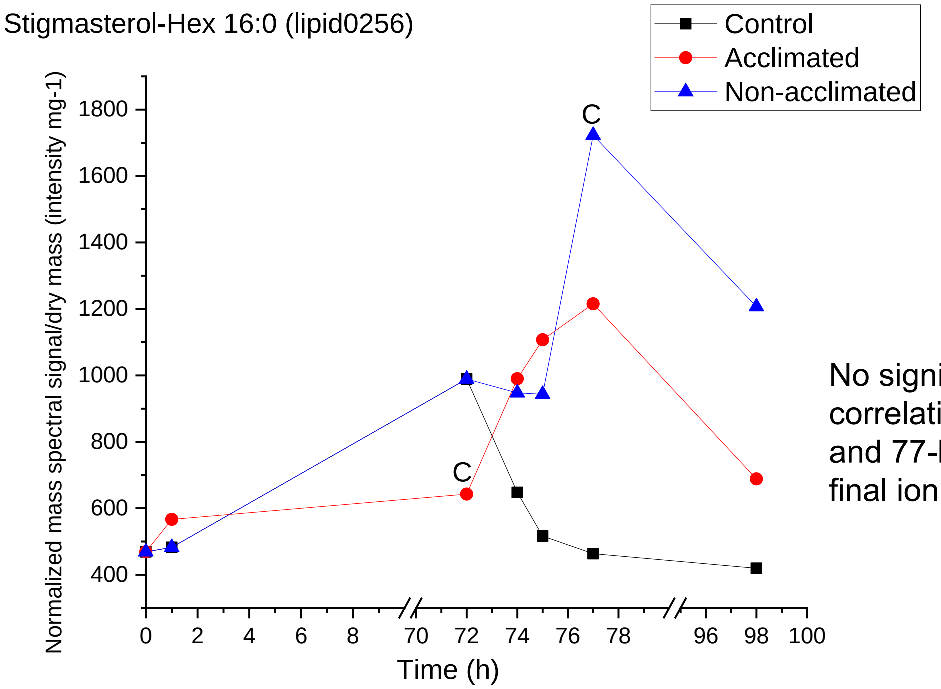

No significant correlation of 74-, 75-, and 77-h lipid levels with final ion leakage

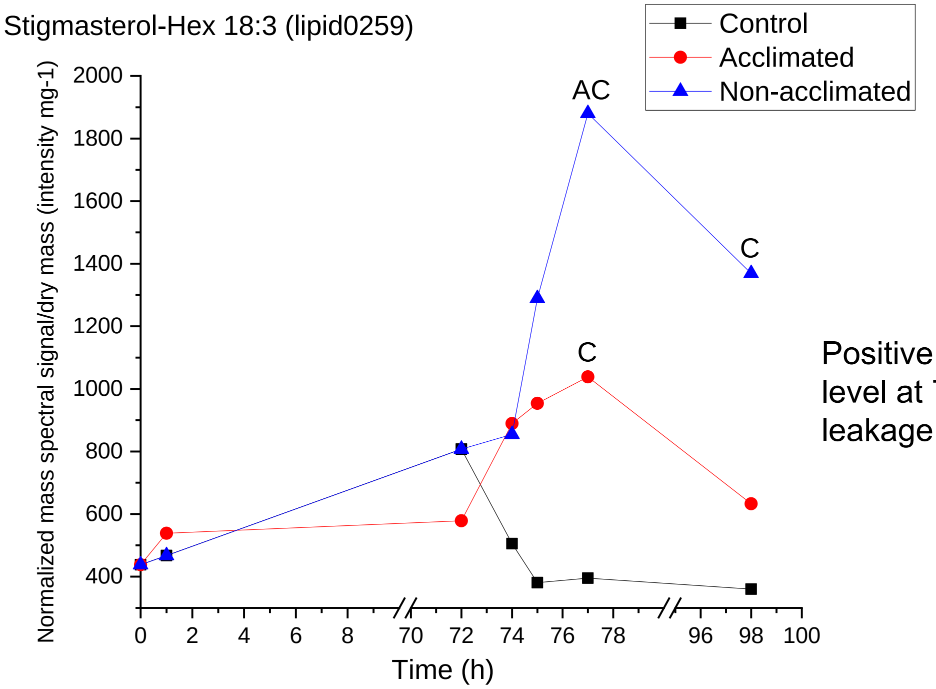

Positive correlation of lipid level at 77 h with final ion leakage

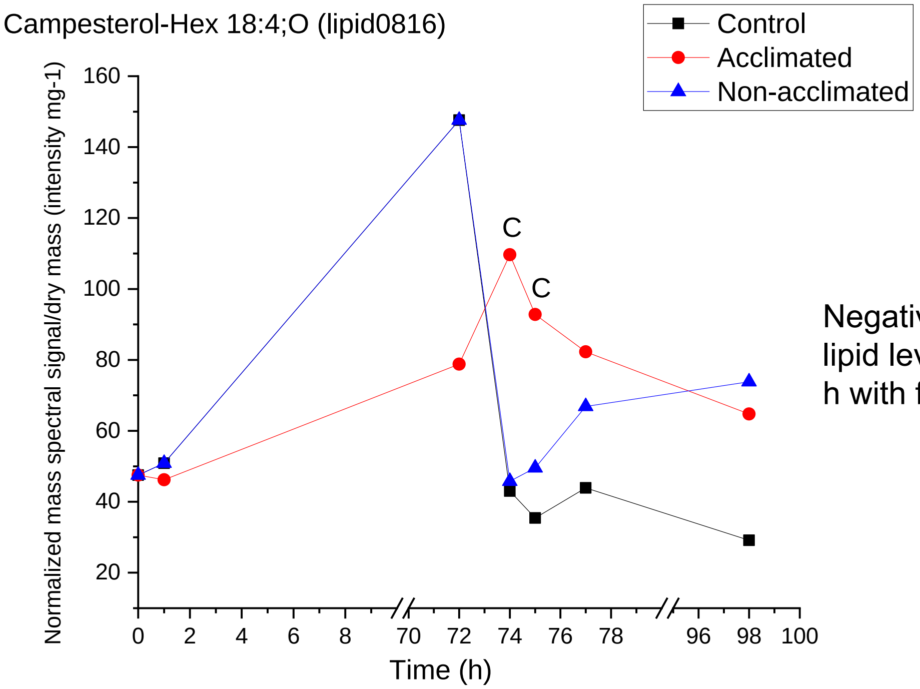

Negative correlation of lipid level at 74, 75, and 77 h with final ion leakage

9D

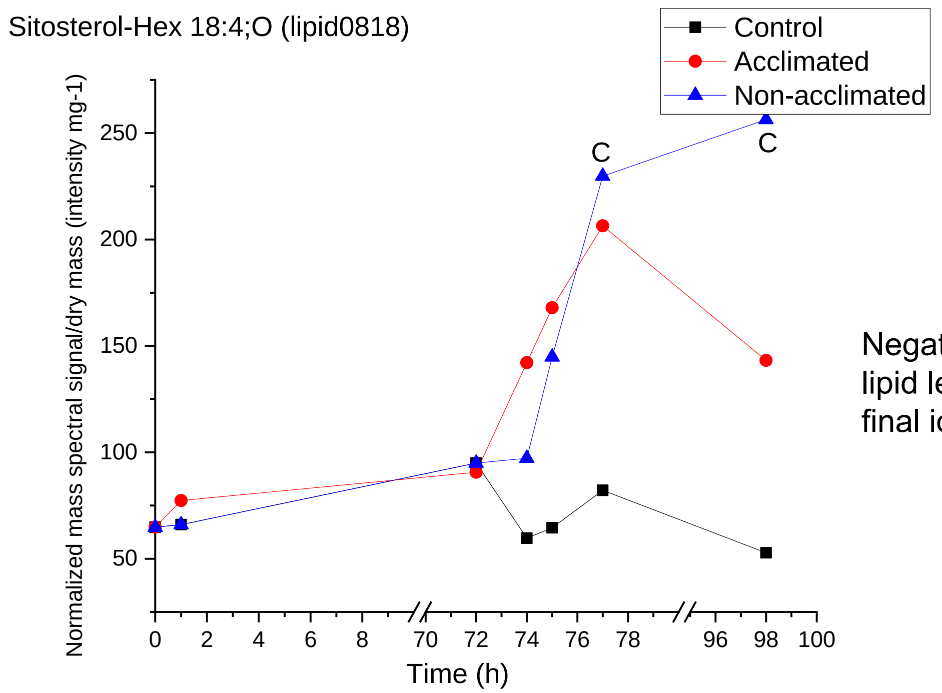

Negative correlation of lipid level at 75 h with final ion leakage

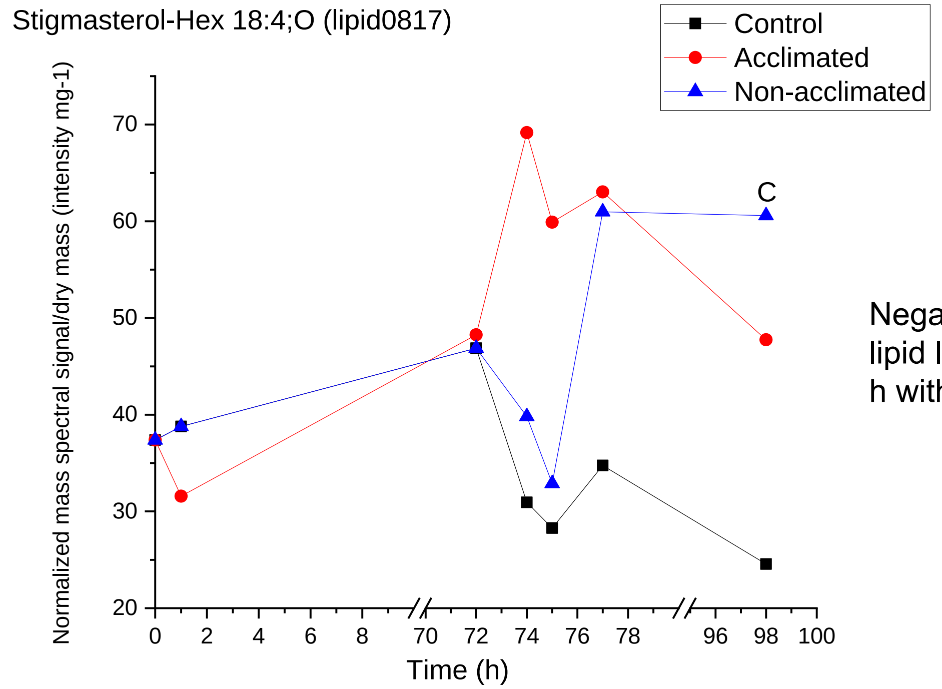

Negative correlation of lipid level at 74 and 75 h with final ion leakage

Campesterol 18:3** (lipid0287)

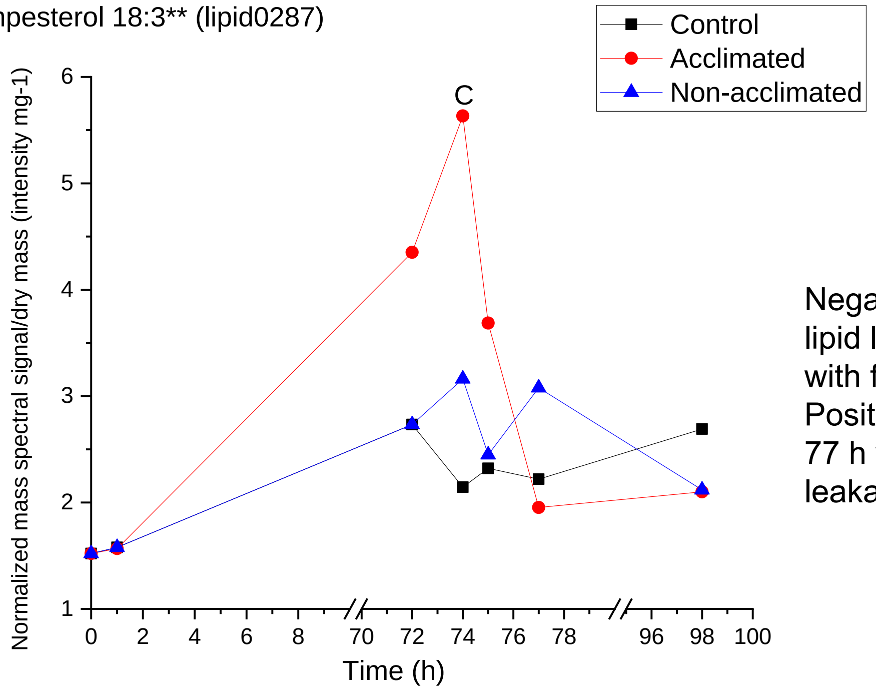

Negative correlation of lipid level at 74 and 75 h with final ion leakage
Positive correlation at 77 h with final ion leakage

Sitosterol 18:2* (lipid0821)

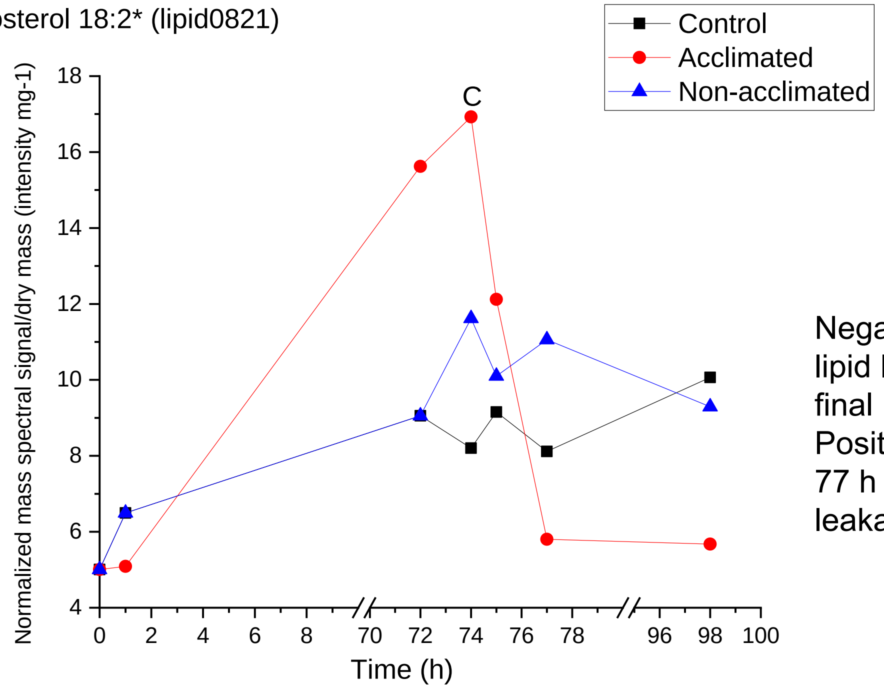

Negative correlation of lipid level at 74 h with final ion leakage
Positive correlation at 77 h with final ion leakage

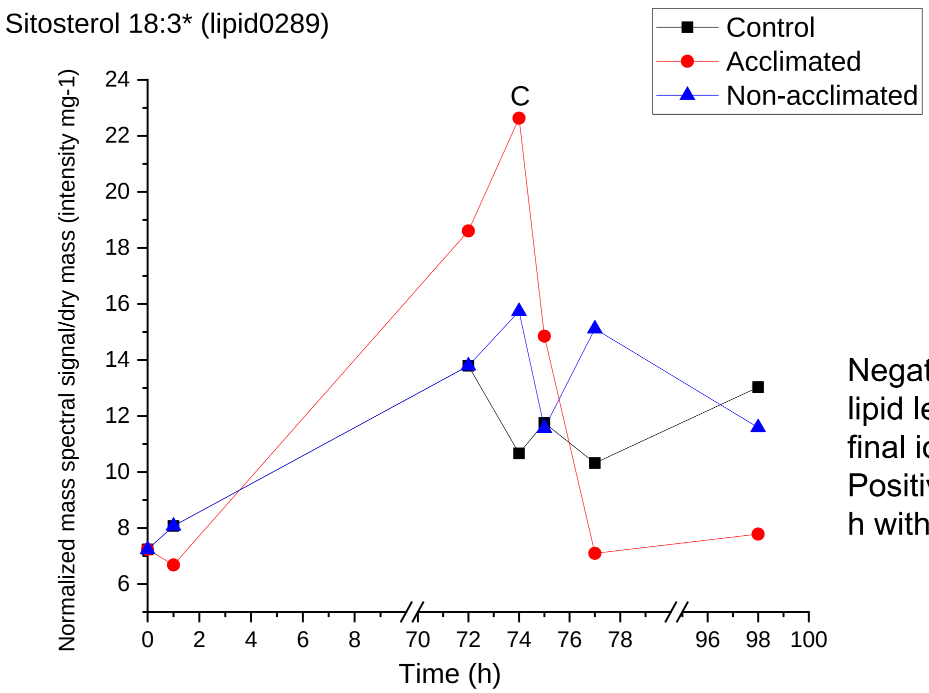

Negative correlation of lipid level at 74 h with final ion leakage
Positive correlation at 77 h with final ion leakage

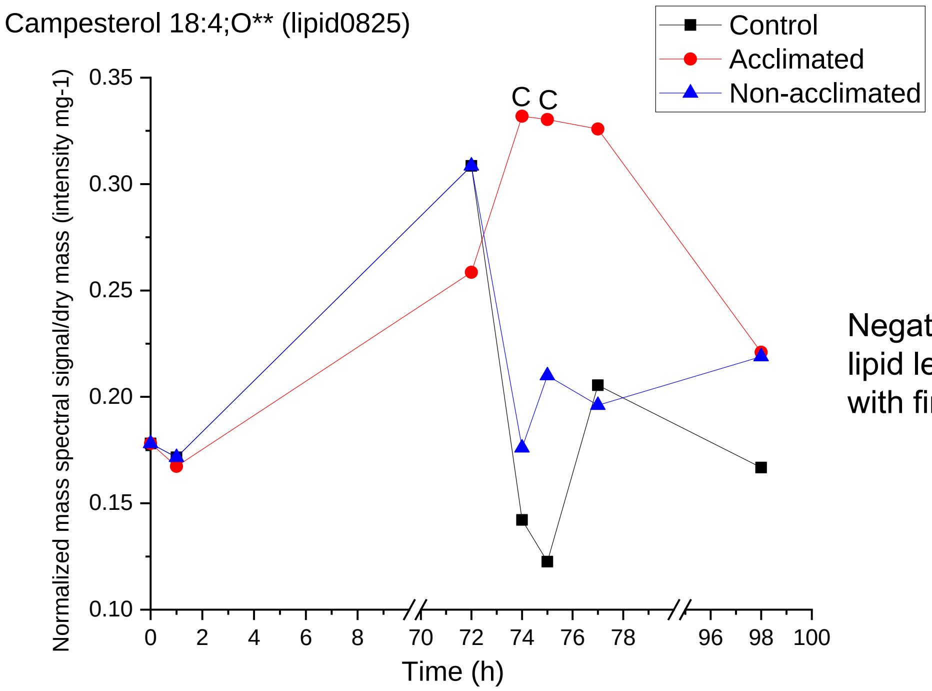

Negative correlation of lipid level at 74 and 75 h with final ion leakage

9G

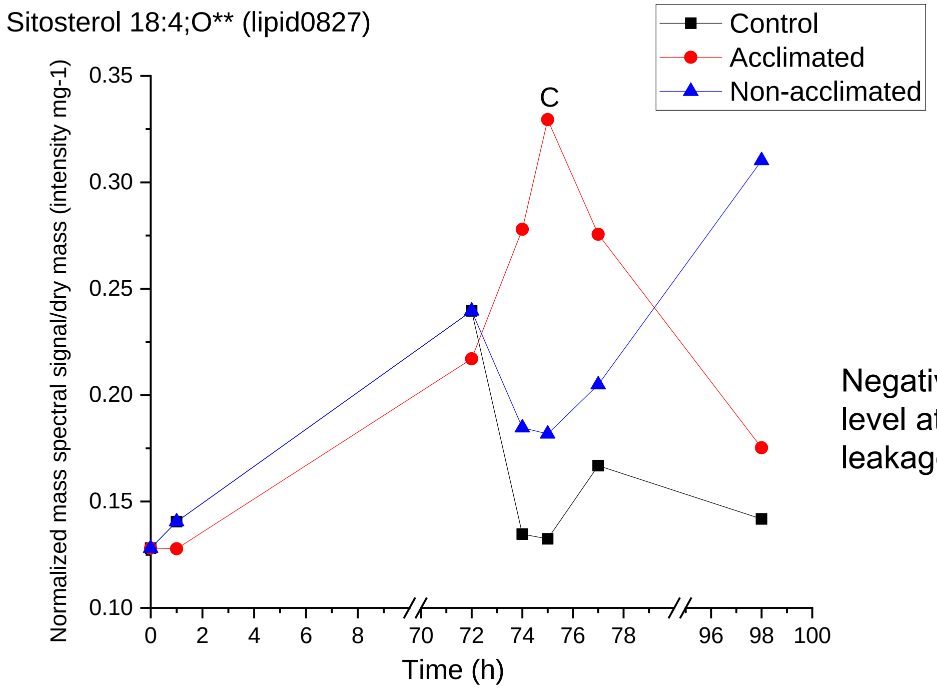

Negative correlation of lipid level at 75 h with final ion leakage

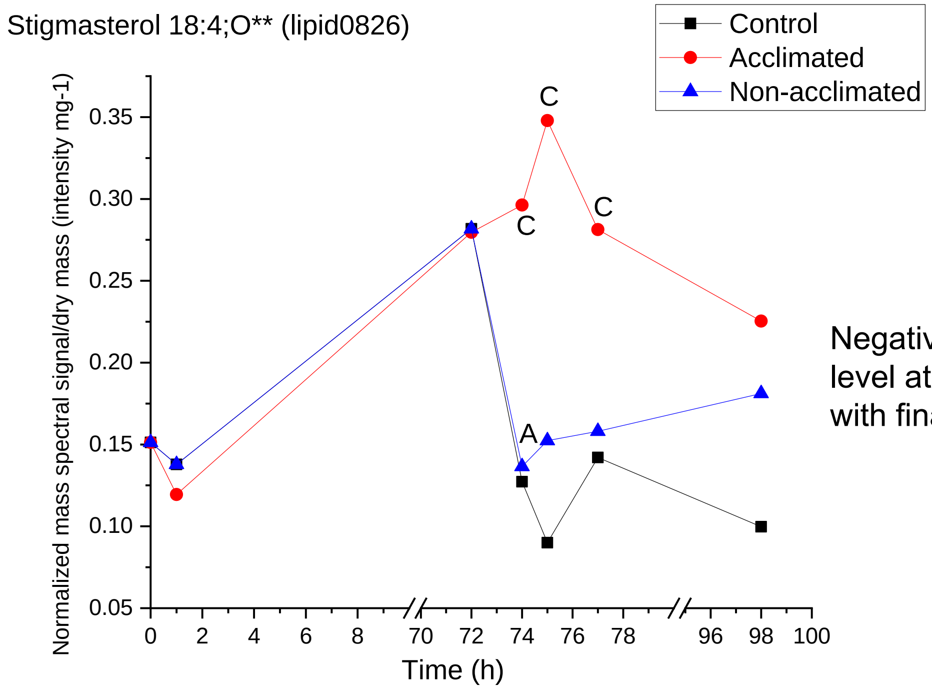

Negative correlation of lipid level at 74, 75, and 77 h with final ion leakage

9H

Figure S10. Time courses of levels of ion leakage and selected lipids in rosettes of control, non-acclimated, and acclimated wild-type and *opr3* plants. Treatments are shown in Figure 1. Wild-type plants are indicated by "WT" after the treatment, and mutants are indicated by "opr3". Asterisks in lipid names indicate lipids with quality control (pooled sample) levels less than 0.75 (*) or 0.25 (**) units of normalized mass spectral intensity, where 1 = intensity of 1 pmol of internal standard. Asterisks on the graphs indicate that the measured value is significantly different for the *opr3* mutant compared to wild-type plants that underwent the same treatment. Abbreviation: lysoPA (LPA).

Lipid number	Panel	Measured parameter (new Lipid Maps nomenclature or similar)
	10A	Ion leakage
**Oxidized, diacyl plastidic lipids containing OPDA**		
lipid0323	10B	MGDG 18:3_16:4;O
lipid0358	10B	MGDG 18:4;O_16:3
lipid0325	10C	MGDG 18:4;O_16:4;O (16:4;O as fragment; Arabidopside A)
lipid0361	10C	MGDG 18:4;O_16:4;O (18:4;O as fragment; Arabidopside A)
lipid0375	10D	MGDG 18:4;O_18:4;O (Arabidopside B)*
lipid0356	10D	PG 18:4;O_16:1
**Oxidized, head group-acylated plastidic lipids containing OPDA**		
lipid0603	10E	DGDG-O(FA 18:4;O) 36:8;O2
lipid0535	10E	MGDG-O(FA 18:4;O) 34:8;O2 (Arabidopside E or MGDG-O(FA 18:4;O) 36:6)
lipid0540	10F	MGDG-O(FA 18:4;O) 36:8;O2 (Arabidopside G)
lipid0574	10F	MGDG-O(FA 18:4;O) 36:8;O2 (alternative fragmentation; Arabidopside G)
lipid0489	10G	MGDG-O(FA 16:4;O) 34:8;O2 or MGDG-O(FA 16:4;O) 36:6
lipid0483	10G	MGDG-O(FA 16:0) 36:8;O2
lipid0512	10H	MGDG-O(FA 18:3) 36:8;O2
lipid0518	10H	MGDG-O(FA 18:2) 36:8;O2
lipid0537	10I	MGDG-O(FA 18:4;O) 36:7;O

lipid0548	10I	MGDG-O(FA 18:3;O) 36:8;O2
lipid0556	10J	MGDG-O(FA 18:5;O2) 36:6 or MGDG-O(FA 18:5;O2) 34:8;O2
lipid0570	10J	MGDG-O(FA 18:3;O2) 36:6 or MGDG-O(FA 18:3;O2) 34:8;O2
lipid0580	10K	MGDG-O(FA 18:3;O2) 36:8;O2
lipid0588	10K	MGDG-O(FA 18:4;O3) 36:6 or MGDG-O(FA 18:4;O3) 34:8;O2
**Sterol esters**		
lipid0287	10L	Campesterol 18:3**
lipid0819	10L	Campesterol 18:2**
lipid0289	10M	Sitosterol 18:3*
lipid0821	10M	Sitosterol 18:2*
lipid0827	10N	Sitosterol 18:4;O**
lipid0288	10N	Stigmasterol 18:3**
lipid0820	10O	Stigmasterol 18:2**
**Miscellaneous lipids**		
lipid0162	10P	DGDG 34:5
lipid0173	10P	DGDG 38:6 or DGDG 36:8;O2
lipid0043	10Q	PC 38:5
lipid0063	10Q	PE 34:2
lipid0072	10R	PE 38:5
lipid0073	10R	PE 38:4
lipid0076	10S	PE 40:2
lipid0079	10S	PE 42:2
lipid0800	10T	LPA 18:3
lipid0276	10T	TG 18:3_34:2*

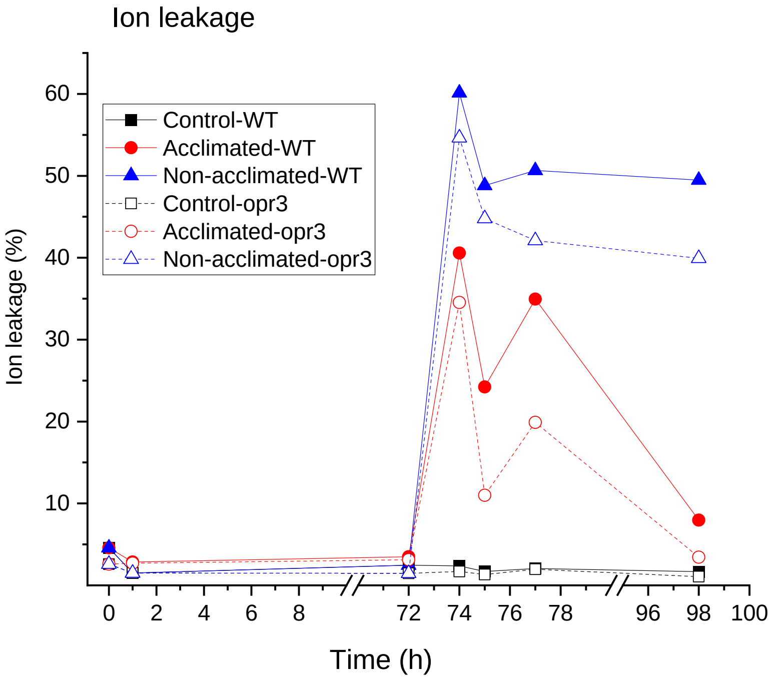

Ion leakage
Ion leakage (%)
Time (h)
Control-WT
Acclimated-WT
Non-acclimated-WT
Control-opr3
Acclimated-opr3
Non-acclimated-opr3

10A

Oxidized, diacyl plastidic lipids containing OPDA

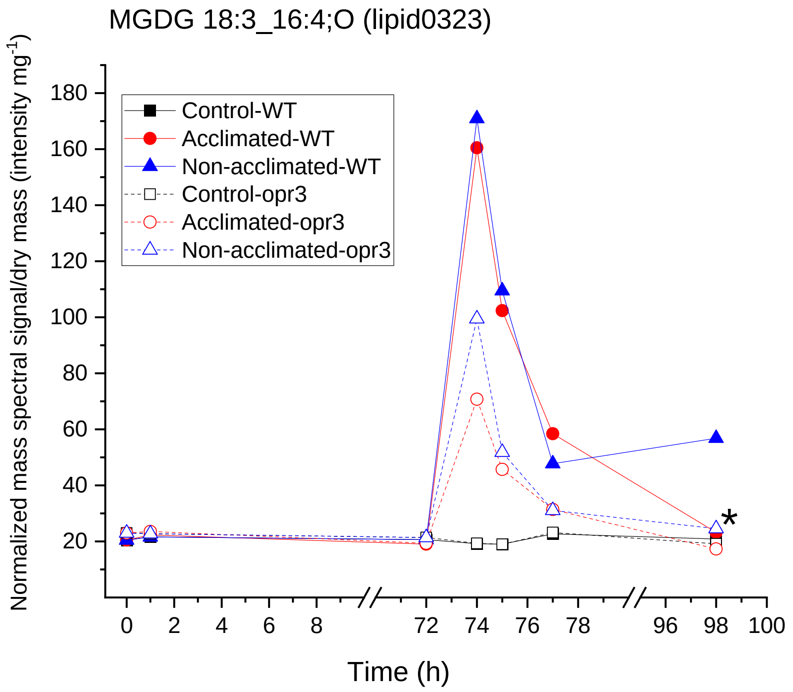

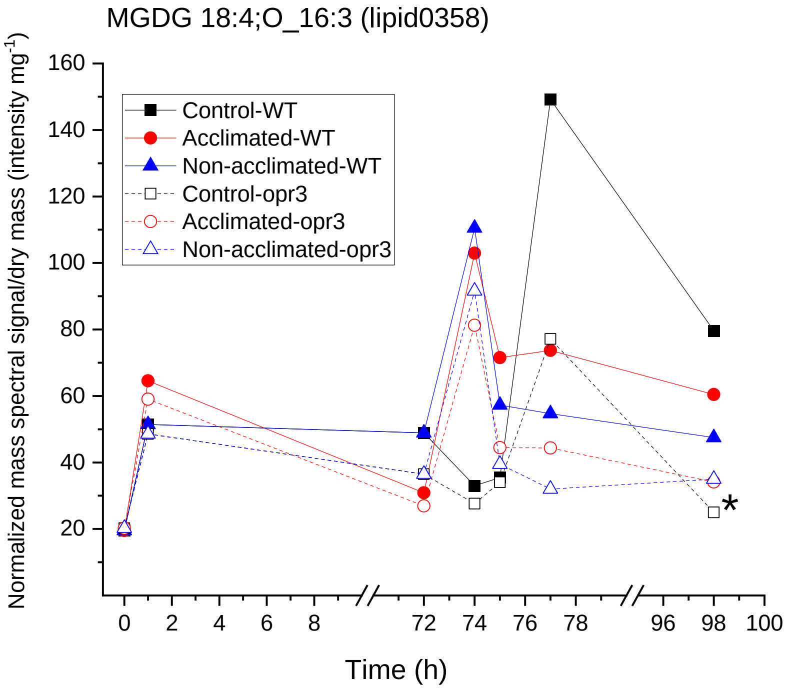

10B

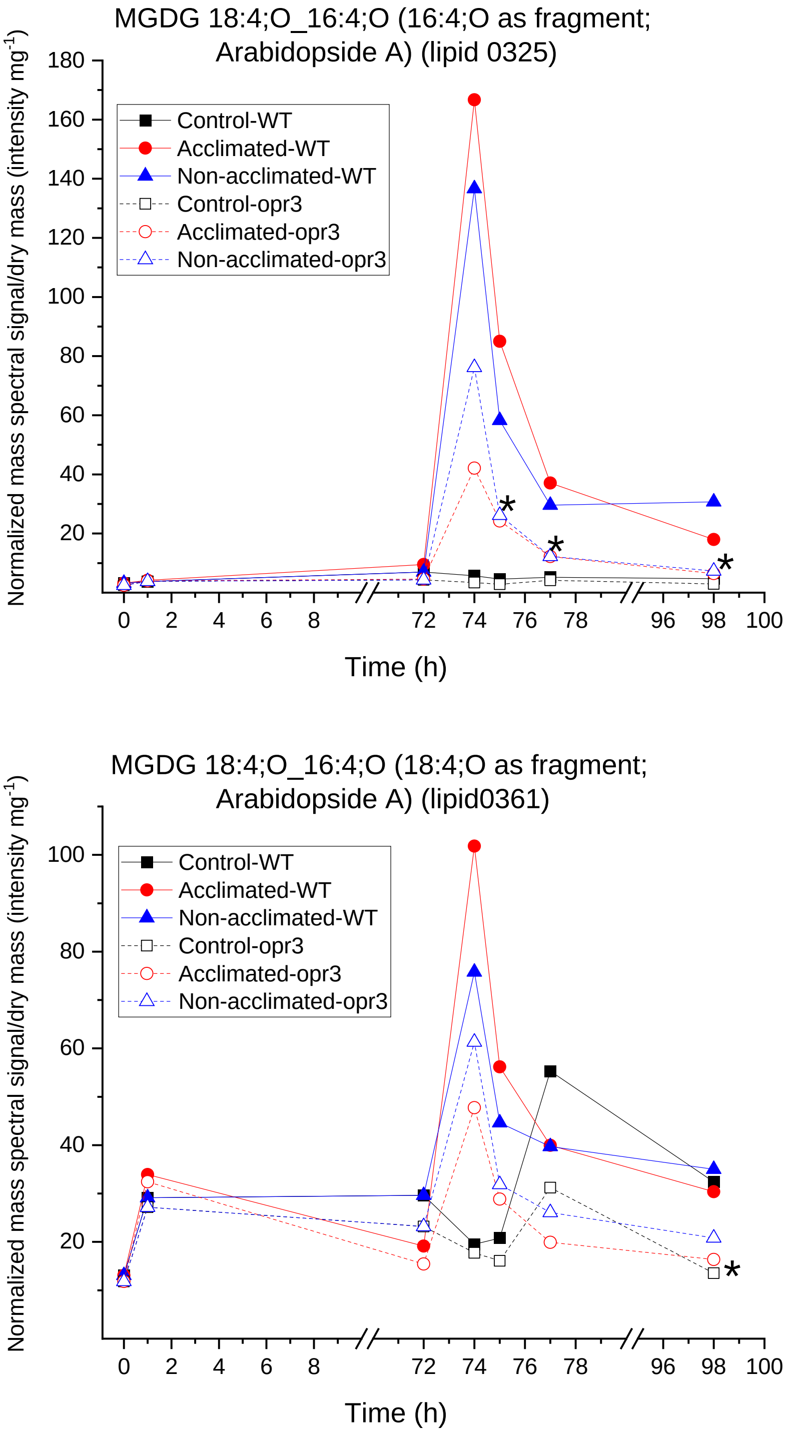
MGDG 18:4;O_16:4;O (16:4;O as fragment; Arabidopside A) (lipid 0325)
Normalized mass spectral signal/dry mass (intensity mg-1)
Control-WT
Acclimated-WT
Non-acclimated-WT
Control-opr3
Acclimated-opr3
Non-acclimated-opr3
Time (h)
MGDG 18:4;O_16:4;O (18:4;O as fragment; Arabidopside A) (lipid0361)
Normalized mass spectral signal/dry mass (intensity mg-1)
Control-WT
Acclimated-WT
Non-acclimated-WT
Control-opr3
Acclimated-opr3
Non-acclimated-opr3
Time (h)

10C

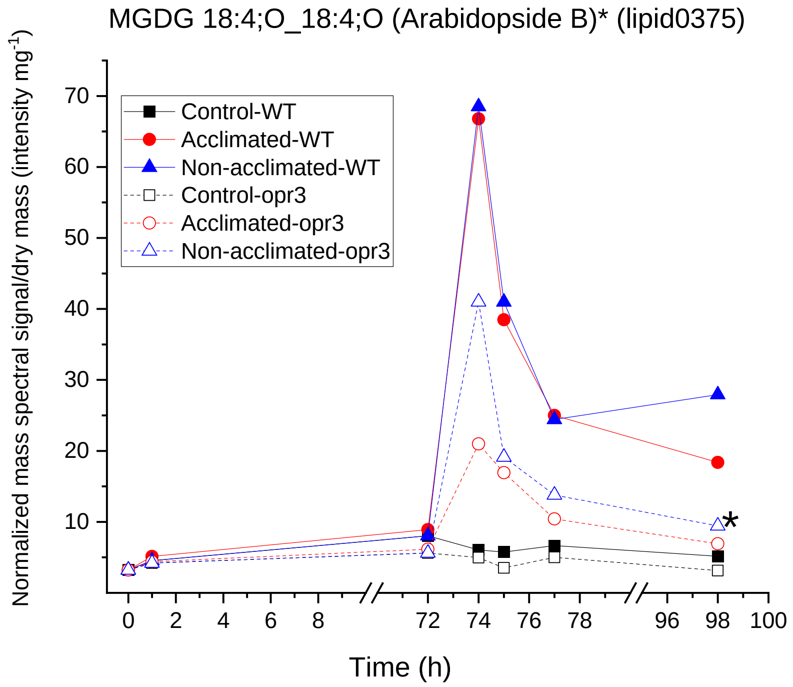
MGDG 18:4;O_18:4;O (Arabidopside B)* (lipid0375)
Normalized mass spectral signal/dry mass (intensity mg-1)
Control-WT
Acclimated-WT
Non-acclimated-WT
Control-opr3
Acclimated-opr3
Non-acclimated-opr3
*
Time (h)

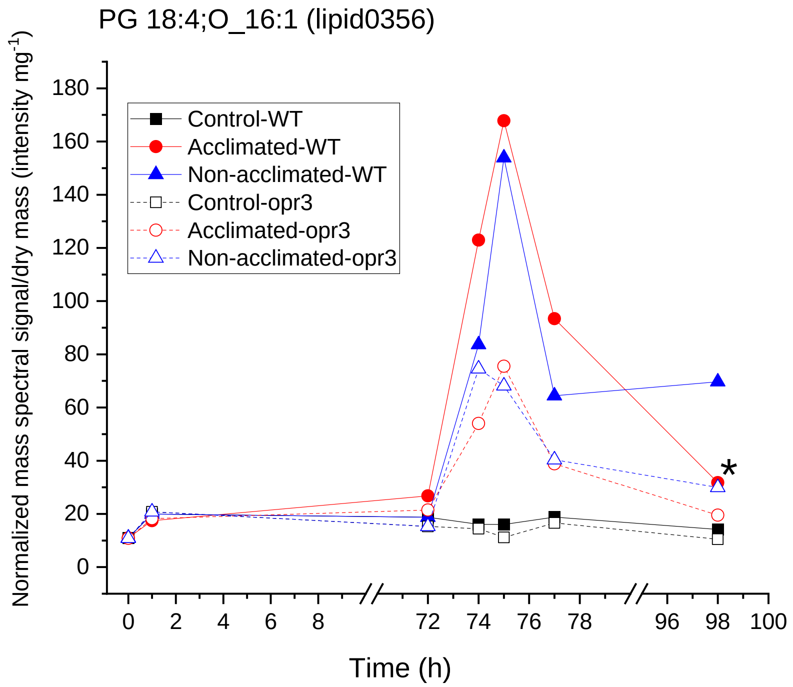
PG 18:4;O_16:1 (lipid0356)
Normalized mass spectral signal/dry mass (intensity mg-1)
Control-WT
Acclimated-WT
Non-acclimated-WT
Control-opr3
Acclimated-opr3
Non-acclimated-opr3
*
Time (h)

10D

Oxidized, head group-acylated plastidic lipids containing OPDA

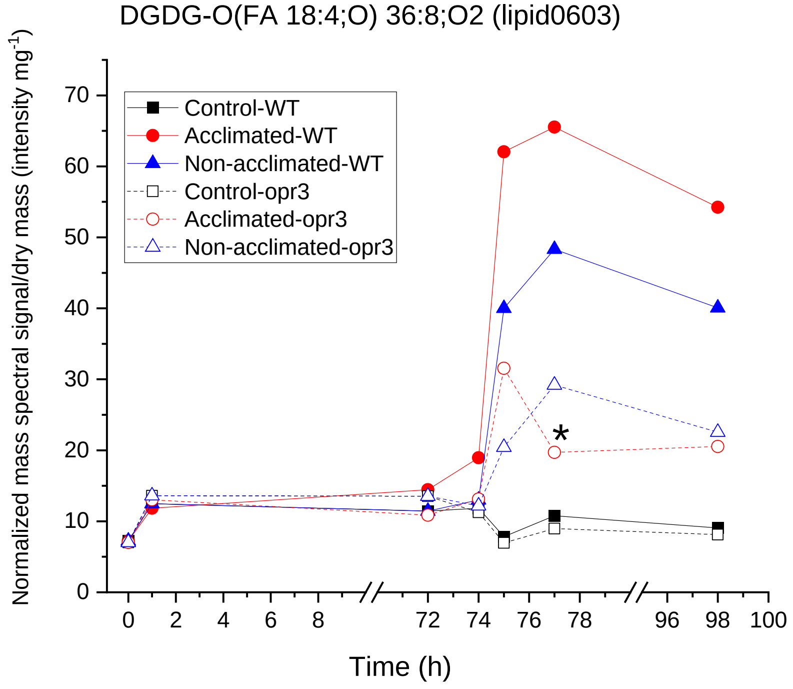

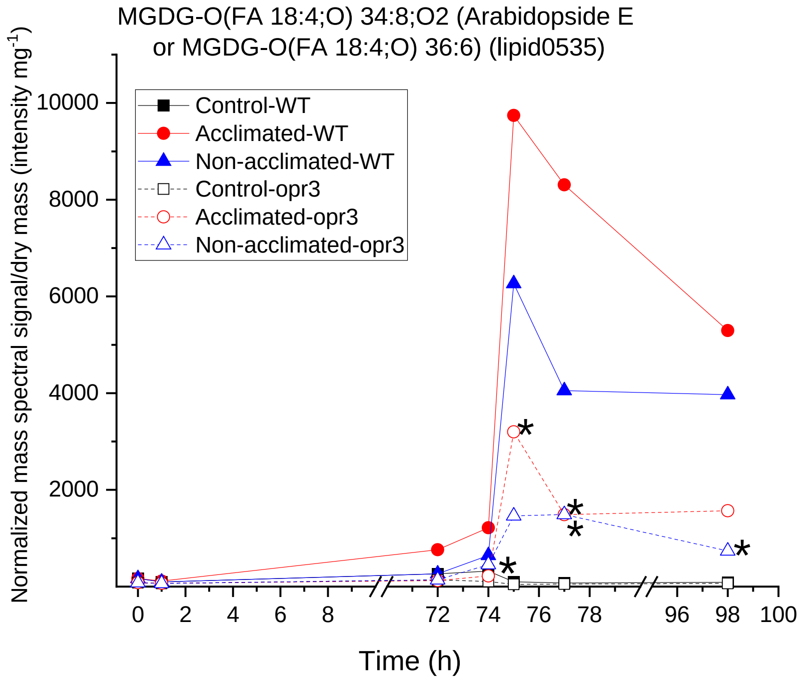

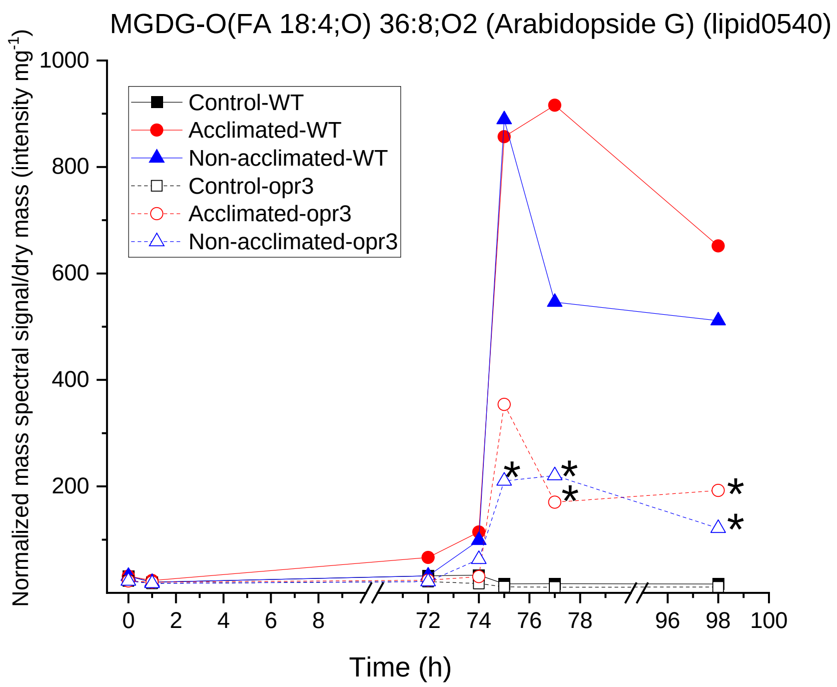
MGDG-O(FA 18:4;O) 36:8;O2 (Arabidopside G) (lipid0540)
Normalized mass spectral signal/dry mass (intensity mg-1)
Control-WT
Acclimated-WT
Non-acclimated-WT
Control-opr3
Acclimated-opr3
Non-acclimated-opr3
Time (h)

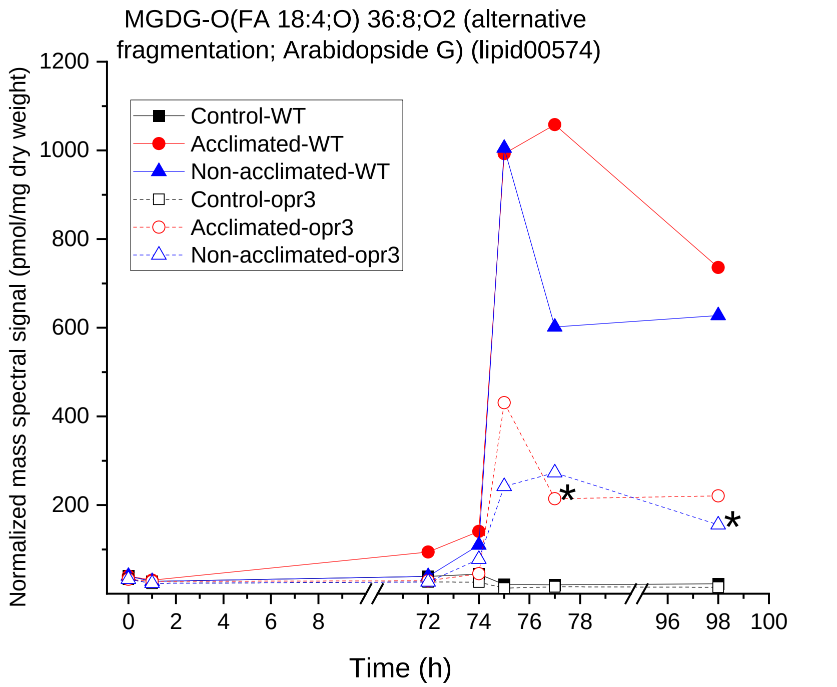
MGDG-O(FA 18:4;O) 36:8;O2 (alternative fragmentation; Arabidopside G) (lipid00574)
Normalized mass spectral signal (pmol/mg dry weight)
Control-WT
Acclimated-WT
Non-acclimated-WT
Control-opr3
Acclimated-opr3
Non-acclimated-opr3
Time (h)

10F

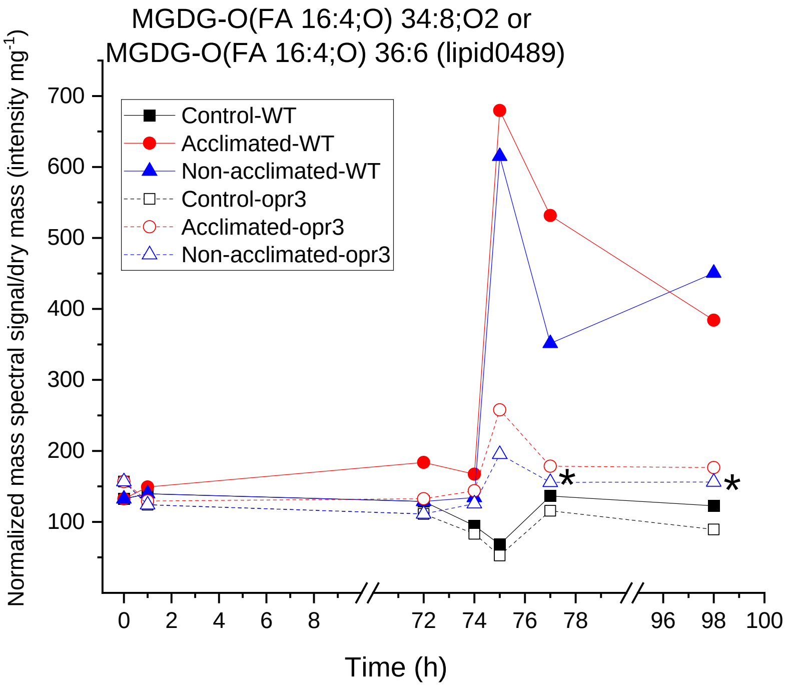
MGDG-O(FA 16:4;O) 34:8;O2 or
MGDG-O(FA 16:4;O) 36:6 (lipid0489)
Normalized mass spectral signal/dry mass (intensity mg-1)
Time (h)
Control-WT
Acclimated-WT
Non-acclimated-WT
Control-opr3
Acclimated-opr3
Non-acclimated-opr3
*
*
100
200
300
400
500
600
700
0
2
4
6
8
72
74
76
78
96
98
100

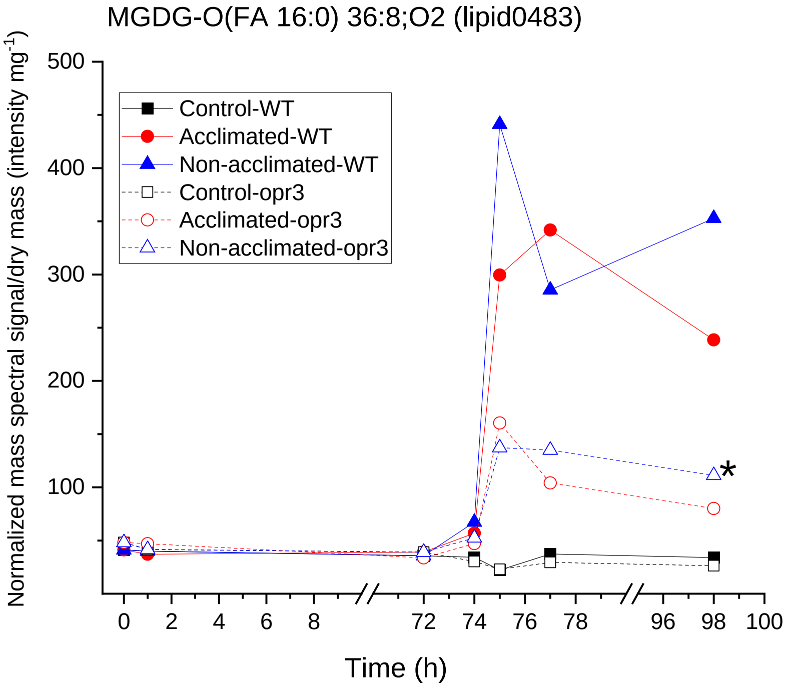
MGDG-O(FA 16:0) 36:8;O2 (lipid0483)
Normalized mass spectral signal/dry mass (intensity mg-1)
Time (h)
Control-WT
Acclimated-WT
Non-acclimated-WT
Control-opr3
Acclimated-opr3
Non-acclimated-opr3
*
100
200
300
400
500
0
2
4
6
8
72
74
76
78
96
98
100

10G

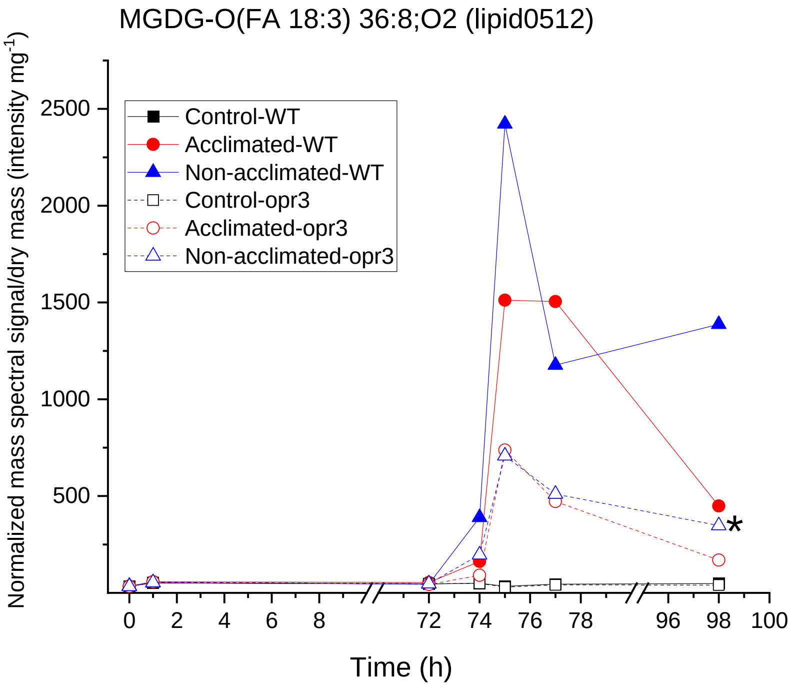
MGDG-O(FA 18:3) 36:8;O2 (lipid0512)
Normalized mass spectral signal/dry mass (intensity mg-1)
Control-WT
Acclimated-WT
Non-acclimated-WT
Control-opr3
Acclimated-opr3
Non-acclimated-opr3
*
Time (h)

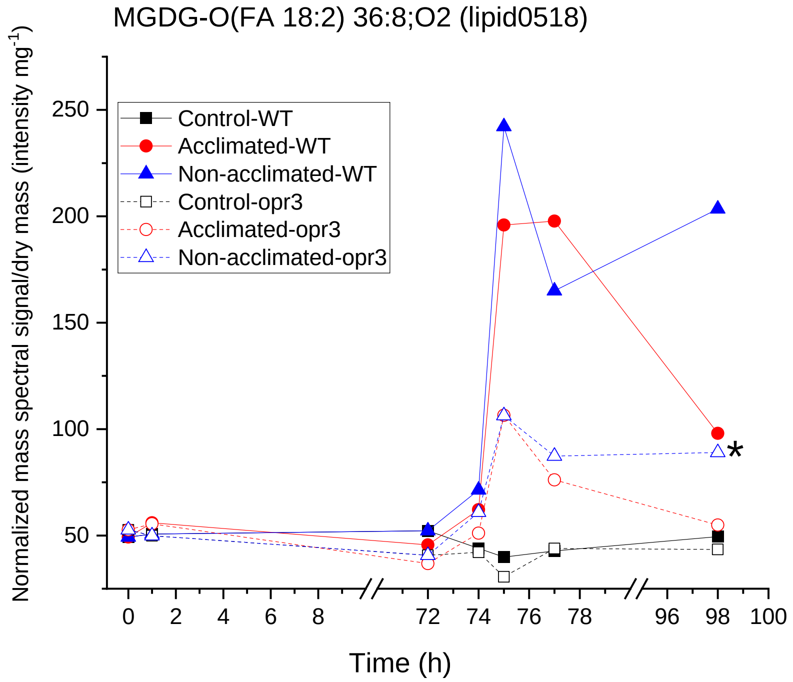
MGDG-O(FA 18:2) 36:8;O2 (lipid0518)
Normalized mass spectral signal/dry mass (intensity mg-1)
Control-WT
Acclimated-WT
Non-acclimated-WT
Control-opr3
Acclimated-opr3
Non-acclimated-opr3
*
Time (h)

10H

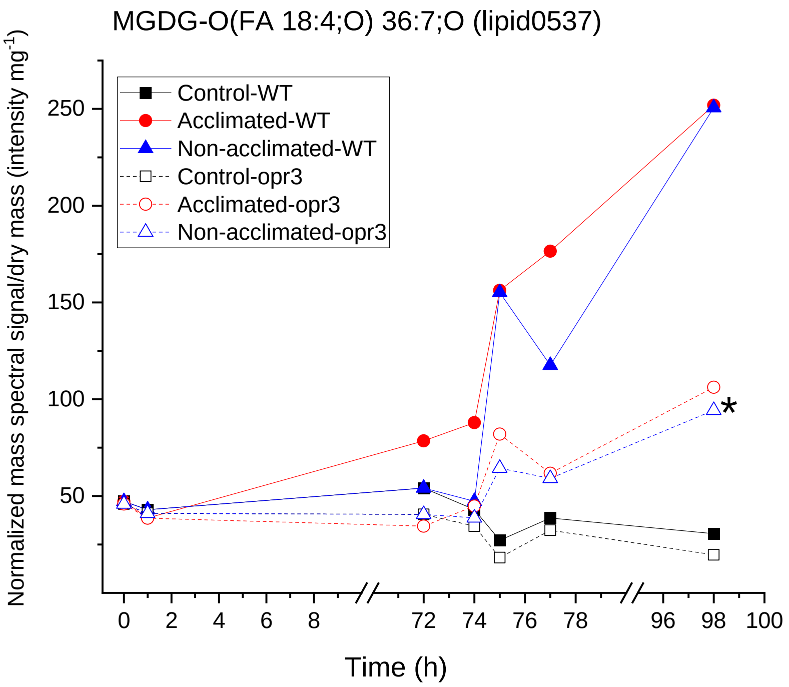
MGDG-O(FA 18:4;O) 36:7;O (lipid0537)
Control-WT
Acclimated-WT
Non-acclimated-WT
Control-opr3
Acclimated-opr3
Non-acclimated-opr3
Normalized mass spectral signal/dry mass (intensity mg-1)
250
200
150
100
50
0
2
4
6
8
72
74
76
78
96
98
100
Time (h)
*

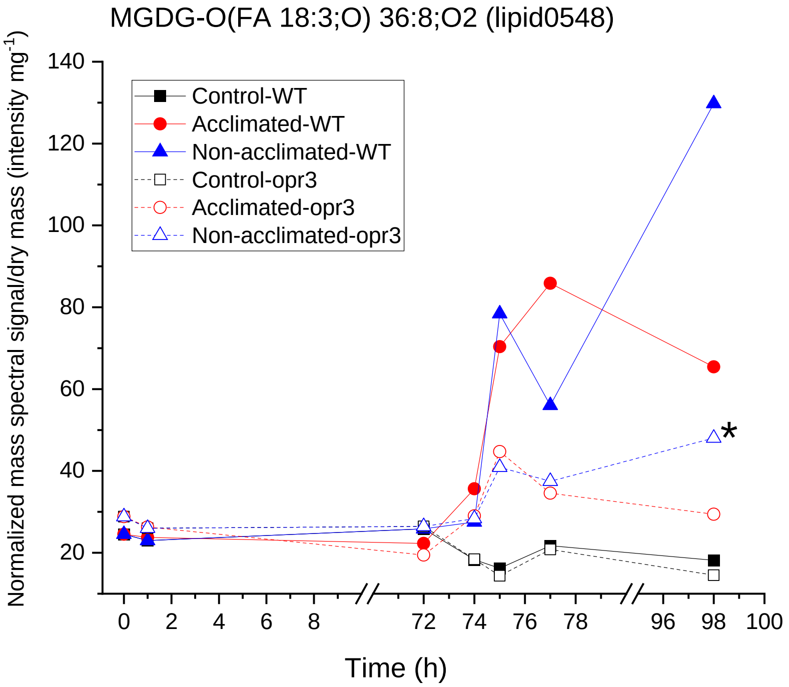
MGDG-O(FA 18:3;O) 36:8;O2 (lipid0548)
Control-WT
Acclimated-WT
Non-acclimated-WT
Control-opr3
Acclimated-opr3
Non-acclimated-opr3
Normalized mass spectral signal/dry mass (intensity mg-1)
140
120
100
80
60
40
20
0
2
4
6
8
72
74
76
78
96
98
100
Time (h)
*

10I

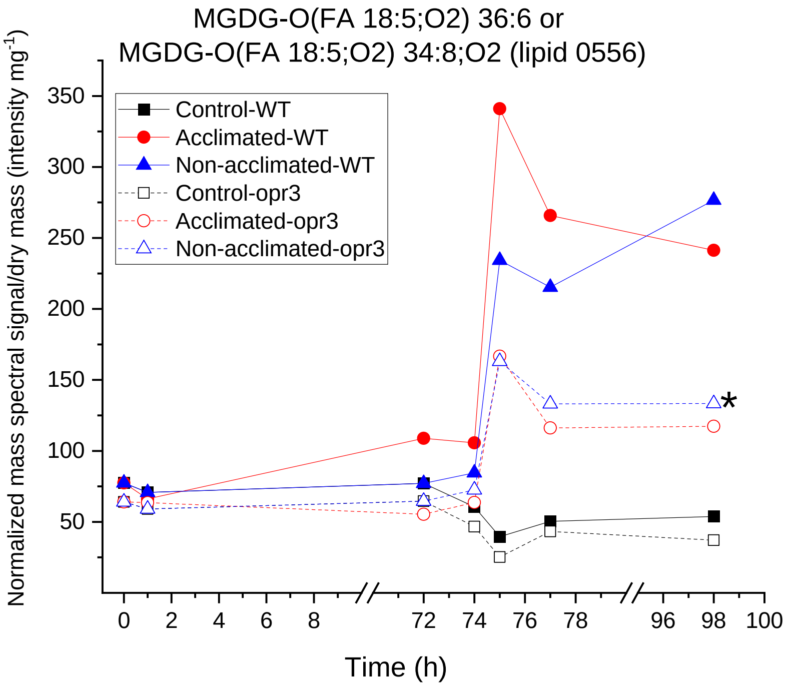
MGDG-O(FA 18:5;O2) 36:6 or
MGDG-O(FA 18:5;O2) 34:8;O2 (lipid 0556)
Normalized mass spectral signal/dry mass (intensity mg-1)
Time (h)
Control-WT
Acclimated-WT
Non-acclimated-WT
Control-opr3
Acclimated-opr3
Non-acclimated-opr3
*

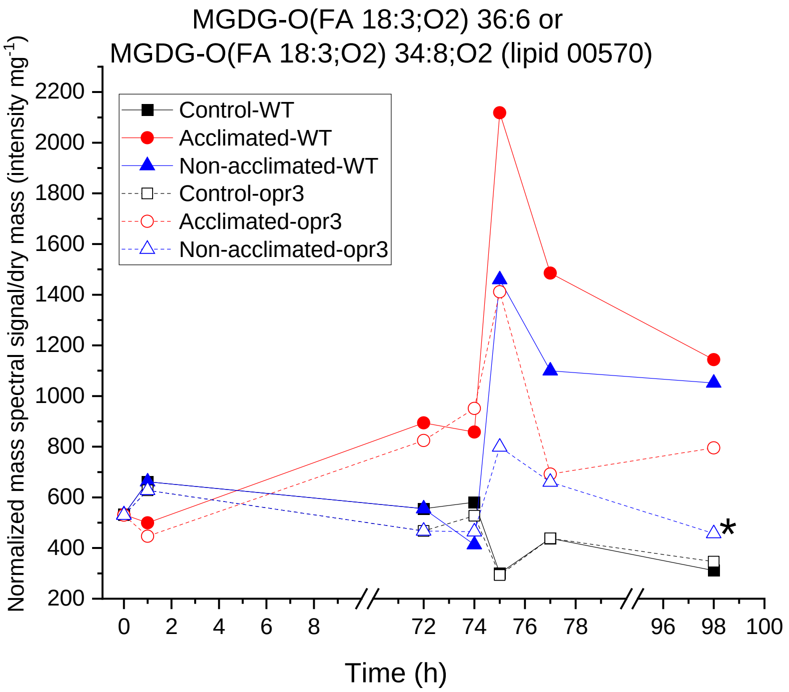
MGDG-O(FA 18:3;O2) 36:6 or
MGDG-O(FA 18:3;O2) 34:8;O2 (lipid 00570)
Normalized mass spectral signal/dry mass (intensity mg-1)
Time (h)
Control-WT
Acclimated-WT
Non-acclimated-WT
Control-opr3
Acclimated-opr3
Non-acclimated-opr3
*

10J

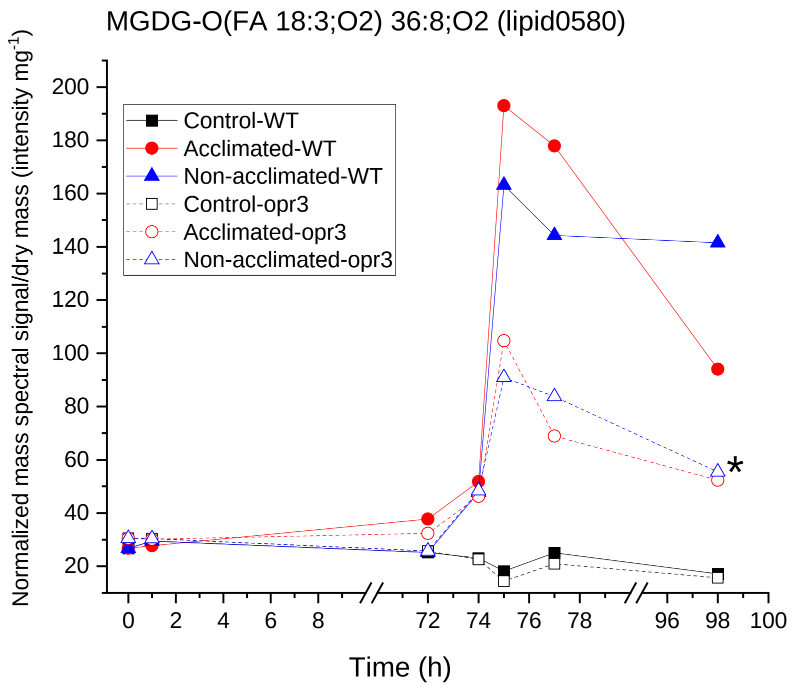
MGDG-O(FA 18:3;O2) 36:8;O2 (lipid0580)
Normalized mass spectral signal/dry mass (intensity mg-1)
Control-WT
Acclimated-WT
Non-acclimated-WT
Control-opr3
Acclimated-opr3
Non-acclimated-opr3
*
Time (h)

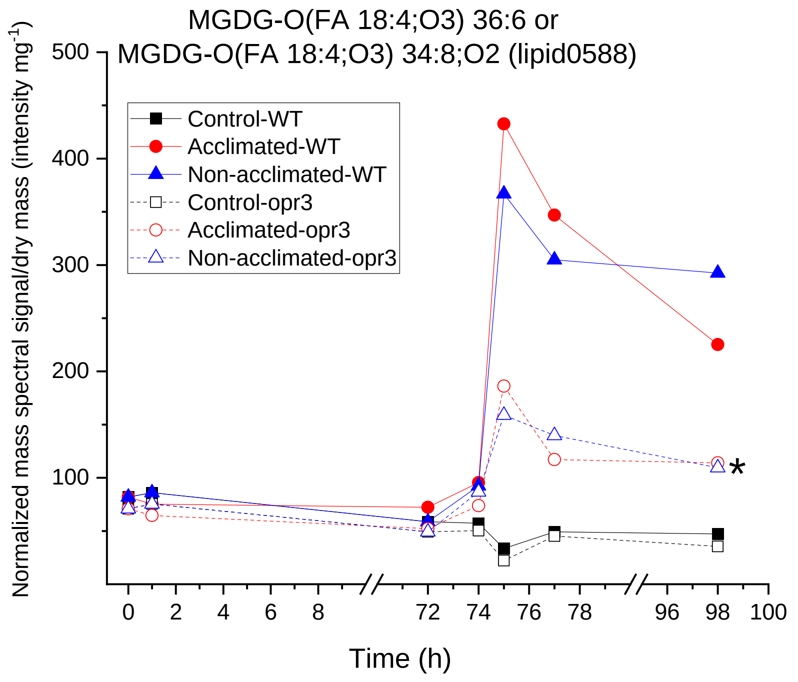
MGDG-O(FA 18:4;O3) 36:6 or
MGDG-O(FA 18:4;O3) 34:8;O2 (lipid0588)
Normalized mass spectral signal/dry mass (intensity mg-1)
Control-WT
Acclimated-WT
Non-acclimated-WT
Control-opr3
Acclimated-opr3
Non-acclimated-opr3
*
Time (h)

Sterol esters

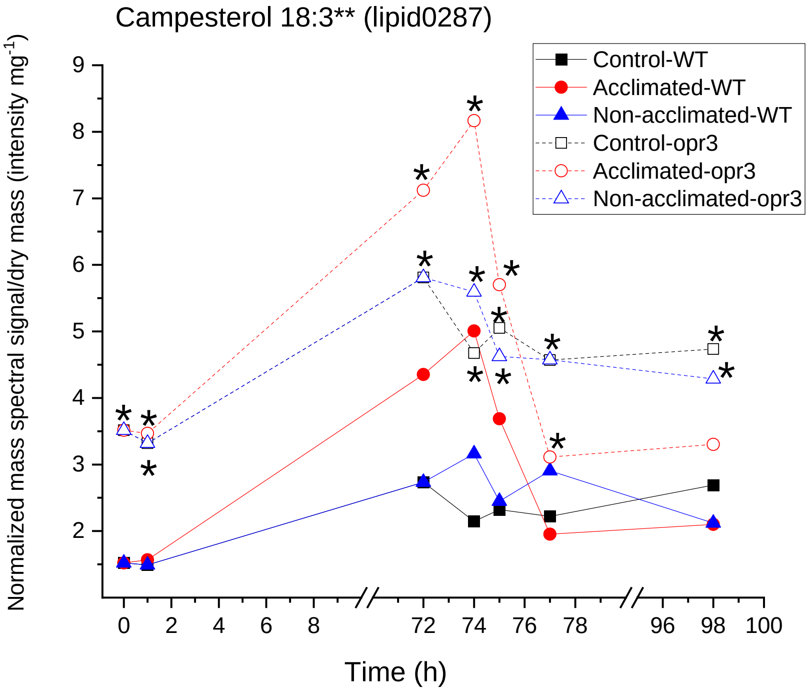

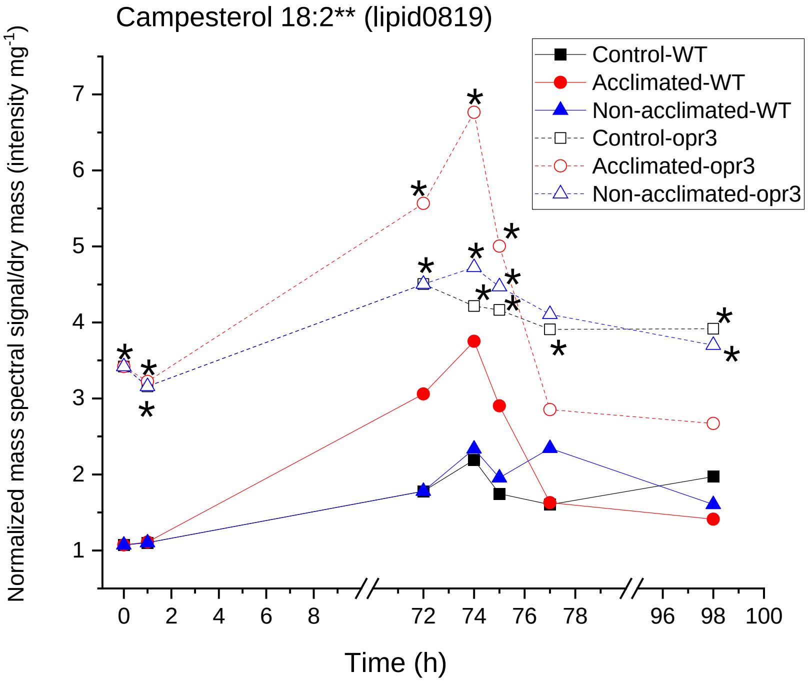

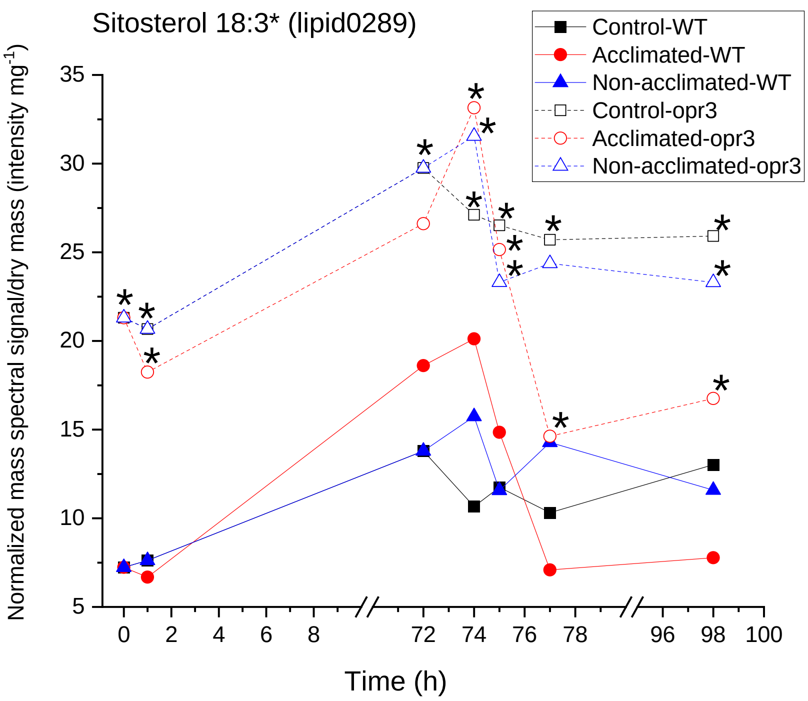
Sitosterol 18:3* (lipid0289)
Control-WT
Acclimated-WT
Non-acclimated-WT
Control-opr3
Acclimated-opr3
Non-acclimated-opr3
Normalized mass spectral signal/dry mass (intensity mg-1)
Time (h)

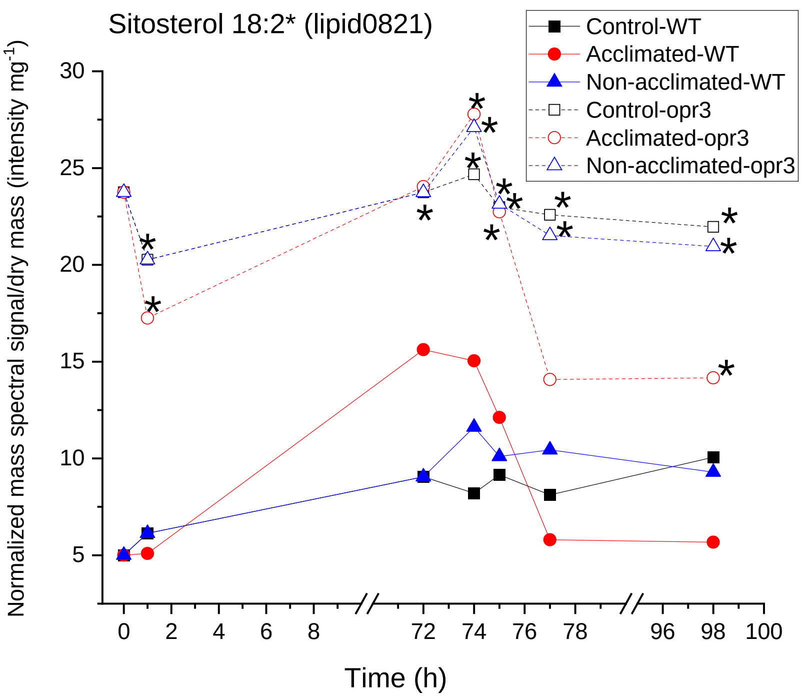
Sitosterol 18:2* (lipid0821)
Control-WT
Acclimated-WT
Non-acclimated-WT
Control-opr3
Acclimated-opr3
Non-acclimated-opr3
Normalized mass spectral signal/dry mass (intensity mg-1)
Time (h)

10M

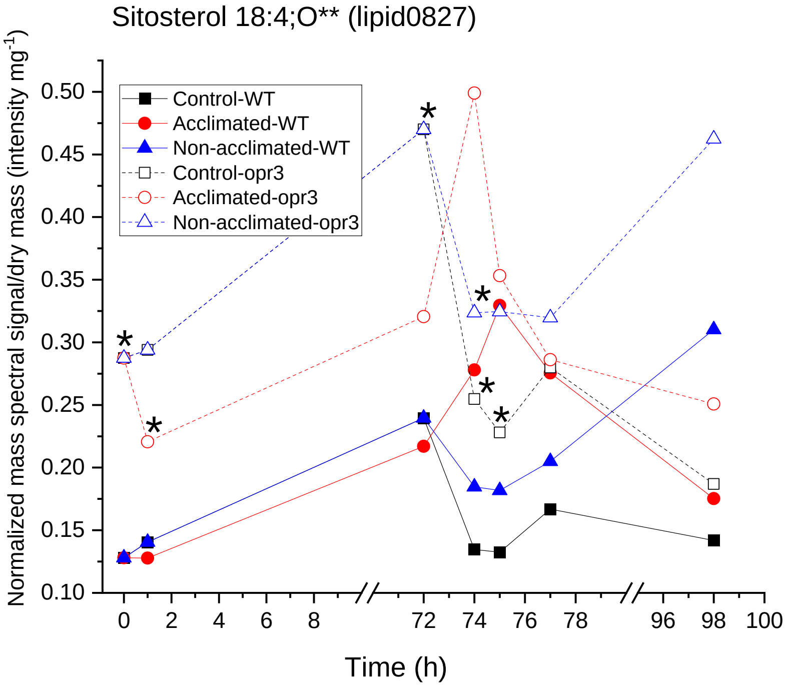
Sitosterol 18:4;O** (lipid0827)
Normalized mass spectral signal/dry mass (intensity mg-1)
Control-WT
Acclimated-WT
Non-acclimated-WT
Control-opr3
Acclimated-opr3
Non-acclimated-opr3
Time (h)

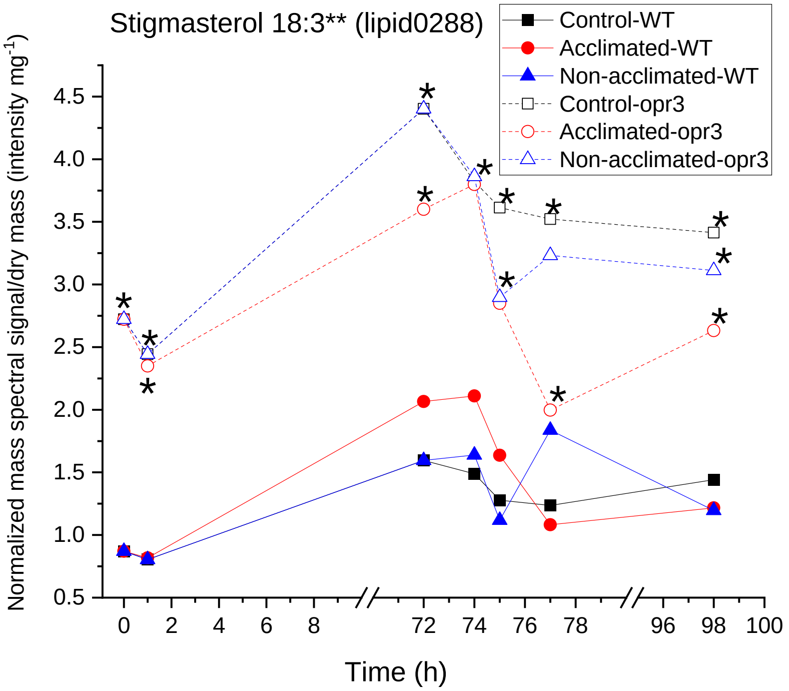
Stigmasterol 18:3** (lipid0288)
Normalized mass spectral signal/dry mass (intensity mg-1)
Control-WT
Acclimated-WT
Non-acclimated-WT
Control-opr3
Acclimated-opr3
Non-acclimated-opr3
Time (h)

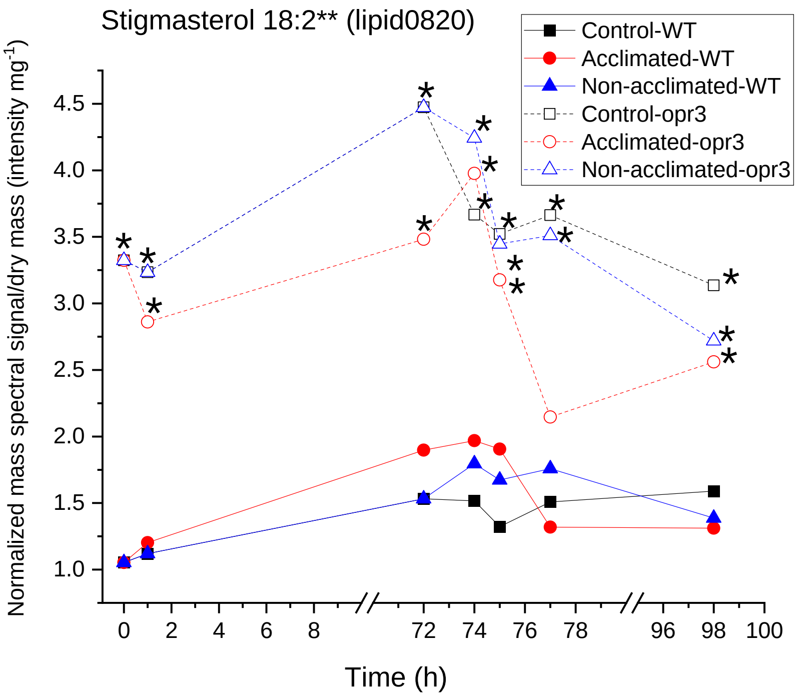

Stigmasterol 18:2** (lipid0820)
Control-WT
Acclimated-WT
Non-acclimated-WT
Control-opr3
Acclimated-opr3
Non-acclimated-opr3
Normalized mass spectral signal/dry mass (intensity mg^{-1})
Time (h)

Miscellaneous lipids

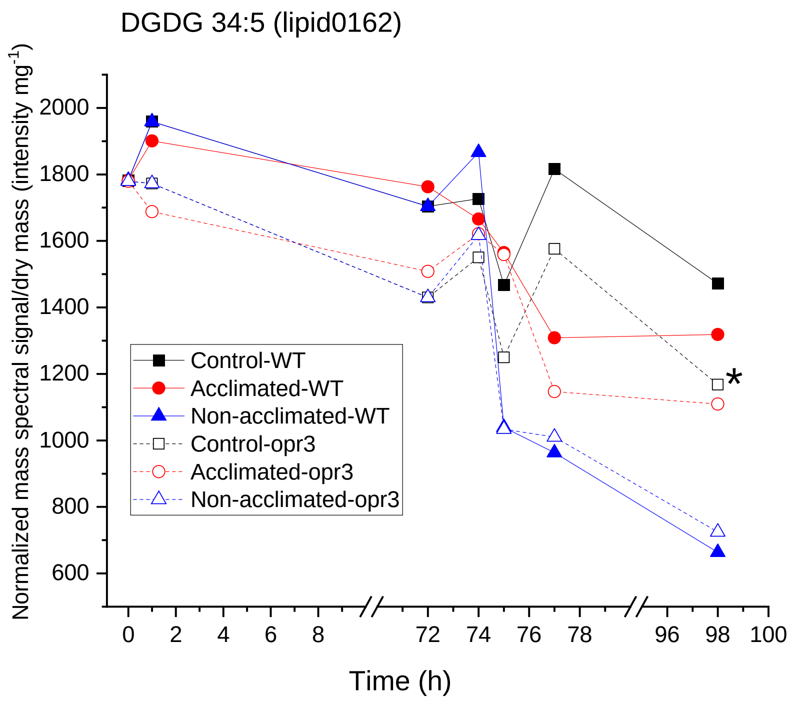

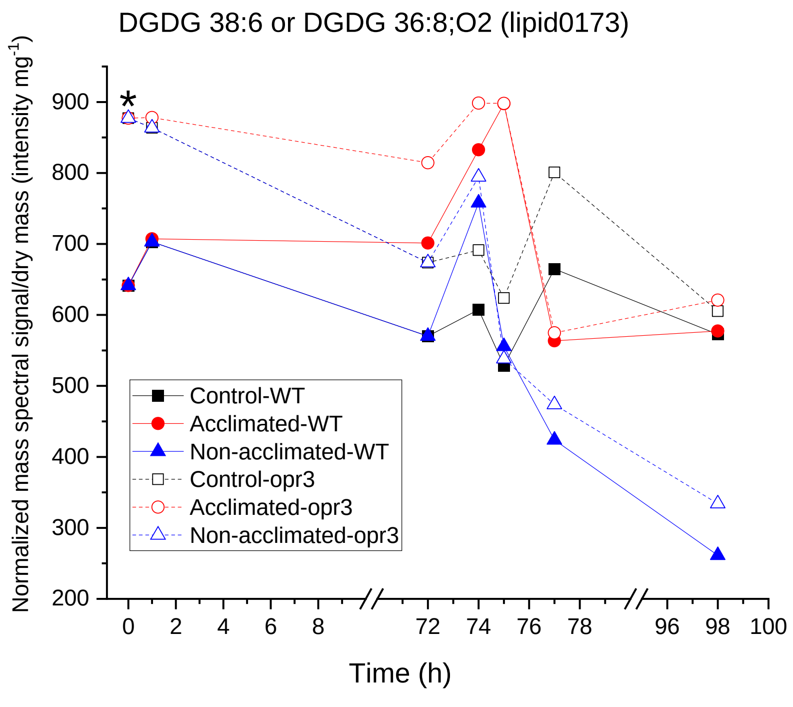

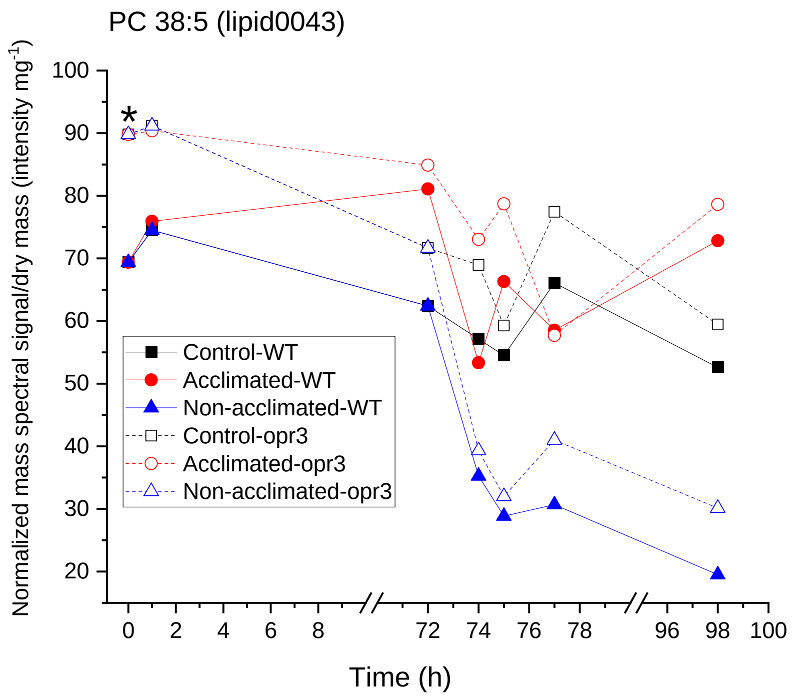
PC 38:5 (lipid0043)
Normalized mass spectral signal/dry mass (intensity mg-1)
100
90
80
70
60
50
40
30
20
0
2
4
6
8
72
74
76
78
96
98
100
Time (h)
Control-WT
Acclimated-WT
Non-acclimated-WT
Control-opr3
Acclimated-opr3
Non-acclimated-opr3
*

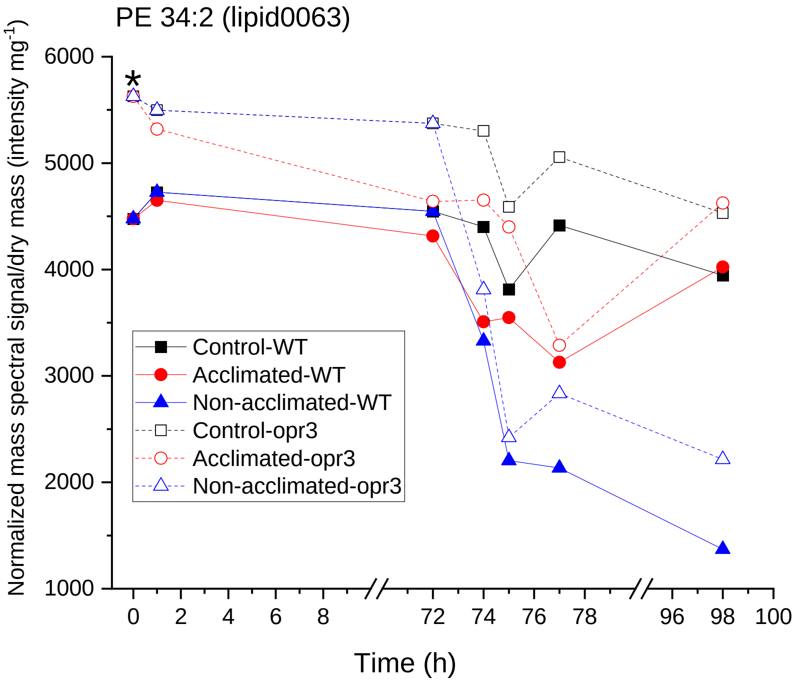
PE 34:2 (lipid0063)
Normalized mass spectral signal/dry mass (intensity mg-1)
6000
5000
4000
3000
2000
1000
0
2
4
6
8
72
74
76
78
96
98
100
Time (h)
Control-WT
Acclimated-WT
Non-acclimated-WT
Control-opr3
Acclimated-opr3
Non-acclimated-opr3
*

10Q

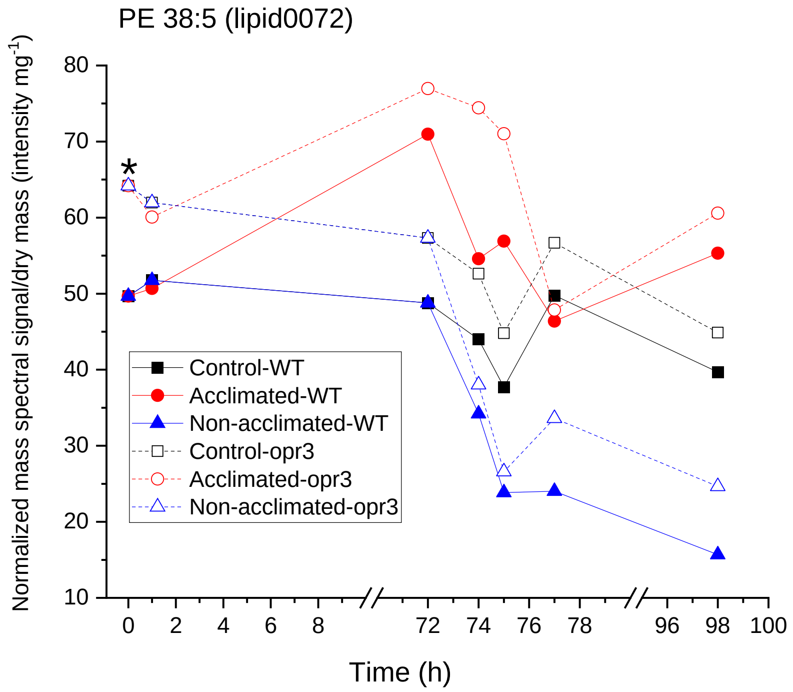
PE 38:5 (lipid0072)
Normalized mass spectral signal/dry mass (intensity mg-1)
80
70
60
50
40
30
20
10
*
Control-WT
Acclimated-WT
Non-acclimated-WT
Control-opr3
Acclimated-opr3
Non-acclimated-opr3
0
2
4
6
8
72
74
76
78
96
98
100
Time (h)

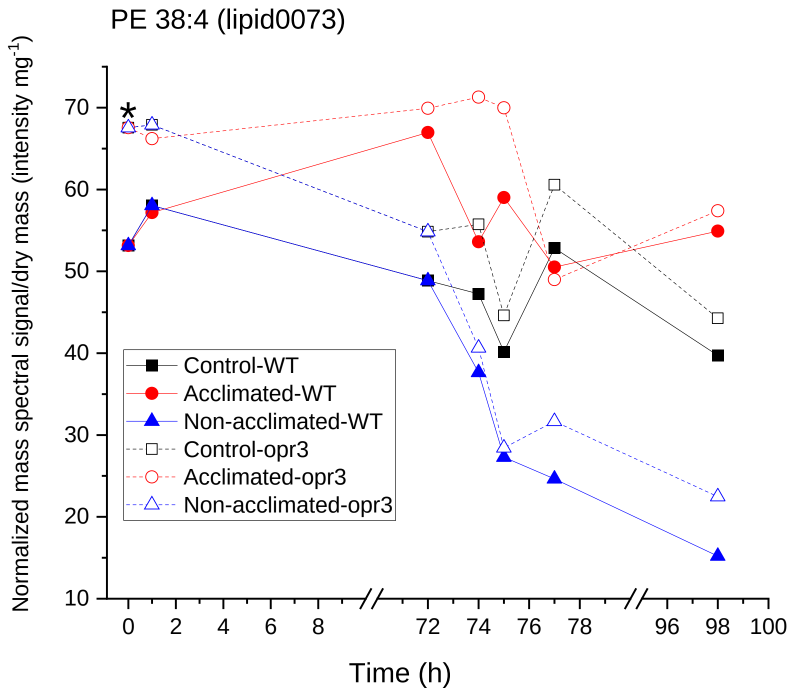
PE 38:4 (lipid0073)
Normalized mass spectral signal/dry mass (intensity mg-1)
70
60
50
40
30
20
10
*
Control-WT
Acclimated-WT
Non-acclimated-WT
Control-opr3
Acclimated-opr3
Non-acclimated-opr3
0
2
4
6
8
72
74
76
78
96
98
100
Time (h)

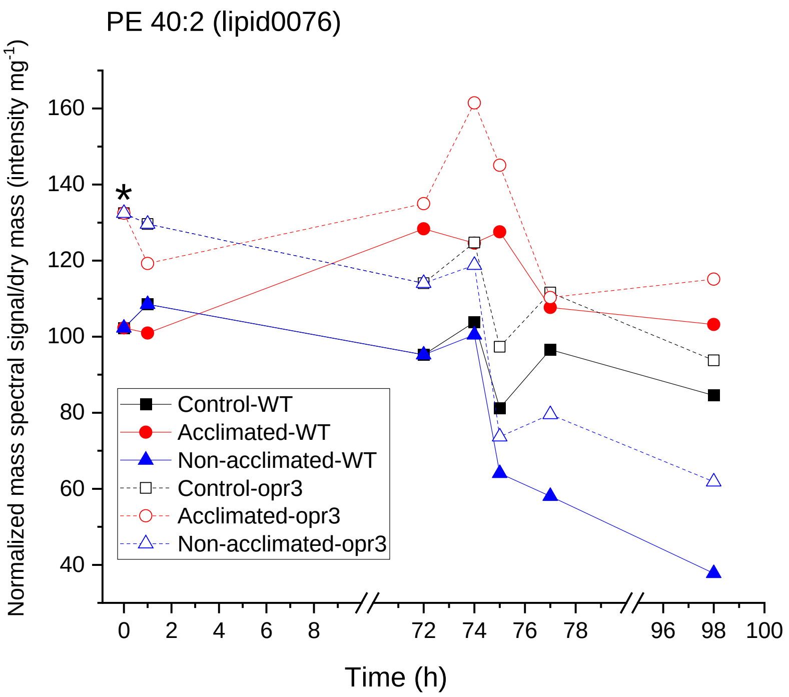
PE 40:2 (lipid0076)
Normalized mass spectral signal/dry mass (intensity mg-1)
Time (h)
Control-WT
Acclimated-WT
Non-acclimated-WT
Control-opr3
Acclimated-opr3
Non-acclimated-opr3
*

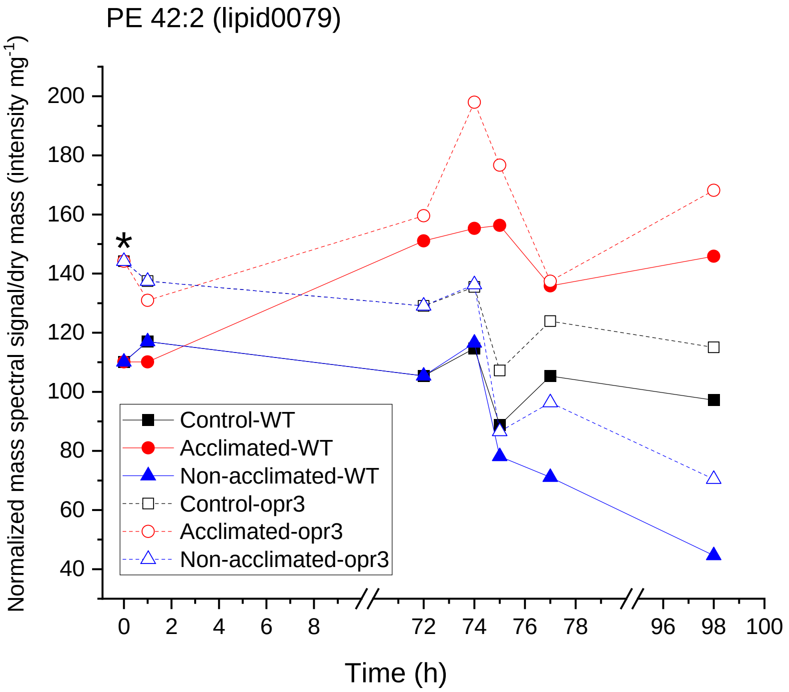
PE 42:2 (lipid0079)
Normalized mass spectral signal/dry mass (intensity mg-1)
Time (h)
Control-WT
Acclimated-WT
Non-acclimated-WT
Control-opr3
Acclimated-opr3
Non-acclimated-opr3
*

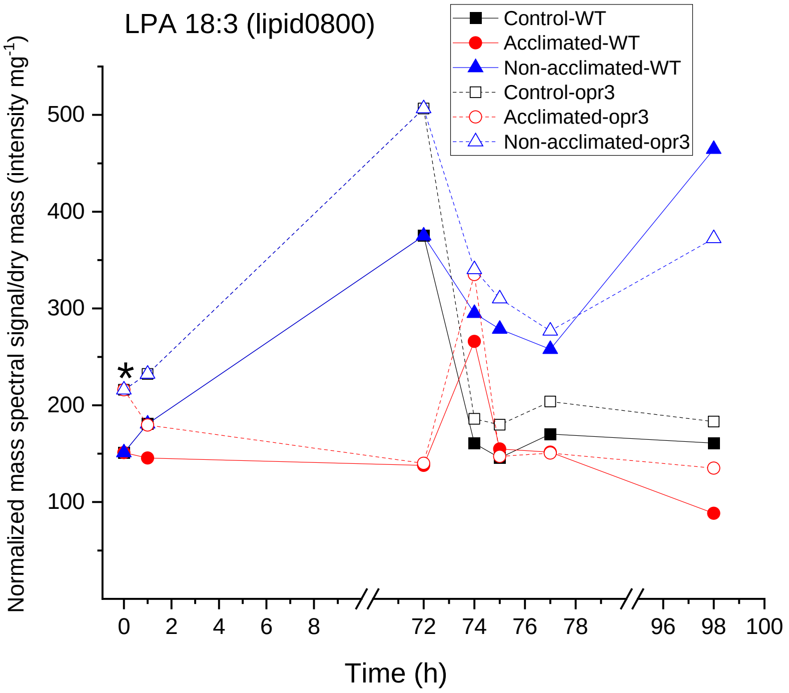
LPA 18:3 (lipid0800)
Control-WT
Acclimated-WT
Non-acclimated-WT
Control-opr3
Acclimated-opr3
Non-acclimated-opr3
Normalized mass spectral signal/dry mass (intensity mg-1)
100
200
300
400
500
*
0
2
4
6
8
72
74
76
78
96
98
100
Time (h)

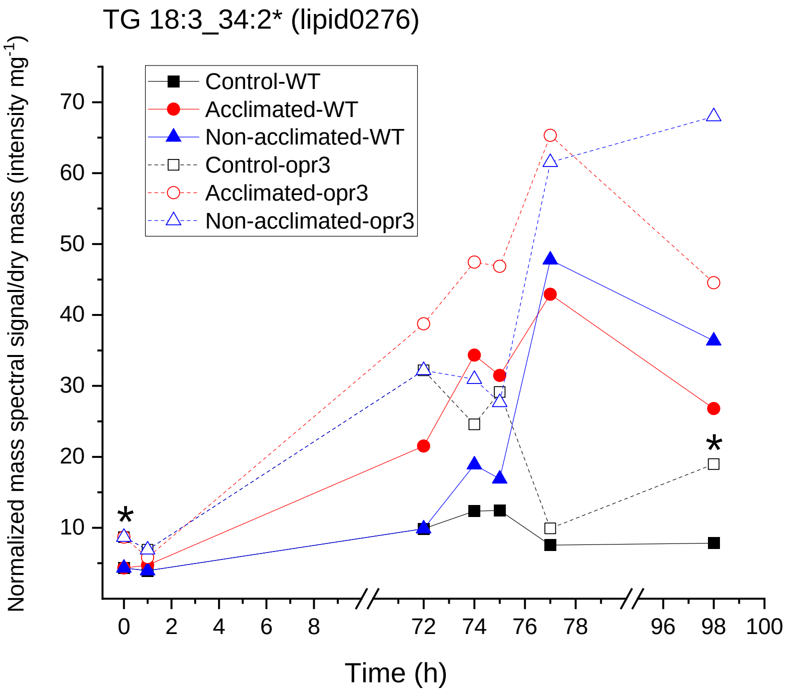
TG 18:3_34:2* (lipid0276)
Control-WT
Acclimated-WT
Non-acclimated-WT
Control-opr3
Acclimated-opr3
Non-acclimated-opr3
Normalized mass spectral signal/dry mass (intensity mg-1)
10
20
30
40
50
60
70
*
*
0
2
4
6
8
72
74
76
78
96
98
100
Time (h)

Figure S11. Positions of seeds/plants in trays. (A) Schematic numbering of wells within a 72-well plug tray. (B), (C), and (D) Positions of letter-coded seeds for all trays of rounds 1, 2, and 3, respectively.

A.

1	2	3	4	5	6	7	8	9	10	11	12
13	14	15	16	17	18	19	20	21	22	23	24
25	26	27	28	29	30	31	32	33	34	35	36
37	38	39	40	41	42	43	44	45	46	47	48
49	50	51	52	53	54	55	56	57	58	59	60
61	62	63	64	65	66	67	68	69	70	71	72

B.

H	V	K	G	I	T	Q	C	A	W	X	P
W	P	H	O	J	P	U	L	O	M	A	D
V	J	F	M	C	S	V	C	I	I	S	T
N	H	G	B	B	K	N	E	E	R	T	X
U	Q	R	X	W	A	K	L	G	D	F	Q
J	D	U	S	O	R	L	E	N	F	M	B

C.

P	L	E	U	X	Q	G	A	B	O	N	V
H	M	B	N	D	G	L	F	I	J	B	A
K	U	Q	F	T	W	T	I	P	V	X	L
Q	C	G	D	K	V	W	X	N	H	R	E
R	O	A	U	M	S	O	E	C	P	S	J
I	H	J	D	S	R	K	F	C	T	M	W

D.

M	Q	I	X	A	G	J	E	D	U	T	B
H	O	B	I	U	V	J	T	C	E	J	V
L	K	B	A	A	I	Q	E	D	M	O	P
F	X	L	R	X	W	N	N	M	F	S	C
W	U	G	G	R	P	H	T	S	K	Q	D
N	F	S	W	C	R	K	V	P	L	H	O

Figure S12. Numbering on tray F1.01. Each plant is referred to by a label including the tray label, e.g., “F1.01”, and the well position from 1 to 72. For example, the plants shown in this figure are F1.01.01 to F1.01.72, indicating F (for Freezing)Block.Tray.Position.

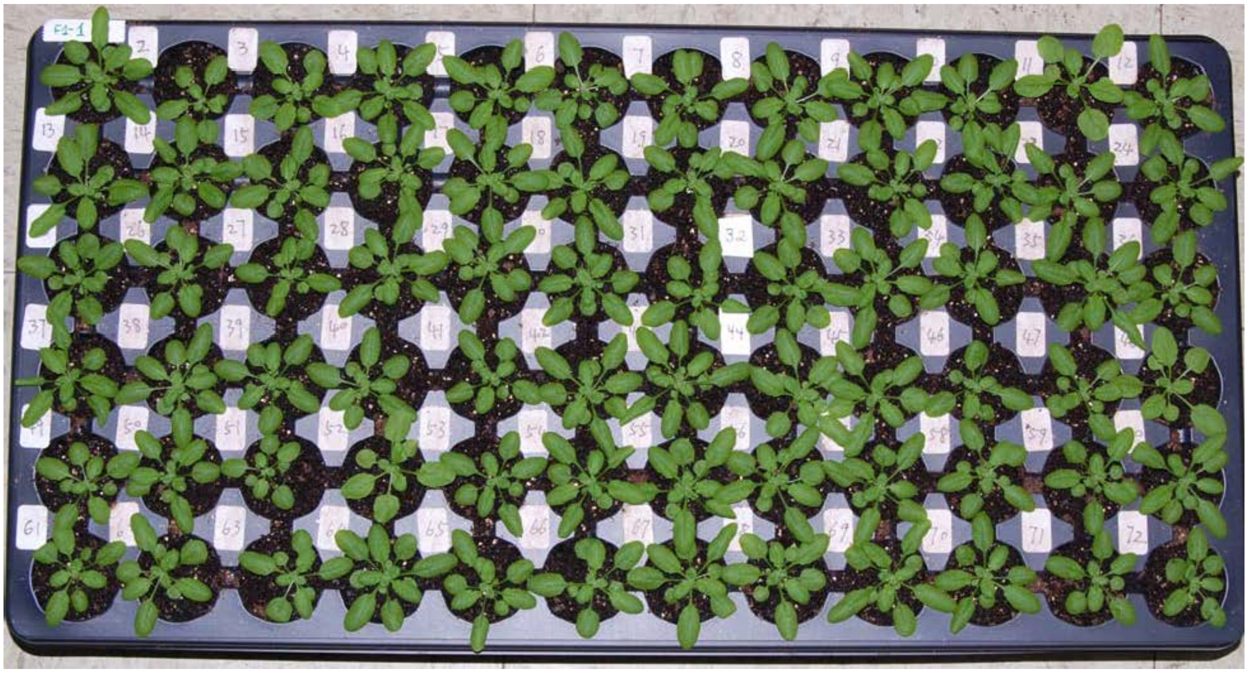

Supplement: Supplementary file 1 [file metabolites-12-00385-s001.zip › Supplemental Figures-Vu et al-3-16-22.pdf]
