# Supplementary material for: Specific Changes in Arabidopsis thaliana Rosette Lipids during Freezing Can Be Associated with Freezing Tolerance
Source: Metabolites. 2022 Apr 23;12(5):385. doi: 10.3390/metabo12050385 (PMC9145600; doi:10.3390/metabo12050385)
Supplement: Supplementary file 1 [file metabolites-12-00385-s001.zip › Supplemental Methods-Vu et al-4-5-22.pdf]

Supplemental methods

Arabidopsis lines

All *lox* and *ppla* lines (Table S7) were obtained from Arabidopsis Biological Resource Center (ABRC). Double mutants were obtained by crossing the lines and isolating F2 segregants homozogous for both mutant alleles. The *lox1 lox5* double mutant was previously described [1 at end of this document]. The *opr3-2* knockout line (X) was kindly shared by Jianmin Zhou. The *opr3-2* line (in Col-0 background with RAP-luciferase transgene inserted) was induced by EMS mutagenesis giving the G2471A base substitution resulting in replacement of Trp138 by a stop codon [2 at end of this document].

Sampling details

Two types of samples were collected from each plant (72 plants per tray): (1) leaves 5 and 6 in a 50-ml tube containing 25 ml of distilled water (Dillons Supermarket, Manhattan, KS, USA) and (2) the rest of the rosette in a 20-ml vial containing 4 ml of isopropanol with 0.01% butylated hydroxytoluene (BHT) at 75 °C. The two samples from each plant were each labeled to indicate the plant from which they were derived (for example, the two samples from plant 3 of tray F1.1 in Figure S12 were both labeled "F1.1.3"). The sample labels were printed on Tough Tag labels (Diversified Biotech, Dedham, MA, USA), which were used to label the 50-ml tubes and the 20-ml vials.

Harvesting was carried out on a cart carrying two heat blocks with the blocks removed to house the 20-ml vials (the vials rested in the area where the blocks normally rest). The thermal blocks were maintained at 75 °C. Other materials included four 40-slot racks to hold 72 tubes containing distilled water (two racks to hold tubes before harvesting, 2 racks to hold tubes after leaves 5 and 6 had been added). For trays 1, 2, 4, 6, 9, 10, 11, 12, 13, 14, 15, 16, and 17 (treatments which ended with a period at 21 °C), the rolling cart was positioned right next to the growth chamber (at room temperature). For trays 3 and 5, the cart was situated inside the cold room where the trays were treated (the heating block heaters were set at 90 °C to compensate for the cold air and still maintain the vials at 75 °C). For trays 7 and 8, the cart was positioned next to the freezing chamber in which the trays were frozen (harvesting occurred at room temperature). At the end of each of the indicated treatment periods (Figure 1), the leaf material from each corresponding tray was harvested simultaneously by four laboratory personnel so that the average harvesting time was less than 20 min per tray. The four personnel worked in two pairs; each pair had one "cutter" and one "dipper" who stood facing each other across the cart. The "cutter" procedure for each plant was: (1) cut the whole rosette off the roots, (2) cut leaves 5 and 6 and drop them into the gloved palm of the corresponding "dipper", and (3) drop the rest of the rosette into the corresponding pre-labeled, pre-heated 20-ml vial, cap the vial, shake the vial slightly to fully submerge the rosette, and return the vial to the heating block. The "dipper" procedure for each plant included: (1) receive leaves 5 and 6 from the corresponding "cutter", (2) rinse the leaves in a beaker of distilled water, and (2) drop the leaves into the pre-labeled 50-ml tube, cap the tube, and shake the tube to completely submerge the leaves in water.

For all trays except for trays 7 and 8, the tray was set on the cart during harvesting and the two pairs of personnel sequentially harvested the plants in order from 1 to 72. For trays 7 and 8, the trays were pre-cut (before seed sowing) into blocks of 4 plants with consecutive labels (e.g., 1-4, 5-8, 9-12…). At the end of the freezing treatment, the freezing chamber continued to maintain temperature at -8 °C. The blocks of 4 plants were taken out of the freezing chamber one at a time and were quickly harvested (two plants by each of the harvesting pair) before thawing occurred. The freezing chamber was opened and closed quickly; the temperature increased from -8 °C to -7 °C or -6 °C; the waiting plants remained frozen. In all cases, after the last plant of a tray was harvested, the vials were incubated at 75 °C for an additional 15 min and were allowed to cool to room temperature before being stored at -80 °C.

Leaf number analysis

Photos of each tray were taken at multiple times including: (1) immediately before fertilizing at 20 days old, (2) immediately before the last watering which occurred on the day before the tray was harvested (trays 1, 2, 4, 6, 9, 12, and 15) or treated (trays 3, 5, 7, 8, 10, 11, 13, 14, 16, and 17). In some cases, photos were also taken immediately before harvest. At each time point, a tray was photographed three times with three F-stop values (f/11, f/13, and f/14), using a Nikon D40 camera with an 18- 55 mm lens. Other camera parameters were: ISO 200, focal length 35-45 mm, exposure time 250 s^{-1}, built-in flash "ON". One high quality photo of each tray at each time point, of the three taken, was chosen for determining the number of leaves of each plant. The cotyledons were not counted in the total number of leaves. For an emerging leaf, it was only counted if the maximal width of the leaf was at least twice the width of the petiole.

References for Supplemental Methods

1. Nalam, V.J.; Keeretaweep, J.; Sarowar, S.; Shah, J. Root-derived oxylipins promote green peach aphid performance on Arabidopsis foliage. *Plant Cell* **2012,** *24*, 1643–1653.
2. Bi, D.; Cheng, Y.T.; Li, X.; Zhang, Y. Activation of plant immune responses by a gain-of-function mutation in an atypical receptor-like kinase. *Plant Physiol*. **2010**, *153*, 1771–1779.
